# How to describe a cryptic species? Practical challenges of molecular taxonomy

**DOI:** 10.1186/1742-9994-10-59

**Published:** 2013-09-27

**Authors:** Katharina M Jörger, Michael Schrödl

**Affiliations:** 1 Mollusca Section, SNSB-Bavarian State Collection of Zoology, Münchhausenstr 21, 81247 München, Germany; 2 Department Biology II, Ludwig-Maximilians-University, Großhaderner Str. 2, 82152 Planegg-Martinsried, Germany

## Abstract

**Background:**

Molecular methods of species delineation are rapidly developing and widely considered as fast and efficient means to discover species and face the 'taxonomic impediment’ in times of biodiversity crisis. So far, however, this form of DNA taxonomy frequently remains incomplete, lacking the final step of formal species description, thus enhancing rather than reducing impediments in taxonomy. DNA sequence information contributes valuable diagnostic characters and –at least for cryptic species – could even serve as the backbone of a taxonomic description. To this end solutions for a number of practical problems must be found, including a way in which molecular data can be presented to fulfill the formal requirements every description must meet. Multi-gene barcoding and a combined molecular species delineation approach recently revealed a radiation of at least 12 more or less cryptic species in the marine meiofaunal slug genus *Pontohedyle* (Acochlidia, Heterobranchia). All identified candidate species are well delimited by a consensus across different methods based on mitochondrial and nuclear markers.

**Results:**

The detailed microanatomical redescription of *Pontohedyle verrucosa* provided in the present paper does not reveal reliable characters for diagnosing even the two major clades identified within the genus on molecular data. We thus characterize three previously valid *Pontohedyle* species based on four genetic markers (mitochondrial cytochrome c oxidase subunit I, 16S rRNA, nuclear 28S and 18S rRNA) and formally describe nine cryptic new species (*P. kepii* sp. nov., *P. joni* sp. nov., *P. neridae* sp. nov., *P. liliae* sp. nov., *P. wiggi* sp. nov., *P. wenzli* sp. nov., *P. peteryalli* sp. nov., *P. martynovi* sp. nov., *P. yurihookeri* sp. nov.) applying molecular taxonomy, based on diagnostic nucleotides in DNA sequences of the four markers. Due to the minute size of the animals, entire specimens were used for extraction, consequently the holotype is a voucher of extracted DNA ('DNA-type’). We used the Character Attribute Organization System (CAOS) to determine diagnostic nucleotides, explore the dependence on input data and data processing, and aim for maximum traceability in our diagnoses for future research. Challenges, pitfalls and necessary considerations for applied DNA taxonomy are critically evaluated.

**Conclusions:**

To describe cryptic species traditional lines of evidence in taxonomy need to be modified. DNA sequence information, for example, could even serve as the backbone of a taxonomic description. The present contribution demonstrates that few adaptations are needed to integrate into traditional taxonomy novel diagnoses based on molecular data. The taxonomic community is encouraged to join the discussion and develop a quality standard for molecular taxonomy, ideally in the form of an automated final step in molecular species delineation procedures.

## Background

Species boundaries are frequently hard to delimit based on morphology only, a fact which has called for integrative taxonomy, including additional sources of information such as molecular data, biogeography, behavior and ecology [1,2]. Founding a species description on a variety of characters from different, independent datasets is generally regarded as best practice [3]. When species are considered as independently evolving lineages [4], different lines of evidence (e.g., from morphology, molecules, ecology or distribution) are additive to each other and no line is necessarily exclusive nor need different lines obligatory be used in combination [3,5]. Taxonomists are urged to discriminate characters according to their quality and suitability for species delineation, rather than to just add more and more data [5]. The specifics of the taxon in question will guide the way to the respective set(s) of characters that will provide the best backbone for the diagnosis. In cases of pseudo-cryptic species (among which morphological differences can be detected upon re-examining lineages separated e.g. on molecular data) or of fully cryptic species (that morphology fails to delimit), the traditional lines of evidence have to be modified by using, e.g., molecular information to break out of the 'taxonomic circle’ [6,7].

Cryptic species are a common phenomenon throughout the metazoan taxa, and can be found in all sorts of habitats and biogeographic zones [8-10]. Groups characterized by poor dispersal abilities (e.g., most meiofaunal organisms or animals inhabiting special regions where direct developers predominate, such as Antarctica), are especially prone to cryptic speciation [11,12]. Uncovering these cryptic species is fundamental for the understanding of evolutionary processes, historical biogeography, ecology, and also to conservation approaches, as distribution ranges that are smaller than initially assumed mean a higher risk of local extinction [8,10]. The lack of morphological characters to distinguish cryptic species should not lead to considerable parts of biological diversity remaining unaddressed.

The utility of DNA barcoding and molecular species delineation approaches to uncover cryptic lineages has been demonstrated by numerous studies (e.g., [11,13-19]). Unfortunately, inconsistencies in terminology associated with the interface between sequence data and taxonomy have led to confusion and various criticisms [6,20]. First of all, one needs to distinguish between species identification via molecular data (DNA barcoding in its strict sense) and species discovery [6,21,22]. While species identification is a primary technical application, species delimitation requires means of molecular species delineation that is either distance, tree or character based [6,23]. Under ideal circumstances sufficient material is collected from different populations across the entire distribution area of a putative group of cryptic species. Using population genetics the distribution of haplotypes can be analyzed and different, genetically isolated lineages can be detected [24]. Population genetic approaches are, however, not always feasible with animals that are rare or hard to collect, which might actually be a common phenomenon across faunas of most marine ecosystems [25-28]. Derived from barcoding initiatives, threshold based species delimitation became the method of choice, aiming for the detection of a 'barcoding gap’ between intra- and interspecific variation [29-31]. This approach has been criticized, however, due to its sensitivity to the degree of sampling, the general arbitrariness of fixed or relative thresholds, and to frequent overlap between intra- and interspecific variation [6,32,33]. In the recently developed Automatic Barcode Gap Discovery (ABGD) [34], progress has been made in avoiding the dependence of *a priori* defined species hypotheses in threshold based approaches, but reservations remain concerning the concept of a barcoding gap [25]. Several independent delineation tools exist, e.g. using haplotype networks based on statistical parsimony [35], maximum likelihood approaches applying the General Mixed Yule-Coalescent model [36,37], or Bayesian species delineation [38,39]. Empirical research currently compares the powers of these different tools on real datasets [25,32,40]. The effect of the inclusion of singletons in analyses is considered as most problematic [25]. At the present stage of knowledge, independent approaches allowing cross-validation between the different methods of molecular species delineation and other sources of information (morphology, biogeography, behavioral traits) seem the most reliable way of delimiting cryptic species [25].

The second inconsistency in terminology concerns usages of 'DNA taxonomy’. Originally, DNA taxonomy was proposed to revolutionize taxonomy by generally founding descriptions on sequence data and overthrowing the Linnaean binominal system [41]. Alternatively, it was suggested as a concept of clustering DNA barcodes into MOTUs [42]. Since then, however, it has been applied as an umbrella term for barcoding, molecular species delineation, and including molecular data in species descriptions (see e.g., [13,14,20,36,43,44]). In a strict sense, one cannot speak of molecular taxonomy if the process of species discovery is not followed by formal species description (i.e. there are two steps to a taxonomic process: species discovery (delimitation) and attributing them with formal diagnoses and names.) Taxonomy remains incomplete if species hypotheses new to science are flagged as merely putative by provisional rather than fully established scientific names. For practical reasons and journal requirements, most studies on molecular species delineation postpone formal descriptions of the discovered species (e.g., [13,14,25,33,36,40,43-46]), and then rarely carry them out later. DNA barcoding and molecular species delineation are promoted as fast and efficient ways to face the 'taxonomic impediment’, i.e. the shortage of time and personnel capable of working through the undescribed species richness in the middle of a biodiversity crisis [7,47,48]. However, keeping discovered entities formally unrecognized does not solve the taxonomic challenges but adds to them by creating parallel worlds populated by numbered MOTUs, OTUs or candidate species. In many cases the discovered taxa remain inapplicable to future research, thus denying the scientific community this taxonomic service, e.g. for species inventories or conservation attempts. Without formal description or a testable hypothesis, i.e. a differential diagnosis, 1) the discovered species might not be properly documented or vouchered by specimens deposited at Natural History Museums; and 2) their reproducibility can be hindered and confusion caused by different numbering systems. A deterrent example of the proliferation of informal epithets circulating as '*nomina nuda’* (i.e. species which lack formal diagnoses and deposited vouchers) in the literature is given by the 'ten species in one’ *Astraptes fulgerator* complex [31,49]. Thus, we consider it as all but indispensable for DNA taxonomy to take the final step and formalize the successfully discovered molecular lineages.

The transition from species delimitation to species description is the major task to achieve. Nearly ten years after the original proposal of DNA taxonomy [41], revolutionizing traditional taxonomy has found little acceptance in the taxonomic community, as most authors agree that there is no need for overthrowing the Linnaean System. Consequently, the challenge is to integrate DNA sequence information in the current taxonomic system. Several studies have attempted to include DNA data in taxonomic descriptions, albeit in various non-standardized ways; see the review by Goldstein and DeSalle ([21]; box 3): In some cases, DNA sequence information is simply added to the taxonomic description (in the form of GenBank numbers or pure sequence data), without evaluating and reporting diagnostic features [21]. Others rely on sequence information for the description, either reporting results of species delineation approaches, e.g. raw distance measurements or model based assumptions, or extracting diagnostic characters from their molecular datasets. There still is a consensus that species descriptions should be character based [50] (but see the Discussion below for attempts at model based taxonomy), and that tree or distance based methods fail to extract diagnostic characters [6]. Character based approaches, like the Characteristic Attribute Organization System (CAOS), are suggested as an efficient and reliable way of defining species barcodes based on discrete nucleotide substitution, and these established diagnostics from DNA sequences can be used directly for species descriptions as molecular taxonomic characters [51,52]. Yet, the application of CAOS or similar tools requires an evaluation of how to select and present molecular synapomorphies and how to formalize procedures to create a 'best practice’ linking DNA sequence information to existing taxonomy [20].

In the present study, we formally describe the candidate species of minute mesopsammic sea slugs in the genus *Pontohedyle* Golikov & Starobogatov (Acochlidia, Heterobranchia) discovered by Jörger et al. [25]. This cryptic radiation was uncovered in a global sampling approach with multi-gene and multiple-method molecular species delineation [25]. The initially identified 12 MOTUs, nine of which do not correspond to described species, are considered as species [following 4] resulting from a conservative minimum consensus approach applying different methods of molecular species delineation [25]. The authors demonstrated that traditional taxonomic characters (external morphology, spicules and radula features) are insufficient to delineate cryptic *Pontohedyle* species [25]. To evaluate the power of more advanced histological and microanatomical data, we first provide a detailed computer based 3D redescription of the anatomy of *Pontohedyle verrucosa* (Challis, 1970) and additional histological semi-thin sections of *P. kepii* sp. nov. In the absence of reliable diagnostic characters from morphology and microanatomy, we then rely on DNA sequence data as the backbone for our species descriptions. For the three previously valid *Pontohedyle* species we extract diagnostic characters using the Character Attribute Organization System (CAOS) based on four standard markers (mitochondrial cytochrome c oxidase subunit I, 16S rRNA, and nuclear 18S rRNA and 28S rRNA). In addition, nine new species are formally described on molecular characteristics and evidence from other data sources. Various approaches to the practical challenges for molecular driven taxonomy – such as critical consideration of the quality of the alignment, detection of diagnostic nucleotides and their presentation aiming for maximum traceability in future studies – are tested and critically evaluated.

## Results

### Evaluation of putative morphological characters

**Figure 1:**
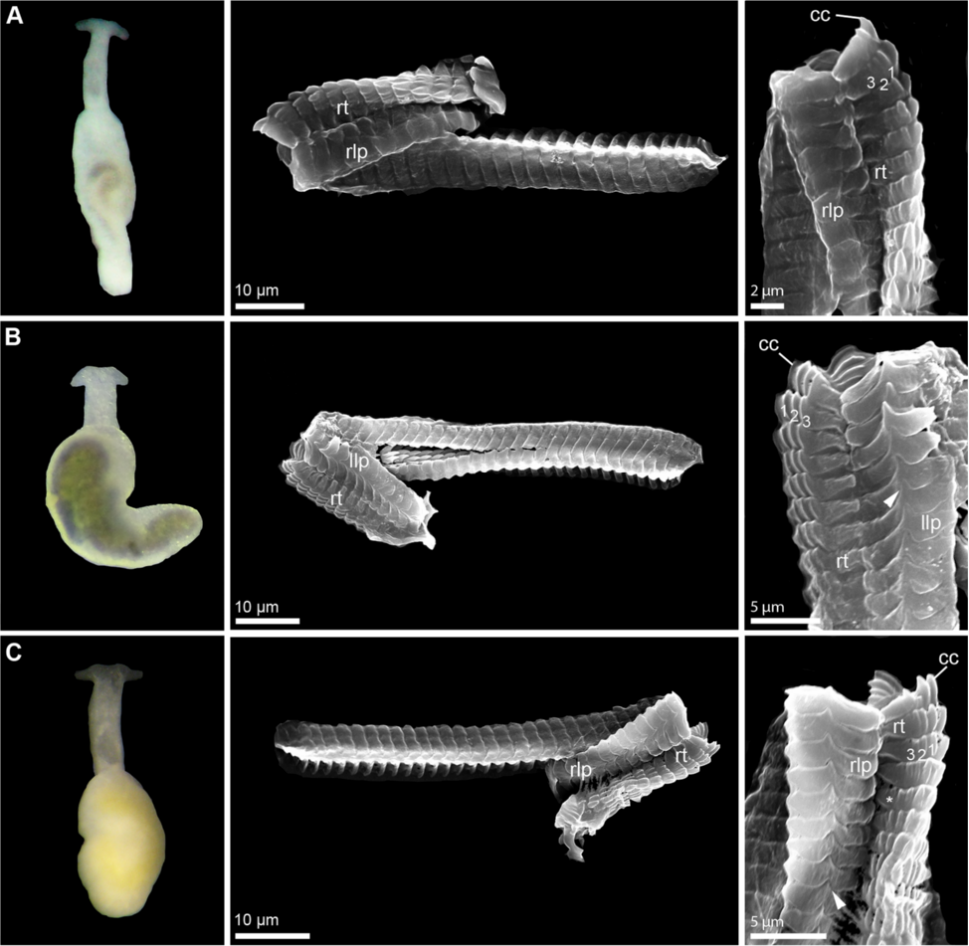
**External morphology (living specimens) and radula characteristics (SEM micrographs) in *Pontohedyle *species (part 1). A)***Pontohedyle kepii* sp. nov. (*Pontohedyle* sp. 1 in [25]); **B)***Pontohedyle joni* sp. nov. (*Pontohedyle* sp. 2 from WA-5 (Belize) in [25]); **C)***Pontohedyle liliae* sp. nov. (*Pontohedyle* sp. 4 in [25]), * marks putative 4^th^ cusp on rhachidian tooth. cc = central cusp of rhachidian tooth, llp = left lateral plate, rlp = right lateral plate, rt = rhachidian tooth.

**Figure 2:**
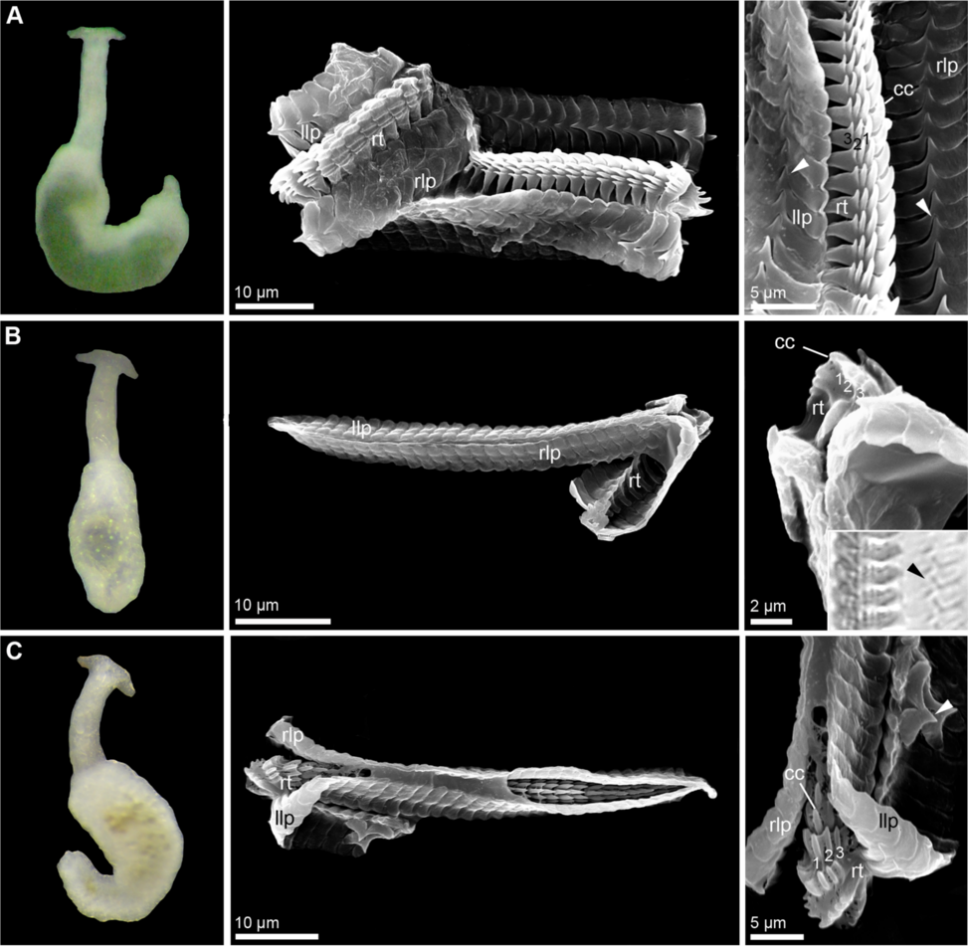
**External morphology (living specimens) and radula characteristics (SEM micrographs) in *Pontohedyle *species (part 2). A)***Pontohedyle peteryalli* sp. nov. (*Pontohedyle* sp. 7 in [25]); **B)***Pontohedyle wenzli* sp. nov. (*Pontohedyle* sp. 6, picture of living animal from WP-1 (holotype), radula from IP-2, see [25]); **C)***P. brasilensis* (living animal from WA-3 (Belize), radula from WA-10 (Brazil), see [25]). cc = central cusp of rhachidian tooth, llp = left lateral plate, rlp = right lateral plate, rt = rhachidian tooth.

The diversity within *Pontohedyle* revealed by molecular data cannot be distinguished externally: the body shows the typical subdivision into the anterior head-foot complex and the posterior visceral hump. Bodies are whitish-translucent, digestive glands are frequently bright green to olive green. Rhinophores are lacking, labial tentacles are bow-shaped and tapered towards the ends (see Figures 1 and 2). Monaxone rodlet-like spicules distributed all over the body and frequently found in an accumulation between the oral tentacles are characteristic for *Pontohedyle*. These spicules can be confirmed for *P. wenzli* sp. nov., for *P. yurihookeri* sp. nov., *P. milaschewitchii* (Kowalevsky, 1901) and *P. brasilensis* (Rankin, 1979), and, in contrast to the original description [53], also in *P. verrucosa*. No spicules could be detected in *P. peteryalli* sp. nov. from Ghana. The absence of spicules is insufficient, however, to delineate microhedylid species, since their presence can vary under environmental influence [54].

The radulae of eight species were investigated using SEM (see Figures 1 and 2). Radulae of *P. neridae* sp. nov., *P. martynovi* sp. nov. and *P. yurihookeri* sp. nov. were not recovered whole from molecular preparations, and thus were unavailable for further examination [25]. The radula of *P. wiggi* sp. nov. could only be observed under the light-microscope, but not successfully transferred to a SEM stub. All radulae are hook-shaped with a longer dorsal and a shorter ventral ramus, typical for Acochlidia. Radula formulas are 38–53 × 1.1.1, lateral plates are curved rectangular, and the rhachidian tooth is triangular and bears a central cusp and typically three smaller lateral denticles. Most radulae bear one pointed denticle centrally on the anterior margin of each lateral plate and a corresponding notch on the posterior side. Only the radula of *P. kepii* sp. nov. and *P. verrucosa* can be clearly distinguished from the others by the absence of this denticle and the more curved lateral teeth (see Figure 1A and [25], Figure 1D,E). Uniquely, *P. verrucosa* bears five lateral denticles next to the central cusp of the rhachidian tooth [25]; in *P. liliae* sp. nov. a tiny fourth denticle borders the central cusp (see * in Figure 1C).

**Figure 3:**
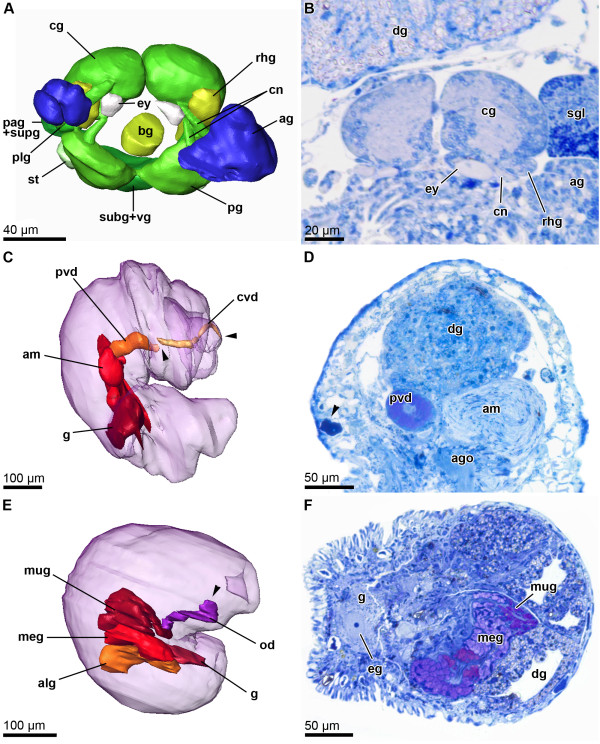
**Microanatomy of *P. verrucosa. *A)** 3D-reconstruction of the central nervous system, frontal view (ZSM Mol 20071832). **B)** Histological semi-thin section of the cerebral ganglia showing unpigmented eyes and rhinophoral ganglia. **C)** 3D-reconstruction of the male reproductive system in a partially retracted specimen, right lateral view (ZSM Mol 20071833). **D)** Histological semi-thin section showing prostatic vas deferens and sperm-filled ampulla (arrowhead = dark blue stained epidermal gland). **E)** 3D-reconstruction of the female reproductive system in a completely retracted specimen, right lateral view (ZSM Mol 20100548). **F)** Histological semi-thin section showing nidamental glands and gonad with oocyte.

Previous phylogenetic analyses [25] recovered a deep split into two *Pontohedyle* clades: the *P. milaschewitchii* clade and the *P. verrucosa* clade. This is supported by novel analyses in a larger phylogenetic framework and additionally including a second nuclear marker (18S rRNA) (own unpublished data). Since no detailed histological account exists of any representative from the large *P. verrucosa* clade, we redescribe *P. verrucosa* (based on ZSM Mol-20071833, 20071837 and 20100548), supplementing the original description with detailed information of the previously undescribed nervous and reproductive systems. The central nervous system (cns) of *P. verrucosa* lies prepharyngeal and shows an epiathroid condition. It consists of paired rhinophoral, cerebral, pleural, pedal and buccal ganglia and three unpaired ganglia on the visceral nerve cord, tentatively identified as left parietal ganglion, median fused visceral and subintestinal ganglion and right fused parietal and supraintestinal ganglion (Figure 3A). An osphradial ganglion or gastro-oesophagial ganglia were not detected. Anterior and lateral to the cerebral ganglia are masses of accessory ganglia. Due to the retracted condition of all examined specimens, tissues are highly condensed and no separation in different complexes of accessory ganglia could be detected. Attached to the pedal ganglia are large monostatolith statocysts. Oval, unpigmented globules are located in an antero-ventral position of the cerebral ganglia, interpreted as the remainder of eyes (see Figure 3B).

*P. verrucosa* is a gonochoristic species. The three sectioned specimens include two males and one female. The male reproductive system is comprised of gonad, ampulla, postampullary sperm duct, prostatic vas deferens, ciliated (non-glandular) vas deferens, genital opening and a small ciliated 'subepidermal’ duct leading to a second genital opening anterodorsally of the mouth opening (Figure 3C). The sac-like gonad is relatively small and bears few irregular distributed spermatozoa. The large tubular ampulla emerges from the gonad without a detectable preampullary sperm duct; it is loosely filled with irregularly distributed spermatozoa (Figure 3D). The ampulla leads into a short, narrow ciliated post-ampullary duct widening into the large tubular prostatic vas deferens (staining pink in methylene-blue sections, Figure 3D). Close to the male genital opening, the duct loses its glandular appearance and bears cilia. The primary genital opening is located on the right side of the body at the visceral hump and close to the transition with the head-foot complex. Next to the genital opening, the anterior vas deferens splits off as an inconspicuous subepithelial ciliated duct that leads anteriorly on the right side of the head foot complex. It terminates in a second genital opening between the oral tentacles anterodorsally from the mouth opening.

The female reproductive system consists of gonad, nidamental glands and oviduct (Figure 3E) and a genital opening located on the right side, in the posterior part of the visceral hump (not visible in Figure 3E, due to the retracted stage of the individual). The gonad is sac-like and bears one large vitellogenic egg (see Figure 3F) and several developing oocytes. Three histologically differentiated tube-like nidamental glands could be detected with a supposedly continuous lumen and with an epithelium bearing cilia. From proximal to distal these glands are identified as albumen gland (cells filled with dark blue stained granules), membrane gland (pinkish, vacuolated secretory cells) and winding mucus gland (secretory cells stained pink-purple). In its proximal part the distal oviduct shows a similar histology as the mucous gland, but then loses its glandular appearance. The epithelium of the distal oviduct bears long, densely arranged cilia.

Additional notable histological features are numerous dark-blue-stained epidermal gland cells (see e.g., arrowhead in Figure 3D) and refracting fusiform structures in the digestive gland (see Figure 3B). An additional series of histological semi-thin sections of *Pontohedyle kepii* sp. nov. was sectioned and brief investigation revealed no variation in the major organization of the organ systems in *Pontohedyle* as described herein and in previous studies [55,56].

### Remarks on the presentation of molecular characters

Diagnostic characters for each species of *Pontohedyle* were extracted using the 'Characteristic Attribute Organization System’ (CAOS) [51,57,58]. We define diagnostic characters as single pure characters, i.e. unique character states that respectively occur in all investigated specimens in a single *Pontohedyle* species but in none of the specimens of its congeners. As additional information single heterogeneous pure characters (i.e., different character states present within the species but absent from the congeners) are reported (for further details on the chosen approach see the Material and methods and Discussion sections). Positions refer to the position of the diagnostic nucleotide within the respective alignment (see Additional files 1, 2, 3, 4, 5 and 6). Where alignment positions differ from those in the deposited sequences, positions within the sequence of the holotype or in another reference sequence are also provided.

### Taxonomy of *Pontohedyle*

Family: Microhedylidae Odhner, 1938 [59]

Genus: *Pontohedyle* Golikov & Starobogatov, 1972 [60]

Synonymy: *Mancohedyle* Rankin, 1979; *Gastrohedyle* Rankin, 1979; *Maraunibina* Rankin, 1979

Type species (by subsequent designation): *Pontohedyle milaschewitchii* (Kowalevsky, 1901) [61]

Phylogenetic analyses of the genus *Pontohedyle*[25] confirmed earlier assumptions, that the three genera established by Rankin [62] (see above) present junior synonyms of *Pontohedyle*.

Morphological characteristics of genus *Pontohedyle*: Minute (0.7–6 mm) marine interstitial microhedylacean acochlid. Body divided into anterior head-foot complex and posterior visceral hump. In case of disturbance head-foot complex can be entirely retracted into visceral hump. Body whithish translucent. Foot with short rounded free posterior end. Head bears one pair of bow-shaped dorso-ventrally flattened oral tentacles. Rhinophores lacking. Monaxone, calcareous spicules irregularly distributed over head-foot complex and visceral hump. Radula hook-shaped band (lateral view), formula 1-1-1, lateral plates curved or with one pointed denticle, rhachidian tooth triangular with one central cusp and 2–4 lateral cusps on each side. Nervous system with accessory ganglia at cerebral nerves anterior to the cns. Sexes separate, male reproductive system aphallic, sperm transferred via spermatophores.

Molecular diagnosis of the genus *Pontohedyle*, based on the sequences analyzed herein (Table 1) and on sequences from a set of outgroups including all acochlidian genera for which data are available [63,64]. Positions refer to the alignments in Additional files 1 and 2, and to the reference sequences of *P. milaschewitchii*, ZSM Mol 20080054 (GenBank HQ168435 and JF828043) from Croatia, Mediterranean Sea (confirmed to be conspecific with material collected at the type locality in molecular species delineation approaches [25]). Molecular diagnosis is given in Table 2.

**Table 1:** **DNA sequence data analyzed in the present study to determine diagnostic nucleotides in *Pontohedyle***

**Species**	**Museums number**	**DNA voucher**	**GenBank accession numbers**			
			**18S rRNA**	**28S rRNA**	**16S rRNA**	**COI**
*P. milaschewitchii*	ZSM Mol 20071381	AB34404214	-	JQ410926	JQ410925	JQ410897
	ZSM Mol 20080054	AB34404241	HQ168435	JF828043	HQ168422	-
	ZSM Mol 20080055	AB34404239	-	-	JQ410927	-
	ZSM Mol 20080925	-	-	-	JQ410928	HQ168459
	ZSM Mol 20080953	AB35081832	KC984282	-	JQ410929	JQ410898
*P. brasilensis*	SI-CBC20 10KJ01-E03	AB34500510	KC984283	JQ410941	JQ410940	-
	SI-CBC20 10KJ01-B07	AB34402082	-	JQ410943	JQ410942	-
	SI-CBC20 10KJ01-D07	AB34500513	-	JQ410944	-	-
	SI-CBC20 10KJ01-B09	AB34402031	-	JQ410946	JQ410945	JQ410904
	SI-CBC20 10KJ01-C09	AB34500576	-	JQ410948	JQ410947	JQ410905
	SI-CBC20 10KJ01-A10	AB34402026	-	-	JQ410949	-
	SI-CBC20 10KJ02-E01	AB34402030	-	JQ410950	-	-
	ZSM Mol 20110723	AB34402034	KC984284	JQ410952	JQ410951	JQ410906
	ZSM Mol 20110722	AB34402086	KC984285	JQ410932	JQ410931	JQ410900
	ZSM Mol 20090198	AB35081813	KC984286	JQ410936	JQ410935	-
*P. verrucosa*	ZSM Mol 20071820	AB34404223	KC984287	JQ410978	JQ410977	JQ410920
	ZSM Mol 20080176	AB34404286	-	JQ410980	JQ410979	JQ410921
	ZSM Mol 20071135	AB34404221	KC984288	JQ410971	JQ410970	JQ410914
	ZSM Mol 20100388	AB34500547	-	-	-	JQ410916
	ZSM Mol 20100389	AB34402044	-	JQ410974	-	JQ410917
	ZSM Mol 20100390	AB34402070	-	JQ410975	-	JQ410918
	ZSM Mol 20100391	AB34500531	KC984289	-	JQ410976	JQ410919
*Pontohedyle kepii* sp. nov.	ZSM Mol 20081013	AB35081769	KC984290	JQ410967	JQ410966	JQ410912
*Pontohedyle joni* sp. nov.	ZSM Mol 20090197	AB34858164	KC984291	JQ410934	JQ410933	JQ410901
	SI-CBC20 10KJ01-D05	AB34402049	KC984292	-	JQ410937	JQ410902
	SI-CBC20 10KJ01-C08	AB34402065	-	JQ410939	JQ410938	JQ410903
*Pontohedyle neridae* sp.nov.	AM C. 476062.001	AB34500497	-	JQ410986	JQ410985	JQ410922
*Pontohedyle liliae* sp.nov.	ZSM Mol 20090471	AB35081802	KC984293	JQ410954	JQ410953	-
	ZSM Mol 20090472	AB35081838	-	JQ410956	JQ410955	-
*Pontohedyle wiggi* sp.nov.	ZSM Mol 20100595	AB34402059	-	JQ410960	JQ410959	JQ410908
	ZSM Mol 20100596	AB34402001	-	-	JQ410961	JQ410909
	ZSM Mol 20100597	AB34500571	-	JQ410963	JQ410962	JQ410910
	ZSM Mol 20100603	AB34402020	-	JQ410965	JQ410964	JQ410911
*Pontohedyle wenzli* sp.nov.	ZSM Mol 20100592	AB34402021	KC984294	JQ410958	JQ410957	JQ410907
	AM C. 476051.001	AB34402037	KC984295	JQ410982	JQ410981	-
	ZSM Mol 20081014	AB35081827	KC984296	JQ410969	JQ410968	JQ410913
	ZSM Mol 20100379	AB34500521	KC984297	JQ410973	JQ410972	JQ410915
*Pontohedyle peteryalli* sp. nov.	ZSM Mol 20071133	AB34404268	KC984298	-	JQ410930	JQ410899
*Pontohedyle martynovi* sp. nov.	AM C. 476054.001	AB34402062	-	JQ410984	JQ410983	-
*Pontohedyle yurihookeri* sp. nov.	ZSM Mol 20080565	AB34402000	KC984299	JQ410987	-	-

Museum numbers (ZSM – Bavarian State Collection of Zoology, SI – Smithsonian Institute, AM - Australian Museum), DNA vouchers (at ZSM) and GenBank accession numbers. 18S rRNA sequences generated in this study marked with *, all remaining sequences retrieved from GenBank.

**Table 2:** **Molecular diagnostic characters of *Pontohedyle***

**Marker**	**Diagnostic characters with position in alignment (in reference sequence)**
18S rRNA	165 (168), G; 1358 (1365), A; 1360 (1367), T; 1371 (1378), T; 1514 (1521), T
28S rRNA	260, C; 576, T; 622, T

### *Pontohedyle milaschewitchii* (Kowalevsky, 1901) [61]

*Hedyle milaschewitchii* Kowalevsky, 1901: p. 19–20 [61]

*Pontohedyle milaschewitchii* (Kowalevsky) – Golikov & Starobogatov [60]

*Mancohedyle milaschewitchii* (Kowalevsky) – Rankin (1979: p. 100) [62]

*Pontohedyle milatchevitchi* (Kowalevsky) – Vonnemann et al. (2005: p. 3) [65]; Göbbeler & Klussmann-Kolb (2011: p. 122) [66].

**Type locality:** Black Sea, bay of St George monastery near Sevastopol, Crimean Peninsula, Ukraine.

**Type material:** To our knowledge no type material remains. Nevertheless we refrain from designating a neotype, as there is no taxonomic need, i.e. no possibility of confusion in the species' area of distribution.

**Distribution and habitat:** Reported from the Black Sea and numerous collecting sites throughout the Mediterranean e.g. [55,61,67,68]; marine, interstitial, subtidal 1–30 m, coarse sand.

Molecular diagnosis is given in Table 3.

**Table 3:** **Molecular diagnostic characters of *Pontohedyle milaschewitchii***

**Marker**	**Diagnostic characters with position in alignment (in reference sequence)**	**Heterogeneous single pure positions**
18S rRNA	159, C; 164 (165), G	-
28S rRNA	329 (324), T	-
16S rRNA	8, G; 26, A; 145 (146), C; 203 (209), A; 243 (274), G; 275 (306), T; 290 (321), T; 333 (363), A; 352 (382), T	351 (381), T (G in ZSM Mol 20080953, position 381)
COI	11, C; 25, C; 58, T; 160, C; 272, A; 273,G; 319, T; 352, G; 371, G; 376, G; 397, A; 451, A; 476, C; 495, G; 496, G; 520, C	-
COI (AA)	4, L; 124, A; 159, L; 165, S	-

**Figure 4:**
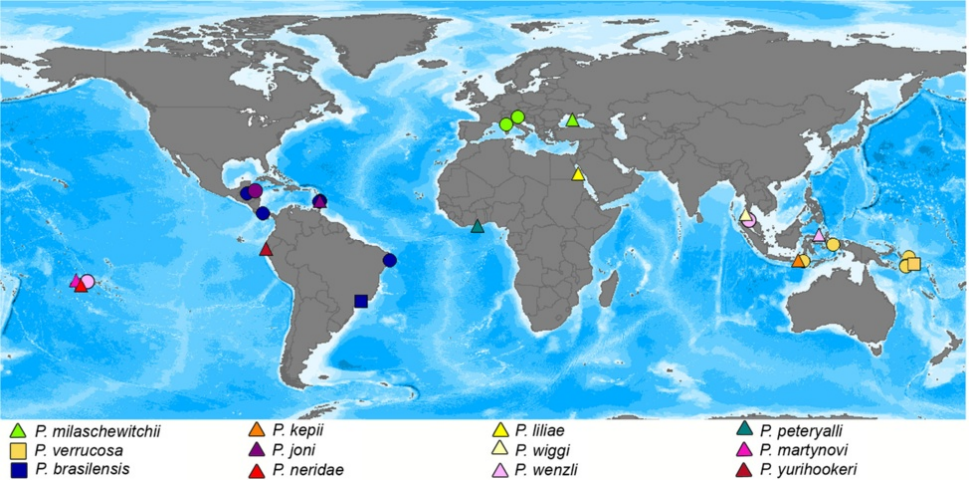
**World map showing the sampling sites and type localities of *Pontohedyle *species (modified after [**[25]**]).** Type localities with material included in this study are marked by triangles. Unsampled type localities are resembled by squares. Additional collecting sites are marked with dots.

ZSM Mol 20071381 (recollected at the type locality, see Figure 4) serves as the reference sequence, unless the sequence could not be successfully amplified. Then sequences (indicated below) from material from the Mediterranean serve as reference sequences (conspecifity was confirmed in a previous molecular species delineation approach 25]). Diagnostic characters in 18S rRNA were determined based on ZSM Mol 20080054 (GenBank HQ168435 = reference sequence) and ZSM Mol 20080953 (GenBank KC984282); in nuclear 28S rRNA based on ZSM Mol 20071381 (GenBank JQ410926) and ZSM Mol 20080054 (GenBank JF828043 = reference sequence), in mitochondrial 16S rRNA based on ZSM Mol 20071381 (GenBank JQ410925), ZSM Mol 20080054 (GenBank HQ168422), ZSM Mol 20080055 (GenBank JQ410927), ZSM Mol 20080925 (GenBank JQ410928) and ZSM Mol 20080953 (GenBank JQ410929), in mitochondrial COI based on ZSM Mol 20071381 (GenBank JQ410827), ZSM Mol 20080925 (GenBank HQ168459) and ZSM Mol 20080953 (GenBank JQ410898).

### *Pontohedyle verrucosa* (Challis, 1970) [53]

*Microhedyle verrucosa* Challis, 1970: pp. 37–38 [53]

*Pontohedyle verrucosa* (Challis) – Wawra (1987: p. 139) [69]

*Maraunibina verrucosa* (Challis) – Rankin (1979: p. 102) [62]

**Type locality:** Coarse, clean shell sand, a little above low water at neap tide, near southern end of Maraunibina Island, Marau Sound, East Guadalcanal, Solomon Islands.

**Type material:** According to Challis [53] in the Natural History Museum, London, and the Dominion Museum, Wellington, New Zealand. Own investigations revealed that the type material of Challis never arrived at the Natural History Museum, London and visiting the Museum of New Zealand Te Papa Tongarewa (former Dominion Museum), we were unable to locate any of her types. Thus, at current stage of knowledge, type material might only remain in her private collection. We refrain from designating a neotype because we were unable to recollect at the type locality (see below).

**Distribution and habitat:** Reported from Indonesia and the Solomon Islands [25,53]; marine, interstitial, intertidal, coarse sand.

**Sequenced material:** In a collecting trip to the Solomon Islands, we were unfortunately unable to recollect at the type locality (Maraunibina Island, East Guadalcanal), but successfully recollected in Komimbo Bay (West Guadalcanal), a locality, from which the describing author noted similar ecological parameters and recorded several meiofaunal slug species occurring at both sites [53,70] Additional material was collected at different collecting sites in Indonesia (see Figure 4).

Molecular diagnosis is given in Table 4.

**Table 4:** **Molecular diagnostic characters of *Pontohedyle verrucosa***

**Marker**	**Diagnostic characters with position in alignment (in reference sequence)**	**Heterogeneous single pure positions**
18S rRNA	-	-
28S rRNA	597 (605), T; 604 (612), G	-
16S rRNA	235, deletion; 243 (266), C; 249 (272), T; 330 (352), C	-
COI	118, A; 343, G; 367, C; 421, A; 451, C	541, T (C in ZSM 20080176, position 541)

ZSM Mol 20071820 (from Komimbo Bay, East Guadalcanal, Solomon Islands) serves as the reference sequence. Diagnostic characters in nuclear 18S rRNA were determined based on ZSM Mol 20071820 (GenBank KC984287), ZSM Mol 20071135 (GenBank KC984288) and ZSM Mol 20100391 (GenBank KC984289), in nuclear 28S rRNA based on ZSM Mol 20071820 (GenBank JQ410978), ZSM Mol 20080176 (GenBank JQ410980), ZSM Mol 20071135 (GenBank JQ410971), ZSM Mol 20100389 (GenBank JQ410974) and ZSM Mol 20100390 (GenBank JQ410975), in mitochondrial 16S rRNA based on ZSM Mol 20071820 (GenBank JQ410977), ZSM Mol 20080176 (GenBank JQ410979), ZSM Mol 20071135 (GenBank JQ410970) and ZSM Mol 20100391 (GenBank JQ410976) and in mitochondrial COIbased on ZSM Mol 20071820 (GenBank JQ410920), ZSM Mol 20080176 (GenBank JQ410921), ZSM Mol 20071135 (GenBank JQ410914), ZSM Mol 20100388 (GenBank JQ410916), ZSM Mol 20100389 (GenBank JQ410917), ZSM Mol 20100390 (GenBank JQ410918) and ZSM Mol 20100391 (GenBank JQ410919).

### *Pontohedyle brasilensis* (Rankin, 1979)

*Microhedyle milaschewitchii* (Kowalevsky) – *sensu* Marcus (1953: pp. 219–220) [71]

*Gastrohedyle brasilensis* Rankin, 1979: p. 101 [62]

*Pontohedyle milaschewitchii* (Kowalevsky) – *sensu* Jörger et al. (2007) [56], *partim*: all Western Atlantic specimens.

**Type locality:** Shell gravel, intertidal, Vila, Ilhabela, São Paulo, Brazil.

**Type material:** No type material remaining in Marcus’ collection (pers. comm. Luiz Simone). We nevertheless refrain from designating a neotype, since we lack material from the type locality.

**Distribution and habitat:** Caribbean Sea to southern Brazil [25,72]; marine, interstitial, intertidal to subtidal, coarse sand and shell gravel.

**Sequenced material:** Despite a series of recollecting attempts at the type locality and its vicinity in the past five years, we were unable to recollect any specimen of *Pontohedyle* in Southern Brazil. Our reference sequence refers to the southern-most specimen of a Western Atlantic *Pontohedyle* clade (see Figure 4), herein assigned to *P. brasilensis* (see Discussion). Additional material was collected at different collecting sites in the Caribbean (see Figure 4 for collecting sites and Figure 2C for photograph of a living specimen and SEM of radula).

Molecular diagnosis is given in Table 5.

**Table 5:** **Molecular diagnostic characters of *Pontohedyle brasilensis***

**Marker**	**Diagnostic characters with position in alignment (in reference sequence)**	**Heterogeneous single pure positions**
18S rRNA	164, T; 213 (225), G; 1693 (1706), T	-
28S rRNA	648 (654), A; 653 (659), T; 678, deletion, 679 (684), T; 683 (688), T; 704 (709), C; 801 (806), T	564 (570), T (in SI-CBC2010KJ01-B09 and ZSM 20090198: A); 793 (798) , C (in SI-CBC2010KJ02-E01: T, position 682)
16S rRNA	1, T; 11, deletion; 18 (17), A ; 80 (81), T; 102 (103), G; 107 (108), T; 131, G; 142, C; 172 (173), C; 182 (184), A; 210 (212), A; 214, deletion; 288 (306), G; 308 (325), C; 359 (376), C; 369 (386), G	-
COI	4, G; 16, C; 40, C; 44, G; 46, G; 68, G; 97, C; 101, C; 102, C; 167, G; 169, C; 170, T; 197, A; 202, G; 217, A; 227, G; 228, C; 239, T; 272, G; 287, A; 295, G; 310, C; 332, T; 351, deletion; 352, deletion; 353, deletion; 357 (354), A; 358( 355), G; 365 (362), T; 372 (369), T; 387 (384), C; 434 (431), G; 456 (453), G; 457 (454), G; 467 (464), G; 482 (479), T; 483 (480), G; 497(494), C; 499 (496), T; 512 (509), T; 518 (515), A; 529 (526), A; 535 (532), G; 542 (539), T; 543 (540), C; 566 (563), C; 619 (616), G; 635 (632), G	70, A (in ZSM Mol 20110722, G); 205, T (in ZSM Mol 20110722, C); 517, T (in ZSM Mol 20110722, C);
COI (AA)	4, I; 15, A; 23, V; 32, T; 34, P; 56, V; 57, L; 66, I; 76, A; 80, L; 91, A; 96, M; 111, L; 118, E; 119, deletion; 124 (123), F; 129 (128), A; 145 (144), V; 152 (151), W; 156 (155), A; 161 (160), W; 171 (170), L; 173 (172), I; 176 (175), L; 189 (188), L; 212 (211), V	-

Diagnostic characters in nuclear 18S rRNA were determined based on ZSM Mol 20110722 from Pernambuco, Brazil (GenBank KC984285 = reference sequence), ZSM Mol 20110723 (GenBank KC984284), SI-CBC2010KJ01-E03 (GenBank KC984283), ZSM Mol 20080198 (GenBank KC984286), in nuclear 28S rRNA based on ZSM Mol 20110722 (GenBank JQ410932); ZSM Mol 20090198 from St. Lucia Caribbean (GenBank JQ410936 = reference sequence); SI-CBC2010KJ01-E03 (GenBank JQ410941); SI-CBC2010KJ01-B07 (GenBank JQ410943), SI-CBC2010KJ01-D07 (GenBank JQ410944); SI-CBC2010KJ01-B09 (GenBank JQ410946), SI-CBC2010KJ01-C09 (GenBank JQ410948), SI-CBC2010KJ02-E01(GenBank JQ410950), ZSM Mol 20110723 (GenBank JQ410952); in mitochondrial 16S rRNA based on ZSM Mol 20110722 (GenBank JQ410931 = reference sequence); ZSM Mol 20090198 (GenBank JQ410935); SI-CBC2010KJ01-E03 (GenBank JQ410940); SI-CBC2010KJ01-B07 (GenBank JQ410942), SI-CBC2010KJ01-B09 (GenBank JQ410945), SI-CBC2010KJ01-C09 (GenBank JQ410947), SI-CBC2010KJ01-A10 (GenBank JQ410949), ZSM Mol 20110723 (GenBank JQ410951) and in mitochondrial COI based on ZSM Mol 20110722 (GenBank JQ410900 = reference sequence); SI-CBC2010KJ01-B09 (GenBank JQ410904); SI-CBC2010KJ01-C09 (GenBank JQ410905); ZSM Mol 20110723 (GenBank JQ410906).

### Descriptions of new *Pontohedyle* species

#### *Pontohedyle kepii* sp. nov.

*Pontohedyle sp.* 1 (MOTU I) in [25]

**Types:** Holotype: DNA voucher (extracted DNA in buffer, stored deep frozen at -80°C) ZSM Mol 20081013 (DNA bank accession number AB35081769). Paratypes: two specimens fixed in 96% ethanol were lost during DNA extraction. Two specimens fixed in glutaraldehyde and embedded in epoxy resin (ZSM 20080877 and 20080977). ZSM 20080877 sectioned at 1 μm. One additional specimen dissolved for radula preparation, SEM stub with radula available (ZSM Mol 20131101). All material collected at type locality.

**Type locality:** S 8°13′59“, E 117°28′32“; Pulau Moyo, Nusa Tengarra, Indonesia, Flores Sea, Indo Pacific (see Figure 4).

**ZooBank   registration:**   urn:lsid:zoobank.org:act:694022A2-BE21-4082-8CFD-A66094740A95

**Etymology:** Named after our good friend and long-time diving companion, Klaus-Peter ('Kepi’) Schaaf, who assisted us in collecting sand samples during diving in Indonesia.

**Distribution and habitat:** Currently known from type locality only; marine, interstitial, subtidal 5–6 m, coarse coral sand.

**Description:** morphologically with diagnostic characters of the genus *Pontohedyle* (see Figure 1A). Radula formula 1-1-1, rhachidian tooth with three lateral cusps, lateral plate smooth without denticle (Figure 1A).

Molecular diagnosis is given in Table 6.

**Table 6:** **Molecular diagnostic characters of *Pontohedyle kepii *** **sp. nov.**

**Marker**	**Diagnostic characters with position in alignment (in reference sequence)**
18S rRNA	199 (182), G; 202 (185), C; 203, deletion; 204, deletion; 206, deletion; 254 (244), T; 707 (697), T; 1355 (1345), A; 1356 (1346), C
28S rRNA	410 (439), T; 419 (448), C; 719 (754), G; 867 (902), C
16S rRNA	11, T; 184 (189), A; 187 (192), C; 239 (267), A; 242, deletion; 243, deletion; 244, deletion; 294 (324), G; 302 (328), G
COI	49, A; 79, T; 118, C; 148, C; 160, A; 193, G; 292, G; 331, G; 466, T; 494, G; 583, G; 628, A; 638, C
COI (AA)	165, D

Positions of the diagnostic characters refer to the sequence of the holotype. Diagnostic characters in nuclear 18S rRNA were determined based on GenBank KC984290, in 28S rRNA based on GenBank JQ410967, in mitochondrial 16S rRNA based on GenBank JQ410966, and in mitochondrial COI based on GenBank JQ410912.

### *Pontohedyle joni* sp. nov.

*Pontohedyle* sp. 2 (MOTU II) in [25]

**Types:** Holotype: DNA voucher (extracted DNA in buffer) ZSM Mol 20090197 (DNA bank accession number AB34858164). Paratype: one specimen fixed in 96% ethanol, collected with the holotype.

**Type locality:** N 14°3′34.56”, W 60°58′18.24”; near Castries, St. Lucia, Central America, Caribbean Sea, West Atlantic Ocean (see Figure 4).

**Additional material:** DNA voucher (extracted DNA in buffer) SI-CBC2010KJ01-D05 (DNAbank at ZSM AB34402049) and SEM preparation of radula (ZSM Mol 20131102) from N 16°48′13.44“, W 88°4′36.9“, and DNA voucher (extracted DNA in buffer) SI-CBC2010KJ01-C08 (DNAbank AB34402065) from N 16°48′7.62“, W 88°4′36.42“ both Carrie Bow Cay, Belize, Central America, Caribbean Sea, West Atlantic Ocean.

**ZooBank registration:** urn:lsid:zoobank.org:act:73AAC79D-5A43-40E4-B0D6-0329CAAA2AA0

**Etymology:** Named after Dr. Jon Norenburg to honor his efforts and enthusiasm for meiofaunal research and to thank him for his support for uncovering the largely unknown Caribbean meiofauna.

**Distribution and habitat:** Currently known from the Caribbean Sea (St. Vincent and Belize), type locality subtidal, 2–3 m depth, sand patches between seagrass, coarse sand. Additional material also subtidal, 14–15 m, sand patches between corals, coarse sand.

**Description:** morphologically with diagnostic characters of the genus *Pontohedyle*. Radula formula 48 × 1-1-1, rhachidian tooth with 3 lateral cusps, lateral plate with one pointed denticle (see Figure 1B).

Molecular diagnosis is given in Table 7.

**Table 7:** **Molecular diagnostic characters of *Pontohedyle joni *** **sp. nov.**

**Marker**	**Diagnostic characters with position in alignment (in reference sequence)**	**Heterogeneous single pure positions**
18S rRNA	207 (215), T; 209 (217), T; 256 (263), A	-
28S rRNA	443 (446), A; 547 (556), T; 868 (873), A	
16S rRNA	44 (47), C; 122 (125), T; 141 (144), A; 142 (145), G; 143 (146), G; 146, G; 152 (157), A; 182 (188), T; 236 (252), A; 259 (284), C	181 (187), T (in SI-CBC20 10KJ01-C08, C at position 187)
COI	31, A; 85, G; 160, G; 283, G; 298, G; 451, G; 523, C; 526, A; 578, C; 580, T	

The sequences retrieved from the holotype ZSM Mol 20090197 serve as reference sequences. Diagnostic characters in nuclear 18S rRNA were determined based on ZSM Mol 20090197 (GenBank KC984291) and SI-CBC2010KJ01-D05 (GenBank KC984292), in nuclear 28S rRNAbased on ZSM Mol 20090197 (GenBank JQ410934) and SI-CBC2010KJ01-C08 (GenBank JQ410939), in mitochondrial 16S rRNA based on ZSM Mol 20090197 (GenBank JQ410933), SI-CBC2010KJ01-D05 (GenBank JQ410937) and SI-CBC2010KJ01-C08 (GenBank JQ410938), and in mitochondrial COI based on ZSM Mol 20090197 (GenBank JQ410901), SI-CBC2010KJ01-D05 (GenBank JQ410902) and SI-CBC2010KJ01-C08 (GenBank JQ410903).

### *Pontohedyle neridae* sp. nov.

*Pontohedyle* sp. 3 (MOTU III) in [25]

**Types:** Holotype: DNA voucher (extracted DNA in buffer, stored deep frozen at -80°C) AM C. 476062.001 (DNA bank accession number at ZSM AB34500497). Paratype: one specimen fixed in 5% formalin and embedded in epoxy resin (AM C.476063.001), collected with the holotype.

**Type locality:** S 17°32′50.172”, W 149°46′35.4”; Motu Iti, Moorea, Oceania, Central Pacific Ocean (see Figure 4).

**ZooBank registration:** urn:lsid:zoobank.org:act:BE3E7920-5451-429D-95E4-C8D2F859C7CB

**Etymology:** Named after our friend and colleague, Dr. Nerida Wilson, with a big 'thank you’ for actively sharing with us the fascination for interstitial Acochlidia.

**Distribution and habitat:** Known from type locality only; subtidal 3-4 m, fine to medium coral sand.

**Description:** Morphologically with diagnostic characters of the genus *Pontohedyle*. Radula characteristics unknown.

Molecular diagnosis is given in Table 8.

**Table 8:** **Molecular diagnostic characters of *Pontohedyle neridae *** **sp. nov.**

**Marker**	**Diagnostic characters with position in alignment (in reference sequence)**
28S rRNA	61 (57), G; 522 (518), A
16S rRNA	11, G; 121 (123), T; 145 (147), T; 147 (149), G; 252 (276), C; 263 (286), T; 330 (352), G; 336 (358), G
COI	46, C; 151, C; 169, G; 220, A; 277, C; 278, T; 289, T; 391, C; 397, G; 421, C; 479, T; 505, A; 601, C

The sequences retrieved from the holotype serve as reference sequences. Diagnostic characters in nuclear 28S rRNA were determined based onAM C. 476062.001 (GenBank JQ410986), in mitochondrial 16S rRNA based on AM C. 476062.001 (GenBank JQ410985), and in mitochondrial COI based on AM C. 476062.001 (GenBank JQ410922).

### *Pontohedyle liliae* sp. nov.

*Pontohedyle* sp. 4 (MOTU IV) in [25]

**Types:** Holotype: DNA voucher (extracted DNA in buffer, stored deep frozen at -80°C) ZSM Mol 20090471 (DNA bank accession number AB35081802). Paratypes (all collected with the holotype): DNA voucher (extracted DNA in buffer) ZSM Mol 20090472 (DNA bank accession number AB35081838), one additional specimen used for radula preparation, SEM stub with radula available (ZSM Mol 20131103).

**Type locality:** N 24°11′50“, E 35°38′26“ (approximation from Google Earth), Sha’ab Malahi, Egypt, Africa, Red Sea (see Figure 4).

**ZooBank registration:** urn:lsid:zoobank.org:act:2711E3E5-1D1D-41B0-B919-7D7E690FD525

**Etymology:** Named after Reinhilde ('Lili’) Schmid, our friend and diving companion, who assisted us during sand collecting in Egypt and shares our fascination for this world of little creatures.

**Distribution and habitat:** Known from type locality only; subtidal 20 m, relatively fine coral sand.

**Description:** Morphologically with diagnostic characters of the genus *Pontohedyle*. Radula formula 45 × 1-1-1, rhachidian tooth with three (to four) lateral cusps, lateral plate with one pointed denticle (Figure 1C). Eyes clearly visibly externally, monaxone spicules in accumulation between oral tentacles and irregular all over the body.

Molecular diagnosis is given in Table 9.

**Table 9:** **Molecular diagnostic characters of *Pontohedyle liliae *** **sp. nov.**

**Marker**	**Diagnostic characters with position in alignment (in reference sequence)**
18S rRNA	33, C; 40, C; 54, G; 117, T; 129, T; 146 (147), C; 149 (150), T; 186 (187), C; 214 (223), A; 215 (224), C; 623 (631), T; 663 (673), T; 677 (687), C; 841 (853), G; 959 (971), G; 1028 (1040), T; 1030 (1042), C; 1348 (1360), A; 1363 (1375), T
28S rRNA	34 (30), C; 63 (59), C; 536 (532), T; 537 (533), G; 542, deletion; 555 (554), G; 590 (589), T; 642 (641), C; 643 (642), T; 658 (657), A; 671 (670), C; 696 (695), A; 827, G; 837, C; 902 (904), C
16S rRNA	10, C; 211 (222), C; 246 (277), C; 330 (359), T; 336 (365), C; 357 (386), C

The sequences retrieved from the holotype (ZSM Mol 20100471) serve as reference sequences. Diagnostic characters in nuclear 18S rRNA were determined based on ZSM Mol 20100471 (GenBank KC984293), in nuclear 28S rRNA based on ZSM Mol 20100471 (GenBank JQ410954) and ZSM Mol 20100472 (GenBank JQ410956), and in mitochondrial 16S rRNA based on ZSM Mol 20100471 (GenBank JQ410953) and ZSM Mol 20100472 (GenBank JQ410955).

### *Pontohedyle wiggi* sp. nov.

*Pontohedyle* sp. 5 (MOTU V) in [25]

**Types:** Holotype: DNA voucher (extracted DNA in buffer) ZSM Mol-20100595 (DNA bank accession number AB34402059). Paratypes (all collected with the holotype): DNA voucher (extracted DNA in buffer) ZSM Mol-20100596 (DNA bank AB34402001), ZSM Mol 20100597 (DNA bank AB34500571), ZSM Mol 20100603 (DNA bank AB34402020); one specimen fixed in glutaraldehyde and embedded in epoxy resin (ZSM Mol 20100598).

**Type locality:** N 7°36′15“, E 98°22′37“, Ko Raccha Yai, Phuket, Thailand, Andaman Sea, Indian Ocean (see Figure 4).

**ZooBank registration:** urn:lsid:zoobank.org:act:808E562E-0E1A-4D79-BB2C-1377B3734F86

**Etymology:** Named in memory of Ludwig ('Wigg’) Demharter, a malacologist friend, passionate diver, 'fun researcher’, and for many years a supporter of the ZSM and the second author's working group.

**Distribution and habitat:** Known from the type locality only; marine, interstitial between sand grains, relatively fine coral sand, subtidal 6–7 m depth, sandy slope among patches of corals.

**Description:** Morphologically with diagnostic characters of the genus *Pontohedyle*. Radula formula 1-1-1, lateral plate with one pointed denticle (as in *P. milaschewitchii*). Eyes visibly externally, monaxone spicules present.

Molecular diagnosis is given in Table 10.

**Table 10:** **Molecular diagnostic characters of *Pontohedyle wiggi *** **sp. nov.**

**Marker**	**Diagnostic characters with position in alignment (in reference sequence)**
28S rRNA	483 (472), T; 508 (497), T; 536, deletion; 537, deletion; 538, deletion; 699 (687), A
16S rRNA	180 (188), C; 374 (406), T
COI	127, C; 325, A; 583, C
COI (AA)	29, T

The sequences retrieved from the holotype (ZSM Mol 20090595) serve as reference sequences. Diagnostic characters in nuclear 28S rRNA were determined based on ZSM Mol 20100595 (GenBank: JQ410960), ZSM Mol 20100597 (GenBank: JQ410963), ZSM Mol 20100603 (GenBank: JQ410965), in mitochondrial 16S rRNA based on ZSM Mol 20100595 (GenBank: JQ410959), ZSM Mol 20100596 (GenBank: JQ410961), ZSM Mol 20100597 (GenBank: JQ410962), ZSM Mol 20100603 (GenBank: JQ410964), and in mitochondrial COI based on ZSM Mol 20100595 (GenBank: JQ410908), ZSM Mol 20100596 (GenBank: JQ410909), ZSM Mol 20100597 (GenBank: JQ410910), ZSM Mol 20100603 (GenBank: JQ410911).

### *Pontohedyle wenzli* sp. nov.

*Pontohedyle* sp. 6 (MOTU VIII) in [25]

**Types:** Holotype: DNA voucher (extracted DNA in buffer) ZSM Mol 20100379 (DNA bank accession number AB34500521).

**Type locality:** N 1°27′53“, E 125°13′48“, Lembeh Strait, Sulawesi, Indonesia, Banda Sea, West Pacific Ocean (see Figure 4).

Additional material DNA voucher (extracted DNA in buffer) ZSM Mol 20081014 (DNA bank accession number AB35081827) and one specimen used for SEM preparation of radula (available at ZSM Mol 20131105), locality S 8°23′58“, E 119°18′56“, Pulau Banta, Nusa Tengarra, Indonesia Flores Sea, Indo-Pacific. DNA voucher (extracted DNA in buffer) ZSM 20100592 (DNA bank AB34402021), locality N 7°36′15“, E 98°22′37“, Ko Raccha Yai, Phuket, Thailand, Andaman Sea, Indian Ocean. DNA voucher (extracted DNA in buffer) AM C. 476051.001 (DNA bank AB34402037) and one specimen fixed in 5% formalin and embedded in epoxy resin (AM C.476050.001), locality S 17°28′33.96”, W 149°49′51.6”, E of Cook’s Bay Pass, Moorea, Oceania, Central Pacific.

**Note:** Most species delineation approaches suggested ZSM 20100592, and some also AM C. 476051.001, as an independently evolving lineage [25]. Due to the conservative consensus approach, these specimens were included in the described species. Future analyses might show that their separation as independent species is warranted.

**ZooBank registration:** urn:lsid:zoobank.org:act:558EC548-1FB3-4B00-B248-4424CA7B098C

**Etymology:** Named after Alexander Wenzl, for his support during the development of this manuscript and his interest for meiofaunal research.

**Distribution and habitat:** Known from Indonesia, with putative distribution across the Indo-Pacific and Central Pacific; marine, subtidal (3–22 m), interstitial, coarse sand and shell grid.

**Description:** Morphologically with diagnostic characters of the genus *Pontohedyle*, eyes clearly visible externally (see Figure 2B, picture of living holotype). Radula 43 × 1-1-1, rhachidian tooth with three lateral cusps, lateral plate with pointed denticle (like in *P. milaschewitchii*).

Molecular diagnosis is given in Table 11.

**Table 11:** **Molecular diagnostic characters of *Pontohedyle wenzli *** **sp. nov.**

**Marker**	**Diagnostic characters with position in alignment (in reference sequence)**	**Heterogeneous single pure positions**
18S rRNA	771 (791), T; 772 (792), T	-
28S rRNA	449 (455), C; 539 (545), A	-
16S rRNA	36, G; 41, T; 84 (88), A; 143 (147), A; 144 (148), A; 161 (167), T; 176 (182), A; 194 (201), T; 207 (214), A; 256 (296), C; 258 (298), A; 269 (309), T; 295, deletion; 331 (369), A; 340 (378), A	332 (370), A (ZSM Mol 20081014, G at position 370)
COI	181, A; 218, G; 219, T; 296, T; 383, C; 430, T; 593, A	-
COI (AA)	73, V; 94, F; 122, A; 198, I	-

The sequences retrieved from the holotype (ZSM Mol 20100379) serve as reference sequences. Diagnostic characters in nuclear 18S rRNA were determined based on ZSM Mol 20100379 (GenBank KC984297), ZSM Mol 20081014 (GenBank KC984296), ZSM Mol 20100592 (GenBank KC984294), AM C. 476051.001 (GenBank KC984295), in nuclear 28S rRNA based on ZSM Mol 20100379 (GenBank JQ410973), ZSM Mol 20081014 (GenBank JQ410969), ZSM Mol 20100592 (GenBank JQ410958), AM C. 476051.001 (GenBank JQ410982), in mitochondrial 16S rRNA based ZSM Mol 20100379 (GenBank JQ410972), ZSM Mol 20081014 (GenBank JQ410968), ZSM Mol 20100592 (GenBank JQ410957), AM C. 476051.001 (GenBank JQ410981), and in mitochondrial COI based on ZSM Mol 20100379 (GenBank JQ410915), ZSM Mol 20081014 (GenBank JQ410913), ZSM Mol 20100592 (GenBank JQ410907).

### *Pontohedyle peteryalli* sp. nov.

*Pontohedyle* sp. 7 (MOTU VII) in [25]

**Types:** Holotype: DNA voucher (extracted DNA in buffer) ZSM Mol 20071133 (DNA bank accession number AB34404268). Paratypes (all collected with the holotype): eight specimens preserved in 96% ethanol (ZSM Mol 20070827); four in 75% ethanol (ZSM Mol 20070827), sixteen specimens fixed in glutaraldehyde, post-fixed in osmium and embedded in epoxy resin (ZSM Mol 20080453–60; ZSM Mol 20080462–69). SEM stub with radula available (ZSM Mol 20131104).

**Type locality:** N 04°47′46”, W 02°10′06”, MiaMia, Ghana, Africa, Gulf of Guinea, East Atlantic Ocean (see Figure 4).

Additional material: six specimens in 75% Ethanol collected at Nzema Cape, Ghana, Africa, Gulf of Guinea, East Atlantic Ocean; conspecifity still needs to be confirmed via barcoding.

**ZooBank registration:** urn:lsid:zoobank.org:act:B25E50F7-F0D2-4842-B6C3-5A79EA784A0C

**Etymology:** Named for our friend and malacologist, Peter ('Pete’) Ryall, who invited us to explore sea slugs right in front of his MiaMia home.

**Distribution and habitat:** Currently only known from the Ghana West Coast around MiaMia, marine, interstitial, subtidal 2-3 m, fine sand.

**Description:** Morphologically with diagnostic characters of the genus *Pontohedyle*. Radula 42 × 1-1-1, rhachidian tooth with three lateral cusps, lateral plate with pointed denticle (like in *P. milaschewitchii*), see Figure 2A.

Molecular diagnosis is given in Table 12.

**Table 12:** **Molecular diagnostic characters of *Pontohedyle peteryalli *** **sp. nov.**

**Marker**	**Diagnostic characters with position in alignment (in reference sequence)**
18S rRNA	160, C; 164, C
COI	14, T; 23, A; 48, C; 68, A; 76, C; 81, T; 83, A; 95, T; 101, A; 102, G; 140, A; 141, C; 167, A; 187, C; 209, C; 232, C; 280, A; 286, C; 293, A; 294, G; 357, C; 358, A; 361, A; 365, A; 373, A; 433, C; 448, G; 467, A; 468, T; 487, T; 503, T; 504, G; 512, A; 535, C; 556, C; 574, A; 586, C; 628, C; 634, C
COI (AA)	5, L; 8, I; 16, A; 23, I; 27, V; 28, T; 32, S; 34, S; 47, T; 56, I; 70, L; 119, T; 156, I; 162, D; 168, C; 171, I

The sequences retrieved from the holotype (ZSM Mol 20071133) serve as reference sequences. Diagnostic characters in nuclear 18S rRNA were determined based on GenBank KC984298, in mitochondrial 16S rRNA based GenBank JQ410930 and in mitochondrial COI based on GenBank JQ410899.

### *Pontohedyle martynovi* sp. nov.

*Pontohedyle* sp. 8 (MOTU IX) in [25]

**Types:** Holotype: DNA voucher (extracted DNA in buffer) AM C. 476054.001 (DNA bank accession number at ZSM AB34402062). Paratype: one specimen fixed in 5% formalin embedded in epoxy resin (AM C.476053.001), collected together with the holotype.

**Type locality:** S 17°28′17”, W 149°48′42”, E of Cook’s Bay Pass, Moorea, Oceania, Central Pacific Ocean (see Figure 4).

**ZooBank registration:** urn:lsid:zoobank.org:act:9431E4B8-EAF3-4E29-9993-BCD7C52928C6

**Etymology:** Named to thank our Russian friend and taxonomist, Alexander ('Sasha’) Martynov, for collecting acochlidians for us in many places, including *Pontohedyle milaschewitchii* at its type locality.

**Distribution and habitat:** Known from type locality only; marine, interstitial, subtidal 18–20 m, coarse sand, shell grid and rubble.

**Description:** Morphologically with diagnostic characters of the genus *Pontohedyle*. Radula characteristics unknown.

Molecular diagnosis is given in Table 13.

**Table 13:** **Molecular diagnostic characters of *Pontohedyle martynovi *** **sp. nov.**

**Marker**	**Diagnostic characters with position in alignment (in reference sequence)**
28S rRNA	539 (541), C; 623 (629), A
16S rRNA	8, deletion; 33 (32), T; 130 (131), C; 144, deletion; 151 (155), G; 168 (172), G; 171 (175), A; 218 (232), A; 230, T; 232 (244), G; 235 (258), C; 242 (274), C; 332 (365), C; 334 (367), G; 353 (386), G; 373 (408), G

The sequences retrieved from the holotype (AM C. 476054.001) serve as reference sequences. Diagnostic characters in nuclear 28S rRNA were determined based on GenBank JQ410984, and in mitochondrial 16S rRNA based on GenBank JQ410983.

### *Pontohedyle yurihookeri* sp. nov.

*Pontohedyle* sp. 9 (MOTU X) in [25]

**Types:** Holotype: DNA voucher (extracted DNA in buffer) ZSM Mol 20080565 (DNA bank accession number AB34402000).

**Type locality:** S 3°58′55”, W 80° 59′10”, Punta Sal, Peru, South America, East Pacific Ocean (see Figure 4).

**ZooBank registration:** urn:lsid:zoobank.org:act:9B858AA5-59FA-4505-AE94-FB2EA27FBEF6

**Etymology:** Named for our Peruvian friend and marine biologist, Yuri Hooker, who joined us during a great diving expedition to explore the Peruvian sea slug fauna.

**Distribution and habitat:** Known from type locality only; marine, interstitial, subtidal (8 m), coarse sand.

**Description:** Morphologically with diagnostic characters of the genus *Pontohedyle*. Radula characteristics unkown.

Molecular diagnosis is given in Table 14.

**Table 14:** **Molecular diagnostic characters of *Pontohedyle yurihookeri *** **sp. nov.**

**Marker**	**Diagnostic characters with position in alignment (in reference sequence)**
18S rRNA	163 (156), T; 200 (193), A; 213 (225), A; 770 (783), T; 810 (823), T
28S rRNA	110 (139), A; 398 (427), T; 399 (428), T; 403 (432), T; 409 (438), A; 410, deletion; 413 (441), G; 436 (464), T; 445, deletion; 446, deletion; 447 (473), C; 449 (475), A; 451 (477), A; 452 (478), A; 457 (483), A; 460 (486), T; 477 (503), C; 563 (593), T

The sequences retrieved from the holotype (ZSM Mol 20080565) serve as reference sequences. Diagnostic characters in nuclear 18S rRNA were determined based on GenBank KC984299, and in nuclear 28S rRNA based on GenBank JQ410987.

## Discussion

### Cryptic species challenging traditional taxonomy

Largely due to the development of molecular methods, research on cryptic species has increased over the past two decades [8,9], demonstrating their commonness across Metazoan taxa, though with random or non-random distribution among taxa and biomes still to be investigated [9,10]. Several recent studies have underlined that there is a large deficit in alpha taxonomy and that the diversity of marine invertebrates and especially meiofaunal animals might be much higher than expected, partly caused by high proportions of cryptic species e.g., [11,13,14,25,73-75]. Rather than global, amphi-Oceanic, circum-tropical or otherwise wide ranging, the distribution areas of the biological meiofaunal species involved may be regional and their ecology more specialized [12,25,76]. At an initial stage of molecular and ecological exploration, cryptic meiofauna is potentially threatened by global change and cannot effectively be included in conservation approaches.

In traditional taxonomy, most species descriptions are based on morphological and anatomical characters. Morphological species delineation, however, can fail to adequately address the diversity of life on Earth by leaving cryptic species unrevealed. Many taxonomists agree that the future of taxonomic descriptions should be integrative, embracing all available data sources (morphology, molecular sequences, biogeography, behavioral traits…) that can contribute to species delineation [1-3]. Previous authors have argued that 'integrative taxonomy’ does not necessarily call for a maximum of different character sets, but rather requires the taxonomist to select character sets adequate for species delineation in the particular group of taxa [3,5]. Thus, there should be no obligation in taxonomic practice to stick to morphology as the primary source [77], and there are no official requirements by the International Code of Zoological Nomenclature to do so [78,79].

The results of Jörger et al. [25] indicate that the members of *Pontohedyle* slug lineages are so extremely uniform that conventional taxonomic characters (i.e. external morphology, radula characteristics, spicules) fail to delineate species. A series of studies have demonstrated the generally high potential of advanced 3D-microanatomy for character mining in Acochlidia (e.g., [80-82]). However, the exclusively mesopsammic microhedylacean Acochlidia form an exception, as they show reduced complexity in all organ systems and uniformity that leaves few anatomical features for species delineation even on higher taxonomic levels [83]. Based on previous histological comparisons, Jörger et al. [56] were unable to find any morphological characters justifying discrimination between the closely related western Atlantic *P. brasilensis* and its Mediterranean congener, *P. milaschewitchii*. Here, we provided a detailed histological (re-)description using 3D-reconstruction based on serial semi-thin sections of *P. verrucosa*, to evaluate whether advanced 3D-microanatomy provides distinguishing morphological characters for the two generally accepted species, *P. milaschewitchii* and *P. verrucosa*, as representatives of the two major *Pontohedyle* clades (see [25], Figure 1)*.* Indeed, we revealed some putative distinguishing features in the reproductive and digestive systems (see Table 15). However, the encountered (minor) morphological differences are problematic to evaluate in the absence of data on ontogenetic and intraspecific variation, and on potential overlap with interspecific differences. For example, slight differences in the reproductive system could be due to different ontogenetic stages, therefore presently they cannot be used to discriminate species. Comparatively investigated serial semi-thin sections of *Pontohedyle kepii* sp. nov. also confirmed the similarity in all major organ systems reported previously [55,56]. We thus conclude that in *Pontohedyle* even advanced microanatomy is inefficient or even inadequate for species diagnoses. Molecular character sets currently offer the only chances for unambiguous discrimination between the different evolutionary lineages. Proponents of morphology based alpha taxonomy [84] might argue that we have not attempted a fully integrative approach since we have not performed 3D-microanatomy on all proposed new species, including enough material for intra-specific comparisons, ultrastructural data on, e.g., cilia, sperm morphology or specific gland types, to reveal whether these forms indeed represent cryptic species. However, in light of the biodiversity crisis and the corresponding challenges to taxonomy, we consider it as little effective to dedicate several years of a taxonomist’s life to the search for morphological characters, when there is little to expect, while molecular characters enable straightforward species delineation. This is not a plea to speed up description processes at the expense of accuracy and quality, or by allowing ignorance of morphology, but for a change in taxonomic practice to give molecular characters similar weight as morphological ones, in cases in which this is more informative or practical.

**Table 15:** **Putative distinguishing features between *P. milaschewitchii *** **and *P. verrucosa *** **(intraspecific variation not evaluated)**

	***P. milaschewitchii *** **(Kowalevsky, 1901)**	***P. verrucosa *** **(Challis, 1970)**
Data source	Jörger et al. 2008 [55]	Present study
Epidermal glands	Predominantly whitish, blue stained only in one small row	Predominantly whitish and numerous dark blue stained ones
Nervous system	Eyes pigmented and externally visible	Eyes unpigmented
Reproductive system	Only one cephalic male genital opening detected	Two male genital openings (cephalic and visceral)
Digestive system/ putatively different feeding habits	Lateral radula teeth with central denticle	Lateral radula teeth without denticle
	Lipid-like droplets in digestive gland	Refracting fusiform structures

Still debated is the way how the traditional Linnaean System needs to be adapted to incorporate different character sets, in the first place the growing amount of molecular data. Probably the most radical way ignores the character-based requirements of the International Code of Zoological Nomenclature [78,79] and proposes to base descriptions of new species directly on support values under species delineation models [85,86]. Aside from the paradigm shift this would bring, far away from long-standing taxonomic practice, opponents criticize that unambiguous allocation of newly collected material is impossible in the absence of definitions and descriptors and requires repetition of the species delineation approach applied [50]. As a method of species delineation, coalescent based approaches are objective and grounded on evolutionary history and population genetics [86,87]; thus it is indeed tempting to use results derived from molecular species delineations approaches directly as species descriptions ('model-based species descriptions’ [87]). This would clearly facilitate descriptions, thus reduce the taxonomic impediment and the risk of an endless number of discovered but undescribed candidate species. Every species description should aim for differentiation from previously described species; therefore, diagnostic characters are usually derived from comparisons to other, closely related species. Nevertheless, the species description itself has to be self-explanatory and should not rely on comparative measurements which are only valid in comparison to a special set of other species used for a certain analysis, i.e. on a complex construct that may not be reproducible when new data are added. In contrast to Fujita & Leaché [87], we believe that each species, i.e. separately evolving lineage [4], will present – in the current snap-shot of evolutionary processes – fixed diagnostic characters of some sort (e.g., from morphology, DNA sequence information, behavioral, karyology…), and we consider it the task of modern taxonomy to detect the most reliable and efficient set of characters on which to found species descriptions.

The Characteristic Attribute Organization System (CAOS) [51,57,58] is a character based method proposed for uniting species discovery and description [88]. As an approach to species delineation, we consider it inferior to coalescent based approaches (e.g., GMYC and BP&P); CAOS successfully determines putative diagnostic nucleotides, but is not predictive, i.e. lacks objective criteria with which to delimit a threshold number of distinguishing nucleotides that would indicate a species boundary. One has to distinguish between diagnosability of entities and the delimitation of species. Diagnostic characters of whatever sort can be found for all levels in the hierarchical classification, but there is no objective criterion for determining a number of characters needed to characterize a (new) species, e.g. versus a population. Nevertheless, for the purpose of species description, we think that character based approaches like CAOS are highly valuable and should complement molecular species delineation procedures, thus enabling the transition from species discovery to description.

### Requirements of molecular taxonomy

While calls for replacing the Linnaean system by a DNA sequence based one [41] have trailed away, we still lack a common procedure on how to include molecular data into the Linnaean system [21]*.* Like any other source of data, molecular data is not explicitly treated by the International Code of Zoological Nomenclature, there are no provisions dictating the choice of characters [78,79]. Currently, molecular data are included in species descriptions in various mutually inconsistent ways [21]. If DNA sequence data are only used as additive to, e.g., morphology based species descriptions or molecular species delineation approaches to confirm pre-identified entities, the addition is straightforward and requires no specific considerations. But if molecular sequence information is to be used as the partial or even sole content of a species description, a discussion of the corresponding best practice is needed.

#### ***Type material for species based on molecular data***

Previous authors highlighted the need for voucher material in molecular studies [89]. Ideally, DNA is extracted from (a subsample of) a name-bearing type specimen (holotype, syntype, lectotype or neotype); if no such specimen is available for molecular studies, an attempt should be made to collect fresh material at the type locality. If parts of larger animals belonging to putative new species are used for DNA extraction, DNA and remaining specimen can both become part of the type material under nomenclatural rules. However, where the members of a putatively new species, e.g. of meiofauna, are so small that molecular extraction from only part of an individual is impossible, taxonomists may be confronted with the critical decision to either have DNA without a morphological type specimen or a type without DNA. In taxonomically unproblematic groups one can add new material or use paratypes for DNA (or other) analyses, relying on specimens to be conspecific if they were collected from 'the same population’, i.e. from a place (and time) close enough to the type locality to assume gene flow. But what if, as has been shown for *Pontohedyle* slugs [25], there is a possibility of cryptic species occurring sympatrically and at the same time? Would it be better (A) to sacrifice a (single available) type specimen to obtain molecular data for species delineation or (B) to save the type and use a secondary specimen, taking the risk that the latter might not be conspecific with the former? In a group like our *Pontohedyle* slugs in which DNA sequence data are much more promising for species delineation than morphological approaches, and considering the wealth of potential DNA sequence characters, we prefer to sacrifice even single specimens to DNA extraction. In absence of a term referring to vouchers exclusively consisting of extracted DNA, we term this type material: 'DNA types’. However, prior to this, researchers should attempt an optimization of microscopical documentation (for details see [90]) and recovery of hard parts (e.g. radulae) from the spin columns used for extraction [91]. In the case of DNA aliquots serving as type material, natural history collections are urged to create long term DNA storage facilities [41,42] like the DNA bank network (http://www.dnabank-network.org/), and should apply the same caution and requirements (i.e. documentation of collection details) as for any morphological type.

#### ***Risk of two parallel taxonomies?***

Old type material often does not allow molecular analyses [84,92], and searching for fresh material at a type locality can be unsuccessful. Future technical advances are likely to enable DNA acquisition from some old type material, as there has been considerable progress in dealing with degenerated DNA [93]. Nevertheless, there are the potential risks that two parallel taxonomic systems could develop, and that the one based on molecular characters could duplicate, under separate names, some taxa already established on morphological grounds [77]. Similar concerns have arisen previously when the taxonomy of certain taxa was based on a character set other than morphology (e.g. cytotaxonomy based on data from chromosomes) and the investigation of one character set hindered the exploration of the other. It clearly remains the duty of taxonomists to carefully check type material of closely related taxa before describing new species [77]. To keep molecule driven taxonomy 'workable’ [94] and connected to traditional morphology based taxonomy, authors should include a brief morphological diagnosis of the (cryptic) species [77], even in the absence of species-diagnostic characters, in order to make the species recognizable as belonging to a certain group of (cryptic) species.

#### ***Trouble with names***

Any specimen identified from molecular data only can belong to a previously established species or to one new to science. If unambiguous identification with a single existing species name is possible then, of course, the latter should be used. In our cases in *Pontohedyle*, we call those Indo-Pacific specimens collected near the type locality of *P. verrucosa* (Challis, 1970) on the Solomon Islands by this single available name for Indo-Pacific *Pontohedyle.* Concerning Atlantic *Pontohedyle*, the name *P. brasilensis* (Rankin, 1979), proposed for Brazilian specimens, was treated as a junior synonym of the older name, *P. milaschewitschii* (Kowalevsky, 1901). Since we have shown that *P. milaschewitschii* refers to Mediterranean and Black Sea specimens only [25], we resurrected the name *P. brasilensis* for Western Atlantic *Pontohedyle*, and now apply it to the only species in of two cryptic ones that has been collected from Brazil. In doing so we accept the risk resulting from the fact that these specimens were collected at some distance from the type locality of *P. brasilensis* (see Figure 4), as the latter has not yielded any *Pontohedyle* specimens for more than the last 50 years, despite considerable and repeated collecting efforts, including our own. These assignments of previously established species names left at least nine additional, clearly separate *Pontohedyle* species for which available names did not exist. In cases of microscopic animals such as *Pontohedyle*, molecular taxonomy thus may benefit from morphology based taxonomy having missed them in the past.

#### ***Species descriptions based on singletons***

Species descriptions based on singleton specimens cannot reflect intraspecific variation, and Dayrat [1] even proposed a guideline to restrict species descriptions to well-sampled taxa. However, there is no objective way to determine any sample size at which intraspecific variation would be covered sufficiently. Moreover, excluding taxa described from singletons would lead to considerably lower, and effectively false, estimates of the scientifically known biodiversity [5,26-28]. The present study on *Pontohedyle* includes five species descriptions based on DNA sequence information from one individual only. Usually, this is done when such a singleton presents a combination of characters so discrete that it is considered highly unlikely to fall within the variational range of another species [28]. In a complex molecular species delineation approach Jörger et al. [25] recognized our five singletons as independently evolving lineages. Approximations with molecular clock analyses estimate the diversification of these species from their respective sister groups to have occurred 54–83 mya (own unpublished data), which indicates significant timespans of genetic isolation. In light of our general revision of the genus *Pontohedyle*, we consider it as less productive to keep these entities on the formally unrecognized level of candidate species than to run the risk that our species hypotheses may have to be modified due to future additional material. Nevertheless, we are well aware of the fact that taxon sampling and data acquisition (i.e. incomplete molecular data sets) are not yet ideal for some of our newly described species (e.g., *P.martynovi* sp. nov., *P. yurihookeri* sp. nov.).

#### ***What is a diagnostic character in molecular taxonomy?***

In character based taxonomy, descriptions of new taxa are, or should be, based on diagnostic differences from previously known taxa. In a phenetic framework (key systematics), similarity based distinction relies on sufficient sampling and detectable degrees of difference, whereas phylogenetic taxonomy additionally presumes knowledge of character homologies and sister group relationships. In an ideal phylogenetic framework diagnoses are based on apomorphic (i.e. derived) versus homologous but plesiomorphic (ancestral) states of a given character. In molecular taxonomy, the detection of homologies and apomorphic conditions among the four character states (bases) is handicapped by the high chance of convergent multiple transformations causing homoplasy. Reconstruction of ancestral sequences to support homology and differentiate between apomorphic and plesiomorphic character states for each node is possible [95]. However, unfortunately, robust phylogenetic hypotheses with strong support values for all sister group relationships are the exception rather than the rule. Since the evaluation of a state as apomorphic highly depends on the topology, and reconstruction of ancestral nucleotides is constrained by sampling coverage, we suggest more conservative approaches for cases of unclear phylogenetic relationships, as in our study. We use diagnostic nucleotides as unique character attributes (which may be apomorphic or plesiomorphic or convergent) within a certain entity, i.e. a monophylum with strong support values. This is clearly a trade off between the number and phylogenetic significance of diagnostic characters and the degree of dependence of these characters on a certain topology, as with increasing size and diversity of the selected entity, the likelihood of homoplasy also rises [96]. To enhance the stability of our molecular taxonomic characters we chose to determine diagnostic characters of each *Pontohedyle* species in relation to all its congeners, rather than just to the respective sister taxon as is the default in CAOS. Equal character states in non-*Pontohedyle* outgroups are left unconsidered, however, due to the larger evolutionary distances and the correspondingly increased risk of homoplasies. It will be one of the major challenges for molecule driven taxonomy to select the appropriate monophylum in which all included taxa are evaluated against each other. Rach et al. [88] addressed homoplasy within the selected ingroup by applying an 80% rule to so-called single private characters (see below). *Pontohedyle* species recognized here offered enough single pure diagnostic bases to avoid using single private characters and some further, more equivocal attributes provided by CAOS.

The Characteristic Attribute Organization System (CAOS) [51,57,58] can be used to identify diagnostic nucleotides for pre-defined taxonomic units [51]. The program offers discrimination between four types of 'character attributes’ (CAs): simple (single nucleotide position) vs. compound (set of character states) and pure vs. private [51]. Pure CAs are nucleotides present in all members of a clade and absent from members of other clades; private CAs are only present in some members of the clade, but absent from others [51]. We consider only single pure CAs as eligible for diagnostic characters in DNA taxonomy, i.e. as supporting new species proposals. In our diagnoses of the new *Pontohedyle* species we emphasize those single pure CAs, which in protein coding genes code for a different amino acid. The probability of single pure CAs referring to fixed genetic differences increases exponentially with their number [88]. In our dataset, all *Pontohedyle* species have between 12 and 36 single pure CAs on independently evolving markers, which supports their treatment as genetically isolated lineages. Additionally, the CAOS program distinguishes between homogeneous pure CAs (shared by all members of the taxon under study, and not present in the outgroups) and heterogeneous pure CAs (with two or three different characters present in the taxon but absent from the outgroups). The latter characters can be treated as diagnostic, but are problematic as they may refer to convergently evolved character states. Therefore, we report them as additional information. In contrast, compound CAs can be unique for certain species, but they may have evolved from several independent mutation events. Consequently, compound CAs as an entity have low probabilities of homology; in analogy to morpho-anatomical key systematics, these compound CAs can serve for re-identification of well-sampled species, but they are not diagnostic characters in a phylogenetic sense and thus should be avoided in DNA taxonomy.

CAOS identifies discrete nucleotide substitutions at every node of a given tree and has been complemented to find diagnostic bases in a 'phylogenetic-free context’ [97], referring to the difference between CAs and true apomorphies. This notion can be misleading, however, as the results provided by CAOS are one hundred percent topology dependent in only comparing sister pairs at each node. To overcome this topology dependence, we ran several analyses placing each species at the root of the ingroup, which we defined as the most inclusive secure and taxonomically relevant monophylum, in our case the genus *Pontohedyle* (see Material and Methods). This procedure of a manually iterative, exhaustive intrageneric comparison of base conditions makes the recognized single pure CAs less numerous but more rigorous than with CAOS default parameters, i.e. by decreasing the chances of homoplasy and increasing the chances of single pure CAs representing apomorphies in our wider taxon comparison.

#### ***Towards a 'best practice’ in molecular taxonomy***

Considering stability and traceability in future research, the presentation of the identified diagnostic nucleotides is not trivial. Some recent studies just reported the number of differing nucleotides without specifying the position and character state e.g., [98]. This is equivalent to a morphological species description that would merely refer to, e.g., 'diagnostic differences in the reproductive system’ without offering any descriptive details. Other studies present part of an alignment without identifying positions, and underline putative diagnostic nucleotides e.g., [99] without explanation what determined these bases as diagnostic. This practice leaves it to future researchers to identify the proposed bases, which is highly time consuming and error-prone, especially when the original alignment is not deposited in a public database. Reporting the positions within the alignment is a step towards reproducibility and traceability of molecular diagnostic characters e.g., [94,100-102], but when new material is added that was generated with different primers or includes insertions or deletions, the critical positions are still difficult to trace. Yassin et al. [103] included the positions within a reference genome, which probably provides the greatest clarity for future research. Unfortunately, for non-model taxa closely related reference genomes which allow for unambiguous alignment of even fast evolving markers are usually unavailable. We thus suggest the following procedure for reporting positions in an alignment. (1) Clearly report primers and alignment programs, and clarify what determined position 1 (e.g., first base after the primer sequence); (2) deposit alignments in public databases or as additional material accompanying the publication's online edition. To make a diagnostic position in a sequence traceable independently from a specific alignment, we additionally recommend to (3) report the corresponding position in a deposited reference sequence (ideally generated from type material). Technically, the necessary values are easily retrievable from sequence editing programs such as Geneious [104]. To evaluate intraspecific variation, sequences from all specimens assigned to a certain species were included in our analyses of diagnostic characters. In new species descriptions the provided reference sequences should be generated from type material. In cases where the molecular data retrieved from the type are, however, incomplete, we consider it little problematic to additionally include data from other specimens, if there is justification on conspecifity (e.g. via other molecular markers). If future research rejects conspecifity, the respective characters can be easily excluded from the original description. We refrain from adopting the term 'genetype’, however, as label for sequences data from type material [105], as it might be easily misunderstood: sequences themselves are not types but amplified copies of certain parts of type material.

Since an alignment presents the positional homology assumptions that are crucial for the determination of diagnostic nucleotides, we consider the quality of the alignment as essential for the success of molecular taxonomy. Therefore, we sincerely recommend to critically compare the output of different alignment programs, as in the present study. While coding mitochondrial markers (such as COI) can be checked via reading frames and translation into amino acids, and are generally less problematic, non-coding fast evolving markers (e.g. 16S rRNA) can be difficult to align even among closely related species. Obviously, undetected misalignments can result in tremendous overestimation of diagnostic characters. For example, a misalignment occurred in the ClustalW approach to our 28S rRNA dataset, which increased the number of characters diagnostic for a sister clade within *Pontohedyle wenzli* sp. nov. on this marker from 0 to 34 compared to the MUSCLE [106] alignment. And even without obvious misalignments, the use of different alignment programs can result in a differing number of diagnostic nucleotides (e.g. 9 vs. 13 diagnostic nucleotides in *P. milaschewitchii* comparing the MUSCLE and ClustalW alignment). By removing ambiguous parts of the alignment, one reduces the number of diagnostic characters considerably (e.g. from 19 to 13 diagnostic nucleotides on 16S rRNA in *P. milaschewitchii* when masking ClustalW alignments with Gblocks [107]). However, those diagnostic characters that remain can be considered as more stable and reliable for species identification. Based on our comparative analyses, we decided to choose the most conservative approach (alignment conducted with MUSCLE [106] and masked with GBlocks [107]), and based on the above mentioned examples stress the need to dedicate time to alignment issues when performing molecular taxonomy.

Several potential sources of error unique to taxonomy from molecular data have been pointed out [23]. (1) contamination and chimeric sequences, (2) faulty alignments resulting in comparisons of non-homologous nucleotides, and (3) the risk of dealing with paralogs. Authors of species descriptions based on molecular data should bear these pitfalls in mind. The risk of chimeric sequences can be reduced by carefully conducting BLAST searches [108] for each amplified fragment; misidentifications of diagnostic characters due to non-homologous alignments can be avoided by applying the considerations discussed above. The quality and stability of molecular taxonomic results considerably increase when several independent loci support the species delineation. To avoid idiosyncrasies of individual markers, misidentifications due to sequencing errors, or the pitfalls of paralogs, we strongly recommend not to base molecular species delineation and subsequent species description on single markers. Otherwise, if subsequent results negate the diagnostic value of nucleotides on that marker, the species description loses its entire foundation. Furthermore, the use of single pure CAs rather than of other types of CAs, and especially the use of genus-level compared CAs as discussed above, increases the chances of establishing and diagnosing new species on apomorphies rather than on homoplasies.

We acknowledge the risk that species descriptions based on molecular data might contain errors in the form of incorrectly assumed apomorphies, especially when working in sparsely sampled groups. Moreover, putative molecular apomorphies of described species may have to be reconsidered as plesiomorphies when new species with the same characteristics are added, or they may vanish in intraspecific variation. The more potentially apomorphic nucleotides are found across independently evolving markers, the higher the chances that at least some of them truly refer to unique mutations accumulated due to the absence of gene exchange. But in all this, molecular characters do not differ from morphological or other sets of characters. Species descriptions are complex hypotheses on several levels: novelty of taxon, placement within systematic context, and hypothesis of homology applying descriptive terms [5,109,110]. Species descriptions based on molecular characters are founded on the well-established hypothesis that character differences reflect lineage independence [50] and that mutations accumulate in the absence of gene exchange. It is the task of the taxonomist to evaluate whether the observed differences in character states can be explained by a historical process causing lineage divergence [3]. According to rough time estimations by molecular clock analyses, the radiation of *Pontohedyle* species included in the present study took place 100–25 mya (own unpublished data). Therefore we are confident that many of the bases recognized as diagnostic within our sampling truly refer to evolutionary novelties and unique attributes of species-level entities. However, even in cases of more recent divergences it should be possible to detect at least some diagnostic bases. Regardless of which character set a species description is based on, species descriptions are hypotheses, which means that they need to be re-evaluated, i.e. confirmed, falsified or modified when new data, material or methods of analysis become available.

## Conclusions

This contribution issues a plea to follow up discoveries of cryptic species by molecular species delineation with the steps necessary to establish formal scientific names for these species. This can be achieved by selection of diagnostic characters, e.g., via the CAOS software. Depending on the robustness of the underlying phylogenetic hypothesis, taxonomists need to evaluate the optimal balance between the number of diagnostic bases and their stability subject to the topology. In general, pure diagnostic bases rather than private or combined ones should be selected, and such single pure CAs should be compared against all the potentially closely related lineages, not only against the direct sister in a predefined tree entered in CAOS as is the default procedure. We also wish to highlight the following considerations. 1) When basing a species description on molecular data the same rules as in traditional taxonomy should be applied considering deposition and accessibility of data; DNA aliquots and additional type material should be deposited in long term storage facilities, and sequences in public databases (GenBank). As with morphological type specimens, special attention should be given to the storage and availability of molecular types. 2) Due to the underlying homology assumption, we consider the quality of the alignment as critical to determining and extracting diagnostic bases. Thus, we recommend exploring changes to the alignment and, thus, the identified diagnostic characters by applying different alignment programs and masking options. 3) Alignments may change when new data is added, especially concerning non-coding markers. For better traceability, we regard it as beneficial to report not only the alignment position but also refer to a closely related reference genome (if applicable) and report the position in a deposited reference sequence (ideally generated from type material). In its current stage of development, the extraction of diagnostic characters for molecular taxonomy is not yet ready for inclusion in automated species delimitation procedures, as it still requires time-consuming manual steps. However, little adaptation of existing programs would be needed to make them serve molecular taxonomy in its entirety, to overcome the current gap between species discovery and species description.

## Methods

### Type localities and collecting sites

The collecting sites of material included in the present study are shown in Figure 4 (modified after Jörger et al. [25]). Of the three valid species, we were able to recollect *P. milaschewitchii* from its type locality. *P. verrucosa* was collected in vicinity of the type locality on Guadalcanal, Solomon Islands. Despite several attempts, we were unsuccessful in recollecting *P. brasilensis* at the type locality (see Discussion for assignment of specimens to this species).

### Morphology and microanatomy

Jörger et al. [25] analyzed the radulae of most of the species described above. Unfortunately, for *Pontohedyle neridae* sp. nov., *P. martynovi* sp. nov. and *P. yurihookeri* sp. nov. radulae could not be recovered from the specimens used for DNA extraction. The radula of *P. wiggi* sp. nov. could only be studied under the light microscope, but was lost when attempting to transfer it to a SEM-stub.

Phylogenetic analyses by Jörger et al. [25] revealed two major clades within *Pontohedyle*. One includes *P. milaschewitchii*, for which detailed microanatomical and ultrastructural data is available [55,111]. The other clade is morphologically poorly characterized, since the original description of *P. verrucosa* lacks details on major organ systems like the reproductive system and the nervous system. For detailed histological comparison of the two major *Pontohedyle* clades, glutaraldehyde fixed specimens of *P. verrucosa* (from near the type locality WP-3 and WP-2 see [25]) were post-fixed in buffered 1% osmium tetroxide, decalcified using ascorbic acid and embedded in Spurr low-viscosity epoxy resin [112] or Epon epoxy resin (for detailed protocols see [113,114]). Serial semi-thin sections (1 and 1.5 μm) of three specimens were prepared using a diamond knife (Histo Jumbo, Diatome, Switzerland) with contact cement on the lower cutting edge to form ribbons [115]. Ribbons were stained using methylene-blue azur II [116] and sealed with Araldit resin under cover slips. Sectioned series are deposited at the Bavarian State Collection of Zoology, Mollusca section (ZSM Mol-20071833, 20071837 and 20100548). Additionally, histological series of *Pontohedyle kepii* sp. nov. were sectioned as described above.

Digital photographs of each section were taken using a ProgRes C3 camera (Jenoptik, Germany) mounted on a Leica DMB-RBE microscope (Leica Microsystems, Germany). Subsequently, photographs were edited (i.e., grey-scale converted, contrast enhanced and reduced in size) using standard imaging software, then loaded into AMIRA 5.2 (Visage Imaging Software, Germany) for 3D reconstruction of the major organ systems. Alignment, labeling of the organ systems and surface rendering followed in principle the method described by Ruthensteiner [115].

### Acquisition of molecular data

This study aims to characterize the genus *Pontohedyle* (Acochlidia, Microhedylacea) based on molecular standard markers, i.e., nuclear 18S and 28S rRNA and mitochondrial COI and 16S rRNA. We included the three previously valid *Pontohedyle* species (for taxonomy see [69,83]): *P. milaschewitchii* (Kowalewsky, 1901), *P. verrucosa* (Challis, 1970) and recently re-established *P. brasilensis* (Rankin, 1979) [25]. The nine additional species earlier identified as candidates in the genus *Pontohedyle*[25] are subject to molecular taxonomy. 28S rRNA, 16S rRNA and COI sequences analyzed by Jörger et al. [25] were retrieved from GenBank (see Table 1 for accession numbers). Additionally, we amplified nuclear 18S rRNA (approx. 1800 bp) for at least one individual per species. 18S rRNA was amplified in three parts using the primers for euthyneuran gastropods by Vonnemann et al. [65] and Wollscheid & Wägele [117]: 18A1 (5’ - CCT ACT TCT GGT TGA TCC TGC CAG T – 3′), 700R (5′ - CGC GGC TGC TGG CAC CAG AC – 3′), 470 F (5′ - CAG CAG GCA CGC AAA TTA CCC – 3′), 1500R (5′ - CAT CTA GGG CAT CAC AGA CC – 3′), 1155 F (5′ - CTG AAA CTT AAA GGA ATT GAC GG – 3′), 1800 (5′ - TAA TGA TCC TTC CGC AGG TT – 3′). Polymerase chain reactions were conducted using Phire polymerase (New England Biolabs) following this protocol: 98°C 30 sec, 30-35x (98°C 5 sec, 55-65°C 5 sec, 72°C 20-25 sec), 72°C 60 sec. Successful PCR products were cleaned up with ExoSap IT. Cycle sequencing such as sequencing reactions was performed by the Genomic Service Unit (GSU) of the Department of Biology, Ludwig-Maximilians-University Munich, using Big Dye 3.1 kit and an ABI 3730 capillary sequencer. Sequences were edited (forward and reverse strands), concatenated and checked for potential contamination via BLAST searches [108] against the GenBank database via Geneious 5.5.2 [104].

#### ***Detection of diagnostic molecular characters***

We used the Characteristic Attribute Organization System (CAOS) [51,57,58] to detect discrete nucleotide substitutions on our previously determined candidate species [25]. The program distinguishes single (single nucleotide) vs. compound (set of nucleotides) 'character attributes’ (CA) [51]. Both, single and compound CAs can be further divided into pure (present in all members of a clade but absent from all members of another clade) and private CAs (only present in some members of the clade, but absent in members of other clades) [51]. For taxonomic purposes at this stage we consider only 'single pure characters’ (sPu) as diagnostic characters for species descriptions (see Discussion). Since some sister group relationships among *Pontohedyle* species are not well supported (see [25], Figure 1), we chose our diagnostic molecular characters in the sense of unique within the genus *Pontohedyle*, rather than assigning plesiomorphic or apomorphic polarity to character states of one species in relation to its direct sister species.

As discussed above, the homology assumption presented in the alignment is crucial for the correct detection of diagnostic characters. For quality control, we performed data input into CAOS with alignments derived from three commonly applied alignment programs and critically compared the resulting differences concerning amounts and positions of the sPus. Alignments were generated for each marker individually using MUSCLE [106], Mafft [118,119] and CLUSTAL W [120]. The COI alignment was checked manually, supported by translation into amino acids. Due to difficulties in aligning highly variable parts of rRNA markers, we removed ambiguous parts of the alignment with two different masking programs, Aliscore [121] and GBlocks [107], and compared the respective effects on character selection. After comparison of the various results we chose MUSCLE [106] in combination with GBlocks [107] as the most conservative approach that results in fewer but more reliable diagnostic characters than the other approaches.

Alignments were analyzed and converted between different formats using Geneious 5.6 (Biomatters) [104]. We performed a phylogenetic analysis under a maximum-likelihood approach with RAxML 7.2.8 on each individual marker, applying the 'easy and fast way’ described in the RAxML 7.0.4 manual to obtain an input tree. For our present study the phylogenetic hypothesis on sister group relationships of the different *Pontohedyle* species, however, is not relevant: We manipulated the resulting trees in Mesquite [122], generating a single starting file for CAOS for each species and for each marker, with each of the analyzed species successively being sister to all remaining *Pontohedyle* species. This iterative procedure retrieves diagnostic characters for the node that compares each single species to all its congeners.

The single gene alignments which formed the basis for the selection of diagnostic nucleotides are available in fasta format as Additional material 3–6. Diagnostic nucleotides are reported with positions in the reference alignment. Position 1 of each alignment refers to position 1 after the primer region, which was removed in the alignment. For better traceability, and in the absence of a closely related reference genome, we additionally report the positions within a reference sequence for each species (deposited in GenBank; see Table 1). In the description of our new species these reference sequences are retrieved from the holotype. Diagnostic molecular characters of the genus *Pontohedyle* in 18S and 28S rRNA are diagnosed based on alignments including all available *Pontohedyle* sequences (Table 1) and representatives of all other acochlidian genera currently available in public databases (see Additional files 1 and 2 for the original alignments in fasta format).

To meet the requirements by the International Code of Zoological Nomenclature (ICZN) [78,79], this article was registered at ZooBank (http://www.zoobank.org) under the ZooBank Life Science Identifiers (LSIDs): urn:lsid:zoobank.org:pub:4AE75E9C-4303-42CB-AED2-77266C8F6601.

## Competing interests

Both authors declare that they have no competing interests.

## Authors' contributions

KMJ generated the morphological and molecular data and drafted the manuscript. MS planned and supervised the study. The material was collected jointly and in cooperation with a series of collaborators (see Acknowledgements). All authors read and approved the final manuscript.

## Supplementary Material

Additional file 1 **18S rRNA alignment of *Pontohedyle *with outgroups to determine diagnostic nucleotides for the genus (fasta format).** The alignment was generated with MUSCLE [107] and ambiguous parts of the alignment were masked with Gblocks [108] (settings for a less stringent selection). Click here for file

Additional file 2 **28S rRNA alignment of *Pontohedyle *with outgroups to determine diagnostic nucleotides for the genus (fasta format).** The alignment was generated with MUSCLE [107] and ambiguous parts of the alignment were masked with Gblocks [108] (settings for a less stringent selection). Click here for file

Additional file 3 **18S rRNA alignment of *Pontohedyle *(fasta format).** The alignment was generated with MUSCLE [107] and ambiguous parts of the alignment were masked with Gblocks [108] (settings for a less stringent selection). Click here for file

Additional file 4 **28S rRNA alignment of *Pontohedyle *(fasta format).** The alignment was generated with MUSCLE [107] and ambiguous parts of the alignment were masked with Gblocks [108] (settings for a less stringent selection). Click here for file

Additional file 5 **16S rRNA alignment of *Pontohedyle *(fasta format).** The alignment was generated with MUSCLE [107] and ambiguous parts of the alignment were masked with Gblocks [108] (settings for a less stringent selection). Click here for file

Additional file 6 **COI alignment of *Pontohedyle *(fasta format).** The alignment was generated with MUSCLE [107]. Click here for file
