# Supplementary material for: How to describe a cryptic species? Practical challenges of molecular taxonomy
Source: Front Zool. 2013 Sep 27;10:59. doi: 10.1186/1742-9994-10-59 (PMC4015967; doi:10.1186/1742-9994-10-59)
Supplement: Additional file 1 — 18S rRNA alignment of Pontohedyle with outgroups to determine diagnostic nucleotides for the genus (fasta format). The alignment was generated with MUSCLE [107] and ambiguous parts of the alignment were masked with Gblocks [108] (settings for a less stringent selection). [file 1742-9994-10-59-S1.docx]

### Additional file 1 – 18S rRNA alignment of *Pontohedyle* with outgroups to determine diagnostic nucleotides for the genus (fasta format)

The alignment was generated with Muscle [[96](#_ENREF_96)] and ambiguous parts of the alignment were masked with Gblocks [[97](#_ENREF_97)] (settings for a less stringent selection).

>ZSM20080565

NNNNNNNAAAGATTAAGCCATGCATGTCTAAGTTCACACTATCTCACGGTGAAACCGCGAATGGCTCATTAAATCAGTCGAGGTTCCTTAGATGACACGATCCTACTTGGATAACTGTGGCAATTCTAGAGCTAATACATGCCTCTGAAGCTCCGACCTCTAGGGAAGAGCGCTTTTATTAGTTCAAAACCAATCGCACGTGTCCGAGGCGGCGCGTCCCATTTGGTGACTCTGGATAACTTTGTGCTGATCGCATGGCCTCCTGCGCCGGCGACGCATCTTTCAAATGTCTGCCCTATCAAATGTCGATGGTACGTGACATGCCTACCATGTTTGTAACGGGTAACGGGGAATCAGGGTTCGATTCCGGAGAGGGAGCATGAGAAACGGCTACCACATCCAAGGAAGGCAGCAGGCGCGCAACTTACCCACTCCCGGCACGGGGAGGTAGTGACGAAAAATAACAATACGGGACTCTTTCGAGGCCCCGTAATTGGAATGAGTACACTTTAAACCCTTTAACGAGGATCTATTGGAGGGCAAGTCTGGTGCCAGCAGCCGCGGTAATTCCAGCTCCAATAGCGTATATTAAAGTTGTTGCAGTTAAAAAGCTCGTAGTTGGATCTCAGGCGCAGGCGGGTGGTCCGGCTCGCGCCGGCTCACTGCCCGTACTCCTGCCCTACCTGTTGTCGGCTCTCTCCCGCGGGTGCTCTTCACTGAGCGTCCCGGGTGGCCGGCGCGTTTACTTTGAAAAAATTAGAGTGTTCAAAGCAGGCCTTGGCTGCCTGAATAATGGTGCATGGAATAATGGAATAGGATCTCGGTTCTATTTTGTTGGTTTTCGGAACTAGAGGTAATGATTAACAGGGACAAACGGGGGCATTCGTATTGCGGCGTTAGAGGTGAAATTCTTGGATCGCCGCAAGACGAGCTACTGCGAAAGCATTTGTCAAGAATGTTTTCATTAATCAA???????????????????????????????????????????????????????????????????????????????????????????????????????????????????????????????????????????????????????????????????????????????????????????????????????AGCCTGCGGCTTAATTTGACTCAACACGGGAAAACTCACCCGGTCCGGACACTGTAAGGATTGACAGATTGATAGCTCTTTCTTGATTCGGTGGGTGGTGGTGCATGGCCGTTCTTAGTTGGTGGAGCGATTTGTCTGGTTAATTCCGATAACGAACGAGACTCTAGCCTATTAAATAGTTCGCCGATTCTTTGATGCGTCGGCGCAACTTCTTAGAGGGACGAGTGGCGTTTAGCCACACGAGATTGAGCAATAACAGGTCTGTGATGCCCTTAGATGTCCGGGGCCGCACGCGCGCTACACTGAAGGAATCAGCGTGGATGCCTCCCTGGTCCGAAAGGATTGGGAAACCCGTTGAATCTCCTTCGTGCTAGGGATTGGGGCTTGTAATTCTTCCCCATGAACGAGGAATTCCCAGTAAGCGCGAGTCATAAGCTCGCGTTGATTACGTCCCTGCCCTTTGTACACACCGCCCGTCGCTACTATCGATTGAGCGGTTCAGTGAGGGCCTCGGATTGGTCTCGGTCTGGCGTGCAAGCGCCGGCACCGTTGGCCGAGAAGACGCTCGAACTCGATCGCTTGGAGAAAGTAAAAGTCGTAACAAGGTTTCCGTAGGTG

>ZSM20100592

TTGTCTCAAAGATTAAGCCATGCATGTCTAAGTTCACACTATCTCACGGTGAAACCGCGAATGGCTCATTAAATCAGTCGAGGTTCCTTAGATGACACGATCCTACTTGGATAACTGTGGCAATTCTAGAGCTAATACATGTCTCTGAAGCTCCGACCTGCAGGGAAGAGCGCTTTTATTAGTTCAAAACCAATCGTGCGGTTCCCGGGCCGCACGCCCACTGTGGTGACTCAGAATAACTTTGTGCTGATCGCATGGCCTCCTGCGCCGGCGACGCATCTTTCAAATGTCTGCCCTATCAAATGTCGATGGTACGTGACATGCCTACCATGTTTGTAACGGGTAACGGGGAATCAGGGTTCGATTCCGGAGAGGGAGCATGAGAAACGGCTACCACATCCAAGGAAGGCAGCAGGCGCGCAACTTACCCACTCCCGGCACGGGGAGGTAGTGACGAAAAATAACAATACGGGACTCTTTCGAGGCCCCGTAATTGGAATGAGTACACTTTAAACCCTTTAACGAGGATCTATTGGAGGGCAAGTCTGGTGCCAGCAGCCGCGGTAATTCCAGCTCCAATAGCGTATATTAAAGTTGTTGCAGTTAAAAAGCTCGTAGTTGGATCTGAGGCGCAGGCGGGTGGTCCGGCTCGCGCCGGCTCACTGCCCGTACTCCTGCCCTACCTGTTGTCGGCTCTCTCCCGCGGGTGCTCTTCGCTGAGCGTCCCGGGTGGCCGGCGCGTTTACTTTGAAAAAATTAGAGTGTTCAAAGCAGG-CTATTCAGCCTGAATAATGGTGCATGGAATAATGGAATAGGACCTCGGTTCTATTTTGTTGGTTTTCGGAACTAGAGGTAATGATTAACAGGGACGAACGGGGGCATTCGTATTGCTGCGTTAGAGGTGAAATTCTTGGATCGCAGCAAGACGAGCTACTGCGAAAGCATTTGTCAAGAATGTTTTCATTAATCAAGAACGAAAGTCAGAGGCGCGAAGACGATCAGATACCGTCGTAGTTCTGACCATAATCGATGCCAGCTAGCAATCCGCAGGAGTTGCTTCGATGACTCTGCGGGCAGCTTCCGGGAAACCAAAGTTTTTGGGTTCCGGGGGAAGTATGGTTGCAAAGCTGAAACTTAAAGGAATTGACGGAAGGGCACCACCAGGAGTGGAGCCTGCGGCTTAATTTGACTCAACACGGGAAAACTCACCCGGTCCGGACACTGTAAGGATTGACAGATTGATAGCTCTTTCTTGATTCGGTGGGTGGTGGTGCATGGCCGTTCTTAGTTGGTGGAGCGATTTGTCTGGTTAATTCCGATAACGAACGAGACTCTAGCCTATTAAATAGTTCGCCGATTCCTTGATGCGTCGGCGCAACTTCTTAGAGGGACGCGTGGCGTTTAGCCACACGAGATTGAGCAATAACAGGTCTGTGATGCCCTTAGATGTCCGGGGCCGCACGCGCGCTACACTGAAGGAATCAGCGTGGATGCCTCCCTGGTCCGAAAGGATTGGGAAACCCGTTGAATCTCCTTCGTGCTAGGGATTGGGGCTTGTAATTCTTCCCCATGAACGAGGAATTCCCAGTAAGCGCGAGTCATAAGCTCGCGTTGATTACGTCCCTGCCCTTTGTACACACCGCCCGTCGCTACTATCGATTGAGCGGTTCAGTGAGGGCCTCGGATTGGTCTCGGTCTGGCGTGCAAGCGCCGGCACCGTTGGCCGAGAAGACGCTCGAACTCGATCGCTNNNNNNNNNNNNNNNNNNNNNNNNNNNNNNNNNNNNNN

>ZSM20071820

TTGTCTCAAAGATTAAGCCATGCATGTCTAAGTTCACACTATCTCACGGTGAAACCGCGAATGGCTCATTAAATCAGTCGAGGTTCCTTAGATGACACGATCCTACTTGGATAACTGTGGCAATTCTAGAGCTAATACATGCTTTTGAAGCTCCGACCTAAAGGGAAGAGCGCTTTTATTAGTTCAAAACCAATCGCTCGGCCTT-GTGTCGGGCGCCCACTTTGGTGACTCTGGATAACTTTGTGCTGATCGCATGGCC-CTGGCGCCGGCGACGCATCTTTCAAATGTCTGCCCTATCAAATGTCGATGGTACGTGACATGCCTACCATGTTTGTAACGGGTAACGGGGAATCAGGGTTCGATTCCGGAGAGGGAGCATGAGAAACGGCTACCACATCCAAGGAAGGCAGCAGGCGCGCAACTTACCCACTCCCGGCACGGGGAGGTAGTGACGAAAAATAACAATACGGGACTCTTTCGAGGCCCCGTAATTGGAATGAGTACACTTTAAACCCTTTAACGAGGATCTATTGGAGGGCAAGTCTGGTGCCAGCAGCCGCGGTAATTCCAGCTCCAATAGCGTATATTAAAGTTGTTGCAGTTAAAAAGCTCGTAGTTGGATCTCAGGCGCAGGCGGGTGGTCCGGCTCGCGCCGGCTCACTGCCCGTACTCCTGCCCTACCTGTTGTCGGCTCTCTCCCGCGGGTGCTCTTCACTGAGCGTCCCGGGTGGCCGGCGCGTTTACTTTGAAAAAATTAGAGTGTTCAAAGCAGG-CTAAATAGCCTGAATAATGGTGCATGGAATAATGGAATAGGACCTCGGTTCTATTTTGTTGGTTTTCGGAACTAGAGGTAATGATTAACAGGGACAAACGGGGGCATTCGTATTGCTGCGTTAGAGGTGAAATTCTTGGATCGCAGCAAGACGAGCTACTGCGAAAGCATTTGTCAAGAATGTTTTCATTAATCAAGAACGAAAGTCAGAGGCGCGAAGACGATCAGATACCGTCGTAGTTCTGACCATAAACGATGCCACCTAGCGATCCGCAGGAGTTGCTTCGATGACTCTGCGGGCAGCTTCCGGGAAACCAAAGGTTTTGGGTTCCGGGGGAAGTATGGTTGCAAAGCTGAAACTTAAAGGAATTGACGGAAGGGCACCACCAGGAGTGGAGCCTGCGGCTTAATTTGACTCAACACGGGAAAACTCACCCGGTCCGGACACTGTAAGGATTGACAGATTGATAGCTCTTTCTTGATTCGGTGGGTGGTGGTGCATGGCCGTTCTTAGTTGGTGGAGCGATTTGTCTGGTTAATTCCGATAACGAACGAGACTCTAGCCTATTAAATAGTTCGCCGATTCTTTGATGCGTCGGCGCAACTTCTTAGAGGGACGAGTGGCGTTTAGCCACACGAGATTGAGCAATAACAGGTCTGTGATGCCCTTAGATGTCCGGGGCCGCACGCGCGCTACACTGAAGGAATCAGCGTGGATGCCTCCCTGGTCCGAAAGGATTGGGAAACCCGTTGAATCTCCTTCGTGCTAGGGATTGGGGCTTGTAATTCTTCCCCATGAACGAGGAATTCCCAGTAAGCGCGAGTCATAAGCTCGCGTTGATTACGTCCCTGCCCTTTGTACACACCGCCCGTCGCTACTATCGATTGAGCGGTTCAGTGAGGGCCTCGGATTGGTCTCGGTCTGGCGTGCAAGCGCCGGCACCGTTGGCCGAGAAGACGCTCGAACTCGATCGCTTGGAGAAAGTAAAANNNNNNNNNNNNNNNNNNNNNNNN

>ZSM20081013

NNNNNNNNNNNNNNNNNCCATGCATGTCTAAGTTCACACTATCTCACGGTGAAACCGCGAATGGCTCATTAAATCAGTCGAGGTTCCTTAGATGACACGATCCTACTTGGATAACTGTGGCAATTCTAGAGCTAATACATGCTTTTGAAGCTCCGACCTAAAGGGAAGAGCGCTTTTATTAGTTCAAAACCAATCGG-----TCCCGGGC----CGCCCCCTTTGGTGACTCTGGATAACTTTGTGCTGATCGCATGGCC-TTTGCGCCGGCGACGCATCTTTCAAATGTCTGCCCTATCAAATGTCGATGGTACGTGACATGCCTACCATGTTTGTAACGGGTAACGGGGAATCAGGGTTCGATTCCGGAGAGGGAGCATGAGAAACGGCTACCACATCCAAGGAAGGCAGCAGGCGCGCAACTTACCCACTCCCGGCACGGGGAGGTAGTGACGAAAAATAACAATACGGGACTCTTTCGAGGCCCCGTAATTGGAATGAGTACACTTTAAACCCTTTAACGAGGATCTATTGGAGGGCAAGTCTGGTGCCAGCAGCCGCGGTAATTCCAGCTCCAATAGCGTATATTAAAGTTGTTGCAGTTAAAAAGCTCGTAGTTGGATCTCAGGCGCAGGCGGGTGGTCCGGCTCGCGCCGGCTCACTGCCCGTACTCCTGCCCTACCTGTTGTCGGCTCTCTCCCGCGGGTGCTCTTTACTGAGCGTCCCGGGTGGCCGGCGCGTTTACTTTGAAAAAATTAGAGTGTTCAAAGCAGG-CTCGATAGCCTGAATAATGGTGCATGGAATAATGGAATAGGACCTCGGTTCTATTTTGTTGGTTTTCGGAACTAGAGGTAATGATTAACAGGGACAAACGGGGGCATTCGTATTGCGGCGTTAGAGGTGAAATTCTTGGATCGCCGCAAGACGAGCTACTGCGAAAGCATTTGTCAAGAATGTTTTCATTAATCAAGAACGAAAGTCAGAGGCGCGAAGACGATCAGATACCGTCGTAGTTCTGACCATAAACGATGCCACCTAGCGATCCGCAGGAGTTGCTTCGATGACTCTGCGGGCAGCTTCCGGGAAACCAAAGGTTTTGGGTTCCGGGGGAAGTATGGTTGCAAAGCTGAAACTTAAAGGAATTGACGGAAGGGCACCACCAGGAGTGGAGCCTGCGGCTTAATTTGACTCAACACGGGAAAACTCACCCGGTCCGGACACTGTAAGGATTGACAGATTGATAGCTCTTTCTTGATTCGGTGGGTGGTGGTGCATGGCCGTTCTTAGTTGGTGGAGCGATTTGTCTGGTTAATTCCGATAACGAACGAGACTCTAGCCTATTAAATAGTTCGCCGATTCCTACATGCGTCGGCGCAACTTCTTAGAGGGACGAGTGGCGTTTAGCCACACGAGATTGAGCAATAACAGGTCTGTGATGCCCTTAGATGTCCGGGGCCGCACGCGCGCTACACTGAAGGAATCAGCGTGGATGCCTCCCTGGTCCGAAAGGATTGGGAAACCCGTTGAATCTCCTTCGTGCTAGGGATTGGGGCTTGTAATTCTTCCCCATGAACGAGGAATTCCCAGTAAGCGCGAGTCATAAGCTCGCGTTGATTACGTCCCTGCCCTTTGTACACACCGCCCGTCGCTACTATCGATTGAGCGGTTCAGTGAGGGCCTCGGATTGGTCTCGGTCTGGCGTGCAAGCGCCGGCACCGTTGGCCGAGAAGACGCTCGAACTCGATCGCTTGGAGAAAGTAAAAGTCGTAACAAGGTTTCNNNNNNNN

>ZSM20090471

TTGTCTCAAAGATTAAGCCATGCATGTCTAAGCTCACACCATCTCACGGTGAAGCCGCGAATGGCTCATTAAATCAGTCGAGGTTCCTTAGATGACACGATCCTACTTGGATAACTTTGGCAATTCTATAGCTAATACATGCTTGTCGAGCTCCGACCTAAAGGGAAGAGCGCTTTTATTAGTCCAAAACCAATCGCTCGGCCCC--GGTCGTGCGCCCCCACTGGTGACTCTGGATAACTTTGTGCTGATCGCATGGCC-CCGGCGCCGGCGACGCATCTTTCAAATGTCTGCCCTATCAAATGTCGATGGTACGTGACATGCCTACCATGTTTGTAACGGGTAACGGGGAATCAGGGTTCGATTCCGGAGAGGGAGCATGAGAAACGGCTACCACATCCAAGGAAGGCAGCAGGCGCGCAACTTACCCACTCCCGGCACGGGGAGGTAGTGACGAAAAATAACAATACGGGACTCTTTCGAGGCCCCGTAATTGGAATGAGTACACTTTAAACCCTTTAACGAGGATCTATTGGAGGGCAAGTCTGGTGCCAGCAGCCGCGGTAATTCCAGCTCCAATAGCGTATATTAAAGTTGTTGCAGTTAAAAAGCTCGTAGTTGGATCTCAGGTGCAGGCGGGTGGTCCGGCTCGCGCCGGCTCACTGCCCGTTCTCCTGCCCTACCCGTTGTCGGCTCTCTCCCGCGGGTGCTCTTCGCTGAGCGTCCCGGGTGGCCGGCGCGTTTACTTTGAAAAAATTAGAGTGTTCAAAGCAGGCCTCGGCTGCCTGAATAATGGTGCATGGAATAATGGAATAGGACCTCGGTTCTATTTTGTTGGTTTTCGGAACTGGAGGTAATGATTAACAGGGACAAACGGGGGCATTCGTATTGCGGCGTTAGAGGTGAAATTCTTGGATCGCCGCAAGACGAGCTACTGCGAAAGCATTTGTCAAGAATGTTTTCATTAGTCAAGAACGAAAGTCAGAGGCGCGAAGACGATCAGATACCGTCGTAGTTCTGACCATAAACGATGCCATCCAGCGATCCGCAGGAGTTGCTTCGATGACTCTGCGGGCAGCTTCCGGGAAACCAAAGTTTTTGGGTTCCGGGGGAAGTATGGTTGCAAAGCTGAAACTTAAAGGAATTGACGGAAGGGCACCACCAGGAGTGGAGCCTGCGGCTTAATTTGACTCAACACGGGAAAACTCACCCGGTCCGGACACTGTAAGGATTGACAGATTGATAGCTCTTTCTTGATTCGGTGGGTGGTGGTGCATGGCCGTTCTTAGTTGGTGGAGCGATTTGTCTGGTTAATTCCGATAACGAACGAGACTCTAGCCTATTAAATAGTTCGCCAATTCCTTGATGCGTTGGCGCAACTTCTTAGAGGGACGAGTGGCGTTTAGCCACACGAGATTGAGCAATAACAGGTCTGTGATGCCCTTAGATGTCCGGGGCCGCACGCGCGCTACACTGAAGGAATCAGCGTGGATGCCTCCCTGGTCCGAAAGGATTGGGAAACCCGTTGAATCTCCTTCGTGCTAGGGATTGGGGCTTGTAATTCTTCCCCATGAACGAGGAATTCCCAGTAAGCGCGAGTCATAAGCTCGCGTTGATTACGTCCCTGCCCTTTGTACACACCGCCCGTCGCTACTATCGATTGAGCGGTTCAGTGAGGGCCTCGGATTGGTCTCGGTCTGGCGTGCAAGCGCCGGCACCGTTGGCCGAGAAGACGCTCGAACTCGATCGCTTGGAGAAAGTAAAAGTCGTAACAAGGTTTCCGTAGGTG

>SICBC2010KJ01D05

TTGTCTCAAAGATTAAGCCATGCATGTCTAAGTTCACACTATCTCACGGTGAAACCGCGAATGGCTCATTAAATCAGTCGAGGTTCCTTAGATGACACGATCCTACTTGGATAACTGTGGCAATTCTAGAGCTAATACATGCTTTTGAAGCTCCGACCTAAAGGGAAGAGCGCTTTTATTAGTTCAAAACCAATCGCTCGGCTTC--GGTCGGGTGTCCCCTTTGGTGACTCTGGATAACTTTGTGCTGATCGCATGGCC-CTAGCGCCGGCGACGCATCTTTCAAATGTCTGCCCTATCAAATGTCGATGGTACGTGACATGCCTACCATGTTTGTAACGGGTAACGGGGAATCAGGGTTCGATTCCGGAGAGGGAGCATGAGAAACGGCTACCACATCCAAGGAAGGCAGCAGGCGCGCAACTTACCCACTCCCGGCACGGGGAGGTAGTGACGAAAAATAACAATACGGGACTCTTTCGAGGCCCCGTAATTGGAATGAGTACACTTTAAACCCTTTAACGAGGATCTATTGGAGGGCAAGTCTGGTGCCAGCAGCCGCGGTAATTCCAGCTCCAATAGCGTATATTAAAGTTGTTGCAGTTAAAAAGCTCGTAGTTGGATCTCAGGCGCAGGCGGGTGGTCCGGCTCGCGCCGGCTCACTGCCCGTACTCCTGCCCTACCTGTTGTCGGCTCTCTCCCGCGGGTGCTCTTCACTGAGCGTCCCGGGTGGCCGGCGCGTTTACTTTGAAAAAATTAGAGTGTTCAAAGCAGG-CTAAATAGCCTGAATAATGGTGCATGGAATAATGGAATAGGACCTCGGTTCTATTTTGTTGGTTTTCGGAACTAGAGGTAATGATTAACAGGGACAAACGGGGGCATTCGTATTGCGGCGTTAGAGGTGAAATTCTTGGATCGCCGCAAGACGAGCTACTGCGAAAGCATTTGTCAAGAATGTTTTCATTAATCAAGAACGAAAGTCAGAGGCGCGAAGACGATCAGATACCGTCGTAGTTCTGACCATAAACGATGCCACCTAGCGATCCGCAGGAGTTGCTTCGATGACTCTGCGGGCAGCTTCCGGGAAACCAAAGGTTTTGGGTTCCGGGGGAAGTATGGTTGCAAAGCTGAAACTTAAAGGAATTGACGGAAGGGCACCACCAGGAGTGGAGCCTGCGGCTTAATTTGACTCAACACGGGAAAACTCACCCGGTCCGGACACTGTAAGGATTGACAGATTGATAGCTCTTTCTTGATTCGGTGGGTGGTGGTGCATGGCCGTTCTTAGTTGGTGGAGCGATTTGTCTGGTTAATTCCGATAACGAACGAGACTCTAGCCTATTAAATAGTTCGCCGATTCCTTGATGCGTCGGCGCAACTTCTTAGAGGGACGAGTGGCGTTTAGCCACACGAGATTGAGCAATAACAGGTCTGTGATGCCCTTAGATGTCCGGGGCCGCACGCGCGCTACACTGAAGGAATCAGCGTGGATGCCTCCCTGGTCCGAAAGGATTGGGAAACCCGTTGAATCTCCTTCGTGCTAGGGATTGGGGCTTGTAATTCTTCCCCATGAACGAGGAATTCCCAGTAAGCGCGAGTCATAAGCTCGCGTTGATTACGTCCCTGCCCTTTGTACACACCGCCCGTCGCTACTATCGATTGAGCGGTTCAGTGAGGGCCTCGGATTGGTCTCGGTCTGGCGTGCAAGCGCCGGCACCGTTGGCCGAGAAGACGCTCGAACTCGATCGCTTGGAGAAAGTAAAAGTCGTAACAAGGTTTCCGTAGGTG

>ZSM20090197

TTGTCTCAAAGATTAAGCCATGCATGTCTAAGTTCACACTATCTCACGGTGAAACCGCGAATGGCTCATTAAATCAGTCGAGGTTCCTTAGATGACACGATCCTACTTGGATAACTGTGGCAATTCTAGAGCTAATACATGCTTTTGAAGCTCCGACCTAAAGGGAAGAGCGCTTTTATTAGTTCAAAACCAATCGCTCGGCTTC--GGTCGGGTGTCCCCTTTGGTGACTCTGGATAACTTTGTGCTGATCGCATGGCC-CTAGCGCCGGCGACGCATCTTTCAAATGTCTGCCCTATCAAATGTCGATGGTACGTGACATGCCTACCATGTTTGTAACGGGTAACGGGGAATCAGGGTTCGATTCCGGAGAGGGAGCATGAGAAACGGCTACCACATCCAAGGAAGGCAGCAGGCGCGCAACTTACCCACTCCCGGCACGGGGAGGTAGTGACGAAAAATAACAATACGGGACTCTTTCGAGGCCCCGTAATTGGAATGAGTACACTTTAAACCCTTTAACGAGGATCTATTGGAGGGCAAGTCTGGTGCCAGCAGCCGCGGTAATTCCAGCTCCAATAGCGTATATTAAAGTTGTTGCAGTTAAAAAGCTCGTAGTTGGATCTCAGGCGCAGGCGGGTGGTCCGGCTCGCGCCGGCTCACTGCCCGTACTCCTGCCCTACCTGTTGTCGGCTCTCTCCCGCGGGTGCTCTTCACTGAGCGTCCCGGGTGGCCGGCGCGTTTACTTTGAAAAAATTAGAGTGTTCAAAGCAGG-CTAAATAGCCTGAATAATGGTGCATGGAATAATGGAATAGGACCTCGGTTCTATTTTGTTGGTTTTCGGAACTAGAGGTAATGATTAACAGGGACAAACGGGGGCATTCGTATTGCGGCGTTAGAGGTGAAATTCTTGGATCGCCGCAAGACGAGCTACTGCGAAAGCATTTGTCAAGAATGTTTTCATTAATCAAGAACGAAAGTCAGAGGCGCGAAGACGATCAGATACCGTCGTAGTTCTGACCATAAACGATGCCACCTAGCGATCCGCAGGAGTTGCTTCGATGACTCTGCGGGCAGCTTCCGGGAAACCAAAGGTTTTGGGTTCCGGGGGAAGTATGGTTGCAAAGCTGAAACTTAAAGGAATTGACGGAAGGGCACCACCAGGAGTGGAGCCTGCGGCTTAATTTGACTCAACACGGGAAAACTCACCCGGTCCGGACACTGTAAGGATTGACAGATTGATAGCTCTTTCTTGATTCGGTGGGTGGTGGTGCATGGCCGTTCTTAGTTGGTGGAGCGATTTGTCTGGTTAATTCCGATAACGAACGAGACTCTAGCCTATTAAATAGTTCGCCGATTCCTTGATGCGTCGGCGCAACTTCTTAGAGGGACGAGTGGCGTTTAGCCACACGAGATTGAGCAATAACAGGTCTGTGATGCCCTTAGATGTCCGGGGCCGCACGCGCGCTACACTGAAGGAATCAGCGTGGATGCCTCCCTGGTCCGAAAGGATTGGGAAACCCGTTGAATCTCCTTCGTGCTAGGGATTGGGGCTTGTAATTCTTCCCCATGAACGAGGAATTCCCAGTAAGCGCGAGTCATAAGCTCGCGTTGATTACGTCCCTGCCCTTTGTACACACCGCCCGTCGCTACTATCGATTGAGCGGTTCAGTGAGGGCCTCGGATTGGTCTCGGTCTGGCGTGCAAGCGCCGGCACCGTTGGCCGAGAAGACGCTCGAACTCGATCGCTTGGAGAAAGTAAAAGTCGTAACAAGGTTTCCGTAGGTG

>ZSM20071135

NNNNNTCAAAGATTAAGCCATGCATGTCTAAGTTCACACTATCTCACGGTGAAACCGCGAATGGCTCATTAAATCAGTCGAGGTTCCTTAGATGACACGATCCTACTTGGATAACTGTGGCAATTCTAGAGCTAATACATGCTTTTGAAGCTCCGACCTAAAGGGAAGAGCGCTTTTATTAGTTCAAAACCAATCGCTCGGCCTT-GTGTCGGGCGCCCACTTTGGTGACTCTGGATAACTTTGTGCTGATCGCATGGCC-CTGGCGCCGGCGACGCATCTTTCAAATGTCTGCCCTATCAAATGTCGATGGTACGTGACATGCCTACCATGTTTGTAACGGGTAACGGGGAATCAGGGTTCGATTCCGGAGAGGGAGCATGAGAAACGGCTACCACATCCAAGGAAGGCAGCAGGCGCGCAACTTACCCACTCCCGGCACGGGGAGGTAGTGACGAAAAATAACAATACGGGACTCTTTCGAGGCCCCGTAATTGGAATGAGTACACTTTAAACCCTTTAACGAGGATCTATTGGAGGGCAAGTCTGGTGCCAGCAGCCGCGGTAATTCCAGCTCCAATAGCGTATATTAAAGTTGTTGCAGTTAAAAAGCTCGTAGTTGGATCTCAGGCGCAGGCGGGTGGTCCGGCTCGCGCCGGCTCACTGCCCGTACTCCTGCCCTACCTGTTGTCGGCTCTCTCCCGCGGGTGCTCTTCACTGAGCGTCCCGGGTGGCCGGCGCGTTTACTTTGAAAAAATTAGAGTGTTCAAAGCAGG-CTAAATAGCCTGAATAATGGTGCATGGAATAATGGAATAGGACCTCGGTTCTATTTTGTTGGTTTTCGGAACTAGAGGTAATGATTAACAGGGACAAACGGGGGCATTCGTATTGCTGCGTTAGAGGTGAAATTCTTGGATCGCAGCAAGACGAGCTACTGCGAAAGCATTTGTCAAGAATGTTTTCATTAATCAAGAACGAAAGTCAGAGGCGCGAAGACGATCAGATACCGTCGTAGTTCTGACCATAAACGATGCCACCTAGCGATCCGCAGGAGTTGCTTCGATGACTCTGCGGGCAGCTTCCGGGAAACCAAAGGTTTTGGGTTCCGGGGGAAGTATGGTTGCAAAGCTGAAACTTAAAGGAATTGACGGAAGGGCACCACCAGGAGTGGAGCCTGCGGCTTAATTTGACTCAACACGGGAAAACTCACCCGGTCCGGACACTGTAAGGATTGACAGATTGATAGCTCTTTCTTGATTCGGTGGGTGGTGGTGCATGGCCGTTCTTAGTTGGTGGAGCGATTTGTCTGGTTAATTCCGATAACGAACGAGACTCTAGCCTATTAAATAGTTCGCCGATTCTTTGATGCGTCGGCGCAACTTCTTAGAGGGACGAGTGGCGTTTAGCCACACGAGATTGAGCAATAACAGGTCTGTGATGCCCTTAGATGTCCGGGGCCGCACGCGCGCTACACTGAAGGAATCAGCGTGGATGCCTCCCTGGTCCGAAAGGATTGGGAAACCCGTTGAATCTCCTTCGTGCTAGGGATTGGGGCTTGTAATTCTTCCCCATGAACGAGGAATTCCCAGTAAGCGCGAGTCATAAGCTCGCGTTGATTACGTCCCTGCCCTTTGTACACACCGCCCGTCGCTACTATCGATTGAGCGGTTCAGTGAGGGCCTCGGATTGGTCTCGGTCTGGCGTGCAAGCGCCGGCACCGTTGGCCGAGAAGACGCTCGAACTCGATCGCTTGGAGAAAGTAAAAGTCGTAACAAGGTTTCCGTAGGTG

>ZSM20100391

TTGTCTCAAAGATTAAGCCATGCATGTCTAAGTTCACACTATCTCACGGTGAAACCGCGAATGGCTCATTAAATCAGTCGAGGTTCCTTAGATGACACGATCCTACTTGGATAACTGTGGCAATTCTAGAGCTAATACATGCTTTTGAAGCTCCGACCTAAAGGGAAGAGCGCTTTTATTAGTTCAAAACCAATCGCTCGGCCTT-GTGTCGGGCGCCCACTTTGGTGACTCTGGATAACTTTGTGCTGATCGCATGGCC-CTGGCGCCGGCGACGCATCTTTCAAATGTCTGCCCTATCAAATGTCGATGGTACGTGACATGCCTACCATGTTTGTAACGGGTAACGGGGAATCAGGGTTCGATTCCGGAGAGGGAGCATGAGAAACGGCTACCACATCCAAGGAAGGCAGCAGGCGCGCAACTTACCCACTCCCGGCACGGGGAGGTAGTGACGAAAAATAACAATACGGGACTCTTTCGAGGCCCCGTAATTGGAATGAGTACACTTTAAACCCTTTAACGAGGATCTATTGGAGGGCAAGTCTGGTGCCAGCAGCCGCGGTAATTCCAGCTCCAATAGCGTATATTAAAGTTGTTGCAGTTAAAAAGCTCGTAGTTGGATCTCAGGCGCAGGCGGGTGGTCCGGCTCGCGCCGGCTCACTGCCCGTACTCCTGCCCTACCTGTTGTCGGCTCTCTCCCGCGGGTGCTCTTCACTGAGCGTCCCGGGTGGCCGGCGCGTTTACTTTGAAAAAATTAGAGTGTTCAAAGCAGG-CTAAATAGCCTGAATAATGGTGCATGGAATAATGGAATAGGACCTCGGTTCTATTTTGTTGGTTTTCGGAACTAGAGGTAATGATTAACAGGGACAAACGGGGGCATTCGTATTGCTGCGTTAGAGGTGAAATTCTTGGATCGCAGCAAGACGAGCTACTGCGAAAGCATTTGTCAAGAATGTTTTCATTAATCAAGAACGAAAGTCAGAGGCGCGAAGACGATCAGATACCGTCGTAGTTCTGACCATAAACGATGCCACCTAGCGATCCGCAGGAGTTGCTTCGATGACTCTGCGGGCAGCTTCCGGGAAACCAAAGGTTTTGGGTTCCGGGGGAAGTATGGTTGCAAAGCTGAAACTTAAAGGAATTGACGGAAGGGCACCACCAGGAGTGGAGCCTGCGGCTTAATTTGACTCAACACGGGAAAACTCACCCGGTCCGGACACTGTAAGGATTGACAGATTGATAGCTCTTTCTTGATTCGGTGGGTGGTGGTGCATGGCCGTTCTTAGTTGGTGGAGCGATTTGTCTGGTTAATTCCGATAACGAACGAGACTCTAGCCTATTAAATAGTTCGCCGATTCTTTGATGCGTCGGCGCAACTTCTTAGAGGGACGAGTGGCGTTTAGCCACACGAGATTGAGCAATAACAGGTCTGTGATGCCCTTAGATGTCCGGGGCCGCACGCGCGCTACACTGAAGGAATCAGCGTGGATGCCTCCCTGGTCCGAAAGGATTGGGAAACCCGTTGAATCTCCTTCGTGCTAGGGATTGGGGCTTGTAATTCTTCCCCATGAACGAGGAATTCCCAGTAAGCGCGAGTCATAAGCTCGCGTTGATTACGTCCCTGCCCTTTGTACACACCGCCCGTCGCTACTATCGATTGAGCGGTTCAGTGAGGGCCTCGGATTGGTCTCGGTCTGGCGTGCAAGCGCCGGCACCGTTGGCCGAGAAGACGCTCGAACTCGATCGCTTGGAGAAAGTAAAAGTCGTAACAAGGTTTCCGTAGGTG

>ZSM20110723

TTGTCTCAAAGATTAAGCCATGCATGTCTAAGTTCACACTATCTCACGGTGAAACCGCGAATGGCTCATTAAATCAGTCGAGGTTCCTTAGATGACACGATCCTACTTGGATAACTGTGGCAATTCTAGAGCTAATACATGCCTCTGAAGCTCCGACCTGCTGGGAAGAGCGCTTTTATTAGTTCAAAACCAATCGTGCGGTCGGCCGGCCGCACGCCCCGTTTGGTGACTCTGGATAACTTTGTGCTGATCGCATGGCCTCCTGCGCCGGCGACGCATCTTTCAAATGTCTGCCCTATCAAATGTCGATGGTACGTGACATGCCTACCATGTTTGTAACGGGTAACGGGGAATCAGGGTTCGATTCCGGAGAGGGAGCATGAGAAACGGCTACCACATCCAAGGAAGGCAGCAGGCGCGCAACTTACCCACTCCCGGCACGGGGAGGTAGTGACGAAAAATAACAATACGGGACTCTTTCGAGGCCCCGTAATTGGAATGAGTACACTTTAAACCCTTTAACGAGGATCTATTGGAGGGCAAGTCTGGTGCCAGCAGCCGCGGTAATTCCAGCTCCAATAGCGTATATTAAAGTTGTTGCAGTTAAAAAGCTCGTAGTTGGATCTCAGGCGCAGGCGGGTGGTCCGGCTCGCGCCGGCTCACTGCCCGTACTCCTGCCCTACCTGTTGTCGGCTCTCTCCCGCGGGTGCTCTTCGCTGAGCGTCCCGGGTGGCCGGCGCGTTTACTTTGAAAAAATTAGAGTGTTCAAAGCAGGCCTCGGCTGCCTGAATAATGGTGCATGGAATAATGGAATAGGACCTCGGTTCTATTTTGTTGGTTTTCGGAACTAGAGGTAATGATTAACAGGGACAAACGGGGGCATTCGTATTGCTGCGTTAGAGGTGAAATTCTTGGATCGCAGCAAGACGAGCTACTGCGAAAGCATTTGTCAAGAATGTTTTCATTAATCAAGAACGAAAGTCAGAGGCGCGAAGACGATCAGATACCGTCGTAGTTCTGACCATAAACGATGCCAGCTAGCGATCCGCAGGAGTTGCTTCGATGACTCTGCGGGCAGCTTCCGGGAAACCAAAGTTTTTGGGTTCCGGGGGAAGTATGGTTGCAAAGCTGAAACTTAAAGGAATTGACGGAAGGGCACCACCAGGAGTGGAGCCTGCGGCTTAATTTGACTCAACACGGGAAAACTCACCCGGTCCGGACACTGTAAGGATTGACAGATTGATAGCTCTTTCTTGATTCGGTGGGTGGTGGTGCATGGCCGTTCTTAGTTGGTGGAGCGATTTGTCTGGTTAATTCCGATAACGAACGAGACTCTAGCCTATTAAATAGTTCGCCGATTCCTTGATGCGTCGGCGCAACTTCTTAGAGGGACGAGTGGCGTTTAGCCACACGAGATTGAGCAATAACAGGTCTGTGATGCCCTTAGATGTCCGGGGCCGCACGCGCGCTACACTGAAGGAATCAGCGTGGATGCCTCCCTGGTCCGAAAGGATTGGGAAACCCGTTGAATCTCCTTCGTGCTAGGGATTGGGGCTTGTAATTCTTCCCCATGAACGAGGAATTCCCAGTAAGCGCGAGTCATAAGCTCGCGTTGATTACGTCCCTGCCCTTTGTACACACCGCCCGTCGCTACTATCGATTGAGCGGTTCAGTGAGGGCCTCGGATTGGTCTCGGTCTGGTGTGCAAGCGCCGGCACCGTTGGCCGAGAAGACGCTCGAACTCGATCGCTTGGAGAAAGTAAAAGTCGTAACAAGGTTTCCGTAGGTG

>ZSM20110722

TTGTCTCAAAGATTAAGCCATGCATGTCTAAGTTCACACTATCTCACGGTGAAACCGCGAATGGCTCATTAAATCAGTCGAGGTTCCTTAGATGACACGATCCTACTTGGATAACTGTGGCAATTCTAGAGCTAATACATGCCTCTGAAGCTCCGACCTGCTGGGAAGAGCGCTTTTATTAGTTCAAAACCAATCGTGCGGTCGGCCGGCCGCACGCCCCGTTTGGTGACTCTGGATAACTTTGTGCTGATCGCATGGCCTCCTGCGCCGGCGACGCATCTTTCAAATGTCTGCCCTATCAAATGTCGATGGTACGTGACATGCCTACCATGTTTGTAACGGGTAACGGGGAATCAGGGTTCGATTCCGGAGAGGGAGCATGAGAAACGGCTACCACATCCAAGGAAGGCAGCAGGCGCGCAACTTACCCACTCCCGGCACGGGGAGGTAGTGACGAAAAATAACAATACGGGACTCTTTCGAGGCCCCGTAATTGGAATGAGTACACTTTAAACCCTTTAACGAGGATCTATTGGAGGGCAAGTCTGGTGCCAGCAGCCGCGGTAATTCCAGCTCCAATAGCGTATATTAAAGTTGTTGCAGTTAAAAAGCTCGTAGTTGGATCTCAGGCGCAGGCGGGTGGTCCGGCTCGCGCCGGCTCACTGCCCGTACTCCTGCCCTACCTGTTGTCGGCTCTCTCCCGCGGGTGCTCTTCGCTGAGCGTCCCGGGTGGCCGGCGCGTTTACTTTGAAAAAATTAGAGTGTTCAAAGCAGGCCTCGGCTGCCTGAATAATGGTGCATGGAATAATGGAATAGGACCTCGGTTCTATTTTGTTGGTTTTCGGAACTAGAGGTAATGATTAACAGGGACAAACGGGGGCATTCGTATTGCTGCGTTAGAGGTGAAATTCTTGGATCGCAGCAAGACGAGCTACTGCGAAAGCATTTGTCAAGAATGTTTTCATTAATCAAGAACGAAAGTCAGAGGCGCGAAGACGATCAGATACCGTCGTAGTTCTGACCATAAACGATGCCAGCTAGCGATCCGCAGGAGTTGCTTCGATGACTCTGCGGGCAGCTTCCGGGAAACCAAAGTTTTTGGGTTCCGGGGGAAGTATGGTTGCAAAGCTGAAACTTAAAGGAATTGACGGAAGGGCACCACCAGGAGTGGAGCCTGCGGCTTAATTTGACTCAACACGGGAAAACTCACCCGGTCCGGACACTGTAAGGATTGACAGATTGATAGCTCTTTCTTGATTCGGTGGGTGGTGGTGCATGGCCGTTCTTAGTTGGTGGAGCGATTTGTCTGGTTAATTCCGATAACGAACGAGACTCTAGCCTATTAAATAGTTCGCCGATTCCTTGATGCGTCGGCGCAACTTCTTAGAGGGACGAGTGGCGTTTAGCCACACGAGATTGAGCAATAACAGGTCTGTGATGCCCTTAGATGTCCGGGGCCGCACGCGCGCTACACTGAAGGAATCAGCGTGGATGCCTCCCTGGTCCGAAAGGATTGGGAAACCCGTTGAATCTCCTTCGTGCTAGGGATTGGGGCTTGTAATTCTTCCCCATGAACGAGGAATTCCCAGTAAGCGCGAGTCATAAGCTCGCGTTGATTACGTCCCTGCCCTTTGTACACACCGCCCGTCGCTACTATCGATTGAGCGGTTCAGTGAGGGCCTCGGATTGGTCTCGGTCTGGTGTGCAAGCGCCGGCACCGTTGGCCGAGAAGACGCTCGAACTCGATCGCTTGGAGAAAGTAAAAGTCGTAACAAGGTTTCCGTAGGTG

>SICBC2010KJ01E03

TTGTCTCAAAGATTAAGCCATGCATGTCTAAGTTCACACTATCTCACGGTGAAACCGCGAATGGCTCATTAAATCAGTCGAGGTTCCTTAGATGACACGATCCTACTTGGATAACTGTGGCAATTCTAGAGCTAATACATGCCTCTGAAGCTCCGACCTGCTGGGAAGAGCGCTTTTATTAGTTCAAAACCAATCGTGCGGTCGGCCGGCCGCACGCCCCGTTTGGTGACTCTGGATAACTTTGTGCTGATCGCATGGCCTCCTGCGCCGGCGACGCATCTTTCAAATGTCTGCCCTATCAAATGTCGATGGTACGTGACATGCCTACCATGTTTGTAACGGGTAACGGGGAATCAGGGTTCGATTCCGGAGAGGGAGCATGAGAAACGGCTACCACATCCAAGGAAGGCAGCAGGCGCGCAACTTACCCACTCCCGGCACGGGGAGGTAGTGACGAAAAATAACAATACGGGACTCTTTCGAGGCCCCGTAATTGGAATGAGTACACTTTAAACCCTTTAACGAGGATCTATTGGAGGGCAAGTCTGGTGCCAGCAGCCGCGGTAATTCCAGCTCCAATAGCGTATATTAAAGTTGTTGCAGTTAAAAAGCTCGTAGTTGGATCTCAGGCGCAGGCGGGTGGTCCGGCTCGCGCCGGCTCACTGCCCGTACTCCTGCCCTACCTGTTGTCGGCTCTCTCCCGCGGGTGCTCTTCGCTGAGCGTCCCGGGTGGCCGGCGCGTTTACTTTGAAAAAATTAGAGTGTTCAAAGCAGGCCTCGGCTGCCTGAATAATGGTGCATGGAATAATGGAATAGGACCTCGGTTCTATTTTGTTGGTTTTCGGAACTAGAGGTAATGATTAACAGGGACAAACGGGGGCATTCGTATTGCTGCGTTAGAGGTGAAATTCTTGGATCGCAGCAAGACGAGCTACTGCGAAAGCATTTGTCAAGAATGTTTTCATTAATCAAGAACGAAAGTCAGAGGCGCGAAGACGATCAGATACCGTCGTAGTTCTGACCATAAACGATGCCAGCTAGCGATCCGCAGGAGTTGCTTCGATGACTCTGCGGGCAGCTTCCGGGAAACCAAAGTTTTTGGGTTCCGGGGGAAGTATGGTTGCAAAGCTGAAACTTAAAGGAATTGACGGAAGGGCACCACCAGGAGTGGAGCCTGCGGCTTAATTTGACTCAACACGGGAAAACTCACCCGGTCCGGACACTGTAAGGATTGACAGATTGATAGCTCTTTCTTGATTCGGTGGGTGGTGGTGCATGGCCGTTCTTAGTTGGTGGAGCGATTTGTCTGGTTAATTCCGATAACGAACGAGACTCTAGCCTATTAAATAGTTCGCCGATTCCTTGATGCGTCGGCGCAACTTCTTAGAGGGACGAGTGGCGTTTAGCCACACGAGATTGAGCAATAACAGGTCTGTGATGCCCTTAGATGTCCGGGGCCGCACGCGCGCTACACTGAAGGAATCAGCGTGGATGCCTCCCTGGTCCGAAAGGATTGGGAAACCCGTTGAATCTCCTTCGTGCTAGGGATTGGGGCTTGTAATTCTTCCCCATGAACGAGGAATTCCCAGTAAGCGCGAGTCATAAGCTCGCGTTGATTACGTCCCTGCCCTTTGTACACACCGCCCGTCGCTACTATCGATTGAGCGGTTCAGTGAGGGCCTCGGATTGGTCTCGGTCTGGTGTGCAAGCGCCGGCACCGTTGGCCGAGAAGACGCTCGAACTCGATCGCTTGGAGAAAGTAAAAGTCGTAACAAGGTTTCCGTAGGTG

>ZSM20090198

TTGTCTCAAAGATTAAGCCATGCATGTCTAAGTTCACACTATCTCACGGTGAAACCGCGAATGGCTCATTAAATCAGTCGAGGTTCCTTAGATGACACGATCCTACTTGGATAACTGTGGCAATTCTAGAGCTAATACATGCCTCTGAAGCTCCGACCTGCTGGGAAGAGCGCTTTTATTAGTTCAAAACCAATCGTGCGGTCGGCCGGCCGCACGCCCCGTTTGGTGACTCTGGATAACTTTGTGCTGATCGCATGGCCTCCTGCGCCGGCGACGCATCTTTCAAATGTCTGCCCTATCAAATGTCGATGGTACGTGACATGCCTACCATGTTTGTAACGGGTAACGGGGAATCAGGGTTCGATTCCGGAGAGGGAGCATGAGAAACGGCTACCACATCCAAGGAAGGCAGCAGGCGCGCAACTTACCCACTCCCGGCACGGGGAGGTAGTGACGAAAAATAACAATACGGGACTCTTTCGAGGCCCCGTAATTGGAATGAGTACACTTTAAACCCTTTAACGAGGATCTATTGGAGGGCAAGTCTGGTGCCAGCAGCCGCGGTAATTCCAGCTCCAATAGCGTATATTAAAGTTGTTGCAGTTAAAAAGCTCGTAGTTGGATCTCAGGCGCAGGCGGGTGGTCCGGCTCGCGCCGGCTCACTGCCCGTACTCCTGCCCTACCTGTTGTCGGCTCTCTCCCGCGGGTGCTCTTCGCTGAGCGTCCCGGGTGGCCGGCGCGTTTACTTTGAAAAAATTAGAGTGTTCAAAGCAGGCCTCGGCTGCCTGAATAATGGTGCATGGAATAATGGAATAGGACCTCGGTTCTATTTTGTTGGTTTTCGGAACTAGAGGTAATGATTAACAGGGACAAACGGGGGCATTCGTATTGCTGCGTTAGAGGTGAAATTCTTGGATCGCAGCAAGACGAGCTACTGCGAAAGCATTTGTCAAGAATGTTTTCATTAATCAAGAACGAAAGTCAGAGGCGCGAAGACGATCAGATACCGTCGTAGTTCTGACCATAAACGATGCCAGCTAGCGATCCGCAGGAGTTGCTTCGATGACTCTGCGGGCAGCTTCCGGGAAACCAAAGTTTTTGGGTTCCGGGGGAAGTATGGTTGCAAAGCTGAAACTTAAAGGAATTGACGGAAGGGCACCACCAGGAGTGGAGCCTGCGGCTTAATTTGACTCAACACGGGAAAACTCACCCGGTCCGGACACTGTAAGGATTGACAGATTGATAGCTCTTTCTTGATTCGGTGGGTGGTGGTGCATGGCCGTTCTTAGTTGGTGGAGCGATTTGTCTGGTTAATTCCGATAACGAACGAGACTCTAGCCTATTAAATAGTTCGCCGATTCCTTGATGCGTCGGCGCAACTTCTTAGAGGGACGAGTGGCGTTTAGCCACACGAGATTGAGCAATAACAGGTCTGTGATGCCCTTAGATGTCCGGGGCCGCACGCGCGCTACACTGAAGGAATCAGCGTGGATGCCTCCCTGGTCCGAAAGGATTGGGAAACCCGTTGAATCTCCTTCGTGCTAGGGATTGGGGCTTGTAATTCTTCCCCATGAACGAGGAATTCCCAGTAAGCGCGAGTCATAAGCTCGCGTTGATTACGTCCCTGCCCTTTGTACACACCGCCCGTCGCTACTATCGATTGAGCGGTTCAGTGAGGGCCTCGGATTGGTCTCGGTCTGGTGTGCAAGCGCCGGCACCGTTGGCCGAGAAGACGCTCGAACTCGATCGCTTGGAGAAAGTAAAAGTCGTAACAAGGTTTCCGTAGGTG

>ZSM20071133

TTGTCTCAAAGATTAAGCCATGCATGTCTAAGTTCACACTATCTCACGGTGAAACCGCGAATGGCTCATTAAATCAGTCGAGGTTCCTTAGATGACACGATCCTACTTGGATAACTGTGGCAATTCTAGAGCTAATACATGCCTCTGAAGCTCCGACCTCACGGGAAGAGCGCTTTTATTAGTTCAAAACCAATCGTGCGGTTCCTGGGCCGCGCGCCCACTTTGGTGACTCTGGATAACTTTGTGCTGATCGCATGGCCTCCTGCGCCGGCGACGCATCTTTCAAATGTCTGCCCTATCAAATGTCGATGGTACGTGACATGCCTACCATGTTTGTAACGGGTAACGGGGAATCAGGGTTCGATTCCGGAGAGGGAGCATGAGAAACGGCTACCACATCCAAGGAAGGCAGCAGGCGCGCAACTTACCCACTCCCGGCACGGGGAGGTAGTGACGAAAAATAACAATACGGGACTCTTTCGAGGCCCCGTAATTGGAATGAGTACACTTTAAACCCTTTAACGAGGATCTATTGGAGGGCAAGTCTGGTGCCAGCAGCCGCGGTAATTCCAGCTCCAATAGCGTATATTAAAGTTGTTGCAGTTAAAAAGCTCGTAGTTGGATCTCAGGCGCAGGCGGGTGGTCCGGCTCGCGCCGGCTCACTGCCCGTACTCCTGCCCTACCTGTTGTCGGCTCTCTCCCGCGGGTGCTCTTCACTGAGCGTCCCGGGTGGCCGGCGCGTTTACTTTGAAAAAATTAGAGTGTTCAAAGCAGGCCTCGGCTGCCTGAATAATGGTGCATGGAATAATGGAATAGGACCTCGGTTCTATTTTGTTGGTTTTCGGAACTAGAGGTAATGATTAACAGGGACAAACGGGGGCATTCGTATTGCTGCGTTAGAGGTGAAATTCTTGGATCGCAGCAAGACGAGCTACTGCGAAAGCATTTGTCAAGAATGTTTTCATTAATCAAGAACGAAAGTCAGAGGCGCGAAGACGATCAGATACCGTCGTAGTTCTGACCATAAACGATGCCAGCTAGCGATCCGCAGGAGTTGCTTCGATGACTCTGCGGGCAGCTTCCGGGAAACCAAAGTTTTTGGGTTCCGGGGGAAGTATGGTTGCAAAGCTGAAACTTAAAGGAATTGACGGAAGGGCACCACCAGGAGTGGAGCCTGCGGCTTAATTTGACTCAACACGGGAAAACTCACCCGGTCCGGACACTGTAAGGATTGACAGATTGATAGCTCTTTCTTGATTCGGTGGGTGGTGGTGCATGGCCGTTCTTAGTTGGTGGAGCGATTTGTCTGGTTAATTCCGATAACGAACGAGACTCTAGCCTATTAAATAGTTCGCCGATTCCTTGATGCGTCGGCGCAACTTCTTAGAGGGACGAGTGGCGTTTAGCCACACGAGATTGAGCAATAACAGGTCTGTGATGCCCTTAGATGTCCGGGGCCGCACGCGCGCTACACTGAAGGAATCAGCGTGGATGCCTCCCTGGTCCGAAAGGATTGGGAAACCCGTTGAATCTCCTTCGTGCTAGGGATTGGGGCTTGTAATTCTTCCCCATGAACGAGGAATTCCCAGTAAGCGCGAGTCATAAGCTCGCGTTGATTACGTCCCTGCCCTTTGTACACACCGCCCGTCGCTACTATCGATTGAGCGGTTCAGTGAGGGCCTCGGATTGGTCTCGGTCTGGCGTGCAAGCGCCGGCACCGTTGGCCGAGAAGACGCTCGAACTCGATCGCTTGGAGAAAGTAAAAGTCGTAACAAGGTTTNNNNNNNNN

>ZSM20080953

TTGTCTCAAAGATTAAGCCATGCATGTCTAAGTTCACACTATCTCACGGTGAAACCGCGAATGGCTCATTAAATCAGTCGAGGTTCCTTAGATGACACGATCCTACTTGGATAACTGTGGCAATTCTAGAGCTAATACATGCCTCTGAAGCTCCGACCCGCGGGGAAGAGCGCTTTTATTAGTTCAAAACCAATCGTGCGGTTCCTGGGCCGCACGCCCACTTTGGTGACTCTGGATAACTTTGTGCTGATCGCATGGCCTCCTGCGCCGGCGACGCATCTTTCAAATGTCTGCCCTATCAAATGTCGATGGTACGTGACATGCCTACCATGTTTGTAACGGGTAACGGGGAATCAGGGTTCGATTCCGGAGAGGGAGCATGAGAAACGGCTACCACATCCAAGGAAGGCAGCAGGCGCGCAACTTACCCACTCCCGGCACGGGGAGGTAGTGACGAAAAATAACAATACGGGACTCTTTCGAGGCCCCGTAATTGGAATGAGTACACTTTAAACCCTTTAACGAGGATCTATTGGAGGGCAAGTCTGGTGCCAGCAGCCGCGGTAATTCCAGCTCCAATAGCGTATATTAAAGTTGTTGCAGTTAAAAAGCTCGTAGTTGGATCTCAGGCGCAGGCGGGTGGTCCGGCTCGCGCCGGCTCACTGCCCGTACTCCTGCCCTACCTGTTGTCGGCTCTCTCCCGCGGGTGCTCTTCACTGAGCGTCCCGGGTGGCCGGCGCGTTTACTTTGAAAAAATTAGAGTGTTCAAAGCAGGCCTCGGCTGCCTGAATAATGGTGCATGGAATAATGGAATAGGACCTCGGTTCTATTTTGTTGGTTTTCGGAACTAGAGGTAATGATTAACAGGGACAAACGGGGGCATTCGTATTGCTGCGTTAGAGGTGAAATTCTTGGATCGCAGCAAGACGAGCTACTGCGAAAGCATTTGTCAAGAATGTTTTCATTAATCAAGAACGAAAGTCAGAGGCGCGAAGACGATCAGATACCGTCGTAGTTCTGACCATAAACGATGCCAGCTAGCGATCCGCAGGAGTTGCTTCGATGACTCTGCGGGCAGCTTCCGGGAAACCAAAGTTTTTGGGTTCCGGGGGAAGTATGGTTGCAAAGCTGAAACTTAAAGGAATTGACGGAAGGGCACCACCAGGAGTGGAGCCTGCGGCTTAATTTGACTCAACACGGGAAAACTCACCCGGTCCGGACACTGTAAGGATTGACAGATTGATAGCTCTTTCTTGATTCGGTGGGTGGTGGTGCATGGCCGTTCTTAGTTGGTGGAGCGATTTGTCTGGTTAATTCCGATAACGAACGAGACTCTAGCCTATTAAATAGTTCGCCGATTCCTTGATGCGTCGGCGCAACTTCTTAGAGGGACGAGTGGCGTTTAGCCACACGAGATTGAGCAATAACAGGTCTGTGATGCCCTTAGATGTCCGGGGCCGCACGCGCGCTACACTGAAGGAATCAGCGTGGATGCCTCCCTGGTCCGAAAGGATTGGGAAACCCGTTGAATCTCCTTCGTGCTAGGGATTGGGGCTTGTAATTCTTCCCCATGAACGAGGAATTCCCAGTAAGCGCGAGTCATAAGCTCGCGTTGATTACGTCCCTGCCCTTTGTACACACCGCCCGTCGCTACTATCGATTGAGCGGTTCAGTGAGGGCCTCGGATTGGTCTCGGTCTGGCGTGCAAGCGCCGGCACCGTTGGCCGAGAAGACGCTCGAACTCGATCGCTTGGAGAAAGTAAAAGTCGTAACAAGGTTTCNNNNNNNN

>ZSM20080054

TTGTCTCAAAGATTAAGCCATGCATGTCTAAGTTCACACTATCTCACGGTGAAACCGCGAATGGCTCATTAAATCAGTCGAGGTTCCTTAGATGACACGATCCTACTTGGATAACTGTGGCAATTCTAGAGCTAATACATGCCTCTGAAGCTCCGACCCGCGGGGAAGAGCGCTTTTATTAGTTCAAAACCAATCGTGCGGTTCCTGGGCCGCACGCCCACTTTGGTGACTCTGGATAACTTTGTGCTGATCGCATGGCCTCCTGCGCCGGCGACGCATCTTTCAAATGTCTGCCCTATCAAATGTCGATGGTACGTGACATGCCTACCATGTTTGTAACGGGTAACGGGGAATCAGGGTTCGATTCCGGAGAGGGAGCATGAGAAACGGCTACCACATCCAAGGAAGGCAGCAGGCGCGCAACTTACCCACTCCCGGCACGGGGAGGTAGTGACGAAAAATAACAATACGGGACTCTTTCGAGGCCCCGTAATTGGAATGAGTACACTTTAAACCCTTTAACGAGGATCTATTGGAGGGCAAGTCTGGTGCCAGCAGCCGCGGTAATTCCAGCTCCAATAGCGTATATTAAAGTTGTTGCAGTTAAAAAGCTCGTAGTTGGATCTCAGGCGCAGGCGGGTGGTCCGGCTCGCGCCGGCTCACTGCCCGTACTCCTGCCCTACCTGTTGTCGGCTCTCTCCCGCGGGTGCTCTTCACTGAGCGTCCCGGGTGGCCGGCGCGTTTACTTTGAAAAAATTAGAGTGTTCAAAGCAGGCCTCGGCTGCCTGAATAATGGTGCATGGAATAATGGAATAGGACCTCGGTTCTATTTTGTTGGTTTTCGGAACTAGAGGTAATGATTAACAGGGACAAACGGGGGCATTCGTATTGCTGCGTTAGAGGTGAAATTCTTGGATCGCAGCAAGACGAGCTACTGCGAAAGCATTTGTCAAGAATGTTTTCATTAATCAAGAACGAAAGTCAGAGGCGCGAAGACGATCAGATACCGTCGTAGTTCTGACCATAAACGATGCCAGCTAGCGATCCGCAGGAGTTGCTTCGATGACTCTGCGGGCAGCTTCCGGGAAACCAAAGTTTTTGGGTTCCGGGGGAAGTATGGTTGCAAAGCTGAAACTTAAAGGAATTGACGGAAGGGCACCACCAGGAGTGGAGCCTGCGGCTTAATTTGACTCAACACGGGAAAACTCACCCGGTCCGGACACTGTAAGGATTGACAGATTGATAGCTCTTTCTTGATTCGGTGGGTGGTGGTGCATGGCCGTTCTTAGTTGGTGGAGCGATTTGTCTGGTTAATTCCGATAACGAACGAGACTCTAGCCTATTAAATAGTTCGCCGATTCCTTGATGCGTCGGCGCAACTTCTTAGAGGGACGAGTGGCGTTTAGCCACACGAGATTGAGCAATAACAGGTCTGTGATGCCCTTAGATGTCCGGGGCCGCACGCGCGCTACACTGAAGGAATCAGCGTGGATGCCTCCCTGGTCCGAAAGGATTGGGAAACCCGTTGAATCTCCTTCGTGCTAGGGATTGGGGCTTGTAATTCTTCCCCATGAACGAGGAATTCCCAGTAAGCGCGAGTCATAAGCTCGCGTTGATTACGTCCCTGCCCTTTGTACACACCGCCCGTCGCTACTATCGATTGAGCGGTTCAGTGAGGGCCTCGGATTGGTCTCGGTCTGGCGTGCAAGCGCCGGCACCGTTGGCCGAGAAGACGCTCGAACTCGATCGCTTGGAGAAAGTAAAAGTCGTAACAAGGTTTCCGTAGGTG

>ZSM20110032

TTGTCTCAAAGATTAAGCCATGCATGTCTAAGTTCACACTATCTCACGGTGAAACCGCGAATGGCTCATTAAATCAGTCGAGGTTCCTTAGATGACACGATCCTACTTGGATAACTGTGGCAATTCTAGAGCTAATACATGCCTCTGAAGCTCCGACCTGCAGGGAAGAGCGCTTTTATTAGTTCAAAACCAATCGCGCGGTTCCCGGGCCGCGCGCCCACTTTGGTGACTCTGGATAACTTTGTGCTGATCGCATGGCCTCCTGCGCCGGCGACGCATCTTTCAAATGTCTGCCCTATCAAATGTCGATGGTACGTGACATGCCTACCATGTTTGTAACGGGTAACGGGGAATCAGGGTTCGATTCCGGAGAGGGAGCATGAGAAACGGCTACCACATCCAAGGAAGGCAGCAGGCGCGCAACTTACCCACTCCCGGCACGGGGAGGTAGTGACGAAAAATAACAATACGGGACTCTTTCGAGGCCCCGTAATTGGAATGAGTACACTTTAAACCCTTTAACGAGGATCTATTGGAGGGCAAGTCTGGTGCCAGCAGCCGCGGTAATTCCAGCTCCAATAGCGTATATTAAAGTTGTTGCAGTTAAAAAGCTCGTAGTTGGATCTCAGGCGCAGGCGGGTGGTCCGGCTCGCGCCGGCTCACTGCCCGTACTCCTGCCCTACCTGTTGTCGGCTCTCTCCCGCGGGTGCTCTTCGCTGAGCGTCCCGGGTGGCCGGCGCGTTTACTTTGAAAAAATTAGAGTGTTCAAAGCAGG-CTATTCAGCCTGAATAATGGTGCATGGAATAATGGAATAGGACCTCGGTTCTATTTTGTTGGTTTTCGGAACTAGAGGTAATGATTAACAGGGACAAACGGGGGCATTCGTATTGCTGCGTTAGAGGTGAAATTCTTGGATCGCAGCAAGACGAGCTACTGCGAAAGCATTTGTCAAGAATGTTTTCATTAATCAAGAACGAAAGTCAGAGGCGCGAAGACGATCAGATACCGTCGTAGTTCTGACCATAAACGATGCCAGCTAGCGATCCGCAGGAGTTGCTTCGATGACTCTGCGGGCAGCTTCCGGGAAACCAAAGTTTTTGGGTTCCGGGGGAAGTATGGTTGCAAAGCTGAAACTTAAAGGAATTGACGGAAGGGCACCACCAGGAGTGGAGCCTGCGGCTTAATTTGACTCAACACGGGAAAACTCACCCGGTCCGGACACTGTAAGGATTGACAGATTGATAGCTCTTTCTTGATTCGGTGGGTGGTGGTGCATGGCCGTTCTTAGTTGGTGGAGCGATTTGTCTGGTTAATTCCGATAACGAACGAGACTCTAGCCTATTAACTAGTTCGCCGATTCCTTGATGCGTCGGCGCAACTTCTTAGAGGGACGAGTGGCGTTTAGCCACACGAGATTGAGCAATAACAGGTCTGTGATGCCCTTAGATGTCCGGGGCCGCACGCGCGCTACACTGAAGGAATCAGCGTGGATGCCTCCCTGGTCCGAAAGGATTGGGAAACCCGTTGAATCTCCTTCGTGCTAGGGATTGGGGCTTGTAATTCTTCCCCATGAACGAGGAATTCCCAGTAAGCGCGAGTCATAAGCTCGCGTTGATTACGTCCCTGCCCTTTGTACACACCGCCCGTCGCTACTATCGATTGAGCGGTTCAGTGAGGGCCTCGGATTGGTCTCGGTCTGGCGTGCAAGCGCCGGCACCGTTGGCCGAGAAGACGCTCGAACTCGATCGCTTGGAGAAAGTAAAAGTCGTAACAAGGTTTCCGTAGGTG

>ZSM20081014

TTGTCTCAAAGATTAAGCCATGCATGTCTAAGTTCACACTATCTCACGGTGAAACCGCGAATGGCTCATTAAATCAGTCGAGGTTCCTTAGATGACACGATCCTACTTGGATAACTGTGGCAATTCTAGAGCTAATACATGCCTCTGAAGCTCCGACCTGCAGGGAAGAGCGCTTTTATTAGTTCAAAACCAATCGTGCGGTTCTCGGGCCGCGCGCCCACTTTGGTGACTCTGGATAACTTTGTGCTGATCGCATGGCCTCCTGCGCCGGCGACGCATCTTTCAAATGTCTGCCCTATCAAATGTCGATGGTACGTGACATGCCTACCATGTTTGTAACGGGTAACGGGGAATCAGGGTTCGATTCCGGAGAGGGAGCATGAGAAACGGCTACCACATCCAAGGAAGGCAGCAGGCGCGCAACTTACCCACTCCCGGCACGGGGAGGTAGTGACGAAAAATAACAATACGGGACTCTTTCGAGGCCCCGTAATTGGAATGAGTACACTTTAAACCCTTTAACGAGGATCTATTGGAGGGCAAGTCTGGTGCCAGCAGCCGCGGTAATTCCAGCTCCAATAGCGTATATTAAAGTTGTTGCAGTTAAAAAGCTCGTAGTTGGATCTCAGGCGCAGGCGGGTGGTCCGGCTCGCGCCGGCTCACTGCCCGTACTCCTGCCCTACCTGTTGTCGGCTCTCTCCCGCGGGTGCTCTTCACTGAGCGTCCCGGGTGGCCGGCGCGTTTACTTTGAAAAAATTAGAGTGTTCAAAGCAGG-CTATTCAGCCTGAATAATGGTGCATGGAATAATGGAATAGGACCTCGGTTCTATTTTGTTGGTTTTCGGAACTAGAGGTAATGATTAACAGGGACAAACGGGGGCATTCGTATTGCTGCGTTAGAGGTGAAATTCTTGGATCGCAGCAAGACGAGCTACTGCGAAAGCATTTGTCAAGAATGTTTTCATTAATCAAGAACGAAAGTCAGAGGCGCGAAGACGATCAGATACCGTCGTAGTTCTGACCATAAACGATGCCAGCTAGCGATCCGCAGGAGTTGCTTCGATGACTCTGCGGGCAGCTTCCGGGAAACCAAAGTTTTTGGGTTCCGGGGGAAGTATGGTTGCAAAGCTGAAACTTAAAGGAATTGACGGAAGGGCACCACCAGGAGTGGAGCCTGCGGCTTAATTTGACTCAACACGGGAAAACTCACCCGGTCCGGACACTGTAAGGATTGACAGATTGATAGCTCTTTCTTGATTCGGTGGGTGGTGGTGCATGGCCGTTCTTAGTTGGTGGAGCGATTTGTCTGGTTAATTCCGATAACGAACGAGACTCTAGCCTATTAAATAGTTCGCCGATTCCTTGATGCGTCGGCGCAACTTCTTAGAGGGACGAGTGGCGTTTAGCCACACGAGATTGAGCAATAACAGGTCTGTGATGCCCTTAGATGTCCGGGGCCGCACGCGCGCTACACTGAAGGAATCAGCGTGGATGCCTCCCTGGTCCGAAAGGATTGGGAAACCCGTTGAATCTCCTTCGTGCTAGGGATTGGGGCTTGTAATTCTTCCCCATGAACGAGGAATTCCCAGTAAGCGCGAGTCATAAGCTCGCGTTGATTACGTCCCTGCCCTTTGTACACACCGCCCGTCGCTACTATCGATTGAGCGGTTCAGTGAGGGCCTCGGATTGGTCTCGGTCTGGCGTGCAAGCGCCGGCACCGTTGGCCGAGAAGACGCTCGAACTCGATCGCTTGGAGAAAGTAAAAGTCGTAACAAGGTTTCCGTAGGTG

>ZSM20100379

TTGTCTCAAAGATTAAGCCATGCATGTCTAAGTTCACACTATCTCACGGTGAAACCGCGAATGGCTCATTAAATCAGTCGAGGTTCCTTAGATGACACGATCCTACTTGGATAACTGTGGCAATTCTAGAGCTAATACATGCCTCTGAAGCTCCGACCTGCAGGGAAGAGCGCTTTTATTAGTTCAAAACCAATCGTGCGGTTCTCGGGCCGCGCGCCCACTTTGGTGACTCTGGATAACTTTGTGCTGATCGCATGGCCTCCTGCGCCGGCGACGCATCTTTCAAATGTCTGCCCTATCAAATGTCGATGGTACGTGACATGCCTACCATGTTTGTAACGGGTAACGGGGAATCAGGGTTCGATTCCGGAGAGGGAGCATGAGAAACGGCTACCACATCCAAGGAAGGCAGCAGGCGCGCAACTTACCCACTCCCGGCACGGGGAGGTAGTGACGAAAAATAACAATACGGGACTCTTTCGAGGCCCCGTAATTGGAATGAGTACACTTTAAACCCTTTAACGAGGATCTATTGGAGGGCAAGTCTGGTGCCAGCAGCCGCGGTAATTCCAGCTCCAATAGCGTATATTAAAGTTGTTGCAGTTAAAAAGCTCGTAGTTGGATCTCAGGCGCAGGCGGGTGGTCCGGCTCGCGCCGGCTCACTGCCCGTACTCCTGCCCTACCTGTTGTCGGCTCTCTCCCGCGGGTGCTCTTCACTGAGCGTCCCGGGTGGCCGGCGCGTTTACTTTGAAAAAATTAGAGTGTTCAAAGCAGG-CTATTCAGCCTGAATAATGGTGCATGGAATAATGGAATAGGACCTCGGTTCTATTTTGTTGGTTTTCGGAACTAGAGGTAATGATTAACAGGGACAAACGGGGGCATTCGTATTGCTGCGTTAGAGGTGAAATTCTTGGATCGCAGCAAGACGAGCTACTGCGAAAGCATTTGTCAAGAATGTTTTCATTAATCAAGAACGAAAGTCAGAGGCGCGAAGACGATCAGATACCGTCGTAGTTCTGACCATAAACGATGCCAGCTAGCGATCCGCAGGAGTTGCTTCGATGACTCTGCGGGCAGCTTCCGGGAAACCAAAGTTTTTGGGTTCCGGGGGAAGTATGGTTGCAAAGCTGAAACTTAAAGGAATTGACGGAAGGGCACCACCAGGAGTGGAGCCTGCGGCTTAATTTGACTCAACACGGGAAAACTCACCCGGTCCGGACACTGTAAGGATTGACAGATTGATAGCTCTTTCTTGATTCGGTGGGTGGTGGTGCATGGCCGTTCTTAGTTGGTGGAGCGATTTGTCTGGTTAATTCCGATAACGAACGAGACTCTAGCCTATTAAATAGTTCGCCGATTCCTTGATGCGTCGGCGCAACTTCTTAGAGGGACGAGTGGCGTTTAGCCACACGAGATTGAGCAATAACAGGTCTGTGATGCCCTTAGATGTCCGGGGCCGCACGCGCGCTACACTGAAGGAATCAGCGTGGATGCCTCCCTGGTCCGAAAGGATTGGGAAACCCGTTGAATCTCCTTCGTGCTAGGGATTGGGGCTTGTAATTCTTCCCCATGAACGAGGAATTCCCAGTAAGCGCGAGTCATAAGCTCGCGTTGATTACGTCCCTGCCCTTTGTACACACCGCCCGTCGCTACTATCGATTGAGCGGTTCAGTGAGGGCCTCGGATTGGTCTCGGTCTGGCGTGCAAGCGCCGGCACCGTTGGCCGAGAAGACGCTCGAACTCGATCGCTTGGAGAAAGTAAAAGTCGTAACAAGGTTTCCGTAGGTG

>ZSM20100356_Palliohedyle_sp

NNNNNNNNNNNNNNNNNNNNNNNNNNNNNNNNNNNNNNNNNNNNNNNNNNNNNNNNNNNNNNNNNNNNNNNNNNNNNNNNNNNNNNNNNNNNNNNNNNNNNNNNNNNNNNNNNNNNNNNNNNNNNNNNNNNNNNNNNNNNNNNNNNNNNNNNNNNNNNNNNNNNNNNNNNNNNNNNNNNNNNNNNNNNNNNNNNNNNNNNNNNNNNNNNNNNNNNNNNNNNNNNNNNNNNNNNNNNNNNNNNNNNNNNNNNNNNNNNNNNNNNNNNNNNNNNNNNNNNNNNNNNNNNNNNNNNNNNNNNNNNNNNNNNNNNNNNNNNNNNNNNNNNNNNNNNNNNNNNNNNNNNNNNNNNNNNNNNNNNNNNNNNNNNNNNNNNNNNNNNNNNNNNNNNNNNNNNNNNNNNNNNNNNNNNNNNNNNNNNNNNNNNNNNNNNNNNNNNNNNNNNNNNNNNNNNNNNNNNNNNNNNNNNNNNNNNNNNNNNNNNNNNNNNNNNNNNNNNNNNNNNNNNNNNNNNNNNNNNNNNNNNNNNNNNNNNNNNNNNNNNNNNNNNNNNNNNNNNNNNNNNNNNNNNNNNNNNNNNNNNNNNNNNNNNNNNNNNNNNNNNNNNNNNNNNNNNNNNNNNNNNNNNNNNNNNNNNNNNNNNNNNNNNNNNNNNNNNNNNNNNNNNNNNNNNNNNNNNNNNNNNNNNNNNNNNNNNNNNNNNNNNNNNNNNNNNNNNNNNNNNNNNNNNNNNNNNNNNNNNNNNNNNNNNNNNNNNNNNNNNNNNNNNNNNNNNNNNNNNNNNNNNNNNNNNNNNNNNNNNNNNNNNNNNNNNNNNNNNNNNNNNNNNNNNNNNNNNNNNNNNNNNNNNNNNNNNNNNNNNNNNNNNNNNNNNNNNNNNNNNNNNNNNNNNNNNNNNNNNNNNNNNNNNNNNNNNNNNNNNNNNNNNNNNNNNNNNNNNNNNNNNNNNNNNNNNNNNNNNNNNNNNNNNNNNNNNNNNNNNNNNNNNNNNNNNNNNNNNNNNNNNNNNNNNNNNNNNNNNNNNNNNNNNNNNNNNNNNNNNNNNNNNNNNNNNNNNNNNNNNNNNNNNNNNNNNNNNNNNNNNNNNNNNNNNNNNNNNNNNNNNNNNNNNNNNNNNNNNNNNNNNNNNNCTGAAACTTTTAGGAATTGACGGAAGGGCACCACCAGGAGTGGAGCCTGCGGCTTAATTTGACTCAACACGGGAAAACTCACCCGGTCCGGACACTGTAAGGATTGACAGATTGATAGCTCTTTCTTGATTCGGTGGGTGGTGGTGCATGGCCGTTCTTAGTTGGTGGAGCGATTTGTCTGGTTAATTCCGATAACGAACGAGACTCTAGCCTATTAAATAGTTCGCCGGTCCCACGATGCGCCGGCGCAACTTCTTAGAGGGACGAGTGGCGTTTAGCCACACGAGATTGAGCAATAACAGGTCTGTGATGCCCTTAGATGTCCGGGGCCGCACGCGCGCTACACTGAAGGAATCAGCGTGGATGCCTCCCTGGTCCGAAAGGACTGGGAAACCCGTTGAATCTCCTTCGTGCTAGGGATTGGGGCTTGTAATTATTCCCCATGAACGAGGAATTCCCAGTAAGCGCGAGTCATAAGCTCGCGTTGATTACGTCCCTGCCCTTTGTACACACCGCCCGTCGCTACTATCGATTGAGCGGTTCAGTGAGGGCCTCGGATTGGTCACGGCCTGGTGTGCAAGCATCGGCGCCGTCGGCCGAGAAGACGCTCGAACTCGATCGCTTGGAGAAAGTAAAAGTCGTAACAAGGTTTCCGTAGGTG

>GenBankJF828036_Aiteng_ater

NNNNNNNNNNNNNNNNNNNNNNNNNNNNNNNNNNNNNNNNNNNNNNNNNNNNNNNNNNNNNNNNNNNNNNNNNNNNNNNNNNNNNNNNNNNNNNNNNNNNNNNNNNNNNNNNNNNNNNNNNNNNNNNNNNNNNNNNNNNNNNNNNNNNNNNNNNNNNNNNNNNNNNNNNNNNNNNNNNNNNNNNNNNNNNNNNNNNNNNNNNNNNNNNNNNNNNNNNNNNNNNNNNNNNNNNNNNNNNNNNNNNNNNNNNNNNNNNNNNNNNNNNNNNNNNNNNNNNNNNNNNNNNNNNNNNNNNNNNNNNNNNNNNNNNNNNNNNNNNNNNNNNNNNNNNNNNNNNNNNNNNNNNNNNNNNNNNNNNNNNNNNNNNNNNNNNNNNNNNNNNNNNNNNNNNNNNNNNNNNNNNNNNNNNNNNNNNNNNNNNNNNNNNNNNNNNNNNNNNNNNNNNNNNNNNNNNNNNNNNNNNACAATACGGGACTCTTTCGAGGCCCCGTAATTGGAATGAGTACACTTTAAACCCTTTAACGAGGATCTATTGGAGGGCAAGTCTGGTGCCAGCAGCCGCGGTAATTCCAGCTCCAATAGCGTATATTAAAGTTGTTGCAGTTAAAAAGCTCGTAGTTGGATCTCAGGTGCAGGCGGGCGGTCCGGCTCGCGCCGGCTCACTGCCCGTTTTCCTGCCCTACC-GTTGTCGGCTCTCTCCCGCGGGTGCTCTTCACTGAGCGTCCCGGGTGGCCGGCGCGTTTACTTTGAAAAAATTAGAGTGTTCAAAGCAGGCCTCGGCTGCCTGAATAATGGTGCATGGAATAATGGAATAGGACCTCGGTTCTATTTTGTTGGTTTTCGGAACTGGAGGTAATGATTAACAGGGACAAACGGGGGCATTCGTATTGCGGCGTTAGAGGTGAAATTCTTGGATCGCCGCAAGACGAGCTACTGCGAAAGCATTTGTCAAGAATGTTTTCATTAATCAAGAACGAAAGTCAGAGGCGCGAAGACGATCAGATACCGTCGTAGTTCTGACCATAAACGATGCCAACTCGCGATCCGCAGGAGTTGCTTAGATGACTCTGCGGGCAGCGTCCGGGAAACCAAAGTTTTTGGGTTCCGGGGGAAGTATGGTTGCAAAGCTGAAACTTAAAGGAATTGACGGAAGGGCACCACCAGGAGTGGAGCCTGCGGCTTAATTTGACTCAACACGGGAAAACTCACCCGGTCCGGACACTGTAAGGATTGACAGATTGATAGCTCTTTCTTGATTCGGTGGGTGGTGGTGCATGGCCGTTCTTAGTTGGTGGAGCGATTTGTCTGGTTAATTCCGATAACGAACGAGACTCTAGCCTACTAAATAGTTCGTCGGTCCCACGATGTGCCGGCGCAACTTCTTAGAGGGACGAGTGGCGTTTAGCCACACGAGATTGAGCAATAACAGGTCTGTGATGCCCTTAGATGTCCGGGGCCGCACGCGCGCTACACTGAAGGAATCAGCGTGGATGCCTCCCTGGCCCGAGAGGGCTGGGAAACCCGTTGAATATCCTTCGTGATTGGGATTGGGGTTTGTAATTATCCCCCATGAACGAGGAATTCCCAGTAAGCGCGAGTCATAAGCTCGCGTTGATTACGTCCCTGCCCTTTGTACACACCGCCCGTCGCTACTATCGATTGAGCGGTTCAGTGAGGGCCTCGGATTGGTCTCGGTCTGGCGTGCAAGCGCCGGCACCGTTGGCCGAGAAGACGCTCGAACTCGATCGCTTGGAGAAAGTAAAAGTCGTAACAAGGTTTCCGTAGGTG

>ZSM20090171_Asperspina_sp

CTGTCTCAAAGATTAAGCCATGCATGTCTAAGTTCACACTGTCTCACGGTGAAACCGCGAATGGCTCATTAAATCAGTCGAGGTTCCTTAGATGACACGATCCTACTTGGATAACTGTGGCAATTCTAGAGCTAATACATGCTTACCAAGCTCCGACCCGT-GGAAAGAGCGCTTTTATTAGTTCAAAACCAATCGT-CGTTGCCCGGGCGCGGCGTCCCCATTGGTGACTCTGGATAACTTTGTGCTGATCGCATGGCCTTTTGTGCCGGCGACGCATCTTTCAAATGTCTGCCCTATCAAATGTCGATGGTACGTGACATGCCTACCATGTTTGTAACGGGTAACGGGGAATCAGGGTTCGATTCCGGAGAGGGAGCATGAGAAACGGCTACCACATCCAAGGAAGGCAGCAGGCGCGCAACTTACCCACTCCCGGCACGGGGAGGTAGTGACGAAAAATAACAATACGGGACTCTTTCGAGGCCCCGTAATTGGAATGAGTACACTTTAAACCCTTTAACGAGGATCTATTGGAGGGCAAGTCTGGTGCCAGCAGCCGCGGTAATTCCAGCTCCAATAGCGTATATTAAAGTTGTTGCAGTTAAAAAGCTCGTAGTTGGATCTCAGGTGCAGGCGGGTGGTCCGGCTCGCGCCGGCTCACTGCCCGTTCTCCTGCCCTACCCGTTGTCGGCTCTCTCCCGCGGGTGCTCTTCGCTGAGCGTCCCGGGTGGCCGGCGCGTTTACTTTGAAAAAATTAGAGTGTTCAAAGCAGGCCTCGGCTGCCTGAATAATGGTGCATGGAATAATGGAATAGGACCTCGGTTCTATTTTGTTGGTTTTCGGAACTGGAGGTAATGATTAACAGGGACAAACGGGGGCATTCGTATTGCGGCGTTAGAGGTGAAATTCTTGGATCGCCGCAAGACGAGCTACTGCGAAAGCATTTGTCAAGAATGTTTTCATTAGTCAAGAACGAAAGTCAGAGGCGCGAAGACGATCAGATACCGTCGTAGTTCTGACCATAAACGATGCCATCCAGCGATCCGCAGGAGTTGCTTCGATGACTCTGCGGGCAGCTTCCGGGAAACCAAAGTTTTTGGGTTCCGGGGGAAGTATGGTTGCAAAGCTGAAACTTAAAGGAATTGACGGAAGGGCACCACCAGGAGTGGAGCCTGCGGCTTAATTTGACTCAACACGGGAAAACTCACCCGGTCCGGACACTGTAAGGATTGACAGATTGATAGCTCTTTCTTGATTCGGTGGGTGGTGGTGCATGGCCGTTCTTAGTTGGTGGAGCGATTTGTCTGGTTAATTCCGATAACGAACGAGACTCTAGCCTATTAAATAGTTCGCCGGTCCCCTGATGCGCCGGCGCAACTTCTTAGAGGGACGAGTGGCGTTTAGCCACACGAGATTGAGCAATAACAGGTCTGTGATGCCCTTAGATGTCCGGGGCCGCACGCGCGCTACACTGAAGGAATCAGCGTGGATGCCTCCCTGGCCCGAAAGGGCTGGGAAACCCGTTGAATCTCCTTCGTGCTAGGGATTGGGGCTTGTAATTGTTCCCCATGAACGAGGAATTCCCAGTAAGCGCGAGTCATAAGCTCGCGTTGATTACGTCCCTGCCCTTTGTACACACCGCCCGTCGCTACTATCGATTGAGCGGTTCAGTGAGGGCCTCGGATTGGTCTCGGTCTGGCGTGCAAGCGCCGGCACCGTTGGCCGAGAAGACGCCCGAACTCGATCGCTTGGAGAAAGTAAAAGTCGTAACAAGGTTNNNNNNNNNN

>ZSM20080393_Pseudunela_marteli

TTGTMTCAAAGATTAAGCCATGCATGTCTAAGTTCACACTGTCTCACGGTGAAACCGCGAATGGCTCATTAAATCAGTCGAGGTTCCTTAGATGACACGATCCTACTTGGATAACTGTGGCAATTCTAGAGCTAATACATGCTTACCAAGCTCCGACCCGC-GGAAAGAGCGCTTTTATTAGTTCAAAACCAATCGT-CGTTGTCCGGGCGCGGCGTCCCTACTGGTGACTCTGGATAACTTTGTGCTGATCGCATGGCCTTTTGCGCCGGCGACGCATCTTTCAAATGTCTGCCCTATCAAATGTCGATGGTACGTGACATGCCTACCATGTTTGTAACGGGTAACGGGGAATCAGGGTTCGATTCCGGAGAGGGAGCATGAGAAACGGCTACCACATCCAAGGAAGGCAGCAGGCGCGCAACTTACCCACTCCCGGCACGGGGAGGTAGTGACGAAAAATAACAATACGGGACTCTTTCGAGGCCCCGTAATTGGAATGAGTACACTTTAAACCCTTTAACGAGGATCTATTGGAGGGCAAGTCTGGTGCCAGCAGCCGCGGTAATTCCAGCTCCAATAGCGTATATTAAAGTTGTTGCAGTTAAAAAGCTCGTAGTTGGATCTCAGGCGCAGGCGGGCGGTCCGGCTCGCGCCGGCTCACTGCCCGTTTTCCTGCCCTACCCGTTGTCGGCTCTCTCCCGCGGGTGCTCTTCACTGAGCGTCCCGGGTGGCCGGCGCGTTTACTTTGAAAAAATTAGAGTGTTCAAAGCAGGCCTC-GCTGCCTGAATAATGGTGCATGGAATAATGGAATAGGACCTCGGTTCTATTTTGTTGGTTTTCGGAACTGGAGGTAATGATTAACAGGGACAAACGGGGGCATTCGTATTGCGGCGTTAGAGGTGAAATTCTTGGATCGCCGCAAGACGAGCTACTGCGAAAGCATTTGTCAAGAATGTTTTCATTAGTCAAGAACGAAAGTCAGAGGCGCGAAGACGATCAGATACCGTCGTAGTTCTGACCATAAACGATGCCAACCCGCGATCCGCAGGAGTTGCTTCGATGACTCTGCGGGCAGCGTCCGGGAAACCAAAGTTTTTGGGTTCCGGGGGAAGTATGGTTGCAAAGCTGAAACTTAAAGGAATTGACGGAAGGGCACCACCAGGAGTGGAGCCTGCGGCTTAATTTGACTCAACACGGGAAAACTCACCCGGTCCGGACACTGTAAGGATTGACAGATTGATAGCTCTTTCTTGATTCGGTGGGTGGTGGTGCATGGCCGTTCTTAGTTGGTGGAGCGATTTGTCTGGTTAATTCCGATAACGAACGAGACTCTAGCCTATTAAATAGTTCGCCGGTCCCACGATGCGCCGGCGCAACTTCTTAGAGGGACGAGTGGCGTTTAGCCACACGAGATTGAGCAATAACAGGTCTGTGATGCCCTTAGATGTCCGGGGCCGCACGCGCGCTACACTGAAGGAATCAGCGTGGATGCCTCCCTGGTCCGAAAGGACTGGGAAACCCGTTGAATCTCCTTCGTGCTAGGGATTGGGGCTTGTAATTATTCCCCATGAACGAGGAATTCCCAGTAAGCGCGAGTCATAAGCTCGCGTTGATTACGTCCCTGCCCTTTGTACACACCGCCCGTCGCTACTATCGATTGAGCGGTTCAGTGAGGGCCTCGGATTGGTCACGGTCTGGTGTGCAAACGTCGGCACCGTTGGCCGAGAAGACGCTCGAACTCGATCGCTTGGAGAAAGTAAAAGTCGTAACAAGGTTTCCGTAGGTG

>ZSM20080063_Acochlidium_fijiense

TTGTCTCAAAGATTAAGCCATGCATGTCTAAGTTCACACTGTCTCACGGTGAAACCGCGAATGGCTCATTAAATCAGTCGAGGTTCCTTAGATGACACGATCCTACTTGGATAACTGTGGCAATTCTAGAGCTAATACATGCTTCCCAAGCTCCGACCCGT-GGAAAGAGCGCTTTTATTAGTTCAAAACCAATCGT-CGTTGTCCGGGCGCGGCGTCCCTACTGGTGACTCTGGATAACTTTGTGCTGATCGCATGGCCTTTTGCGCCGGCGACGCATCTTTCAAATGTCTGCCCTATCAAATGTCGATGGTACGTGACATGCCTACCATGTTTGTAACGGGTAACGGGGAATCAGGGTTCGATTCCGGAGAGGGAGCATGAGAAACGGCTACCACATCCAAGGAAGGCAGCAGGCGCGCAACTTACCCACTCCCGGCACGGGGAGGTAGTGACGAAAAATAACAATACGGGACTCTTTCGAGGCCCCGTAATTGGAATGAGTACACTTTAAACCCTTTAACGAGGATCTATTGGAGGGCAAGTCTGGTGCCAGCAGCCGCGGTAATTCCAGCTCCAATAGCGTATATTAAAGTTGTTGCAGTTAAAAAGCTCGTAGTTGGATCTCAGGCGCAGGCGGGCGGTCCGGCTCGCGCCGGCTCACTGCCCGTTTTCCTGCCCTACTCGTTGTCGGCTCTCTCCCGCGGGTGCTCTTCACTGAGCGTCCCGGGTGGCCGGCGCGTTTACTTTGAAAAAATTAGAGTGTTCAAAGCAGGCCTC-GCTGCCTGAATAATGGTGCATGGAATAATGGAATAGGACCTCGGTTCTATTTTGTTGGTTTTCGGAACTGGAGGTAATGATTAACAGGGACAAACGGGGGCATTCGTATTGCGGCGTTAGAGGTGAAATTCTTGGATCGCCGCAAGACGAGCTACTGCGAAAGCATTTGTCAAGAATGTTTTCATTAGTCAAGAACGAAAGTCAGAGGCGCGAAGACGATCAGATACCGTCGTAGTTCTGACCATAAACGATGCCAACCCGCGATCCGCAGGAGTTGCTTCGATGACTCTGCGGGCAGCGTCCGGGAAACCAAAGTTTTTGGGTTCCGGGGGAAGTATGGTTGCAAAGCTGAAACTTAAAGGAATTGACGGAAGGGCACCACCAGGAGTGGAGCCTGCGGCTTAATTTGACTCAACACGGGAAAACTCACCCGGTCCGGACACTGTAAGGATTGACAGATTGATAGCTCTTTCTTGATTCGGTGGGTGGTGGTGCATGGCCGTTCTTAGTTGGTGGAGCGATTTGTCTGGTTAATTCCGATAACGAACGAGACTCTAGCCTATTAAATAGTTCGCCGGTCCCACGATGCGCCGGCGCAACTTCTTAGAGGGACGAGTGGCGTTTAGCCACACGAGATTGAGCAATAACAGGTCTGTGATGCCCTTAGATGTCCGGGGCCGCACGCGCGCTACACTGAAGGAATCAGCGTGGATGCCTCCCTGGTCCGAAAGGACTGGGAAACCCGTTGAATCTCCTTCGTGCTAGGGATTGGGGCTTGTAATTATTCCCCATGAACGAGGAATTCCCAGTAAGCGCGAGTCATAAGCTCGCGTTGATTACGTCCCTGCCCTTTGTACACACCGCCCGTCGCTACTATCGATTGAGCGGTTCAGTGAGGGCCTCGGATTGGTCACGGCCTGGTGTGCAAATGCCGGCGCCGTCGGCCGAGAAGACGCTCGAACTCGATCGCTTGGAGAAAGTAAAAGTCGTAACAAGGTTTCCGTAGGTG

>BerlinMoll193944_Strubellia_paradoxa

TTGTMTCAAAGATTAAGCCATGCATGTCTAAGTTCACACTGTCTCACGGTGAAACCGCGAATGGCTCATTAAATCAGTCGAGGTTCCTTAGATGACACGATCCTACTTGGATAACTGTGGCAATTCTAGAGCTAATACATGCTTACCAAGCTCCGACCCGT-GGAAAGAGCGCTTTTATTAGTTCAAAACCAATCGT-CGTTGTCCGGGCGCGGCGTCCCTACTGGTGACTCTGGATAACTTTGTGCTGATCGCATGGCCTTTTGCGCCGGCGACGCATCTTTCAAATGTCTGCCCTATCAAATGTCGATGGTACGTGACATGCCTACCATGTTTGTAACGGGTAACGGGGAATCAGGGTTCGATTCCGGAGAGGGAGCATGAGAAACGGCTACCACATCCAAGGAAGGCAGCAGGCGCGCAACTTACCCACTCCCGGCACGGGGAGGTAGTGACGAAAAATAACAATACGGGACTCTTTCGAGGCCCCGTAATTGGAATGAGTACACTTTAAACCCTTTAACGAGGATCTATTGGAGGGCAAGTCTGGTGCCAGCAGCCGCGGTAATTCCAGCTCCAATAGCGTATATTAAAGTTGTTGCAGTTAAAAAGCTCGTAGTTGGATCTCAGGCGCAGGCGGGCGGTCCGGCTCGCGCCGGCTCACTGCCCGTTTTCCTGCCCTACCCGTTGTCGGCTCTCTCCCGCGGGTGCTCTTCACTGAGCGTCCCGGGTGGCCGGCGCGTTTACTTTGAAAAAATTAGAGTGTTCAAAGCAGGCCTC-GCTGCCTGAATAATGGTGCATGGAATAATGGAATAGGACCTCGGTTCTATTTTGTTGGTTTTCGGAACTGGAGGTAATGATTAACAGGGACAAACGGGGGCATTCGTATTGCGGCGTTAGAGGTGAAATTCTTGGATCGCCGCAAGACGAGCTACTGCGAAAGCATTTGTCAAGAATGTTTTCATTAGTCAAGAACGAAAGTCAGAGGCGCGAAGACGATCAGATACCGTCGTAGTTCTGACCATAAACGATGCCAACCCGCGATCCGCAGGAGTTGCTTCGATGACTCTGCGGGCAGCGTCCGGGAAACCAAAGTTTTTGGGTTCCGGGGGAAGTATGGTTGCAAAGCTGAAACTTAAAGGAATTGACGGAAGGGCACCACCAGGAGTGGAGCCTGCGGCTTAATTTGACTCAACACGGGAAAACTCACCCGGTCCGGACACTGTAAGGATTGACAGATTGATAGCTCTTTCTTGATTCGGTGGGTGGTGGTGCATGGCCGTTCTTAGTTGGTGGAGCGATTTGTCTGGTTAATTCCGATAACGAACGAGACTCTAGCCTATTAAATAGTTCGCCGGTCCCACGATGCGCCGGCGCAACTTCTTAGAGGGACGAGTGGCGTTTAGCCACACGAGATTGAGCAATAACAGGTCTGTGATGCCCTTAGATGTCCGGGGCCGCACGCGCGCTACACTGAAGGAATCAGCGTGGATACCTCCCTGGTCCGAAAGGACTGGGAAACCCGTTGAATCTCCTTCGTGCTAGGGATTGGGGCTTGTAATTATTCCCCATGAACGAGGAATTCCCAGTAAGCGCGAGTCATAAGCTCGCGTTGATTACGTCCCTGCCCTTTGTACACACCGCCCGTCGCTACTATCGATTGAGCGGTTCAGTGAGGGCCTCGGATTGGTCACGGCCTGGTGTGCAAACGCCGGCGCCGTCGGCCGAGAAGACGCTCGAACTCGATCGCTTGGAGAAAGTAAAAGTCGTAACAAGGTTTCCGTAGGTG

>ZSM20090244_Hedylopsis_ballantinei

TTSTMTMAAAGATTAAGCCATGCATGTCTAAGTTCACACTGTATCACGGTGAAACCGCGAATGGCTCATTAAATCAGTCGAGGTTCCTTAGATGACACGATCCTACTTGGATAACTGTGGCAATTCTAGAGCTAATACATGCTTACCAAGCTCCGACCCGC-GGAAAGAGCGCTTTTATTAGTTCAAAACCAATCGT-CGTTTCCCGGGGGCGGCGTCCCCACTGGTGACTCTGGATAACTTTGTGCTGATCGCATGGCCTTTTGCGCCGGCGACGCATCTTTCAAATGTCTGCCCTATCAAATGTCGATGGTACGTGACATGCCTACCATGTTTGTAACGGGTAACGGGGAATCAGGGTTCGATTCCGGAGAGGGAGCATGAGAAACGGCTACCACATCCAAGGAAGGCAGCAGGCGCGCAACTTACCCACTCCCGGCACGGGGAGGTAGTGACGAAAAATAACAATACGGGACTCTTTCGAGGCCCCGTAATTGGAATGAGTACACTTTAAACCCTTTAACGAGGATCTATTGGAGGGCAAGTCTGGTGCCAGCAGCCGCGGAAATTCCAGCTCCAATAGCGTATATTAAAGTTGTTGCAGTTAAAAAGCTCGTAGTTGGATCTCAGGCGCAGGCGGGCGGTCCGGCTCGCGCCGGCTCACTGCCCGTTCTCCTGCCCTACCCGTTGTCGGCTCTCTCCCGCGGGTGCTCTTCGCTGAGCGTCCCGGGTGGCCGGCGCGTTTACTTTGAAAAAATTAGAGTGTTCAAAGCAGGCCTC-GCTGCCTGAATAATGGTGCATGGAATAATGGAATAGGACCTCGGTTCTATTTTGTTGGTTTTCGGAACTGGAGGTAATGATTAACAGGGACAAACGGGGGCATTCGTATTGCGGCGTTAGAGGTGAAATTCTTGGATCGCCGCAAGACGAGCTACTGCGAAAGCATTTGTCAAGAATGTTTTCATTAGTCAAGAACGAAAGTCAGAGGCGCGAAGACGATCAGATACCGTCGTAGTTCTGACCATAAACGATGCCAACTCGCGATCCGCAGGAGTTGCTTCGATGACTCTGCGGGCAGCGTCCGGGAAACCMAAGTTTTTGGGTTCCGGGGGAAGTATGGTTGCAAAGCTGAAACTTAAAGGAATTGACGGAAGGGCACCACCAGGAGTGGAGCCTGCGGCTTAATTTGACTCAACACGGGAAAACTCACCCGGTCCGGACACTGTAAGGATTGACAGATTGATAGCTCTTTCTTGATTCGGTGGGTGGTGGTGCATGGCCGTTCTTAGTTGGTGGAGCGATTTGTCTGGTTAATTCCGATAACGAACGAGACTCTAGCCTATTAAATAGTTCGCCGGTCCCACGATGCGCCGGCGCAACTTCTTAGAGGGACGAGTGGCGTTTAGCCACACGAGATTGAGCAATAACAGGTCTGTGATGCCCTTAGATGTCCGGGGCCGCACGCGCGCTACACTGAAGGAATCAGCGTGGATGCCTCCCTGGCCCGAAAGGGCTGGGAAACCCGTTGAATCTCCTTCGTGCTAGGGATTGGGGCTTGTAATTCTTCCCCATGAACGAGGAATTCCCAGTAAGCGCGAGTCATAAGCTCGCGTTGATTACGTCCCTGCCCTTTGTACACACCGCCCGTCGCTACTATCGATTGAGCGGTTCAGTGAGGGCCTCGGATTGGTCTCGGTCTGGCGTGCAAGCGCCGGCACCGTGGGCCGAGAAGACGCTCGAACTCGATCGCTTGGAGAAAGTAAAAGTCGTAACAAGGTTTCCGTAGGTG

>ZSM20081019_Microhedyle_glandulifera

TTSTMTCAAWGATTAAGCCATGCATGTCTAAGTTCACACTATCTCACGGTGAAACCGCGAATGGCTCATTAAATCAGTCGAGGTTCCTTAGATGACACGATCCTACTTGGATAACTGTGGCAATTCTAGAGCTAATACATGCTTACCAAGCTCCGACCCGT-GGAAAGAGCGCTTTTATTAGTTCAAAACCAATCGC-CGTTACCCGGGTGCGGCGTCCCCACTGGTGACTCTGGATAACTTTGTGCTGATCGCATGGCCTTTTGCGCCGGCGACGCATCTTTCAAATGTCTGCCCTATCAAATGTCGATGGTACGTGACATGCCTACCATGTTTGTAACGGGTAACGGGGAATCAGGGTTCGATTCCGGAGAGGGAGCATGAGAAACGGCTACCACATCCAAGGAAGGCAGCAGGCGCGCAACTTACCCACTCCCGGCACGGGGAGGTAGTGACGAAAAATAACAATACGGGACTCTTTCGAGGCCCCGTAATTGGAATGAGTACACTTTAAACCCTTTAACGAGGATCTATTGGAGGGCAAGTCTGGTGCCAGCAGCCGCGGTAATTCCAGCTCCAATAGCGTATATTAAAGTTGTTGCAGTTAAAAAGCTCGTAGTTGGATCTCAGGCGCAGGCGGGTGGTCCGGCTCGCGCCGGCTCACTGCCCGTACTCCTGCCCTACCCGTTGTCGGCTCTCTCCCGTGGGTGCTCTTCACTGAGCGTCCCGGGTGGCCGGCGCGTTTACTTTGAAAAAATTAGAGTGTTCAAAGCAGGCCTCAGCTGCCTGAATAATGGTGCATGGAATAATGGAATAGGACCTCGGTTCTATTTTGTTGGTTTTCGGAACTGGAGGTAATGATTAACAGGGACAAACGGGGGCATTCGTATTGCGGCGTTAGAGGTGAAATTCTTGGATCGCCGCAAGACGAGCTACTGCGAAAGCATTTGTCAAGAATGTTTTCATTAATCAAGAACGAAAGTCAGAGGCGCGAAGACGATCAGATACCGTCGTAGTTCTGACCATAAACGATGCCACCTAGCGATCCGCAGGAGTTGCTTCGATGACTCTGCGGGCAGCTTCCGGGAAACCAAAGTTTTTGGGTTCCGGGGGAAGTATGGTTGCAAAGCTGAAACTTAAAGGAATTGACGGAAGGGCACCACCAGGAGTGGAGCCTGCGGCTTAATTTGACTCAACACGGGAAAACTCACCCGGTCCGGACACTGTAAGGATTGACAGATTGATAGCTCTTTCTTGATTCGGTGGGTGGTGGTGCATGGCCGTTCTTAGTTGGTGGAGCGATTTGTCTGGTTAATTCCGATAACGAACGAGACTCTAGCCTATTAAATAGTTCGCCGGTCCCTTGATGCGCCGGCGCAACTTCTTAGAGGGACGAGTGGCGTTTAGCCACACGAGATTGAGCAATAACAGGTCTGTGATGCCCTTAGATGTCCGGGGCCGCACGCGCGCTACACTGAAGGAATCAGCGTGGATGCTTCCCTGGCCCGAGAGGGCTGGGAAACCCGTTGAATCTCCTTCGTGCTAGGGATTGGGGCTTGTAATTCTTCCCCATGAACGAGGAATTCCCAGTAAGCGCGAGTCATAAGCTCGCGTTGATTACGTCCCTGCCCTTTGTACACACCGCCCGTCGCTACTATCGATTGAGCGGTTCAGTGAGGGCCTCGGATTGGTCTCGGTCTGGCGTGCAAGCGCCGGCACCGTTGGCCGAGAAGAAGCTCGAACTCGATCGCTTGGAGAAAGTAAAAGTCGTAACAAGGTTTCCGTAGGTG

>ZSM2010328_Ganitus_evelinae

NNNNNNNNNNNNNNNNNNNNNNNNNNNNNNNNNNNNNNNNNNNNNNNNNNNNNNNNNNNNNNNNNNNNNNNNNNNNNNNNNNNNNNNNNNNNNNNNNNNNNNNNNNNNNNNNNNNNNNNNNNNNNNNNNNNNNNNNNNNNNNNNNNNNNNNNNNNNNNNNNNNNNNNNNNNNNNNNNNNNNNNNNNNNNNNNNNNNNNNNNNNNNNNNNNNNNNNNNNNNNNNNNNNNNNNNNNNNNNNNNNNNNNNNNNNNNNNNNNNNNNNNNNNNNNNNNNNNNNNNNNNNNNNNNNNNNNNNNNNNNNNNNNNNNNNNNNNNNNNNNNNNNNNNNNNNNNNNNNNNNNNNNNNNNNNNNNNNNNNNNNNNNNNNNNNNNNNNNNNNNNNNNNNNNNNNNNNNNNNNNNNNNNNNNNNNNNNNNNNNNNNNNNNNNNNNNNNNNNNNNNNNNNNNNNNNNNNNNNNNNNNNNNNNNCGGGACTCTTTCGAGGCCCCGTAATTGGAATGAGTACACTTTAAACCCTTTAACGAGGATCTATTGGAGGGCAAGTCTGGTGCCAGCAGCCGCGGTAATTCCAGCTCCAATAGCGTATATTAAAGTTGTTGCAGTTAAAAAGCTCGTAGTTGGATCTCAGGCGCAGGCGGGTGGTCCGGCTCGCGCCGGCTCACTGCCCGTACTCCTGCCCTACCCGTTGTCGGCTCTCTCCCGTGGGTGCTCTTCACTGAGCGTCCCGGGTGGCCGGCGCGTTTACTTTGAAAAAATTAGAGTGTTCAAAGCAGGCCTCGGCTGCCTGAATAATGGTGCATGGAATAATGGAATAGGACCTCGGTTCTATTTTGTTGGTTTTCGGAACTGGAGGTAATGATTAACAGGGACAAACGGGGGCATTCGTATTGCGGCGTTAGAGGTGAAATTCTTGGATCGCCGCAAGACGAGCTACTGCGAAAGCATTTGTCAAGAATGTTTTCATTAATCAAGAACGAAAGTCAGAGGCGCGAAGACGATCAGATACCGTCGTAGTTCTGACCATAAACGATGCCACCTAGCGATCCGCAGGAGTTGCTTCGATGACTCTGCGGGCAGCTTCCGGGAAACCAAAGTTTTTGGGTTCCGGGGGAAGTATGGTTGCAAAGCTGAAACTTAAAGGAATTGACGGAAGGGCACCACCAGGAGTGGAGCCTGCGGCTTAATTTGACTCAACACGGGAAAACTCACCCGGTCCGGACACTGTAAGGATTGACAGATTGATAGCTCTTTCTTGATTCGGTGGGTGGTGGTGCATGGCCGTTCTTAGTTGGTGGAGCGATTTGTCTGGTTAATTCCGATAACGAACGAGACTCTAGCCTATTAAATAGTTCGCCGGTCCCTTGATGCGCCGGCGCAACTTCTTAGAGGGACGAGTGGCGTTTAGCCACACGAGATTGAGCAATAACAGGTCTGTGATGCCCTTAGATGTCCGGGGCCGCACGCGCGCTACACTGAAGGAATCAGCGTGGATGCTTCCCTGGCCCGAAAGGGCTGGGAAACCCGTTGAATCTCCTTCGTGCTAGGGATTGGGGCTTGTAATTCTTCCCCATGAACGAGGAATTCCCAGTAAGCGCGAGTCATAAGCTCGCGTTGATTACGTCCCTGCCCTTTGTACACACCGCCCGTCGCTACTATCGATTGAGCGGTTCAGTGAGGGCCTCGGATTGGTCTCGGTCTGGCGTGCAAGCGCCGGCACCGTTGGCCGAGAAGAAGCTCGAACTCGATCGCTTGGAGAAAGTAAAAGTCGTAACAAGGTTTCCGTAGGTG

>ZSM20080170_Paraganitus_ellynae

TTGTCTCAAAGATTAAGCCATGCATGTCTAAGTTCACACTATCTCACGGTGAAACCGCGAATGGCTCATTAAATCAGTCGAGGTTCCTTAGATGACACGATCCTACTTGGATAACTGTGGCAATTCTAGAGCTAATACATGCTTATCAAGCTCCGACCCGT-GGAAAGAGCGCTTTTATTAGTTCAAAACCAATCGC-CGTTACCCGGGTGCGGCGTCCCCACTGGTGACTCTGGATAACTTTGTGCTGATCGCATGGCCTTTTGCGCCGGCGACGCATCTTTCAAATGTCTGCCCTATCAAATGTCGATGGTACGTGACATGCCTACCATGTTTGTAACGGGTAACGGGGAATCAGGGTTCGATTCCGGAGAGGGAGCATGAGAAACGGCTACCACATCCAAGGAAGGCAGCAGGCGCGCAACTTACCCACTCCCGGCACGGGGAGGTAGTGACGAAAAATAACAATACGGGACTCTTTCGAGGCCCCGTAATTGGAATGAGTACACTTTAAACCCTTTAACGAGGATCTATTGGAGGGCAAGTCTGGTGCCAGCAGCCGCGGTAATTCCAGCTCCAATAGCGTATATTAAAGTTGTTGCAGTTAAAAAGCTCGTAGTTGGATCTCAGGCGCAGGCGGGTGGTCCGGCTCGCGCCGGCTCACTGCCCGTACTCCTGCCCTACCCGTTGTCGGCTCTCTCCCGTGGGTGCTCTTCACTGAGCGTCCCGGGTGGCCGGCGCGTTTACTTTGAAAAAATTAGAGTGTTCAAAGCAGGCCTCGGCTGCCTGAATAATGGTGCATGGAATAATGGAATAGGACCTCGGTTCTATTTTGTTGGTTTTCGGAACTGGAGGTAATGATTAACAGGGACAAACGGGGGCATTCGTATTGCGGCGTTAGAGGTGAAATTCTTGGATCGCCGCAAGACGAGCTACTGCGAAAGCATTTGTCAAGAATGTTTTCATTAATCAAGAACGAAAGTCAGAGGCGCGAAGACGATCAGATACCGTCGTAGTTCTGACCATAAACGATGCCACCTAGCGATCCGCAGGAGTTGCTTCGATGACTCTGCGGGCAGCTTCCGGGAAACCAAAGTTTTTGGGTTCCGGGGGAAGTATGGTTGCAAAGCTGAAACTTAAAGGAATTGACGGAAGGGCACCACCAGGAGTGGAGCCTGCGGCTTAATTTGACTCAACACGGGAAAACTCACCCGGTCCGGACACTGTAAGGATTGACAGATTGATAGCTCTTTCTTGATTCGGTGGGTGGTGGTGCATGGCCGTTCTTAGTTGGTGGAGCGATTTGTCTGGTTAATTCCGATAACGAACGAGACTCTAGCCTATTAAATAGTTCGCCGGTCCCTTGATGCGCCGGCGCAACTTCTTAGAGGGACGAGTGGCGTTTAGCCACACGAGATTGAGCAATAACAGGTCTGTGATGCCCTTAGATGTCCGGGGCCGCACGCGCGCTACACTGAAGGAATCAGCGTGGATGCTTCCCTGGCCCGAAAGGGCTGGGAAACCCGTTGAATCTCCTTCGTGCTAGGGATTGGGGCTTGTAATTATTCCCCATGAACGAGGAATTCCCAGTAAGCGCGAGTCATAAGCTCGCGTTGATTACGTCCCTGCCCTTTGTACACACCGCCCGTCGCTACTATCGATTGAGCGGTTCAGTGAGGGCCTCGGATTGGTCTCGGTCTGGCGTGCAAGCGCCGGCACCGTTGGCCGAGAAGAAGCTCGAACTCGATCGCTTGGAGAAAGTAAAAGTCGTAACAAGGTTTCCGTAGGTG
