# Supplementary material for: How to describe a cryptic species? Practical challenges of molecular taxonomy
Source: Front Zool. 2013 Sep 27;10:59. doi: 10.1186/1742-9994-10-59 (PMC4015967; doi:10.1186/1742-9994-10-59)
Supplement: Additional file 2 — 28S rRNA alignment of Pontohedyle with outgroups to determine diagnostic nucleotides for the genus (fasta format). The alignment was generated with MUSCLE [107] and ambiguous parts of the alignment were masked with Gblocks [108] (settings for a less stringent selection). [file 1742-9994-10-59-S2.docx]

### Additional file 2 – 28S rRNA alignment of *Pontohedyle* with outgroups to determine diagnostic nucleotides for the genus (fasta format)

The alignment was generated with Muscle [[96](#_ENREF_96)] and ambiguous parts of the alignment were masked with Gblocks [[97](#_ENREF_97)] (settings for a less stringent selection).

>ZSM20080565

AGGATTCCCCCAGTAACGGCGAGTGAAGCGGGAAGAGCCCAGCACCGAATCCCTCAGTGTCATGCTGACGGGAACTGTGGTGTGTGGGACGCCACCAGTCGCACATGCGAGCGCCGAAGTCCTCCTGATCGAGGCTTCACCCAGAGCGGGTGTAAGGCCAGTGCTGGTGCTTCGCTGTGCGGCCGCGAGCGTCTCAGGAGTCGGGTTGTTTGGGAATGCAGCCCAAAGCGGGTGGTAAACTCCATCTAAGGCTAAATACCGGCACGAGTCCGATAGCGGACAAGTACCGTGAGGGAAAGTTGAAAAGAACTTTGAAGAGAGAGTTCAAGAGTACGTGAAACCGCCCAGAGGTAAACGGGTGCATCCGCAAAGTCGGCCCGTGGAATTCAGCGCGGCGTTCGGTCTGGAA-TGGTTCGTTTCGGGATCCCTGGGACTCGGGCGGG--CCAGAACCGGACCTCGCCGCGTGCACTTTCCGCGGGCAGAGCGCCACGACCGGTTTCGCGGCGGCGACGAGCCGGGCGGGAAGGTAGGCGTCTCCTCCGGGCGCGCTGTTATAGACCGCTCTGGTGAGCTGCTGCGGGACCGAGGGACGGCCGCGTTCTTCGAGGCCAGGCTTTCTCGGGGAGTTCGACTGGCAGAGACTGGGCAACCGTGTCTGCCGACCGCTTTCCGGGACCGGACCGGGCTGGCCGGGAGCGCTGAGGGTCGGTGGCGAATCTGTCGGCATTGCACCCGACCCGTCTTGAAACACGNNNNNNNNNNNNNNNNNNNNNNNNNNNNNNNNNNNNNNNNNNNNNNNNNNNNNNNNNNNNNNNNNNNNNNNNNNNNNNNNNNNNNNNNNNNNNNNNNNNNNNNNNNNNNNNNNNNNNNNNNNNNNNNNNNNNNNNNNNNNNNNNNNNNNNNNNNNNNNNNNNNNNNNNNNNNNNNNNNNNNNNNNNNNNNNNNNNNNNNNNNNNNNNNNNNNNNNNNNNNNNNNNNNNN

>ZSM20080176

AGGATTCCCCCAGTAACGGCGAGTGAAGCGGGAAGAGCCCAGCACCGAATCCCTCAGTGTCATGCTGACGGGAACTGTGGTGTGTGGGACGCCACCAGTCGCATTAAAGGGCGCCGAAGTCCTCCTGATCGAGGCTTCACCCAGAGCGGGTGTAAGGCCAGTGCTGGTGCCTCTTTGTGCGGCCGCGAGCGTCTCAGGAGTCGGGTTGTTTGGGAATGCAGCCCAAAGCGGGTGGTAAACTCCATCTAAGGCTAAATACCGGCACGAGTCCGATAGCGGACAAGTACCGTGAGGGAAAGTTGAAAAGAACTTTGAAGAGAGAGTTCAAGAGTACGTGAAACCGCCCAGAGGTAAACGGGTGCATCCGCAAAGTCGGCCCGTGGAATTCAGCGCGGCGCGCGGCCTGGGGCTGCTTCGTTCCGGGATCCCTGGGACCCGGGCGGGGTGCTGCGCCGGGCTCCGCCGCGTGCACTTTCTGCGGGCAGAGCGCCACGACCGGTTTCGCGGCGGCGACGAGCCGGGCGGGAAGGTAGGCG-------AGGGCGCGCTGTTACAGCCCGCCCTGGTGAGCTGCTGCGGGACCGAGGGACGGCCGTGCTCTTGGAGGCCAGGCCTTCTCGGGGAGTTCGACTGGTAGAGACTGGGCAACCGTGTCTGCCGACCGCTTCTCGAGACCGGATCGGGCTGGCCGGGAGCGCTGAGGGTCGGTGGCGAATCTGTCGGCATTGCACCCGACCCGTCTTGNNNNNNNNNNNNNNNNNNNNNNNNNNNNNNNNNNNNNNNNNNNNNNNNNNNNNNNNNNNNNNNNNNNNNNNNNNNNNNNNNNNNNNNNNNNNNNNNNNNNNNNNNNNNNNNNNNNNNNNNNNNNNNNNNNNNNNNNNNNNNNNNNNNNNNNNNNNNNNNNNNNNNNNNNNNNNNNNNNNNNNNNNNNNNNNNNNNNNNNNNNNNNNNNNNNNNNNNNNNNNNNNNNNNNNNNNNNN

>ZSM20071135

NGGATTCCCCCAGTAACGGCGAGTGAAGCGGGAAGAGCCCAGCACCGAATCCCTCAGTGTCATGCTGGCGGGAACTGTGGTGTGTGGGACGCCACCAGTCGCATTAAAGGGCGCCGAAGTCCTCCTGATCGAGGCTTCACCCAGAGCGGGTGTAAGGCCAGTGCTGGTGCCTCTTTGTGCGGCCGCGAGCGTCTCAGGAGTCGGGTTGTTTGGGAATGCAGCCCAAAGCGGGTGGTAAACTCCATCTAAGGCTAAATACCGGCACGAGTCCGATAGCGGACAAGTACCGTGAGGGAAAGTTGAAAAGAACTTTGAAGAGAGAGTTCAAGAGTACGTGAAACCGCCCAGAGGTAAACGGGTGCATCCGCAAAGTCGGCCCGTGGAATTCAGCGCGGCGCGCGGCCTGGGGCTGCTTCGTTCCGGGATCCCTGGGACCCGGGCGGGGTGCTGCGCCGGGCTCCGCCGCGTGCACTTTCTGCGGGCAGAGCGCCACGACCGGTTTCGCGGCGGCGACGAGCCGGGCGGGAAGGTAGGCG-------AGGGCGCGCTGTTACAGCCCGCCCTGGTGAGCTGCTGCGGGACCGAGGGACGGCCGTGCTCTTGGAGGCCAGGCCTTCTCGGGGAGTTCGACTGGTAGAGACTGGGCAACCGTGTCTGCCGACCGCTTCTCGAGACCGGATCGGGCTGGCCGGGAGCGCTGAGGGTCGGTGGCGAATCTGTCGGCATTGCACCCGACCCGTCTTGAAACACGGACCAAGGAGTCTAACATGCGCGCGAGTCATTGGGTCGTACGAAACTCAAAGGCGCAGTGAAAGCGAGGGTCGCCCCGGGCTGACCCAGGTGGGATCTCTCCGCTCC----GGCGGGGAGCGCACCACCGGCCCGTCCCGTCCGCGTTGTCGGTNNNNNNNNNNNNNNNNNNNNNNNNNNNNNNNNNNNNNNNNNNNNNNNNNNNNNNNNNNNNNNNNNNNNNNNNNNN

>SICBC2010KJ02E01

NNNNNNNNNNNNNNNNNNNNNNNNNNNNNNNNNNNNNNNNNNNNNNNNNNNNNNNNNNNNNNNNNNNNNNNNNNNNNNNNNNNNNNNNNNNNNNNNNNNNNNNNNNNNNNNNNCCGAAGTCCTCCTGATCGAGGCTTCACCCAGAGCGGGTGTAAGGCCAGTGCTGGTGCTTCGCTGTGCGGCCGCGAGCGTCTCAGGAGTCGGGTTGTTTGGGAATGCAGCCCAAAGCGGGTGGTAAACTCCATCTAAGGCTAAATACCGGCACGAGTCCGATAGCGGACAAGTACCGTGAGGGAAAGTTGAAAAGAACTTTGAAGAGAGAGTTCAAGAGTACGTGAAACCGCCCAGAGGTAAACGGGTGCATCCGCAAAGTCGGCCCGTGGAATTCAGCGCGGCGCGCGGCCTGGAGCTGCTTCGTTTCGGGATCCC-GGGACCCGGGCGGGGTGCTGCGCCGGGCCCCGCCGCGTGCACTTTCTGCGGGCAGAGCGCCACGACCGGTTTCGCGGCGGCGACGAGCCGGGCGGGAAGGTAGGCGTCTCCTTCGGGCGCGCTGTTATAGACCGCCTCGGTGAGCTGCTGCGGGACCGAGGGACGGCCGCGTTCTTCGAGGCCAGGCTTTCTCGGGGAGTTCGACTGGCAGAGACTGAGCAATCGTGTCTGCCGACCGCTTCTCGA-GTCGGTCCGGGCTGGCCGGGAGCGCTCAGGGTCGGTGGCGAATCTGTCGGCATTGCACCCGACCCGTCTTGAAACACGGACCAAGGAGTCTAACATGCGCGCGAGTCATTGGGTTTTTTGAAATCCAAAGGCGCAGTGAAAGCGAGGGTCGCCCCGGGCTGACCCAGGTGGGATCTTTCCGTCTCTNNNNNNNNNNNNNNNNNNNNNNNNNNNNNNNNNNNNNNNNNNNNNNNNNNNNNNNNNNNNNNNNNNNNNNNNNNNNNNNNNNNNNNNNNNNNNNNNNNNNNNNNNNNNNNNNNNNNNNNNN

>SICBC2010KJ01E03

NNNATTCCCCCAGTAACGGCGAGTGAAGCGGGAAGAGCCCAGCACCGAATCCCTCAGTGTCATGCTGGCGGGAACTGTGGTGTGTGGGACGCCACCAGTCG-ACATGCGGGCGCCGAAGTCCTCCTGATCGAGGCTTCACCCAGAGCGGGTGTAAGGCCAGTGCTGGTGCTTCGCTGTGCGGCCGCGAGCGTCTCAGGAGTCGGGTTGTTTGGGAATGCAGCCCAAAGCGGGTGGTAAACTCCATCTAAGGCTAAATACCGGCACGAGTCCGATAGCGGACAAGTACCGTGAGGGAAAGTTGAAAAGAACTTTGAAGAGAGAGTTCAAGAGTACGTGAAACCGCCCAGAGGTAAACGGGTGCATCCGCAAAGTCGGCCCGTGGAATTCAGCGCGGCGCGCGGCCTGGAGCTGCTTCGTTTCGGGATCCCTGGGACCCGGGCGGGGTGCTGCGCCGGGCCCCGCCGCGTGCACTTTCTGCGGGCAGAGCGCCACGACCGGTTTCGCGGCGGCGACGAGCCGGGCGGGAAGGTAGGCGTCTCCTTCGGGCGCGCTGTTATAGACCGCCTCGGTGAGCTGCTGCGGGACCGAGGGACGGCCGCGTTCTTCGAGGCCAGGCTTTCTCGGGGAGTTCGACTGGCAGAGACTGAGCAATCGTGTCTGCCGACCGCTTCTCGA-GTCGGTCCGGGCTGGCCGGGAGCGCTCAGGGTCGGTGGCGAATCTGTCGGCATTGCACCCGACCCGTCTTGAAACACGGACCAAGGAGTCTAACATGCGCGCGAGTCATTGGGTTCTACGAAATCCAAAGGCGCAGTGAAAGCGAGGGTCGCCCCGGGCTGACCCAGGTGGGATCTTTCCGTCTCTCGGAGCGGGGAGCGCACCACCGGCCCGTCCCGTCCGNNNNNNNNNNNNNNNNNNNNNNNNNNNNNNNNNNNNNNNNNNNNNNNNNNNNNNNNNNNNNNNNNNNNNNNNNNNNNNNNNNNNN

>ZSM20090244_Hedylopsis_ballantinei

NGGATTTCCCCAGTAACGGCGAGTGAAGCGGGAAGAGCCCAGCACCGAATCCCCCAGTGTCATGCTGGCGGGAACTGTGGTGTGAGGGACGCCACCAGTCGCACCAGCGGGCGCCGAAGTCCTCCTGATCGAGGCTTCACCCAGAGCGGGTGTAAGGCCTTTGC-GGTGCTTCGCTGTGCGGCCGCGAGCGTCTCAGGAGTCGGGTTGTTTGGGAATGCAGCCCAAAGCGGGTGGTAAACTCCATCTAAGGCTAAATACTGGCACGAGTCCGATAGCGGACAAGTACCGTGAGGGAAAGTTGAAAAGAACTTTGAAGAGAGAGTTCAAGAGTACGTGAAACCGCCCAGAGGTAAACGGGTGGATCCGCAAAGTCGGCCCGCGGAATTCAGCGCGGCGCGCGGCTCGGAGCTGCTTCGGTTTGGGATCCTTCGGACTCATCCGGGGTGCTGCGCCGGGCTCCGCCGCGTGCACTTTCTGCGGGCAGAGCGCCACGACCGATTTCGTGGCGGCCACAAGCCAGGTGGGAAGGTAGGCG-CCGCCTCGTGCGCGCTGTTACAGCCCGTCATGGTGAGCCGCCTGGGGATCGAGGAACGGCCGCGCGCTTCGAGGCCTGGCCCTCCGGGGGAGTTCGACTGGCAGAGACTGGGCAACCGTGTCTGCCGACCGCTCCCTCGGATTGGATCGGGAAGGCCGGGCGTGCTTAGGGTCAGTGGCGAGTCTGTCGGCATTCCACCCGACCNNNNNNNNNNNNNNNNNNNNNNNNNNNNNNNNNNNNNNNNNNNNNNNNNNNNNNNNNNNNNNNNNNNNNNNNNNNNNNNNNNNNNNNNNNNNNNNNNNNNNNNNNNNNNNNNNNNNNNNNNNNNNNNNNNNNNNNNNNNNNNNNNNNNNNNNNNNNNNNNNNNNNNNNNNNNNNNNNNNNNNNNNNNNNNNNNNNNNNNNNNNNNNNNNNNNNNNNNNNNNNNNNNNNNNNNNNNNNNNNNNN

>ZSM20090171_Asperspina_sp

NGGATTTCCCCAGTAACGGCGAGTGAAGCGGGAAAAGCCCAGCACCGAATCCCTCAGTGTCATGCTGACGGGAACTGTGGTGTGCGGGACGCCACCAGTCGCACCAGTGGGCACCGAAGTCCTCCTGATCGAGGCTTCACCCAGAGCGGGTGTAAGGCCTTTGCAGGTGCTTCGCTGTGCGGCCGCGAGCGTCTCAGGAGTCGGGTTGTTTGGGAATGCAGCCCTAAGTGGGTGGTAAACTCCATCTAAGGCTAAATACTGGCACGAGTCCGATAGCGGACAAGTACCGTGAGGGAAAGTTGAAAAGAACTTTGAAGAGAGAGTTCAAGAGTACGTGAAACCGCTTAGAGGTAAACGGGTGGATCCGCAAAGTCGGCCCGTGGAATTCAGCGCGGCGCGCGGTCTGGGGCTGCTTCGATTCGGGATCCCTGGGACCCGGTCGAGGTGCCGCGCCGGACCCCGCCGCGTGCACTTTCCGCGGGCAGAGCGCCACGACCGGTTTCGCTGCGGCCATGAGCCGGGTGGGAAGGTAGGCG-------AGAGCGTGCTGTTACAGACCACCCCGGTGGGTCGCCGCGGGACCGAGGGACTGCCGTGCTCTTCGTGGCCAGGCCTTCGTTGGGAGTTCGACCTGCAGAGACTGGGCAACCGTGTCTGCTAACCGCTCCCTCTGACTGGACTGGGCTGGCCGGGAGCGCCAAGGGTCTGTGGCGAATCTGTCGGCATTCCACCCGACCCGTCTNNNNNNNNNNNNNNNNNNNNNNNNNNNNNNNNNNNNNNNNNNNNNNNNNNNNNNNNNNNNNNNNNNNNNNNNNNNNNNNNNNNNNNNNNNNNNNNNNNNNNNNNNNNNNNNNNNNNNNNNNNNNNNNNNNNNNNNNNNNNNNNNNNNNNNNNNNNNNNNNNNNNNNNNNNNNNNNNNNNNNNNNNNNNNNNNNNNNNNNNNNNNNNNNNNNNNNNNNNNNNNNNNNNNNNNNNNNNNN

>ZSM20100584_Parhedyle_cryptophthalma

AGGATTTCCCCAGTAACGGCGAGTGAAGCGGGAAGAGCCCAGCACCGAATCCCTCAGTGTCATGCTGACGGGAACTGTGGTGTGTGGGACGCCACCAGTCGCACCAGCGGGCGCCGAAGTCCTCCTGATCGAGGCTTCACCCAGAGCGGGTGTAAGGCCAGTGCAGGTGCTTCGCTGTGCGGCCGCGAGCGTCTCAGGAGTCGGGTTGTTTGGGAATGCAGCCCTAAGCGGGTGGTAAACTCCATCTAAGGCTAAATACTGGCACGAGTCCGATAGCGGACAAGTACCGTGAGGGAAAGTTGAAAAGAACTTTGAAGAGAGAGTTCAAGAGTACGTGAAACCGCCCAGAGGTAAACGGGTGCATCCGCAAAGTCGGCCCGCGGAATTCAGCGCGGCGCGCGGCCTGGGGCTGCCTCGTCTCGGGATCCCTGGGACCCGGGCGGGGTGCCGCGCCGGGCTCCGCCGCGTGCACTTTCCGCGGGCAGAGCGCCACGACCGGTTTCGCGGCGGTGACAAGCCGGGCAGGAAGGTAGGCG-------GGGGCGCGCTGTTACAG-CCTCCTTGGTGAGCCGCTGCGGGACCGAGGGACGGCCGCGCTCTTCGTGGCCAGGCCTTCCTGGGGAGTTCGACTGACAGAGACTGGGCAACCGTGTCTGTCGACCGCTTCTCTGGACTGGATCGGGCTGGCCGGGAGCGCTGAGGGTCGGTGGCGAGTCTGTCGGCATTGCACCCGACCCGTCTTGAAACACGGACCAAGGAGTCTAACATGCGCGCGAGTCGTTGGGTTGTACGAAACCCAAAGGCGCAGTGAAAGCGAGGGTCGCCCCGGGCTGACCCAGGTGGGATCTCTCTGGCCCTCGGGCCGGGGAGCGCACCACCGGCCCGTCCCGTCTGCGCTGTCAGTGGGGCGGAGCAGGAGCGTGCACGCTGGGACCCGAAAGATGGTGAACTATGCCTGAGTAGAACGAAGCCAGAGNNN

>ZSM20080170_Paraganitus_ellynae

NNNNTTTCCCCAGTAACGGCGAGTGAAGCGGGAAGAGCCCAGCACCGAATCCCTCAGTGTCATGCTGACGGGAACTGTGGTGTGTGGGACGCCACCAGTCGCACTAGCGGGCGCCGAAGTCCTCCTGATCGAGGCTTCACCCAGAGCGGGTGTAAGGCCAGTGCAGGTGCCTCGCTATGCGGCCGCGAGCGTCTCAGGAGTCGGGTTGTTTGGGAATGCAGCCCAAAGCGGGTGGTAAACTCCATCTAAGGCTAAATACTGGCACGAGTCCGATAGCGGACAAGTACCGTGAGGGAAAGTTGAAAAGAACTTTGAAGAGAGAGTTCAAGAGTACGTGAAACCGCCCAGAGGTAAACGGGTGCATCCGCAAAGTCGGCCCGCGGAATTCAGCGCGGCGCGCGGCCTGGGGCTGCCTCGTCTCGGGATCCCTGGGACCCGGGCGGGGTGTCGCGCCGGGCTCCGCCGCGTGCACTTTCCGCGGGCAGAGCGCCACGACCGGTTTCTCGGCGGTGACGAGCCGGGTAGGAAGGTAGGCG-------AGGGCGCGCTGTTACAGCCTGCCCTGGTGAGCCGCTGGGGGACCGAGGGACGGCCGCGTTCTTCGTGGCCAGGCCTTCCGCGGGAGTTCGACTGGCAGAGACTGGGCAACCGTGTCTGCCGACCGCTCTTGTGGACTGGATCGGGCTGGCCGGGAGCGCTGAGGGTCGGTGGCGAGTCTGTCGGCATTGCACCCGACCCGTCTTGAAACACGGACCAAGGAGTCTAACATGCGCGCGAGTCGTTGGGTTGTACGAAACCCAAAGGCGCAGTGAAAGCGAGGGTCGCCCCGGGCTGACCCAGGTGGGATCCTTCTGGCCCTCGGGCCGGGGGGCGCACCACCGGCCCGTCCCGTCTGCGTTGTCAGTGGGGCGGAGCAGGAGCGTGCACGCTGGGACCCGAAAGATGGTGAACTATGCCTGAGTAGAACGAAGCCAGNNNNN

>ZSM20100328_Ganitus_evelinae

AGGATTTCCCCAGTAACGGCGAGTGAAGCGGGAAGAGCCCAGCACCGAATCCCTCAGTGTCATGCTGACGGGAACTGTGGTGTGTGGGACGCCACCAGTCGCACTAGCGGGCGCCGAAGTCCTCCTGATCGAGGCTTCACCCAGAGCGGGTGTAAGGCCAGTGCAGGTGCTTCGCTATGCGGCCGCGAGCGTCTCAGGAGTCGGGTTGTTTGGGAATGCAGCCCAAAGCGGGTGGTAAACTCCATCTAAGGCTAAATACTGGCACGAGTCCGATAGCGGACAAGTACCGTGAGGGAAAGTTGAAAAGAACTTTGAAGAGAGAGTTCAAGAGTACGTGAAACCGCCCAGAGGTAAACGGGTGCATCCGCAAAGTCGGCCCGCGGAATTCAACGCGGCGCGCGGCCTGGGGCTGCTTCGTCTCGGGATCCCTGCGACCCGGGCGGGGTGCCGCGCCGGGCTCCGCCGCGTGCACTTTCCGCGGGCAGAGCGCCACGACCGGTTTCGCGGCGGTGACGAGCCGGGTGGGAAGGTAGGCG-------AGGGCGCGCTGTTAAAGCCCGCCCTGGTGAGCCGCTGCGGGACCGAGGGACGGCCGTGCTCTTTGAGGCCAGGCCTTCCTGGGGAGTTCGACTGACAGAGACTGGGCAACCGTGTCTGTCGACCGCTCCTCTGGACTGGATCGGGCTGGCCGGGAGCGCTGAGGGTCGGTGGCGAGTCTGTCGGCATTGCACCCGACCCGTCTTGAAACACGGACCAAGGAGTCTAACATGCGCGCGAGTCGTTGGGTTGTACGAAACCCAAAGGCGCAGTGAAAGCGAGGGTCGCCCCGGGCTGACCCAGGTGGGATCTCTCTGGCCCTCGGGCCGGGGAGCGCACCACCGGCCCGTCCCGTCTGCGTTGTCAGTGGGGCGGAGCAGGAGCGTGCACGCTGGGACCCGAAAGATGGTGAACTATGCCTGAGTAGAACGAAGCCAGAGGAA

>ZSM20081019_Microhedyle_glandulifera

AGGATTTCCCCAGTAACGGCGAGTGAAGCGGGAAGAGCCCAGCACCGAATCCCTCAGTGTCATGCTGACGGGAACTGTGGTGTGTGGGACGCCACCAGTCGCACTAGCGGGCGCCGAAGTCCTCCTGATCGAGGCTTTACCCAGAGCGGGTGTAAGGCCAGTGCAGGTGCTTCGCTATGCGGCCGCGAGCGTCTCAGGAGTCGGGTTGTTTGGGAATGCAGCCCTAAGCGGGTGGTAAACTCCATCTAAGGCTAAATACTGGCACGAGTCCGATAGCGGACAAGTACCGTGAGGGAAAGTTGAAAAGAACTTTGAAGAGAGAGTTCAAGAGTACGTGAAACCGCCCAGAGGTAAACGGGTGCATCCGCAAAGTCGGCCCGTGGAATTCAGCGCGGCGCGCGGCCTGGGGCTGCCTCGTCTCGGGATCCCTGGGACCCGGGCGGGGTGCCGCGCCGGGCTCCGCCGCGTGCACTTTCCGCGGGCAGAGCGCCACGACCGGTTTCGCGGCGGTGACGAGCCGGGTGGGAAGGTAGGCG-------AGGGCGCGCTGTTACAGCCCRCCCTGGTGAGCCGCTGCGGGACCGAGGGACGGCCGTGCTCTTCGTGGCCAGGCCTTCCTGGGGAGTTCGACTGACAGAGACTGGGCAACCGTGTCTGTCGACCGCTCCTCTGGACTGGATCGGGCTGGCCGGGAGCGCTGAGGGTCGGTGGCGAGTCTGTCGGCATTGCACCCGACCCGTCTTGAAACACGGACCAAGGAGTCTAACATGCGCGCGAGTCGTTGGGTTGTACGAAACCCAAAGGCGCAGTGAAAGCGAGGGTCGCCCCGGGCTGACCCAGGTGGGATCCCTCTGGCCCTCGGGTCGGGGGGCGCACCACCGGCCCGTCCCGTCTGCGTTGTCAGTGGGGCGGAGCAGGAGCGTGCACGCTGGGACCCGAAAGATGGTGAACTATGCCTGAGTAGAACGAAGCCAGAGGAA

>ZSM20090471

NNNNTTCCCCCAGTAACGGCGAGTGAAGCGGGACGAGCCCAGCACCGAATCCCTCAGTGTCACGCTGACGGGAACTGTGGTGTGTGGGACGCCACCAGTCGCATTAAAGGGCGCCGAAGTCCTCCTGATCGAGGCTTCACCCAGAGCGGGTGTAAGGCCAGTGCTGGTGCCTCTTTGTGCGGCCGCGAGCGTCTCAGGAGTCGGGTTGTTTGGGAATGCAGCCCAAAGCGGGTGGTAAACTCCATCTAAGGCTAAATACCGGCACGAGTCCGATAGCGGACAAGTACCGTGAGGGAAAGTTGAAAAGAACTTTGAAGAGAGAGTTCAAGAGTACGTGAAACCGCCCAGAGGTAAACGGGTGCATCCGCAAAGTCGGCCCGTGGAATTCAGCGCGGCGCGCGGCCTGGGGCTGCTCCGCTTCGGGATCCCTGGGACCCGGGCGGGGTGCTGCGCCGGGCTCCGCCGCGTGCACTTTCTGCGGGCAGAGCGCCACGACCGGTTTCGCGGCGGCGACGAGCCGGGCGGGAAGGTATGCTGCTCCCCGGAGCC-GCTGTTAGAGCCCGTCCCGGTGAGCTGCTGCGGGACCGAGGGTCGGCCGCGCTCTTCGAGGCCAGGCCTTCTCGGGGAGTTCGACTGGCAGCTACTGGGCAACCGTGACTGCCGACCGCTCCTCGAGACCGGATCGGGCTGGCCGAGAGCGCTGAGGGTCTGTGGCGAATCTGTCGGCATTGCACCCGACCCGTCTTGAAACACGGACCAAGGAGTCTAACATGCGCGCGAGTCATTGGGTCGTACGAAACTCAAAGGCGCAGTGAAAGCGAGGGTGGCCCCGGGCCGACCCAGGTGGGATCCCTCCGTCTTCCGGAGCGGGGGGCGCACCACCGGCCCGTCCCGTCCGCGCTGTCGGTGGGGCGGAGCAGGAGCGTGCACGCTGGGACCCGAAAGATGGTGAACTATGCTTGAGTAGAACGAAGCCAGAGGAA

>ZSM20100597

NNNATTCCCCCAGTAACGGCGAGTGAAGCGGGAAGAGCCCAGCACCGAATCCCTCAGTGTCATGCTGACGGGAACTGTGGTGTGTGGGACGCCACCAGTCGCATTAAAGGGCGCCGAAGTCCTCCTGATCGAGGCTTCACCCAGAGCGGGTGTAAGGCCAGTGCTGGTGCCTCTTTGTGCGGCCGCGAGCGTCTCAGGAGTCGGGTTGTTTGGGAATGCAGCCCAAAGCGGGTGGTAAACTCCATCTAAGGCTAAATACCGGCACGAGTCCGATAGCGGACAAGTACCGTGAGGGAAAGTTGAAAAGAACTTTGAAGAGAGAGTTCAAGAGTACGTGAAACCGCCCAGAGGTAAACGGGTGCATCCGCAAAGTCGGCCCGTGGAATTCAGCGCGGCGCGCGGCCTGGGGCTGCTTCGTTTCGGGATCCCTGGGACCCGAGCGGGGTGCTGCGCCGGGCTCCGCCGCGTGCACTTTCTGCGGGTAGAGCGCCACGACCGGTTTCGCGGTGGCGACGAGCCGGGCGGGAAGGTATGCTGCTTC----GGCT-GCTGTTATAGCCCGTCCTGGTGAGCTGCTGCGGGACCGAGGGACGGCCGCGTTCTTCGAGGCCAGGCCTTCTCGGGGAGTTCGACTGGTAGAGACTGGGCAACCGTGTCTGCCGACCGCTTCTCGAGACCGGATCGGGCTGGCCGGGAACGCTGAGGGTCGGTGGCGAATCTGTCGGCATTGCACCCGACCCGTCTTGAAACACGGACCAAGGAGTCTAACATGCGCGCGAGTCATTGGGTCGTACGAAACTCAAAGGCGCAGTGAAAGCGAGGGTCGCCCCGGGCTGACCCAGGTGGGATCCCTCCGTTCCTCGGAGCGGGGGGCGCACCACCGGCCCGTCCCGTCCGCGTTGTCGGTGGGGCGGAGCAGGAGCGTGCACGCTGGGACCCGAAAGATGGTGAACTATGCCTGAGTAGAACGAAGCCAGAGAAA

>ZSM20100603

NNNATTCCCCCAGTAACGGCGAGTGAAGCGGGAAGAGCCCAGCACCGAATCCCTCAGTGTCATGCTGACGGGAACTGTGGTGTGTGGGACGCCACCAGTCGCATTAAAGGGCGCCGAAGTCCTCCTGATCGAGGCTTCACCCAGAGCGGGTGTAAGGCCAGTGCTGGTGCCTCTTTGTGCGGCCGCGAGCGTCTCAGGAGTCGGGTTGTTTGGGAATGCAGCCCAAAGCGGGTGGTAAACTCCATCTAAGGCTAAATACCGGCACGAGTCCGATAGCGGACAAGTACCGTGAGGGAAAGTTGAAAAGAACTTTGAAGAGAGAGTTCAAGAGTACGTGAAACCGCCCAGAGGTAAACGGGTGCATCCGCAAAGTCGGCCCGTGGAATTCAGCGCGGCGCGCGGCCTGGGGCTGCTTCGTTTCGGGATCCCTGGGACCCGAGCGGGGTGCTGCGCCGGGCTCCGCCGCGTGCACTTTCTGCGGGTAGAGCGCCACGACCGGTTTCGCGGTGGCGACGAGCCGGGCGGGAAGGTATGCTGCTTC----GGCT-GCTGTTATAGCCCGTCCTGGTGAGCTGCTGCGGGACCGAGGGACGGCCGCGTTCTTCGAGGCCAGGCCTTCTCGGGGAGTTCGACTGGTAGAGACTGGGCAACCGTGTCTGCCGACCGCTTCTCGAGACCGGATCGGGCTGGCCGGGAACGCTGAGGGTCGGTGGCGAATCTGTCGGCATTGCACCCGACCCGTCTTGAAACACGGACCAAGGAGTCTAACATGCGCGCGAGTCATTGGGTCGTACGAAACTCAAAGGCGCAGTGAAAGCGAGGGTCGCCCCGGGCTGACCCAGGTGGGATCCCTCCGTTCCTCGGAGCGGGGGGCGCACCACCGGCCCGTCCCGTCCGCGTTGTCGGTGGGGCGGAGCAGGAGCGTGCACGCTGGGACCCGAAAGATGGTGAACTATGCCTGAGTAGAACGAAGCCAGAGGAA

>ZSM20100595

NNNNNNNNNNNAGTAACGGCGAGTGAAGCGGGAAGAGCCCAGCACCGAATCCCTCAGTGTCATGCTGACGGGAACTGTGGTGTGTGGGACGCCACCAGTCGCATTAAAGGGCGCCGAAGTCCTCCTGATCGAGGCTTCACCCAGAGCGGGTGTAAGGCCAGTGCTGGTGCCTCTTTGTGCGGCCGCGAGCGTCTCAGGAGTCGGGTTGTTTGGGAATGCAGCCCAAAGCGGGTGGTAAACTCCATCTAAGGCTAAATACCGGCACGAGTCCGATAGCGGACAAGTACCGTGAGGGAAAGTTGAAAAGAACTTTGAAGAGAGAGTTCAAGAGTACGTGAAACCGCCCAGAGGTAAACGGGTGCATCCGCAAAGTCGGCCCGTGGAATTCAGCGCGGCGCGCGGCCTGGGGCTGCTTCGTTTCGGGATCCCTGGGACCCGAGCGGGGTGCTGCGCCGGGCTCCGCCGCGTGCACTTTCTGCGGGTAGAGCGCCACGACCGGTTTCGCGGTGGCGACGAGCCGGGCGGGAAGGTATGCTGCTTC----GGCT-GCTGTTATAGCCCGTCCTGGTGAGCTGCTGCGGGACCGAGGGACGGCCGCGTTCTTCGAGGCCAGGCCTTCTCGGGGAGTTCGACTGGTAGAGACTGGGCAACCGTGTCTGCCGACCGCTTCTCGAGACCGGATCGGGCTGGCCGGGAACGCTGAGGGTCGGTGGCGAATCTGTCGGCATTGCACCCGACCCGTCTTGAAACACGGACCAAGGAGTCTAACATGCGCGCGAGTCATTGGGTCGTACGAAACTCAAAGGCGCAGTGAAAGCGAGGGTCGCCCCGGGCTGACCCAGGTGGGATCCCTCCGTTCCTCGGAGCGGGGGGCGCACCACCGGCCCGTCCCGTCCGCGTTGTCGGTGGGGCGGAGCAGGAGCGTGCACGCTGGGACCCGAAAGATGGTGAACTATGCCTGAGTAGAACGAAGCCAGANNNN

>AMC476062001

NNNNTTCCCCCAGTAACGGCGAGTGAAGCGGGAAGAGCCCAGCACCGAATCCCTCAGTGTGATGCTGACGGGAACTGTGGTGTGTGGGACGCCACCAGTCGCATTAAAGGGCGCCGAAGTCCTCCTGATCGAGGCTTCACCCAGAGCGGGTGTAAGGCCAGTGCTGGTGCCTCTTTGTGCGGCCGCGAGCGTCTCAGGAGTCGGGTTGTTTGGGAATGCAGCCCAAAGCGGGTGGTAAACTCCATCTAAGGCTAAATACCGGCACGAGTCCGATAGCGGACAAGTACCGTGAGGGAAAGTTGAAAAGAACTTTGAAGAGAGAGTTCAAGAGTACGTGAAACCGCCCAGAGGTAAACGGGTGCATCCGCAAAGTCGGCCCGTGGAATTCAGCGCGGCGCGCGGCCTGGGGCTGCTTCGTTCCGGGATCCCTGGGACCCGAGCGGGGTGCTGCGCCGGGCTCCGCCGCGTGCACTTTCTGCGGGCAGAGCGCCACGACCGGTTTCGCGGCGGCGACGAGCCGGACGGGAAGGTAGGCG-------AGGACGCGCTGTTACAGCCCGCCCTGGTGAGCTGCTGCGGGACCGAGGGACGGCCGCGTTCTTCGAGGCCAGGCCTTCTCGGGGAGTTCGACTGGTAGAGACTGGGCAACCGTGTCTGCCGACCGCTTCTCGAGACCGGATCGGGCTGGCCGGGAGCGCTGAGGGTCGGTGGCGAATCTGTCGGCATTGCACCCGACCCGTCTTGAAACACGGACCAAGGAGTCTAACATGCGCGCGAGTCATTGGGTCGTACGAAACTCAAAGGCGCAGTGAAAGCGAGGGTCGCCCCGGGCTGACCCAGGTGGGATCTCTCCGTTCCTCGGAGCGGGGAGCGCACCACCGGCCCGTCCCGTCCGCGTTGTCGGTGGGGCGGAGCAGGAGCGTGCACGCTGGGACCCGAAAGATGGTGAACTATGCCTGAGTAGAACGAAGCCAGAGGAA

>ZSM20100389

NNNATTCCCCCAGTAACGGCGAGTGAAGCGGGAAGAGCCCAGCACCGAATCCCTCAGTGTCATGCTGGCGGGAACTGTGGTGTGTGGGACGCCACCAGTCG-ATTAAAGGGCGCCGAAGTCCTCCTGATCGAGGCTTCACCCAGAGCGGGTGTAAGGCCAGTGCTGGTGCCTCTTTGTGCGGCCGCGAGCGTCTCAGGAGTCGGGTTGTTTGGGAATGCAGCCCAAAGCGGGTGGTAAACTCCATCTAAGGCTAAATACCGGCACGAGTCCGATAGCGGACAAGTACCGTGAGGGAAAGTTGAAAAGAACTTTGAAGAGAGAGTTCAAGAGTACGTGAAACCGCCCAGAGGTAAACGGGTGCATCCGCAAAGTCGGCCCGTGGAATTCAGCGCGGCGCGCGGCCTGGGGCTGCTTCGTTCCGGGATCCCTGGGACCCGGGCGGGGTGCTGCGCCGGGCTCCGCCGCGTGCACTTTCTGCGGGCAGAGCGCCACGACCGGTTTCGCGGCGGCGACGAGCCGGGCGGGAAGGTAGGCG-------AGGGCGCGCTGTTACAGCCCGCCCTGGTGAGCTGCTGCGGGACCGAGGGACGGCCGTGCTCTTGGAGGCCAGGCCTTCTCGGGGAGTTCGACTGGTAGAGACTGGGCAACCGTGTCTGCCGACCGCTTCTCGAGACCGGATCGGGCTGGCCGGGAGCGCTGAGGGTCGGTGGCGAATCTGTCGGCATTGCACCCGACCCGTCTTGAAACACGGACCAAGGAGTCTAACATGCGCGCGAGTCATTGGGTCGTACGAAACTCAAAGGCGCAGTGAAAGCGAGGGTCGCCCCGGGCTGACCCAGGTGGGATCTCTCCGCTCC----GGCGGGGAGCACACCACCGGCCCGTCCCGTCCGCGTTGTCGGTGGGGCGGAGCAGGAGCGTGCACGCTGGGACCCGAAAGATGGTGAACTATGCCTGAGTAGAACGAAGCCAGAGGAA

>ZSM20071820

AGGATTCCCCCAGTAACGGCGAGTGAAGCGGGAAGAGCCCAGCACCGAATCCCTCAGTGTCATGCTGACGGGAACTGTGGTGTGTGGGACGCCACCAGTCGCATTAAAGGGCGCCGAAGTCCTCCTGATCGAGGCTTCACCCAGAGCGGGTGTAAGGCCAGTGCTGGTGCCTCTTTGTGCGGCCGCGAGCGTCTCAGGAGTCGGGTTGTTTGGGAATGCAGCCCAAAGCGGGTGGTAAACTCCATCTAAGGCTAAATACCGGCACGAGTCCGATAGCGGACAAGTACCGTGAGGGAAAGTTGAAAAGAACTTTGAAGAGAGAGTTCAAGAGTACGTGAAACCGCCCAGAGGTAAACGGGTGCATCCGCAAAGTCGGCCCGTGGAATTCAGCGCGGCGCGCGGCCTGGGGCTGCTTCGTTCCGGGATCCCTGGGACCCGGGCGGGGTGCTGCGCCGGGCTCCGCCGCGTGCACTTTCTGCGGGCAGAGCGCCACGACCGGTTTCGCGGCGGCGACGAGCCGGGCGGGAAGGTAGGCG-------AGGGCGCGCTGTTACAGCCCGCCCTGGTGAGCTGCTGCGGGACCGAGGGACGGCCGTGCTCTTGGAGGCCAGGCCTTCTCGGGGAGTTCGACTGGTAGAGACTGGGCAACCGTGTCTGCCGACCGCTTCTCGAGACCGGATCGGGCTGGCCGGGAGCGCTGAGGGTCGGTGGCGAATCTGTCGGCATTGCACCCGACCCGTCTTGAAACACGGACCAAGGAGTCTAACATGCGCGCGAGTCATTGGGTCGTACGAAACTCAAAGGCGCAGTGAAAGCGAGGGTCGCCCCGGGCTGACCCAGGTGGGATCTCTCCGCTCC----GGCGGGGAGCGCACCACCGGCCCGTCCCGTCCGCGTTGTCGGTGGGGCGGAGCAGGAGCGTGCACGCTGGGACCCGAAAGATGGTGAACTATGCCTGAGTAGAACGAAGCCAGAGGAA

>ZSM20100390

AGGATTCCCCCAGTAACGGCGAGTGAAGCGGGAAGAGCCCAGCACCGAATCCCTCAGTGTCATGCTGACGGGAACTGTGGTGTGTGGGACGCCACCAGTCGCATTAAAGGGCGCCGAAGTCCTCCTGATCGAGGCTTCACCCAGAGCGGGTGTAAGGCCAGTGCTGGTGCCTCTTTGTGCGGCCGCGAGCGTCTCAGGAGTCGGGTTGTTTGGGAATGCAGCCCAAAGCGGGTGGTAAACTCCATCTAAGGCTAAATACCGGCACGAGTCCGATAGCGGACAAGTACCGTGAGGGAAAGTTGAAAAGAACTTTGAAGAGAGAGTTCAAGAGTACGTGAAACCGCCCAGAGGTAAACGGGTGCATCCGCAAAGTCGGCCCGTGGAATTCAGCGCGGCGCGCGGCCTGGGGCTGCTTCGTTCCGGGATCCCTGGGACCCGGGCGGGGTGCTGCGCCGGGCTCCGCCGCGTGCACTTTCTGCGGGCAGAGCGCCACGACCGGTTTCGCGGCGGCGACGAGCCGGGCGGGAAGGTAGGCG-------AGGGCGCGCTGTTACAGCCCGCCCTGGTGAGCTGCTGCGGGACCGAGGGACGGCCGTGCTCTTGGAGGCCAGGCCTTCTCGGGGAGTTCGACTGGTAGAGACTGGGCAACCGTGTCTGCCGACCGCTTCTCGAGACCGGATCGGGCTGGCCGGGAGCGCTGAGGGTCGGTGGCGAATCTGTCGGCATTGCACCCGACCCGTCTTGAAACACGGACCAAGGAGTCTAACATGCGCGCGAGTCATTGGGTCGTACGAAACTCAAAGGCGCAGTGAAAGCGAGGGTCGCCCCGGGCTGACCCAGGTGGGATCTCTCCGCTCC----GGCGGGGAGCGCACCACCGGCCCGTCCCGTCCGCGTTGTCGGTGGGGCGGAGCAGGAGCGTGCACGCTGGGACCCGAAAGATGGTGAACTATGCCTGAGTAGAACGAAGCCAGAGGAA

>SICBC2010KJ01C08

AGGATTCCCCCAGTAACGGCGAGTGAAGCGGGAAGAGCCCAGCACCGAATCCCTCAGTGTCATGCTGACGGGAACTGTGGTGTGTGGGACGCCACCAGTCGCATTAACGGGCGCCGAAGTCCTCCTGATCGAGGCTTCACCCAGAGCGGGTGTAAGGCCAGTGCTGGTGCCTCGTTGTGCGGCCGCGAGCGTCTCAGGAGTCGGGTTGTTTGGGAATGCAGCCCAAAGCGGGTGGTAAACTCCATCTAAGGCTAAATACCGGCACGAGTCCGATAGCGGACAAGTACCGTGAGGGAAAGTTGAAAAGAACTTTGAAGAGAGAGTTCAAGAGTACGTGAAACCGCCCAGAGGTAAACGGGTGCATCCGCAAAGTCGGCCCGTGGAATTCAGCGCGGCGCGCGGCCTGGGGCTGCTTCGCTTCGGGATCCCTGGGACCCGGGCGAGGTGCTGCGCCGGGCTCCGCCGCGTGCACTTTCTGCGGGCAGAGCGCCACGACCGGTTTCGCGGCGGCGACGAGCCGGGCGGGAAGGTAGGCG-------AGGACGTGCTGTTACAGCCCGTCCTGGTGAGCTGCTGCGGGACCGAGGGACGGCCGCGTTCTTCGAGGCCAGGCCTTCTCGGGGAGTTCGACTGGTAGAGACTGGGCAACCGTGTCTGCCGACCGCTTCTCGAGACCGGATCGGGCTGGCCGGGAGCGCTGAGGGTCGGTGGCGAATCTGTCGGCATTGCACCCGACCCGTCTTGAAACACGGACCAAGGAGTCTAACATGCGCGCGAGTCATTGGGTCGTACGAAACTCAAAGGCGCAGTGAAAGCGAGGGTCGCCCCGGGCTGACCCAGGTGGGATCTCTCCGTGCC----AACGGGGAGCGCACCACCGGCCCGTCCCGTCCGCGTTGTCGGTGGGGCGGAGCAGGAGCGTGCACGCTGGGACCCGAAAGATGGTGAACTATGCCTGAGTAGAACGAAGCCAGAGGAA

>ZSM20090197

AGGATTCCCCCAGTAACGGCGAGTGAAGCGGGAAGAGCCCAGCACCGAATCCCTCAGTGTCATGCTGACGGGAACTGTGGTGTGTGGGACGCCACCAGTCGCATTAACGGGCGCCGAAGTCCTCCTGATCGAGGCTTCACCCAGAGCGGGTGTAAGGCCAGTGCTGGTGCCTCGTTGTGCGGCCGCGAGCGTCTCAGGAGTCGGGTTGTTTGGGAATGCAGCCCAAAGCGGGTGGTAAACTCCATCTAAGGCTAAATACCGGCACGAGTCCGATAGCGGACAAGTACCGTGAGGGAAAGTTGAAAAGAACTTTGAAGAGAGAGTTCAAGAGTACGTGAAACCGCCCAGAGGTAAACGGGTGCATCCGCAAAGTCGGCCCGTGGAATTCAGCGCGGCGCGCGGCCTGGGGCTGCTTCGCTTCGGGATCCCTGGGACCCGGGCGAGGTGCTGCGCCGGGCTCCGCCGCGTGCACTTTCTGCGGGCAGAGCGCCACGACCGGTTTCGCGGCGGCGACGAGCCGGGCGGGAAGGTAGGCG-------AGGACGTGCTGTTACAGCCCGTCCTGGTGAGCTGCTGCGGGACCGAGGGACGGCCGCGTTCTTCGAGGCCAGGCCTTCTCGGGGAGTTCGACTGGTAGAGACTGGGCAACCGTGTCTGCCGACCGCTTCTCGAGACCGGATCGGGCTGGCCGGGAGCGCTGAGGGTCGGTGGCGAATCTGTCGGCATTGCACCCGACCCGTCTTGAAACACGGACCAAGGAGTCTAACATGCGCGCGAGTCATTGGGTCGTACGAAACTCAAAGGCGCAGTGAAAGCGAGGGTCGCCCCGGGCTGACCCAGGTGGGATCTCTCCGTGCC----AACGGGGAGCGCACCACCGGCCCGTCCCGTCCGCGTTGTCGGTGGGGCGGAGCAGGAGCGTGCACGCTGGGACCCGAAAGATGGTGAACTATGCCTGAGTAGAACGAAGCCAGAGGAA

>ZSM20081013

AGGATTCCCCCAGTAACGGCGAGTGAAGCGGGAAGAGCCCAGCACCGAATCCCTCAGTGTCATGCTGACGGGAACTGTGGTGTGTGGGACGCCACCAGTCGCATTAACGGGCGCCGAAGTCCTCCTGATCGAGGCTTCACCCAGAGCGGGTGTAAGGCCAGTGCTGGTGCCTCGTTGTGCGGCCGCGAGCGTCTCAGGAGTCGGGTTGTTTGGGAATGCAGCCCAAAGCGGGTGGTAAACTCCATCTAAGGCTAAATACCGGCACGAGTCCGATAGCGGACAAGTACCGTGAGGGAAAGTTGAAAAGAACTTTGAAGAGAGAGTTCAAGAGTACGTGAAACCGCCCAGAGGTAAACGGGTGCATCCGCAAAGTCGGCCCGTGGAATTCAGCGCGGCGCGCGGCCTGGGGTTGCTTCGTCTCGGGATCCCTGGGACCCGGGCGGGGTGCTGCGCCGGGCTCCGCCGCGTGCACTTTCTGCGGGCAGAGCGCCACGACCGGTTTCGCGGCGGCGACGAGCCGGGCGGGAAGGTAGGCG-------AGGACGCGCTGTTACAGCCCGCCCTGGTGAGCTGCTGCGGGACCGAGGGACGGCCGCGTTCTTCGAGGCCAGGCCTTCTCGGGGAGTTCGACTGGTAGAGACTGGGCAACCGTGTCTGCCGACCGCTTCTCGAGACCGGATCGGGCTGGCCGGGAGCGCTGAGGGTCGGTGGCGAGTCTGTCGGCATTGCACCCGACCCGTCTTGAAACACGGACCAAGGAGTCTAACATGCGCGCGAGTCATTGGGTCGTACGAAACTCAAAGGCGCAGTGAAAGCGAGGGTCGCCCCGGGCTGACCCAGGTGGGATCTCTCCGTGCCTCGGCGCGGGGAGCGCACCACCGGCCCGTCCCGTCCGCGTTGTCGGTGGGGCGGAGCAGGAGCGTGCACGCTGGGACCCGAAAGATGGTGAACTATGCCTGAGTAGAACGAAGCCAGAGGAA

>ZSM20080054

NNNNNTCCCCCAGTAACGGCGAGTGAAGCGGGAAGAGCCCAGCACCGAATCCCTCAGTGTCATGCTGACGGGAACTGTGGTGTGTGGGACGCCACCAGTCGCACATGCGGGCGCCGAAGTCCTCCTGATCGAGGCTTCACCCAGAGCGGGTGTAAGGCCAGTGCTGGTGCTTCGCTGTGCGGCCGCGAGCGTCTCAGGAGTCGGGTTGTTTGGGAATGCAGCCCAAAGCGGGTGGTAAACTCCATCTAAGGCTAAATACCGGCACGAGTCCGATAGCGGACAAGTACCGTGAGGGAAAGTTGAAAAGAACTTTGAAGAGAGAGTTCAATAGTACGTGAAACCGCCCAGAGGTAAACGGGTGCATCCGCAAAGTCGGCCCGTGGAATTCAGCGCGGCGCGCGGCCTGGGGCTGCTTCGTTCCGGGATCCCTGGGACCCGAGCGGGGTGCTGCGCCGGGCTCCGCCGCGTGCACTTTCTGCGGGCAGAGCGCCACGACCGGTTTCGCGGCGGCGACGAGCCGGGCGGGAAGGTAGGCGTCTCCTTCGGGCGCGCTGTTATAGACCGCCCTGGTGAGCTGCTGCGGGACCGAGGGACGGCCGCGTTCTTCGAGGCCAGGCTTTCTCGGGGAGTTCGACTGGCAGAGACTGGGCAACCGTGTCTGCCGACCGCTTTCCGGGACCGGACCGGGCTGGCCGGGAGCGCTGAGGGTCGGTGGCGAATCTGTCGGCATTGCACCCGACCCGTCTTGAAACACGGACCAAGGAGTCTAACATGCGCGCGAGTCATTGGGTTGTACGAAACCCAAAGGCGCAGTGAAAGCGAGGGTCGCCCCGGGCTGACCCAGGTGGGATCTTTCCGTTCTCCGGAGCGGGGAGCGCACCACCGGCCCGTCCCGTCCGCGTTGTCGGTGGGGCGGAGCAGGAGCGTGCACGCTGGGACCCGAAAGATGGTGAACTATGCCTGAGTAGAACGAAGCCAGANNNN

>SICBC2010KJ01B07

AGGATTCCCCCAGTAACGGCGAGTGAAGCGGGAAGAGCCCAGCACCGAATCCCTCAGTGTCATGCTGACGGGAACTGTGGTGTGTGGGACGCCACCAGTCGCACATGCGGGCGCCGAAGTCCTCCTGATCGAGGCTTCACCCAGAGCGGGTGTAAGGCCAGTGCTGGTGCTTCGCTGTGCGGCCGCGAGCGTCTCAGGAGTCGGGTTGTTTGGGAATGCAGCCCAAAGCGGGTGGTAAACTCCATCTAAGGCTAAATACCGGCACGAGTCCGATAGCGGACAAGTACCGTGAGGGAAAGTTGAAAAGAACTTTGAAGAGAGAGTTCAAGAGTACGTGAAACCGCCCAGAGGTAAACGGGTGCATCCGCAAAGTCGGCCCGTGGAATTCAGCGCGGCGCGCGGCCTGGAGCTGCTTCGTTTCGGGATCCCTGGGACCCGGGCGGGGTGCTGCGCCGGGCCCCGCCGCGTGCACTTTCTGCGGGCAGAGCGCCACGACCGGTTTCGCGGCGGCGACGAGCCGGGCGGGAAGGTAGGCGTCTCCTTCGGGCGCGCTGTTATAGACCGCCTCGGTGAGCTGCTGCGGGACCGAGGGACGGCCGCGTTCTTCGAGGCCAGGCTTTCTCGGGGAGTTCGACTGGCAGAGACTGAGCAATCGTGTCTGCCGACCGCTTCTCGA-GTCGGTCCGGGCTGGCCGGGAGCGCTCAGGGTCGGTGGCGAATCTGTCGGCATTGCACCCGACCCGTCTTGAAACACGGACCAAGGAGTCTAACATGCGCGCGAGTCATTGGGTTCTACGAAATCCAAAGGCGCAGTGAAAGCGAGGGTCGCCCCGGGCTGACCCAGGTGGGATCTTTCCGTCTCTCGGAGCGGGGAGCGCACCACCGGCCCGTCCCGTCCGCGTCGTCGGTGGGGCGGAGCAGGAGCGTGCACGCTGGGACCCGAAAGATGGTGAACTATGCCTGAGTAGAACGAAGCCAGANNNN

>SICBC2010KJ01D07

NNNNTTCCCCCAGTAACGGCGAGTGAAGCGGGAAGAGCCCAGCACCGAATCCCTCAGCGTCATGCTGACGGGAACTGTGGTGTGTGGGACGCCACCAGTCGCACATGCGGGCGCCGAAGTCCTCCTGATCGAGGCTTCACCCAGAGCGGGTGTAAGGCCAGTGCTGGTGCTTCGCTGTGCGGCCGCGAGCGTCTCAGGAGTCGGGTTGTTTGGGAATGCAGCCCAAAGCGGGTGGTAAACTCCATCTAAGGCTAAATACCGGCACGAGTCCGATAGCGGACAAGTACCGTGAGGGAAAGTTGAAAAGAACTTTGAAGAGAGAGTTCAAGAGTACGTGAAACCGCCCAGAGGTAAACGGGTGCATCCGCAAAGTCGGCCCGTGGAATTCAGCGCGGCGCGCGGCCTGGAGCTGCTTCGTTTCGGGATCCCTGGGACCCGGGCGGGGTGCTGCGCCGGGCCCCGCCGCGTGCACTTTCTGCGGGCAGAGCGCCACGACCGGTTTCGCGGCGGCGACGAGCCGGGCGGGAAGGTAGGCGTCTCCTTCGGGCGCGCTGTTATAGACCGCCTCGGTGAGCTGCTGCGGGACCGAGGGACGGCCGCGTTCTTCGAGGCCAGGCTTTCTCGGGGAGTTCGACTGGCAGAGACTGAGCAATCGTGTCTGCCGACCGCTTCTCGA-GTCGGTCCGGGCTGGCCGGGAGCGCTCAGGGTCGGTGGCGAATCTGTCGGCATTGCACCCGACCCGTCTTGAAACACGGACCAAGGAGTCTAACATGCGCGCGAGTCATTGGGTTCTACGAAATCCAAAGGCGCAGTGAAAGCGAGGGTCGCCCCGGGCTGACCCAGGTGGGATCTTTCCGTCTCTCGGAGCGGGGAGC-CACCACCGGCCCGTCCCGTCCGCGTCGTCGGTGGGGCGGAGCAGGAGCGTGCACGCTGGGACCCGAAAGATGGTGAACTATGCCTGAGTAGAACGAAGCCAGAGGAA

>SICBC2010KJ01B09

AGGATTCCCCCAGTAACGGCGAGTGAAGCGGGAAGAGCCCAGCACCGAATCCCTCAGTGTCATGCTGACGGGAACTGTGGTGTGTGGGACGCCACCAGTCGCACATGCGGGCGCCGAAGTCCTCCTGATCGAGGCTTCACCCAGAGCGGGTGTAAGGCCAGTGCTGGTGCTTCGCTGTGCGGCCGCGAGCGTCTCAGGAGTCGGGTTGTTTGGGAATGCAGCCCAAAGCGGGTGGTAAACTCCATCTAAGGCTAAATACCGGCACGAGTCCGATAGCGGACAAGTACCGTGAGGGAAAGTTGAAAAGAACTTTGAAGAGAGAGTTCAAGAGTACGTGAAACCGCCCAGAGGTAAACGGGTGCATCCGCAAAGTCGGCCCGTGGAATTCAGCGCGGCGCGCGGCCTGGAGCTGCTTCGTTTCGGGATCCCTGGGACCCGGGCGGGGTGCTGCGCCGGGCCCCGCCGCGTGCACTTTCTGCGGGCAGAGCGCCACGACCGGTTTCGCGGCGGCGACGAGCCGGGCGGGAAGGTAGGCGTCTCCTTCGGGCGCGCTGTTATAGACCGCCACGGTGAGCTGCTGCGGGACCGAGGGACGGCCGCGTTCTTCGAGGCCAGGCTTTCTCGGGGAGTTCGACTGGCAGAGACTGAGCAATCGTGTCTGCCGACCGCTTCTCGA-GTCGGTCCGGGCTGGCCGGGAGCGCTCAGGGTCGGTGGCGAATCTGTCGGCATTGCACCCGACCCGTCTTGAAACACGGACCAAGGAGTCTAACATGCGCGCGAGTCATTGGGTTCTACGAAATCCAAAGGCGCAGTGAAAGCGAGGGTCGCCCCGGGCTGACCCAGGTGGGATCTTTCCGTCTCTCGGAGCGGGGAGCGCACCACCGGCCCGTCCCGTCCGCGTCGTCGGTGGGGCGGAGCAGGAGCGTGCACGCTGGGACCCGAAAGATGGTGAACTATGCCTGAGTAGAACGAAGCCAGANNNN

>SICBC2010KJ01C09

AGGATTCCCCCAGTAACGGCGAGTGAAGCGGGAAGAGCCCAGCACCGAATCCCTCAGTGTCATGCTGACGGGAACTGTGGTGTGTGGGACGCCACCAGTCGCACATGCGGGCGCCGAAGTCCTCCTGATCGAGGCTTCACCCAGAGCGGGTGTAAGGCCAGTGCTGGTGCTTCGCTGTGCGGCCGCGAGCGTCTCAGGAGTCGGGTTGTTTGGGAATGCAGCCCAAAGCGGGTGGTAAACTCCATCTAAGGCTAAATACCGGCACGAGTCCGATAGCGGACAAGTACCGTGAGGGAAAGTTGAAAAGAACTTTGAAGAGAGAGTTCAAGAGTACGTGAAACCGCCCAGAGGTAAACGGGTGCATCCGCAAAGTCGGCCCGTGGAATTCAGCGCGGCGCGCGGCCTGGAGCTGCTTCGTTTCGGGATCCCTGGGACCCGGGCGGGGTGCTGCGCCGGGCCCCGCCGCGTGCACTTTCTGCGGGCAGAGCGCCACGACCGGTTTCGCGGCGGCGACGAGCCGGGCGGGAAGGTAGGCGTCTCCTTCGGGCGCGCTGTTATAGACCGCCTCGGTGAGCTGCTGCGGGACCGAGGGACGGCCGCGTTCTTCGAGGCCAGGCTTTCTCGGGGAGTTCGACTGGCAGAGACTGAGCAATCGTGTCTGCCGACCGCTTCTCGA-GTCGGTCCGGGCTGGCCGGGAGCGCTCAGGGTCGGTGGCGAATCTGTCGGCATTGCACCCGACCCGTCTTGAAACACGGACCAAGGAGTCTAACATGCGCGCGAGTCATTGGGTTCTACGAAATCCAAAGGCGCAGTGAAAGCGAGGGTCGCCCCGGGCTGACCCAGGTGGGATCTTTCCGTCTCTCGGAGCGGGGAGCGCACCACCGGCCCGTCCCGTCCGCGTCGTCGGTGGGGCGGAGCAGGAGCGTGCACGCTGGGACCCGAAAGATGGTGAACTATGCCTGAGTAGAACGAAGCCAGAGGAA

>ZSM20090198

AGGATTCCCCCAGTAACGGCGAGTGAAGCGGGAAGAGCCCAGCACCGAATCCCTCAGTGTCATGCTGACGGGAACTGTGGTGTGTGGGACGCCACCAGTCGCACATGCGGGCGCCGAAGTCCTCCTGATCGAGGCTTCACCCAGAGCGGGTGTAAGGCCAGTGCTGGTGCTTCGCTGTGCGGCCGCGAGCGTCTCAGGAGTCGGGTTGTTTGGGAATGCAGCCCAAAGCGGGTGGTAAACTCCATCTAAGGCTAAATACCGGCACGAGTCCGATAGCGGACAAGTACCGTGAGGGAAAGTTGAAAAGAACTTTGAAGAGAGAGTTCAAGAGTACGTGAAACCGCCCAGAGGTAAACGGGTGCATCCGCAAAGTCGGCCCGTGGAATTCAGCGCGGCGCGCGGCCTGGAGCTGCTTCGTTTCGGGATCCCTGGGACCCGGGCGGGGTGCTGCGCCGGGCCCCGCCGCGTGCACTTTCTGCGGGCAGAGCGCCACGACCGGTTTCGCGGCGGCGACGAGCCGGGCGGGAAGGTAGGCGTCTCCTTCGGGCGCGCTGTTATAGACCGCCACGGTGAGCTGCTGCGGGACCGAGGGACGGCCGCGTTCTTCGAGGCCAGGCTTTCTCGGGGAGTTCGACTGGCAGAGACTGAGCAATCGTGTCTGCCGACCGCTTCTCGA-GTCGGTCCGGGCTGGCCGGGAGCGCTCAGGGTCGGTGGCGAATCTGTCGGCATTGCACCCGACCCGTCTTGAAACACGGACCAAGGAGTCTAACATGCGCGCGAGTCATTGGGTTCTACGAAATCCAAAGGCGCAGTGAAAGCGAGGGTCGCCCCGGGCTGACCCAGGTGGGATCTTTCCGTCTCTCGGAGCGGGGAGCGCACCACCGGCCCGTCCCGTCCGCGTCGTCGGTGGGGCGGAGCAGGAGCGTGCACGCTGGGACCCGAAAGATGGTGAACTATGCCTGAGTAGAACGAAGCCAGAGGAA

>ZSM20100592

AGGATTTCCCCAGTAACGGCGAGTGAAGCGGGAAGAGCCCAGCACCGAATCCCTCAGTGTCATGCTGACGGGAACTGTGGTGTGTGGGACGCCACCAGTCGCACATGCGGGCGCCGAAGTCCTCCTGATCGAGGCTTCACCCAGAGCGGGTGTAAGGCCAGTGCTGGTGCTTCGCTGTGCGGCCGCGAGCGTCTCAGGAGTCGGGTTGTTTGGGAATGCAGCCCAAAGCGGGTGGTAAACTCCATCTAAGGCTAAATACCGGCACGAGTCCGATAGCGGACAAGTACCGTGAGGGAAAGTTGAAAAGAACTTTGAAGAGAGAGTTCAAGAGTACGTGAAACCGCCCAGAGGTAAACGGGTGCATCCGCAAAGTCGGCCCGTGGAATTCAGCGCGGCGCGCGGCCCGGGGCTGCCTCGCTTCGGGATCCCTGGGACCCGAGCGGGGTGCCGCGCCGGGCTCCGCCGCGTGCACTTTCTGCGGGCAGAGCGCCACGACCGGTTTCGCGGCGGCGACGAGCCGGGCGGGAAGGTAGGCGTCAGCTTCGGCCGCGCTGTTACAGACCGCCCTGGTGAGCTGCTGCGGGACCGAGGGACGGCCGCGTTCTTCGAGGCCAGGCTTTCTCGGGGAGTTCGACTGGCAGAGACTGGGCAACCGTGTCTGCCGACCGCTTCTCGAGACGGGACCGGGCTGGCCGGGAGCGCTGAGGGTCGGTGGCGAATCTGTCGGCATTGCACCCGACCCGTCTTGAAACACGGACCAAGGAGTCTAACATGCGCGCGAGTCATTGAGTTGTACGAAACCCAAAGGCGCAGTGAAAGCGAGGGTCGCCCCGGGCTGACCCAGGTGGGATCTTTCCGTTCCTCGGAGCGGGGAGCGCACCACCGGCCCGTCCCGTCCGCGTCGTCGGTGGGGCGGAGCAGGAGCGTGCACGCTGGGACCCGAAAGATGGTGAACTATGCTTGAGTAGAACGAAGCCAGAGAAN

>AMC476054001

AGGATTCCCCCAGTAACGGCGAGTGAAGCGGGAAGAGCCCAGCACCGAATCCCTCAGTGTCATGCTGACGGGAACTGTGGTGTGTGGGACGCCACCAGTCGCACATGCGGGCGCCGAAGTCCTCCTGATCGAGGCTTCACCCAGAGCGGGTGTAAGGCCAGTGCTGGTGCTTCGCTGTGCGGCCGCGAGCGTCTCAGGAGTCGGGTTGTTTGGGAATGCAGCCCAAAGCGGGTGGTAAACTCCATCTAAGGCTAAATACCGGCACGAGTCCGATAGCGGACAAGTACCGTGAGGGAAAGTTGAAAAGAACTTTGAAGAGAGAGTTCAAGAGTACGTGAAACCGCCCAGAGGTAAACGGGTGCATCCGCAAAGTCGGCCCGTGGAATTCAGCGCGGCGCGCGGCCTGGAGCTGCTTCGTTTCGGGATCCCTGGGACCCGGGCGGGGTGCTGCGCCGGGCTCCGCCGCGTGCACTTTCTGCGGGCAGAGCGCCACGACCGGTTTCGCGGCGGCGACGAGCCGGGCGGGAAGGTAGGCGTCCGCTTCGGTGGCGCTGTTATAGACCGCCCTGGTGAGCTGCTGCGGGACCGAGGGACGGCCGCGCTCTTCGAGGCCAGGCTTTCTAGGGGAGTTCGACTGGCAGAGACTGGGCAACCGTGTCTGCCGACCGCTCCTCGAGACCGGACCGGGCTGGCCGGGAGCGCTGAGGGTCTGTGGCGAATCTGTCGGCATTGCACCCGACCCGTCTTGAAACACGGACCAAGGAGTCTAACATGCGCGCGAGTCATTGGGTTGTACGAAACCCAAAGGCGCAGTGAAAGCGAGGGTCGCCCCGGGCTGACCCAGGTGGGATCTTTCCGTCCCTCGGGGCGGGGAGCGCACCACCGGCCCGTCCCGTCCGCGTTGTCGGTGGGGCGGAGCAGGAGCGTGCACGCTGGGACCCGAAAGATGGTGAACTATGCCTGAGTAGAACGAAGCCAGAGAAN

>ZSM20081014

AGGATTCCCCCAGTAACGGCGAGTGAAGCGGGAAGAGCCCAGCACCGAATCCCTCAGTGTCATGCTGACGGGAACTGTGGTGTGTGGGACGCCACCAGTCGCACATGCGGGCGCCGAAGTCCTCCTGATCGAGGCTTCACCCAGAGCGGGTGTAAGGCCAGTGCTGGTGCTTCGCTGTGCGGCCGCGAGCGTCTCAGGAGTCGGGTTGTTTGGGAATGCAGCCCAAAGCGGGTGGTAAACTCCATCTAAGGCTAAATACCGGCACGAGTCCGATAGCGGACAAGTACCGTGAGGGAAAGTTGAAAAGAACTTTGAAGAGAGAGTTCAAGAGTACGTGAAACCGCCCAGAGGTAAACGGGTGCATCCGCAAAGTCGGCCCGTGGAATTCAGCGCGGCGCGCGGCCTGGGGCTGCTTCGTTTCGGGATCCCTGGGACCCGAGCGGGGTGCCGCGCCGGGCTCCGCCGCGTGCACTTTCTGCGGGCAGAGCGCCACGACCGGTTTCGCGGCGGCGACGAGCCGGGCGGGAAGGTAGGCGTCAGCTTCGGCCGCGCTGTTATAGACCGTCCTGGTGAGCTGCTGCGGGACCGAGGGACGGCCGCGTTCTTCGAGGCCAGGCTTTCTCGGGGAGTTCGACTGGCAGAGACTGGGCAACCGTGTCTGCCGACCGCTTCTCGAGACCGGACCGGGCTGGCCGGGAGCGCTGAGGGTCGGTGGCGAATCTGTCGGCATTGCACCCGACCCGTCTTGAAACACGGACCAAGGAGTCTAACATGCGCGCGAGTCATTGGGTTGTACGAAACCCAAAGGCGCAGTGAAAGCGAGGGTCGCCCCGGGCTGACCCAGGTGGGATCTTTCCGTCTCCTGGAGCGGGGAGCGCACCACCGGCCCGTCCCGTCCGCGTCGTCGGTGGGGCGGAGCAGGAGCGTGCACGCTGGGACCCGAAAGATGGTGAACTATGCCTGAGTAGAACGAAGCCAGAGGAA

>ZSM20100379

AGGATTCCCCCAGTAACGGCGAGTGAAGCGGGAAGAGCCCAGCACCGAATCCCTCAGTGTCATGCTGACGGGAACTGTGGTGTGTGGGACGCCACCAGTCGCACATGCGGGCGCCGAAGTCCTCCTGATCGAGGCTTCACCCAGAGCGGGTGTAAGGCCAGTGCTGGTGCTTCGCTGTGCGGCCGCGAGCGTCTCAGGAGTCGGGTTGTTTGGGAATGCAGCCCAAAGCGGGTGGTAAACTCCATCTAAGGCTAAATACCGGCACGAGTCCGATAGCGGACAAGTACCGTGAGGGAAAGTTGAAAAGAACTTTGAAGAGAGAGTTCAAGAGTACGTGAAACCGCCCAGAGGTAAACGGGTGCATCCGCAAAGTCGGCCCGTGGAATTCAGCGCGGCGCGCGGCCTGGGGCTGCTTCGTTTCGGGATCCCTGGGACCCGAGCGGGGTGCCGCGCCGGGCTCCGCCGCGTGCACTTTCTGCGGGCAGAGCGCCACGACCGGTTTCGCGGCGGCGACGAGCCGGGCGGGAAGGTAGGCGTCAGCTTCGGCCGCGCTGTTATAGACCGTCCTGGTGAGCTGCTGCGGGACCGAGGGACGGCCGCGTTCTTCGAGGCCAGGCTTTCTCGGGGAGTTCGACTGGCAGAGACTGGGCAACCGTGTCTGCCGACCGCTTCTCGAGACCGGACCGGGCTGGCCGGGAGCGCTGAGGGTCGGTGGCGAATCTGTCGGCATTGCACCCGACCCGTCTTGAAACACGGACCAAGGAGTCTAACATGCGCGCGAGTCATTGGGTTGTACGAAACCCAAAGGCGCAGTGAAAGCGAGGGTCGCCCCGGGCTGACCCAGGTGGGATCTTTCCGTCTCTTGGAGCGGGGAGCGCACCACCGGCCCGTCCCGTCCGCGTCGTCGGTGGGGCGGAGCAGGAGCGTGCACGCTGGGACCCGAAAGATGGTGAACTATGCCTGAGTAGAACGAAGCCAGAGNNN

>GenBankHQ168441_Aiteng_mysticus

AGGATTTCCCCAGTAACGGCGAGTGAAGCGGGAAAAGCCCAGCACCGAATCCCTCAGTGTCACGCTGACGGGAACTGTGGTGTGTGGGACGCCACCAGTCGCATTAGCGGGCGCCGAAGTCCTCCTGATCGAGGCTTCACCCAGAGCGGGTGTAAGGCCTTTGCTGGTGCCTCGCTGTGCGGCCGCGAGCGTCCTAGGAGTCGGGTTGTTTGGGAATGCAGCCCAAAGTGGGTGGTAAACTCCATCTAAGGCTAAATACTTGCACGAGTCCGATAGCGGACAAGTACCGTGAGGGAAAGTTGAAAAGAACTTTGAAGAGAGAGTTCAAGAGTACGTGAAACCGCTCAGAGGTAAACGGGTGGATCCGCAAAGTCGGCCTGTGGAATTCAGCGCGGCGAGCGGCTCGGGGCTGTTCCGGTCCGGGATCCTCGTGACCCGGCCGGGGCGCCGCGCCGGGCCCCGCCGCGTGCACTTTCCGCAGGCAGAGCGCCACGACCGGTTTCGCGGCGGGCAAAAGTCCGGTGGGAAGGTAGGCTGCTGCTCGCA----GCTGTTACAGCCCGCCGTGACGGCCCGCTCCGGGACCGAGGGACGGCCGCGCGCTTCGAGGCCTGGCTTTCCGGGGGAGTTCGACTGGCAGGGACTGGGCAACCGTGCCTGCCGACCGCTCCTCCGGACTGGCTCGGGTCGGCCGGGCGTGCTGAGGGTCAGTGGCGAGTCTGTCGGCACTCCACCCGACCCGTCTTGAAACACGGACCAAGGAGTCTAACATGCACGCGAGTCATTGGGCTGTACGAAACCCAAAGGCGCAGTGAAAGCGAGGGTCGCCTCGGGCTGACCCAGGTGGGATCCCTCCTTCCTTCGGGCGGGGGGGCGCACCACCGGCCCGTCCCGTCTGCGTCGGCAGTGGGGCGGAGCAGGAGCGTGTATGCTGGGACCCGAAAGATGGTGAACTATGCCTGAGTAGAACGAAGCCAGAGGAA

>ZSM20100356_Palliohedyle_sp

AGGATTCCCCCAGTAACGGCGAGTGAAGCGGGAAAAGCCCAGCACCGAATCCCTCAGTGTGACGCTGACGGGAACTGTGGTGTGTGGGACGCCACCAGTCGCATCAGCGGGCACCGAAGTTCTCCTGATCGAGGCTTCATCCATAGCGGGTGTAAGGCCTTTGC-GGTGCCTCGCTGTGCGGCCGCGAGCGTCTCAGGAGTCGGGTTGTTTGGGAATGCAGCCCAAAGCGGGTGGTAAACTCCATCTAAGGCTAAATACTTGCACGAGTCCGATAGCGGACAAGTACCGTGAGGGAAAGTTGAAAAGAACTTTGAAGAGAGAGTTCAAGAGTACGTGAAACCGCCCAGAGGTAAACGGGTGGATCCGCAAAGTCGGCCCGCGGAATTCAGCGCGGCGCGCGGCTCGGGGCTGCCTCGGCTCGGGATCCTCGGGACCCGGCCGGGGTGYCGCGCCGGGCTCCGCCGCGTGCACTTTCCGCGGGCAGAGCGCCACGACCGGTTCCGCGGCGGCTATAAGCCGGGCGGGAAGGTAGGCC----CATCGCGCG-GCTGTTACAGCCCGCCTTGGTGAGCCGCCGCGGGACCGAGGGACGGCCGCGCGCCTCGAGGCCTGGCCTTCCTGGGGAGTTCGACTGGCAGGGACTGGGCAACCGTGCCTGCCGACCGCTCCCCAGGACCGGACCGGGCGGGCCGGGCGCGCTTAGGGTCTGTGGCGAGTCTGTCGGCACTCCACCCGACCCGTCTTGAAACACGGACCAAGGAGTCTAACATGCGCGCGAGTCATTGGGCGGTACGAAACCCAAAGGCGCAGTGAAAGCGAGGGTCGCCTCGGGCTGACCCAGGTGGGATCCCTCTCCCTC----GGGAGGGGGCGCACCACCGGCCCGTCCCATCTGCGCCGTCAGTGGGGCGGAGCAGGAGCGTGCACGCTGGGACCCGAAAGATGGTGAACTATGCCTGAGTAGAACGAAGCCAGAGGAA

>ZSM20080063_Acochlidium_fijiense

AGGATTCCCCCAGTAACGGCGAGTGAAGCGGGAAAAGCCCAGCACCGAATCCCTCAGTGTCACGCTGACGGGAACTGTGGTGTGTGGGACGCCACCAGTCGCATCAGCAGGCACCGAAGTTCTCCTGATCGAGGCTTCACCCATAGCGGGTGTAAGGCCTTTGC-GGAGCCTCGCTGTGCGGCCGCGAGCGTCTCAGGAGTCGGGTTGTTTGGGAATGCAGCCCAAAGCGGGTGGTAAACTCCATCTAAGGCTAAATACTTGCACGAGTCCGATAGCGGACAAGTACCGTGAGGGAAAGTTGAAAAGAACTTTGAAGAGAGAGTTCAAGAGTACGTGAAACCGCCCAGAGGTAAACGGGTGGATCCGCAAAGTCGGCCCGCGGAATTCAGCGCGGCGCGCGGCTCGGGGCTGCCTCGGCTCGGGATC-CTCGGACCCGGCCGAGGTGCCGCGCCGGGCTCCGCCGCGTGCACTTTCCGCGGGCAGAGCGCCACGACCGGTTCCGCGGCGGCTACAAGCCGGGCGGGAAGGTAGGCC----CATTGCGCG-GCTGTTACAGCCCGCCTTGGTGAGCCGCCGCGGGACCGAGGGACGGCCGCGCGCTTCGAGGCCTGGCCTTCCCGGGGAGTTCGACTGGCAGGGACTGGGCAACCGTGCCTGCCGACCGCTCCTCGGGACCGGACCGGGCGGGCCGGGCGCGCTTAGGGTCTGTGGCGAGTCTGTCGGCACTCCACCCGACCCGTCTTGAAACACGGACCAAGGAGTCTAACATGCGCGCGAGTCATTGGGCTGTACGAAACCCAAAGGCGCAGTGAAAGCGAGGGTCGCCCCGGGCTGACCCAGGTGGGATCCCTCTCCCTC----GGGAGGGGGCGCACCACCGGCCCGTCCCATCTGCGCCGTCAGTGGGGCGGAGCAAGANCGTGCACGCTGGGACCCGAAAGATGGTGAACTATGCCTGAGTAGAACGAAGCCAGAGNAA

>ZSM20080393_Pseudunela_marteli

AGGATTCCCCCAGTAACGGCGAGTGAAGCGGGAAAAGCCCAGCACCGAATCCCTCAGTGTCATGCTGACGGGAACTGTGGTGTGTGGGACGCCACCAGTCGCATCAGCGGGCACCGAAGTTCTCCTGATCGAGGCTTCATCCATAGCGGGTGTAAGGCCTTTGC-GGTGCCTCGCTGTGCGGCCGCGAGCGTCTCAGGAGTCGGGTTGTTTGGGAATGCAGCCCAAAGCGGGTGGTAAACTCCATCTAAGGCTAAATACTTGCACGAGTCCGATAGCGGACAAGTACCGTGAGGGAAAGTTGAAAAGAACTTTGAAGAGAGAGTTCAAGAGTACGTGAAACCGCCCAGAGGTAAACGGGTGGATCCGCAAAGTCGGCCCGCGGAATTCAGCGCGGCGCGCGGCTCGGGGCTGCCCCGGCTTGGGATC-TTCGGACCCGGCCGGGGTGCCGCGCCGAGCTCCGCCGCGTGCACTTTCCGCGGGCAGAGCGCCACGACCGGTTTCGCGGCGGCTATAAGCCGGGCGGGAAGGTAGGCC----CATTGCGCG-GCTGTTACAGCCCGCCTTGGTGAGCCGCCGCGGGACCGAGGGACGGCCGCGCGCTTCGAGGCCTGGCCTTCCTGGGGAGTTCGACTGGCAGGGACTGGGCAACCGTGCCTGCCGACCGCTCTTCAGGACTGGACCGGGCGGGCCGGGCGCGCTTAGGGTCTGTGGCGAGTCTGTCGGCACTCCACCCGACCCGTCTTGAAACACGGACCAAGGAGTCTAACATGCGCGCGAGTCATTGGGCTGTACGAAACCCAAAGGCGCAGTGAAAGCGAGGGTCGCCTCGGGCTGACCCAGGTGGGATCCCTCTCCCTC----GGGAGGGGGCGCACCACCGGCCCGTCCCGTCTGCGCTGTCAGTGGGGCGGAGCAGGAGCGTGCACGCTGGGACCCGAAAGATGGTGAACTATGCCTGAGTAGAACGAAGCCAGAGGAA

>BerlinMoll193944_Strubellia_paradoxa

NGGATTCCCCCAGTAACGGCGAGTGAAGCGGGAAAAGCCCAGCACCGAATCCCTCAGTGTCATGCTGACGGGAACTGTGGTGTGTGGGACGCCACCAGTCGCATCAGCGGGCACCGAAGTTCTCCTGATCGAGGCTTCACCCATAGCGGGTGTAAGGCCTTTGC-GGTGCCTCGCTGTGCGGCCGCGAGCGTCTCAGGAGTCGGGTTGTTTGGGAATGCAGCCCAAAGCGGGTGGTAAACTCCATCTAAGGCTAAATACTTGCACGAGTCCGATAGCGGACAAGTACCGTGAGGGAAAGTTGAAAAGAACTTTGAAGAGAGAGTTCAAGAGTACGTGAAACCGCCCAGAGGTAAACGGGTGGATCCGCAAAGTCGGCCCGCGGAATTCAGCGCGGCGCGCGGCTCGGGGCTGCCTCGGCTCGGGATCCCTGGGACCCGGCCGGGGTGCCGCGCCGAGCTCCGCCGCGTGCACTTTCCGCGGGCAGAGCGCCACGACCGGTTTCGCGGCGGCTATAAGCCGGGCGGGAAGGTAGGCC----CATTGCGCG-GCTGTTACAGCCCGCCTTGGTGAGCCGCCGCGGGACCGAGGGACGGCCGCGCGCTTCGAGGCCTGGCCTTCCTGGGGAGTTCGACTGGCAGGGACTGGGCAACCGTGCCTGCCGACCGCTCCTCAGGACCGGACCGGGCGGGCCGGGCGCGCTGAGGGTCTGTGGCGAGTCTGTCGGCACTCCACCCGACCCGTCTTGAAACACGGACCAAGGAGTCTAACATGCGCGCGAGTCATTGGGCTGTACGAAACCCAAAGGCGCAGTGAAAGCGAGGGTCGCCTCGGGCTGACCCAGGTGGGATCCCTCTCTCTC----GGGAGGGGGCGCACCASCGGCCCGTCCCGTNNNNNNNNNNNNNNNNNNNNNNNNNNNNNNNNNNNNNNNNNNNNNNNNNNNNNNNNNNNNNNNNNNNNNNNNNNNNNNNNNNNNNNNN

>ZSM20090472

NNNNTTCCCCCAGTAACGGCGAGTGAAGCGGGACGAGCCCAGCACCGAATCCCTCAGTGTCACGCTGACGGGAACTGTGGTGTGTGGGACGCCACCAGTCGCATTAAAGGGCGCCGAAGTCCTCCTGATCGAGGCTTCACCCAGAGCGGGTGTAAGGCCAGTGCTGGTGCCTCTTTGTGCGGCCGCGAGCGTCTCAGGAGTCGGGTTGTTTGGGAATGCAGCCCAAAGCGGGTGGTAAACTCCATCTAAGGCTAAATACCGGCACGAGTCCGATAGCGGACAAGTACCGTGAGGGAAAGTTGAAAAGAACTTTGAAGAGAGAGTTCAAGAGTACGTGAAACCGCCCAGAGGTAAACGGGTGCATCCGCAAAGTCGGCCCGTGGAATTCAGCGCGGCGCGCGGCCTGGGGCTGCTCCGCTTCGGGATCCCTGGGACCCGGGCGGGGTGCTGCGCCGGGCTCCGCCGCGTGCACTTTCTGCGGGCAGAGCGCCACGACCGGTTTCGCGGCGGCNNNNNNNNNNNNNNNNNNNNNNNNNNNNNNNNNNNNNNNNNNNNNNNNNNNNNNNNNNNNNNNNNNNNNNNNNNNNNNNNNNNNNNNNNNNNNNNNNNNNNNNNNNNNNNNNNNNNNNNNNNNNNNNNNNNNNNNNNNNNNNNNNNNNNNNNNNNNNNNNNNNNNNNNNNNNNNNNNNNNNNNNNNNNNNNNNNNNNNNNNNNNNNNNNNNNNNNNNNNNNNNNNNNNNNNNNNNNNNNNNNNNNNNNNNNNNNNNNNNNNNNNNNNNNNNNNNNNNNNNNNNNNNNNNNNNNNNNNNNNNNNNNNNNNNNNNNNNNNNNNNNNNNNNNNNNNNNNNNNNNNNNNNNNNNNNNNNNNNNNNNNNNNNNNNNNNNNNNNNNNNNNNNNNNNNNNNNNNNNNNNNNNNNNNNNNNNNNNNNNNNNNNNNNNNNNNNNNNNNNNNNNNNNNNNNNNNNNNNNNNNNNNNNNNNNNN

>ZSM20110032

AGGATTCCCCCAGTAACGGCGAGTGAAGCGGGAAGAGCCCAGCACCGAATCCCTCAGTGTCATGCTGACGGGAACTGTGGTGTGTGGGACGCCACCAGTCGCACATGCGGGCGCCGAAGTCCTCCTGATCGAGGCTTCACCCAGAGCGGGTGTAAGGCCAGTGCTGGTGCTTCGCTGTGCGGCCGCGAGCGTCTCAGGAGTCGGGTTGTTTGGGAATGCAGCCCAAAGCGGGTGGTAAACTCCATCTAAGGCTAAATACCGGCACGAGTCCGATAGCGGACAAGTACCGTGAGGGAAAGTTGAAAAGAACTTTGAAGAGAGAGTTCAAGAGTACGTGAAACCGCCCAGAGGTAAACGGGTGCATCCGCAAAGTCGGCCCGTGGAATTCAGCGCGGCGCGCGGCCTGGGGCTGCTCCGTTTCGGGATCCCTGGGACCCGAGCGGGGTGCCGCGCCGGGCTCCGCCGCGTGCACTTTCTGCGGGCAGAGCGCCACGACCGGTTTCGCGGCGGCGACGAGCCGGGCGGGAAGGTAGGCGTCAGCTTCGGCCGCGCTGTTATAGACCGCCCTGGTGAGCTGCTGCGGGACCGAGGGACGGCCGCGTTCTTCGAGGCCTGGCTTTCTCGGGGAGTTCGACTGGCAGANNNNNNNNNNNNNNNNNNNNNNNNNNNNNNNNNNNNNNNNNNNNNNNNNNNNNNNNNNNNNNNNNNNNNNNNNNNNNNNNNNNNNNNNNNNNNNNNNNNNNNNNNNNNNNNNNNNNNNNNNNNNNNNNNNNNNNNNNNNNNNNNNNNNNNNNNNNNNNNNNNNNNNNNNNNNNNNNNNNNNNNNNNNNNNNNNNNNNNNNNNNNNNNNNNNNNNNNNNNNNNNNNNNNNNNNNNNNNNNNNNNNNNNNNNNNNNNNNNNNNNNNNNNNNNNNNNNNNNNNNNNNNNNNNNNNNNNNNNNNNNNNNNNNNNNNNNNNNNNNNNNNNNNNNNNNNNNNNNNNNN

>ZSM20071381

NNNNNNNNNNNNNNNNNNNNNNNNNNNNNNNNNNNNNNNNNNNNNNNNNNNNNNNNNNNNNNNNNNNNNNNNNNNNNNNNNNNNNNNNNNNNNNNNNNNNNNNNNNNNNNNNNNNNNNNNNNNNNNNNNNNNNNNNNNNNNNNNNNNNNNNNNNNNNNNNNNNNNNNNNNNNNNNNNNNNNNNNNNNNNNNNNNNNNNNNNNNNNNNNNNNNNNNNNNNNNNNNNNNNNNNNNNNNNNNNNNNNNNNNNNNNNNNNNNNNNNNNNNNNNNNNNNNNNNNNNNNNNNNNNNNNNNNNNNNNNNNNNNNNNNNNNNNNNNNNNNNNNNNNNNNNNNNNNNNNNNNNNNNNNNNNNNNNNNNNNNNNNNNNNNNNNNNNNNNNNNNNNNNNNNNNNNNNNNNNNNNNNNNNNNNNNNNNNNNNNNNNNNNNNNNNNNNNNNNNNNNNNNNNNNNNNNNNNNNNNNNNNNNNNNNNNNNNNNNNNNNNNNNNNNNNNNNNNNNNNNNNNNNNNNNNNNNNNNNNNNNNNNAAGGTAGGCGTCTCCTTCGGGCGCGCTGTTATAGACCGCCCTGGTGAGCTGCTGCGGGACCGAGGGACGGCCGCGTTCTTCGAGGCCAGGCTTTCTCGGGGAGTTCGACTGGCAGAGACTGGGCAACCGTGTCTGCCGACCGCTTTCCGGGACCGGACCGGGCTGGCCGGGAGCGCTGAGGGTCGGTGGCGAATCTGTCGGCATTGCACCCGACCCGTCTTGAAACACGGACCAAGGAGTCTAACATGCGCGCGAGTCATTGGGTTGTACGAAACCCAAAGGCGCAGTGAAAGCGAGGGTCGCCCCGGGCTGACCCAGGTGGGATCTTTCCGTTCTTCGGAGCGGGGAGCGCACCACCGGCCCGTCCCGTCCGCGTTGTCGGTGGGGCGGAGCAGGAGCGTGCACGCTGGGACCCGAAAGATGGTGAACTATGCCTGAGTAGAACGAAGCCAGAGGAA

>ZSM20110722

NNNNNNNNNNNNNNNNNNNNNNNNNNNNNNNNNNNNNNNNNNNNNNNNNNNNNNNNNNNNNNNNNNNNNNNNNNNNNNNNNNNNNNNNNNNNNNNNNNNNNNNNNNNNNNNNNNNNNNNNNNNNNNNNNNNNNNNNNNNNNNNNNNNNNNNNNNNNNNNNNNNNNNNNNNNNNNNNNNNNNNNNNNNNNNNNNNNNNNNNNNNNNNNNNNNNNNNNNNNNNNNNNNNNNNNNNNNNNNNNNNNNNNNNNNNNNNNNNNNNNNNNNNNNNNNNNNNNNNNNNNNNNNNNNNNNNNNNNNNNNNNNNNNNNNNNNNNNNNNNNNNNNNNNNNNNNNNNNNNNNNNNNNNNNNNNNNNNNNNNNCATCCGCAAAGTCGGCCCGTGGAATTCAGCGCGGCGCGCGGCCTGGAGCTGCTTCGTTTCGGGATTCCTGGGACCCGGGCGGGGTGCTGCGCCGGGCCCCGCCGCGTGCACTTTCTGCGGGCAGAGCGCCACGACCGGTTTCGCGGCGGCGACGAGCCGGGCGGGAAGGTAGGCGTCTCCTTCGGGCGCGCTGTT--TGACCGCCTCGGTGAGCTGCTGCGGGACCGAGGGACGGCCGCGTTCTTCGAGGCCAGGCTTTCTCGGGGAGTTCGACTGGCAGAGACTGAGCAATCGTGTCTGCCGACCGCTTCTCGA-GTCGGTCCGGGCTGGCCGGGAGCGCTCAGGGTCGGTGGCGAATCTGTCGGCATTGCACCCGACCCGTCTTGAAACACGGACCAAGGAGTCTAACATGCGCGCGAGTCATTGGGTTCTACGAAATCCAAAGGCGCAGTGAAAGCGAGGGTCGCCCCGGGCTGACCCAGGTGGGATCTTTCCGTCTCTCGGAGCGGGGAGC-CCCCACCGGCCCGTCCCGTCCGCGTCGTCGGTGGGGCGGAGCAGGAGCGTGCACGCTGGGACCCGAAAGATGGTGAACTATGCCTGAGTAGAACGAAGCCAGAGGAA

>ZSM20110723

NNNNNNNNNNNNNNNNNNNNNNNNNNNNNNNNNNNNNNNNNNNNNNNNNNNNNNNNNNNNNNNNNNNNNNNNNNNNNNNNNNNNNNNNNNNNNNNNNNNNNNNNNNNNNNNNNNNNNNNNNNNNNNNNNNNNNNNNNNNNNNNNNNNNNNNNNNNNNNNNNNNNNNNNNNNNNNNNNNNNNNNNNNNNNNNNNNNNNNNNNNNNNNNNNNNNNNNNNNNNNNNNNNNNNNNNNNNNNNNNNNNNNNNNNNNNNNNNNNNNNNNNNNAGTCCGATAGCGGACAAGTACCGTGAGGGAAAGTTGAAAAGAACTTTGAAGAGAGAGTTCAAGAGTACGTGAAACCGCCCAGAGGTAAACGGGTGCATCCGCAAAGTCGGCCCGTGGAATTCAGCGCGGCGCGCGGCCTGGAGCTGCTTCGTTTCGGGATCCCTGGGACCCGGGCGGGGTGCTGCGCCGGGCCCCGCCGCGTGCACTTTCTGCGGGCAGAGCGCCACGACCGGTTTCGCGGCGGCGACGAGCCGGGCGGGAAGGTAGGCGTCTCCTTCGGGCGCGCTGTTATAGACCGCCTCGGTGAGCTGCTGCGGGACCGAGGGACGGCCGCGTTCTTCGAGGCCAGGCTTTCTCGGGGAGTTCGACTGGCAGAGACTGAGCAATCGTGTCTGCCGACCGCTTCTCGA-GTCGGTCCGGGCTGGCCGGGAGCGCTCAGGGTCGGTGGCGAATCTGTCGGCATTGCACCCGACCCGTCTTGAAACACGGACCAAGGAGTCTAACATGCGCGCGAGTCATTGGGTTCTACGAAATCCAAAGGCGCAGTGAAAGCGAGGGTCGCCCCGGGCTGACCCAGGTGGGATCTTTCCGT-CTCCGGAGCGGGGAGC-CACCACCGGCCC-TCCCGTCCGCGTCGTCGGTGGGGCGGAGCAGGAGCGTGCACGCTGGGACCCGAAAGATGGTGAACTATGCCTGAGTAGAACGAAGCCAGAGGAA
