# Supplementary material for: How to describe a cryptic species? Practical challenges of molecular taxonomy
Source: Front Zool. 2013 Sep 27;10:59. doi: 10.1186/1742-9994-10-59 (PMC4015967; doi:10.1186/1742-9994-10-59)
Supplement: Additional file 3 — 18S rRNA alignment of Pontohedyle (fasta format). The alignment was generated with MUSCLE [107] and ambiguous parts of the alignment were masked with Gblocks [108] (settings for a less stringent selection). [file 1742-9994-10-59-S3.docx]

### Additional file 3 – 18S rRNA alignment of *Pontohedyle* (fasta format)

The alignment was generated with Muscle [[96](#_ENREF_96)] and ambiguous parts of the alignment were masked with Gblocks [[97](#_ENREF_97)] (settings for a less stringent selection).

>ZSM20080565

NNNNNNNAAAGATTAAGCCATGCATGTCTAAGTTCACACTATCTCACGGTGAAACCGCGAATGGCTCATTAAATCAGTCGAGGTTCCTTAGATGACACGATCCTACTTGGATAACTGTGGCAATTCTAGAGCTAATACATGCCTCTGAAGCTCCGACCTTCCTAGGGAAGAGCGCTTTTATTAGTTCAAAACCAATCGCACGCGTGCGCCCCATTTGGTGACTCTGGATAACTTTGTGCTGATCGCATGGCCTCCTGCGCCGGCGACGCATCTTTCAAATGTCTGCCCTATCAAATGTCGATGGTACGTGACATGCCTACCATGTTTGTAACGGGTAACGGGGAATCAGGGTTCGATTCCGGAGAGGGAGCATGAGAAACGGCTACCACATCCAAGGAAGGCAGCAGGCGCGCAACTTACCCACTCCCGGCACGGGGAGGTAGTGACGAAAAATAACAATACGGGACTCTTTCGAGGCCCCGTAATTGGAATGAGTACACTTTAAACCCTTTAACGAGGATCTATTGGAGGGCAAGTCTGGTGCCAGCAGCCGCGGTAATTCCAGCTCCAATAGCGTATATTAAAGTTGTTGCAGTTAAAAAGCTCGTAGTTGGATCTCAGGCGCAGGCGGGTGGTCCGGCTCGCGCCGGCTCACTGCCCGTACTCCTGCCCTACCTGTTGTCGGCTCTCTCCCGCGGGTGCTCTTCACTGAGCGTCCCGGGTGGCCGGCGCGTTTACTTTGAAAAAATTAGAGTGTTCAAAGCAGGCTTGGCTGCCTGAATAATGGTGCATGGAATAATGGAATAGGATCTCGGTTCTATTTTGTTGGTTTTCGGAACTAGAGGTAATGATTAACAGGGACAAACGGGGGCATTCGTATTGCGGCGTTAGAGGTGAAATTCTTGGATCGCCGCAAGACGAGCTACTGCGAAAGCATTTGTCAAGAATGTTTTCATTAATCAA???????????????????????????????????????????????????????????????????????????????????????????????????????????????????????????????????????????????????????????????????????????????????????????????????????AGCCTGCGGCTTAATTTGACTCAACACGGGAAAACTCACCCGGTCCGGACACTGTAAGGATTGACAGATTGATAGCTCTTTCTTGATTCGGTGGGTGGTGGTGCATGGCCGTTCTTAGTTGGTGGAGCGATTTGTCTGGTTAATTCCGATAACGAACGAGACTCTAGCCTATTAAATAGTTCGCCGATTCTTTGATGCGTCGGCGCAACTTCTTAGAGGGACGAGTGGCGTTTAGCCACACGAGATTGAGCAATAACAGGTCTGTGATGCCCTTAGATGTCCGGGGCCGCACGCGCGCTACACTGAAGGAATCAGCGTGGATGCCTCCCTGGTCCGAAAGGATTGGGAAACCCGTTGAATCTCCTTCGTGCTAGGGATTGGGGCTTGTAATTCTTCCCCATGAACGAGGAATTCCCAGTAAGCGCGAGTCATAAGCTCGCGTTGATTACGTCCCTGCCCTTTGTACACACCGCCCGTCGCTACTATCGATTGAGCGGTTCAGTGAGGGCCTCGGATTGGTCTCGGTCTGGCGTGCAAGCGCCGGCACCGTTGGCCGAGAAGACGCTCGAACTCGATCGCTTGGAGAAAGTAAAAGTCGTAACAAGGTTTCCGTAGGTG

>ZSM20090471

TTGTCTCAAAGATTAAGCCATGCATGTCTAAGCTCACACCATCTCACGGTGAAGCCGCGAATGGCTCATTAAATCAGTCGAGGTTCCTTAGATGACACGATCCTACTTGGATAACTTTGGCAATTCTATAGCTAATACATGCTTTCGATGCTCCGACCTGCAAAGGGAAGAGCGCTTTTATTAGTCCAAAACCAATCGCTCGCGTGCGCCCCCACTGGTGACTCTGGATAACTTTGTGCTGATCGCATGGCC-CCGGCGCCGGCGACGCATCTTTCAAATGTCTGCCCTATCAAATGTCGATGGTACGTGACATGCCTACCATGTTTGTAACGGGTAACGGGGAATCAGGGTTCGATTCCGGAGAGGGAGCATGAGAAACGGCTACCACATCCAAGGAAGGCAGCAGGCGCGCAACTTACCCACTCCCGGCACGGGGAGGTAGTGACGAAAAATAACAATACGGGACTCTTTCGAGGCCCCGTAATTGGAATGAGTACACTTTAAACCCTTTAACGAGGATCTATTGGAGGGCAAGTCTGGTGCCAGCAGCCGCGGTAATTCCAGCTCCAATAGCGTATATTAAAGTTGTTGCAGTTAAAAAGCTCGTAGTTGGATCTCAGGTGCAGGCGGGTGGTCCGGCTCGCGCCGGCTCACTGCCCGTTCTCCTGCCCTACCCGTTGTCGGCTCTCTCCCGCGGGTGCTCTTCGCTGAGCGTCCCGGGTGGCCGGCGCGTTTACTTTGAAAAAATTAGAGTGTTCAAAGCAGGCTCGGCTGCCTGAATAATGGTGCATGGAATAATGGAATAGGACCTCGGTTCTATTTTGTTGGTTTTCGGAACTGGAGGTAATGATTAACAGGGACAAACGGGGGCATTCGTATTGCGGCGTTAGAGGTGAAATTCTTGGATCGCCGCAAGACGAGCTACTGCGAAAGCATTTGTCAAGAATGTTTTCATTAGTCAAGAACGAAAGTCAGAGGCGCGAAGACGATCAGATACCGTCGTAGTTCTGACCATAAACGATGCCATCCAGCGATCCGCAGGAGTTGCTTCGATGACTCTGCGGGCAGCTTCCGGGAAACCAAAGTTTTTGGGTTCCGGGGGAAGTATGGTTGCAAAGCTGAAACTTAAAGGAATTGACGGAAGGGCACCACCAGGAGTGGAGCCTGCGGCTTAATTTGACTCAACACGGGAAAACTCACCCGGTCCGGACACTGTAAGGATTGACAGATTGATAGCTCTTTCTTGATTCGGTGGGTGGTGGTGCATGGCCGTTCTTAGTTGGTGGAGCGATTTGTCTGGTTAATTCCGATAACGAACGAGACTCTAGCCTATTAAATAGTTCGCCAATTCCTTGATGCGTTGGCGCAACTTCTTAGAGGGACGAGTGGCGTTTAGCCACACGAGATTGAGCAATAACAGGTCTGTGATGCCCTTAGATGTCCGGGGCCGCACGCGCGCTACACTGAAGGAATCAGCGTGGATGCCTCCCTGGTCCGAAAGGATTGGGAAACCCGTTGAATCTCCTTCGTGCTAGGGATTGGGGCTTGTAATTCTTCCCCATGAACGAGGAATTCCCAGTAAGCGCGAGTCATAAGCTCGCGTTGATTACGTCCCTGCCCTTTGTACACACCGCCCGTCGCTACTATCGATTGAGCGGTTCAGTGAGGGCCTCGGATTGGTCTCGGTCTGGCGTGCAAGCGCCGGCACCGTTGGCCGAGAAGACGCTCGAACTCGATCGCTTGGAGAAAGTAAAAGTCGTAACAAGGTTTCCGTAGGTG

>ZSM20081013

NNNNNNNNNNNNNNNNNCCATGCATGTCTAAGTTCACACTATCTCACGGTGAAACCGCGAATGGCTCATTAAATCAGTCGAGGTTCCTTAGATGACACGATCCTACTTGGATAACTGTGGCAATTCTAGAGCTAATACATGCTTTTGAAGCTCCGACCTGCAAAGGGAAGAGCGCTTTTATTAGTTCAAAACCAATCGGTCC----CGCCCCCTTTGGTGACTCTGGATAACTTTGTGCTGATCGCATGGCC-TTTGCGCCGGCGACGCATCTTTCAAATGTCTGCCCTATCAAATGTCGATGGTACGTGACATGCCTACCATGTTTGTAACGGGTAACGGGGAATCAGGGTTCGATTCCGGAGAGGGAGCATGAGAAACGGCTACCACATCCAAGGAAGGCAGCAGGCGCGCAACTTACCCACTCCCGGCACGGGGAGGTAGTGACGAAAAATAACAATACGGGACTCTTTCGAGGCCCCGTAATTGGAATGAGTACACTTTAAACCCTTTAACGAGGATCTATTGGAGGGCAAGTCTGGTGCCAGCAGCCGCGGTAATTCCAGCTCCAATAGCGTATATTAAAGTTGTTGCAGTTAAAAAGCTCGTAGTTGGATCTCAGGCGCAGGCGGGTGGTCCGGCTCGCGCCGGCTCACTGCCCGTACTCCTGCCCTACCTGTTGTCGGCTCTCTCCCGCGGGTGCTCTTTACTGAGCGTCCCGGGTGGCCGGCGCGTTTACTTTGAAAAAATTAGAGTGTTCAAAGCAGGCTCGATAGCCTGAATAATGGTGCATGGAATAATGGAATAGGACCTCGGTTCTATTTTGTTGGTTTTCGGAACTAGAGGTAATGATTAACAGGGACAAACGGGGGCATTCGTATTGCGGCGTTAGAGGTGAAATTCTTGGATCGCCGCAAGACGAGCTACTGCGAAAGCATTTGTCAAGAATGTTTTCATTAATCAAGAACGAAAGTCAGAGGCGCGAAGACGATCAGATACCGTCGTAGTTCTGACCATAAACGATGCCACCTAGCGATCCGCAGGAGTTGCTTCGATGACTCTGCGGGCAGCTTCCGGGAAACCAAAGGTTTTGGGTTCCGGGGGAAGTATGGTTGCAAAGCTGAAACTTAAAGGAATTGACGGAAGGGCACCACCAGGAGTGGAGCCTGCGGCTTAATTTGACTCAACACGGGAAAACTCACCCGGTCCGGACACTGTAAGGATTGACAGATTGATAGCTCTTTCTTGATTCGGTGGGTGGTGGTGCATGGCCGTTCTTAGTTGGTGGAGCGATTTGTCTGGTTAATTCCGATAACGAACGAGACTCTAGCCTATTAAATAGTTCGCCGATTCCTACATGCGTCGGCGCAACTTCTTAGAGGGACGAGTGGCGTTTAGCCACACGAGATTGAGCAATAACAGGTCTGTGATGCCCTTAGATGTCCGGGGCCGCACGCGCGCTACACTGAAGGAATCAGCGTGGATGCCTCCCTGGTCCGAAAGGATTGGGAAACCCGTTGAATCTCCTTCGTGCTAGGGATTGGGGCTTGTAATTCTTCCCCATGAACGAGGAATTCCCAGTAAGCGCGAGTCATAAGCTCGCGTTGATTACGTCCCTGCCCTTTGTACACACCGCCCGTCGCTACTATCGATTGAGCGGTTCAGTGAGGGCCTCGGATTGGTCTCGGTCTGGCGTGCAAGCGCCGGCACCGTTGGCCGAGAAGACGCTCGAACTCGATCGCTTGGAGAAAGTAAAAGTCGTAACAAGGTTTCNNNNNNNN

>SICBC2010KJ01D05

TTGTCTCAAAGATTAAGCCATGCATGTCTAAGTTCACACTATCTCACGGTGAAACCGCGAATGGCTCATTAAATCAGTCGAGGTTCCTTAGATGACACGATCCTACTTGGATAACTGTGGCAATTCTAGAGCTAATACATGCTTTTGAAGCTCCGACCTGCAAAGGGAAGAGCGCTTTTATTAGTTCAAAACCAATCGCTCGCGGGTGTCCCCTTTGGTGACTCTGGATAACTTTGTGCTGATCGCATGGCC-CTAGCGCCGGCGACGCATCTTTCAAATGTCTGCCCTATCAAATGTCGATGGTACGTGACATGCCTACCATGTTTGTAACGGGTAACGGGGAATCAGGGTTCGATTCCGGAGAGGGAGCATGAGAAACGGCTACCACATCCAAGGAAGGCAGCAGGCGCGCAACTTACCCACTCCCGGCACGGGGAGGTAGTGACGAAAAATAACAATACGGGACTCTTTCGAGGCCCCGTAATTGGAATGAGTACACTTTAAACCCTTTAACGAGGATCTATTGGAGGGCAAGTCTGGTGCCAGCAGCCGCGGTAATTCCAGCTCCAATAGCGTATATTAAAGTTGTTGCAGTTAAAAAGCTCGTAGTTGGATCTCAGGCGCAGGCGGGTGGTCCGGCTCGCGCCGGCTCACTGCCCGTACTCCTGCCCTACCTGTTGTCGGCTCTCTCCCGCGGGTGCTCTTCACTGAGCGTCCCGGGTGGCCGGCGCGTTTACTTTGAAAAAATTAGAGTGTTCAAAGCAGGCTAAATAGCCTGAATAATGGTGCATGGAATAATGGAATAGGACCTCGGTTCTATTTTGTTGGTTTTCGGAACTAGAGGTAATGATTAACAGGGACAAACGGGGGCATTCGTATTGCGGCGTTAGAGGTGAAATTCTTGGATCGCCGCAAGACGAGCTACTGCGAAAGCATTTGTCAAGAATGTTTTCATTAATCAAGAACGAAAGTCAGAGGCGCGAAGACGATCAGATACCGTCGTAGTTCTGACCATAAACGATGCCACCTAGCGATCCGCAGGAGTTGCTTCGATGACTCTGCGGGCAGCTTCCGGGAAACCAAAGGTTTTGGGTTCCGGGGGAAGTATGGTTGCAAAGCTGAAACTTAAAGGAATTGACGGAAGGGCACCACCAGGAGTGGAGCCTGCGGCTTAATTTGACTCAACACGGGAAAACTCACCCGGTCCGGACACTGTAAGGATTGACAGATTGATAGCTCTTTCTTGATTCGGTGGGTGGTGGTGCATGGCCGTTCTTAGTTGGTGGAGCGATTTGTCTGGTTAATTCCGATAACGAACGAGACTCTAGCCTATTAAATAGTTCGCCGATTCCTTGATGCGTCGGCGCAACTTCTTAGAGGGACGAGTGGCGTTTAGCCACACGAGATTGAGCAATAACAGGTCTGTGATGCCCTTAGATGTCCGGGGCCGCACGCGCGCTACACTGAAGGAATCAGCGTGGATGCCTCCCTGGTCCGAAAGGATTGGGAAACCCGTTGAATCTCCTTCGTGCTAGGGATTGGGGCTTGTAATTCTTCCCCATGAACGAGGAATTCCCAGTAAGCGCGAGTCATAAGCTCGCGTTGATTACGTCCCTGCCCTTTGTACACACCGCCCGTCGCTACTATCGATTGAGCGGTTCAGTGAGGGCCTCGGATTGGTCTCGGTCTGGCGTGCAAGCGCCGGCACCGTTGGCCGAGAAGACGCTCGAACTCGATCGCTTGGAGAAAGTAAAAGTCGTAACAAGGTTTCCGTAGGTG

>ZSM20090197

TTGTCTCAAAGATTAAGCCATGCATGTCTAAGTTCACACTATCTCACGGTGAAACCGCGAATGGCTCATTAAATCAGTCGAGGTTCCTTAGATGACACGATCCTACTTGGATAACTGTGGCAATTCTAGAGCTAATACATGCTTTTGAAGCTCCGACCTGCAAAGGGAAGAGCGCTTTTATTAGTTCAAAACCAATCGCTCGCGGGTGTCCCCTTTGGTGACTCTGGATAACTTTGTGCTGATCGCATGGCC-CTAGCGCCGGCGACGCATCTTTCAAATGTCTGCCCTATCAAATGTCGATGGTACGTGACATGCCTACCATGTTTGTAACGGGTAACGGGGAATCAGGGTTCGATTCCGGAGAGGGAGCATGAGAAACGGCTACCACATCCAAGGAAGGCAGCAGGCGCGCAACTTACCCACTCCCGGCACGGGGAGGTAGTGACGAAAAATAACAATACGGGACTCTTTCGAGGCCCCGTAATTGGAATGAGTACACTTTAAACCCTTTAACGAGGATCTATTGGAGGGCAAGTCTGGTGCCAGCAGCCGCGGTAATTCCAGCTCCAATAGCGTATATTAAAGTTGTTGCAGTTAAAAAGCTCGTAGTTGGATCTCAGGCGCAGGCGGGTGGTCCGGCTCGCGCCGGCTCACTGCCCGTACTCCTGCCCTACCTGTTGTCGGCTCTCTCCCGCGGGTGCTCTTCACTGAGCGTCCCGGGTGGCCGGCGCGTTTACTTTGAAAAAATTAGAGTGTTCAAAGCAGGCTAAATAGCCTGAATAATGGTGCATGGAATAATGGAATAGGACCTCGGTTCTATTTTGTTGGTTTTCGGAACTAGAGGTAATGATTAACAGGGACAAACGGGGGCATTCGTATTGCGGCGTTAGAGGTGAAATTCTTGGATCGCCGCAAGACGAGCTACTGCGAAAGCATTTGTCAAGAATGTTTTCATTAATCAAGAACGAAAGTCAGAGGCGCGAAGACGATCAGATACCGTCGTAGTTCTGACCATAAACGATGCCACCTAGCGATCCGCAGGAGTTGCTTCGATGACTCTGCGGGCAGCTTCCGGGAAACCAAAGGTTTTGGGTTCCGGGGGAAGTATGGTTGCAAAGCTGAAACTTAAAGGAATTGACGGAAGGGCACCACCAGGAGTGGAGCCTGCGGCTTAATTTGACTCAACACGGGAAAACTCACCCGGTCCGGACACTGTAAGGATTGACAGATTGATAGCTCTTTCTTGATTCGGTGGGTGGTGGTGCATGGCCGTTCTTAGTTGGTGGAGCGATTTGTCTGGTTAATTCCGATAACGAACGAGACTCTAGCCTATTAAATAGTTCGCCGATTCCTTGATGCGTCGGCGCAACTTCTTAGAGGGACGAGTGGCGTTTAGCCACACGAGATTGAGCAATAACAGGTCTGTGATGCCCTTAGATGTCCGGGGCCGCACGCGCGCTACACTGAAGGAATCAGCGTGGATGCCTCCCTGGTCCGAAAGGATTGGGAAACCCGTTGAATCTCCTTCGTGCTAGGGATTGGGGCTTGTAATTCTTCCCCATGAACGAGGAATTCCCAGTAAGCGCGAGTCATAAGCTCGCGTTGATTACGTCCCTGCCCTTTGTACACACCGCCCGTCGCTACTATCGATTGAGCGGTTCAGTGAGGGCCTCGGATTGGTCTCGGTCTGGCGTGCAAGCGCCGGCACCGTTGGCCGAGAAGACGCTCGAACTCGATCGCTTGGAGAAAGTAAAAGTCGTAACAAGGTTTCCGTAGGTG

>ZSM20071820

TTGTCTCAAAGATTAAGCCATGCATGTCTAAGTTCACACTATCTCACGGTGAAACCGCGAATGGCTCATTAAATCAGTCGAGGTTCCTTAGATGACACGATCCTACTTGGATAACTGTGGCAATTCTAGAGCTAATACATGCTTTTGAAGCTCCGACCTGCAAAGGGAAGAGCGCTTTTATTAGTTCAAAACCAATCGCTCGCGGGCGCCCACTTTGGTGACTCTGGATAACTTTGTGCTGATCGCATGGCC-CTGGCGCCGGCGACGCATCTTTCAAATGTCTGCCCTATCAAATGTCGATGGTACGTGACATGCCTACCATGTTTGTAACGGGTAACGGGGAATCAGGGTTCGATTCCGGAGAGGGAGCATGAGAAACGGCTACCACATCCAAGGAAGGCAGCAGGCGCGCAACTTACCCACTCCCGGCACGGGGAGGTAGTGACGAAAAATAACAATACGGGACTCTTTCGAGGCCCCGTAATTGGAATGAGTACACTTTAAACCCTTTAACGAGGATCTATTGGAGGGCAAGTCTGGTGCCAGCAGCCGCGGTAATTCCAGCTCCAATAGCGTATATTAAAGTTGTTGCAGTTAAAAAGCTCGTAGTTGGATCTCAGGCGCAGGCGGGTGGTCCGGCTCGCGCCGGCTCACTGCCCGTACTCCTGCCCTACCTGTTGTCGGCTCTCTCCCGCGGGTGCTCTTCACTGAGCGTCCCGGGTGGCCGGCGCGTTTACTTTGAAAAAATTAGAGTGTTCAAAGCAGGCTAAATAGCCTGAATAATGGTGCATGGAATAATGGAATAGGACCTCGGTTCTATTTTGTTGGTTTTCGGAACTAGAGGTAATGATTAACAGGGACAAACGGGGGCATTCGTATTGCTGCGTTAGAGGTGAAATTCTTGGATCGCAGCAAGACGAGCTACTGCGAAAGCATTTGTCAAGAATGTTTTCATTAATCAAGAACGAAAGTCAGAGGCGCGAAGACGATCAGATACCGTCGTAGTTCTGACCATAAACGATGCCACCTAGCGATCCGCAGGAGTTGCTTCGATGACTCTGCGGGCAGCTTCCGGGAAACCAAAGGTTTTGGGTTCCGGGGGAAGTATGGTTGCAAAGCTGAAACTTAAAGGAATTGACGGAAGGGCACCACCAGGAGTGGAGCCTGCGGCTTAATTTGACTCAACACGGGAAAACTCACCCGGTCCGGACACTGTAAGGATTGACAGATTGATAGCTCTTTCTTGATTCGGTGGGTGGTGGTGCATGGCCGTTCTTAGTTGGTGGAGCGATTTGTCTGGTTAATTCCGATAACGAACGAGACTCTAGCCTATTAAATAGTTCGCCGATTCTTTGATGCGTCGGCGCAACTTCTTAGAGGGACGAGTGGCGTTTAGCCACACGAGATTGAGCAATAACAGGTCTGTGATGCCCTTAGATGTCCGGGGCCGCACGCGCGCTACACTGAAGGAATCAGCGTGGATGCCTCCCTGGTCCGAAAGGATTGGGAAACCCGTTGAATCTCCTTCGTGCTAGGGATTGGGGCTTGTAATTCTTCCCCATGAACGAGGAATTCCCAGTAAGCGCGAGTCATAAGCTCGCGTTGATTACGTCCCTGCCCTTTGTACACACCGCCCGTCGCTACTATCGATTGAGCGGTTCAGTGAGGGCCTCGGATTGGTCTCGGTCTGGCGTGCAAGCGCCGGCACCGTTGGCCGAGAAGACGCTCGAACTCGATCGCTTGGAGAAAGTAAAANNNNNNNNNNNNNNNNNNNNNNNN

>ZSM20071135

NNNNNTCAAAGATTAAGCCATGCATGTCTAAGTTCACACTATCTCACGGTGAAACCGCGAATGGCTCATTAAATCAGTCGAGGTTCCTTAGATGACACGATCCTACTTGGATAACTGTGGCAATTCTAGAGCTAATACATGCTTTTGAAGCTCCGACCTGCAAAGGGAAGAGCGCTTTTATTAGTTCAAAACCAATCGCTCGCGGGCGCCCACTTTGGTGACTCTGGATAACTTTGTGCTGATCGCATGGCC-CTGGCGCCGGCGACGCATCTTTCAAATGTCTGCCCTATCAAATGTCGATGGTACGTGACATGCCTACCATGTTTGTAACGGGTAACGGGGAATCAGGGTTCGATTCCGGAGAGGGAGCATGAGAAACGGCTACCACATCCAAGGAAGGCAGCAGGCGCGCAACTTACCCACTCCCGGCACGGGGAGGTAGTGACGAAAAATAACAATACGGGACTCTTTCGAGGCCCCGTAATTGGAATGAGTACACTTTAAACCCTTTAACGAGGATCTATTGGAGGGCAAGTCTGGTGCCAGCAGCCGCGGTAATTCCAGCTCCAATAGCGTATATTAAAGTTGTTGCAGTTAAAAAGCTCGTAGTTGGATCTCAGGCGCAGGCGGGTGGTCCGGCTCGCGCCGGCTCACTGCCCGTACTCCTGCCCTACCTGTTGTCGGCTCTCTCCCGCGGGTGCTCTTCACTGAGCGTCCCGGGTGGCCGGCGCGTTTACTTTGAAAAAATTAGAGTGTTCAAAGCAGGCTAAATAGCCTGAATAATGGTGCATGGAATAATGGAATAGGACCTCGGTTCTATTTTGTTGGTTTTCGGAACTAGAGGTAATGATTAACAGGGACAAACGGGGGCATTCGTATTGCTGCGTTAGAGGTGAAATTCTTGGATCGCAGCAAGACGAGCTACTGCGAAAGCATTTGTCAAGAATGTTTTCATTAATCAAGAACGAAAGTCAGAGGCGCGAAGACGATCAGATACCGTCGTAGTTCTGACCATAAACGATGCCACCTAGCGATCCGCAGGAGTTGCTTCGATGACTCTGCGGGCAGCTTCCGGGAAACCAAAGGTTTTGGGTTCCGGGGGAAGTATGGTTGCAAAGCTGAAACTTAAAGGAATTGACGGAAGGGCACCACCAGGAGTGGAGCCTGCGGCTTAATTTGACTCAACACGGGAAAACTCACCCGGTCCGGACACTGTAAGGATTGACAGATTGATAGCTCTTTCTTGATTCGGTGGGTGGTGGTGCATGGCCGTTCTTAGTTGGTGGAGCGATTTGTCTGGTTAATTCCGATAACGAACGAGACTCTAGCCTATTAAATAGTTCGCCGATTCTTTGATGCGTCGGCGCAACTTCTTAGAGGGACGAGTGGCGTTTAGCCACACGAGATTGAGCAATAACAGGTCTGTGATGCCCTTAGATGTCCGGGGCCGCACGCGCGCTACACTGAAGGAATCAGCGTGGATGCCTCCCTGGTCCGAAAGGATTGGGAAACCCGTTGAATCTCCTTCGTGCTAGGGATTGGGGCTTGTAATTCTTCCCCATGAACGAGGAATTCCCAGTAAGCGCGAGTCATAAGCTCGCGTTGATTACGTCCCTGCCCTTTGTACACACCGCCCGTCGCTACTATCGATTGAGCGGTTCAGTGAGGGCCTCGGATTGGTCTCGGTCTGGCGTGCAAGCGCCGGCACCGTTGGCCGAGAAGACGCTCGAACTCGATCGCTTGGAGAAAGTAAAAGTCGTAACAAGGTTTCCGTAGGTG

>ZSM20100391

TTGTCTCAAAGATTAAGCCATGCATGTCTAAGTTCACACTATCTCACGGTGAAACCGCGAATGGCTCATTAAATCAGTCGAGGTTCCTTAGATGACACGATCCTACTTGGATAACTGTGGCAATTCTAGAGCTAATACATGCTTTTGAAGCTCCGACCTGCAAAGGGAAGAGCGCTTTTATTAGTTCAAAACCAATCGCTCGCGGGCGCCCACTTTGGTGACTCTGGATAACTTTGTGCTGATCGCATGGCC-CTGGCGCCGGCGACGCATCTTTCAAATGTCTGCCCTATCAAATGTCGATGGTACGTGACATGCCTACCATGTTTGTAACGGGTAACGGGGAATCAGGGTTCGATTCCGGAGAGGGAGCATGAGAAACGGCTACCACATCCAAGGAAGGCAGCAGGCGCGCAACTTACCCACTCCCGGCACGGGGAGGTAGTGACGAAAAATAACAATACGGGACTCTTTCGAGGCCCCGTAATTGGAATGAGTACACTTTAAACCCTTTAACGAGGATCTATTGGAGGGCAAGTCTGGTGCCAGCAGCCGCGGTAATTCCAGCTCCAATAGCGTATATTAAAGTTGTTGCAGTTAAAAAGCTCGTAGTTGGATCTCAGGCGCAGGCGGGTGGTCCGGCTCGCGCCGGCTCACTGCCCGTACTCCTGCCCTACCTGTTGTCGGCTCTCTCCCGCGGGTGCTCTTCACTGAGCGTCCCGGGTGGCCGGCGCGTTTACTTTGAAAAAATTAGAGTGTTCAAAGCAGGCTAAATAGCCTGAATAATGGTGCATGGAATAATGGAATAGGACCTCGGTTCTATTTTGTTGGTTTTCGGAACTAGAGGTAATGATTAACAGGGACAAACGGGGGCATTCGTATTGCTGCGTTAGAGGTGAAATTCTTGGATCGCAGCAAGACGAGCTACTGCGAAAGCATTTGTCAAGAATGTTTTCATTAATCAAGAACGAAAGTCAGAGGCGCGAAGACGATCAGATACCGTCGTAGTTCTGACCATAAACGATGCCACCTAGCGATCCGCAGGAGTTGCTTCGATGACTCTGCGGGCAGCTTCCGGGAAACCAAAGGTTTTGGGTTCCGGGGGAAGTATGGTTGCAAAGCTGAAACTTAAAGGAATTGACGGAAGGGCACCACCAGGAGTGGAGCCTGCGGCTTAATTTGACTCAACACGGGAAAACTCACCCGGTCCGGACACTGTAAGGATTGACAGATTGATAGCTCTTTCTTGATTCGGTGGGTGGTGGTGCATGGCCGTTCTTAGTTGGTGGAGCGATTTGTCTGGTTAATTCCGATAACGAACGAGACTCTAGCCTATTAAATAGTTCGCCGATTCTTTGATGCGTCGGCGCAACTTCTTAGAGGGACGAGTGGCGTTTAGCCACACGAGATTGAGCAATAACAGGTCTGTGATGCCCTTAGATGTCCGGGGCCGCACGCGCGCTACACTGAAGGAATCAGCGTGGATGCCTCCCTGGTCCGAAAGGATTGGGAAACCCGTTGAATCTCCTTCGTGCTAGGGATTGGGGCTTGTAATTCTTCCCCATGAACGAGGAATTCCCAGTAAGCGCGAGTCATAAGCTCGCGTTGATTACGTCCCTGCCCTTTGTACACACCGCCCGTCGCTACTATCGATTGAGCGGTTCAGTGAGGGCCTCGGATTGGTCTCGGTCTGGCGTGCAAGCGCCGGCACCGTTGGCCGAGAAGACGCTCGAACTCGATCGCTTGGAGAAAGTAAAAGTCGTAACAAGGTTTCCGTAGGTG

>ZSM20100592

TTGTCTCAAAGATTAAGCCATGCATGTCTAAGTTCACACTATCTCACGGTGAAACCGCGAATGGCTCATTAAATCAGTCGAGGTTCCTTAGATGACACGATCCTACTTGGATAACTGTGGCAATTCTAGAGCTAATACATGTCTCTGAAGCTCCGACCTTTGCAGGGAAGAGCGCTTTTATTAGTTCAAAACCAATCGTGCGCGCACGCCCACTGTGGTGACTCAGAATAACTTTGTGCTGATCGCATGGCCTCCTGCGCCGGCGACGCATCTTTCAAATGTCTGCCCTATCAAATGTCGATGGTACGTGACATGCCTACCATGTTTGTAACGGGTAACGGGGAATCAGGGTTCGATTCCGGAGAGGGAGCATGAGAAACGGCTACCACATCCAAGGAAGGCAGCAGGCGCGCAACTTACCCACTCCCGGCACGGGGAGGTAGTGACGAAAAATAACAATACGGGACTCTTTCGAGGCCCCGTAATTGGAATGAGTACACTTTAAACCCTTTAACGAGGATCTATTGGAGGGCAAGTCTGGTGCCAGCAGCCGCGGTAATTCCAGCTCCAATAGCGTATATTAAAGTTGTTGCAGTTAAAAAGCTCGTAGTTGGATCTGAGGCGCAGGCGGGTGGTCCGGCTCGCGCCGGCTCACTGCCCGTACTCCTGCCCTACCTGTTGTCGGCTCTCTCCCGCGGGTGCTCTTCGCTGAGCGTCCCGGGTGGCCGGCGCGTTTACTTTGAAAAAATTAGAGTGTTCAAAGCAGGCTATTCAGCCTGAATAATGGTGCATGGAATAATGGAATAGGACCTCGGTTCTATTTTGTTGGTTTTCGGAACTAGAGGTAATGATTAACAGGGACGAACGGGGGCATTCGTATTGCTGCGTTAGAGGTGAAATTCTTGGATCGCAGCAAGACGAGCTACTGCGAAAGCATTTGTCAAGAATGTTTTCATTAATCAAGAACGAAAGTCAGAGGCGCGAAGACGATCAGATACCGTCGTAGTTCTGACCATAATCGATGCCAGCTAGCAATCCGCAGGAGTTGCTTCGATGACTCTGCGGGCAGCTTCCGGGAAACCAAAGTTTTTGGGTTCCGGGGGAAGTATGGTTGCAAAGCTGAAACTTAAAGGAATTGACGGAAGGGCACCACCAGGAGTGGAGCCTGCGGCTTAATTTGACTCAACACGGGAAAACTCACCCGGTCCGGACACTGTAAGGATTGACAGATTGATAGCTCTTTCTTGATTCGGTGGGTGGTGGTGCATGGCCGTTCTTAGTTGGTGGAGCGATTTGTCTGGTTAATTCCGATAACGAACGAGACTCTAGCCTATTAAATAGTTCGCCGATTCCTTGATGCGTCGGCGCAACTTCTTAGAGGGACGCGTGGCGTTTAGCCACACGAGATTGAGCAATAACAGGTCTGTGATGCCCTTAGATGTCCGGGGCCGCACGCGCGCTACACTGAAGGAATCAGCGTGGATGCCTCCCTGGTCCGAAAGGATTGGGAAACCCGTTGAATCTCCTTCGTGCTAGGGATTGGGGCTTGTAATTCTTCCCCATGAACGAGGAATTCCCAGTAAGCGCGAGTCATAAGCTCGCGTTGATTACGTCCCTGCCCTTTGTACACACCGCCCGTCGCTACTATCGATTGAGCGGTTCAGTGAGGGCCTCGGATTGGTCTCGGTCTGGCGTGCAAGCGCCGGCACCGTTGGCCGAGAAGACGCTCGAACTCGATCGCTNNNNNNNNNNNNNNNNNNNNNNNNNNNNNNNNNNNNNN

>ZSM20110723

TTGTCTCAAAGATTAAGCCATGCATGTCTAAGTTCACACTATCTCACGGTGAAACCGCGAATGGCTCATTAAATCAGTCGAGGTTCCTTAGATGACACGATCCTACTTGGATAACTGTGGCAATTCTAGAGCTAATACATGCCTCTGAAGCTCCGACCTTTGCTGGGAAGAGCGCTTTTATTAGTTCAAAACCAATCGTGCGCGCACGCCCCGTTTGGTGACTCTGGATAACTTTGTGCTGATCGCATGGCCTCCTGCGCCGGCGACGCATCTTTCAAATGTCTGCCCTATCAAATGTCGATGGTACGTGACATGCCTACCATGTTTGTAACGGGTAACGGGGAATCAGGGTTCGATTCCGGAGAGGGAGCATGAGAAACGGCTACCACATCCAAGGAAGGCAGCAGGCGCGCAACTTACCCACTCCCGGCACGGGGAGGTAGTGACGAAAAATAACAATACGGGACTCTTTCGAGGCCCCGTAATTGGAATGAGTACACTTTAAACCCTTTAACGAGGATCTATTGGAGGGCAAGTCTGGTGCCAGCAGCCGCGGTAATTCCAGCTCCAATAGCGTATATTAAAGTTGTTGCAGTTAAAAAGCTCGTAGTTGGATCTCAGGCGCAGGCGGGTGGTCCGGCTCGCGCCGGCTCACTGCCCGTACTCCTGCCCTACCTGTTGTCGGCTCTCTCCCGCGGGTGCTCTTCGCTGAGCGTCCCGGGTGGCCGGCGCGTTTACTTTGAAAAAATTAGAGTGTTCAAAGCAGGCTCGGCTGCCTGAATAATGGTGCATGGAATAATGGAATAGGACCTCGGTTCTATTTTGTTGGTTTTCGGAACTAGAGGTAATGATTAACAGGGACAAACGGGGGCATTCGTATTGCTGCGTTAGAGGTGAAATTCTTGGATCGCAGCAAGACGAGCTACTGCGAAAGCATTTGTCAAGAATGTTTTCATTAATCAAGAACGAAAGTCAGAGGCGCGAAGACGATCAGATACCGTCGTAGTTCTGACCATAAACGATGCCAGCTAGCGATCCGCAGGAGTTGCTTCGATGACTCTGCGGGCAGCTTCCGGGAAACCAAAGTTTTTGGGTTCCGGGGGAAGTATGGTTGCAAAGCTGAAACTTAAAGGAATTGACGGAAGGGCACCACCAGGAGTGGAGCCTGCGGCTTAATTTGACTCAACACGGGAAAACTCACCCGGTCCGGACACTGTAAGGATTGACAGATTGATAGCTCTTTCTTGATTCGGTGGGTGGTGGTGCATGGCCGTTCTTAGTTGGTGGAGCGATTTGTCTGGTTAATTCCGATAACGAACGAGACTCTAGCCTATTAAATAGTTCGCCGATTCCTTGATGCGTCGGCGCAACTTCTTAGAGGGACGAGTGGCGTTTAGCCACACGAGATTGAGCAATAACAGGTCTGTGATGCCCTTAGATGTCCGGGGCCGCACGCGCGCTACACTGAAGGAATCAGCGTGGATGCCTCCCTGGTCCGAAAGGATTGGGAAACCCGTTGAATCTCCTTCGTGCTAGGGATTGGGGCTTGTAATTCTTCCCCATGAACGAGGAATTCCCAGTAAGCGCGAGTCATAAGCTCGCGTTGATTACGTCCCTGCCCTTTGTACACACCGCCCGTCGCTACTATCGATTGAGCGGTTCAGTGAGGGCCTCGGATTGGTCTCGGTCTGGTGTGCAAGCGCCGGCACCGTTGGCCGAGAAGACGCTCGAACTCGATCGCTTGGAGAAAGTAAAAGTCGTAACAAGGTTTCCGTAGGTG

>ZSM20110722

TTGTCTCAAAGATTAAGCCATGCATGTCTAAGTTCACACTATCTCACGGTGAAACCGCGAATGGCTCATTAAATCAGTCGAGGTTCCTTAGATGACACGATCCTACTTGGATAACTGTGGCAATTCTAGAGCTAATACATGCCTCTGAAGCTCCGACCTTTGCTGGGAAGAGCGCTTTTATTAGTTCAAAACCAATCGTGCGCGCACGCCCCGTTTGGTGACTCTGGATAACTTTGTGCTGATCGCATGGCCTCCTGCGCCGGCGACGCATCTTTCAAATGTCTGCCCTATCAAATGTCGATGGTACGTGACATGCCTACCATGTTTGTAACGGGTAACGGGGAATCAGGGTTCGATTCCGGAGAGGGAGCATGAGAAACGGCTACCACATCCAAGGAAGGCAGCAGGCGCGCAACTTACCCACTCCCGGCACGGGGAGGTAGTGACGAAAAATAACAATACGGGACTCTTTCGAGGCCCCGTAATTGGAATGAGTACACTTTAAACCCTTTAACGAGGATCTATTGGAGGGCAAGTCTGGTGCCAGCAGCCGCGGTAATTCCAGCTCCAATAGCGTATATTAAAGTTGTTGCAGTTAAAAAGCTCGTAGTTGGATCTCAGGCGCAGGCGGGTGGTCCGGCTCGCGCCGGCTCACTGCCCGTACTCCTGCCCTACCTGTTGTCGGCTCTCTCCCGCGGGTGCTCTTCGCTGAGCGTCCCGGGTGGCCGGCGCGTTTACTTTGAAAAAATTAGAGTGTTCAAAGCAGGCTCGGCTGCCTGAATAATGGTGCATGGAATAATGGAATAGGACCTCGGTTCTATTTTGTTGGTTTTCGGAACTAGAGGTAATGATTAACAGGGACAAACGGGGGCATTCGTATTGCTGCGTTAGAGGTGAAATTCTTGGATCGCAGCAAGACGAGCTACTGCGAAAGCATTTGTCAAGAATGTTTTCATTAATCAAGAACGAAAGTCAGAGGCGCGAAGACGATCAGATACCGTCGTAGTTCTGACCATAAACGATGCCAGCTAGCGATCCGCAGGAGTTGCTTCGATGACTCTGCGGGCAGCTTCCGGGAAACCAAAGTTTTTGGGTTCCGGGGGAAGTATGGTTGCAAAGCTGAAACTTAAAGGAATTGACGGAAGGGCACCACCAGGAGTGGAGCCTGCGGCTTAATTTGACTCAACACGGGAAAACTCACCCGGTCCGGACACTGTAAGGATTGACAGATTGATAGCTCTTTCTTGATTCGGTGGGTGGTGGTGCATGGCCGTTCTTAGTTGGTGGAGCGATTTGTCTGGTTAATTCCGATAACGAACGAGACTCTAGCCTATTAAATAGTTCGCCGATTCCTTGATGCGTCGGCGCAACTTCTTAGAGGGACGAGTGGCGTTTAGCCACACGAGATTGAGCAATAACAGGTCTGTGATGCCCTTAGATGTCCGGGGCCGCACGCGCGCTACACTGAAGGAATCAGCGTGGATGCCTCCCTGGTCCGAAAGGATTGGGAAACCCGTTGAATCTCCTTCGTGCTAGGGATTGGGGCTTGTAATTCTTCCCCATGAACGAGGAATTCCCAGTAAGCGCGAGTCATAAGCTCGCGTTGATTACGTCCCTGCCCTTTGTACACACCGCCCGTCGCTACTATCGATTGAGCGGTTCAGTGAGGGCCTCGGATTGGTCTCGGTCTGGTGTGCAAGCGCCGGCACCGTTGGCCGAGAAGACGCTCGAACTCGATCGCTTGGAGAAAGTAAAAGTCGTAACAAGGTTTCCGTAGGTG

>SICBC2010KJ01E03

TTGTCTCAAAGATTAAGCCATGCATGTCTAAGTTCACACTATCTCACGGTGAAACCGCGAATGGCTCATTAAATCAGTCGAGGTTCCTTAGATGACACGATCCTACTTGGATAACTGTGGCAATTCTAGAGCTAATACATGCCTCTGAAGCTCCGACCTTTGCTGGGAAGAGCGCTTTTATTAGTTCAAAACCAATCGTGCGCGCACGCCCCGTTTGGTGACTCTGGATAACTTTGTGCTGATCGCATGGCCTCCTGCGCCGGCGACGCATCTTTCAAATGTCTGCCCTATCAAATGTCGATGGTACGTGACATGCCTACCATGTTTGTAACGGGTAACGGGGAATCAGGGTTCGATTCCGGAGAGGGAGCATGAGAAACGGCTACCACATCCAAGGAAGGCAGCAGGCGCGCAACTTACCCACTCCCGGCACGGGGAGGTAGTGACGAAAAATAACAATACGGGACTCTTTCGAGGCCCCGTAATTGGAATGAGTACACTTTAAACCCTTTAACGAGGATCTATTGGAGGGCAAGTCTGGTGCCAGCAGCCGCGGTAATTCCAGCTCCAATAGCGTATATTAAAGTTGTTGCAGTTAAAAAGCTCGTAGTTGGATCTCAGGCGCAGGCGGGTGGTCCGGCTCGCGCCGGCTCACTGCCCGTACTCCTGCCCTACCTGTTGTCGGCTCTCTCCCGCGGGTGCTCTTCGCTGAGCGTCCCGGGTGGCCGGCGCGTTTACTTTGAAAAAATTAGAGTGTTCAAAGCAGGCTCGGCTGCCTGAATAATGGTGCATGGAATAATGGAATAGGACCTCGGTTCTATTTTGTTGGTTTTCGGAACTAGAGGTAATGATTAACAGGGACAAACGGGGGCATTCGTATTGCTGCGTTAGAGGTGAAATTCTTGGATCGCAGCAAGACGAGCTACTGCGAAAGCATTTGTCAAGAATGTTTTCATTAATCAAGAACGAAAGTCAGAGGCGCGAAGACGATCAGATACCGTCGTAGTTCTGACCATAAACGATGCCAGCTAGCGATCCGCAGGAGTTGCTTCGATGACTCTGCGGGCAGCTTCCGGGAAACCAAAGTTTTTGGGTTCCGGGGGAAGTATGGTTGCAAAGCTGAAACTTAAAGGAATTGACGGAAGGGCACCACCAGGAGTGGAGCCTGCGGCTTAATTTGACTCAACACGGGAAAACTCACCCGGTCCGGACACTGTAAGGATTGACAGATTGATAGCTCTTTCTTGATTCGGTGGGTGGTGGTGCATGGCCGTTCTTAGTTGGTGGAGCGATTTGTCTGGTTAATTCCGATAACGAACGAGACTCTAGCCTATTAAATAGTTCGCCGATTCCTTGATGCGTCGGCGCAACTTCTTAGAGGGACGAGTGGCGTTTAGCCACACGAGATTGAGCAATAACAGGTCTGTGATGCCCTTAGATGTCCGGGGCCGCACGCGCGCTACACTGAAGGAATCAGCGTGGATGCCTCCCTGGTCCGAAAGGATTGGGAAACCCGTTGAATCTCCTTCGTGCTAGGGATTGGGGCTTGTAATTCTTCCCCATGAACGAGGAATTCCCAGTAAGCGCGAGTCATAAGCTCGCGTTGATTACGTCCCTGCCCTTTGTACACACCGCCCGTCGCTACTATCGATTGAGCGGTTCAGTGAGGGCCTCGGATTGGTCTCGGTCTGGTGTGCAAGCGCCGGCACCGTTGGCCGAGAAGACGCTCGAACTCGATCGCTTGGAGAAAGTAAAAGTCGTAACAAGGTTTCCGTAGGTG

>ZSM20090198

TTGTCTCAAAGATTAAGCCATGCATGTCTAAGTTCACACTATCTCACGGTGAAACCGCGAATGGCTCATTAAATCAGTCGAGGTTCCTTAGATGACACGATCCTACTTGGATAACTGTGGCAATTCTAGAGCTAATACATGCCTCTGAAGCTCCGACCTTTGCTGGGAAGAGCGCTTTTATTAGTTCAAAACCAATCGTGCGCGCACGCCCCGTTTGGTGACTCTGGATAACTTTGTGCTGATCGCATGGCCTCCTGCGCCGGCGACGCATCTTTCAAATGTCTGCCCTATCAAATGTCGATGGTACGTGACATGCCTACCATGTTTGTAACGGGTAACGGGGAATCAGGGTTCGATTCCGGAGAGGGAGCATGAGAAACGGCTACCACATCCAAGGAAGGCAGCAGGCGCGCAACTTACCCACTCCCGGCACGGGGAGGTAGTGACGAAAAATAACAATACGGGACTCTTTCGAGGCCCCGTAATTGGAATGAGTACACTTTAAACCCTTTAACGAGGATCTATTGGAGGGCAAGTCTGGTGCCAGCAGCCGCGGTAATTCCAGCTCCAATAGCGTATATTAAAGTTGTTGCAGTTAAAAAGCTCGTAGTTGGATCTCAGGCGCAGGCGGGTGGTCCGGCTCGCGCCGGCTCACTGCCCGTACTCCTGCCCTACCTGTTGTCGGCTCTCTCCCGCGGGTGCTCTTCGCTGAGCGTCCCGGGTGGCCGGCGCGTTTACTTTGAAAAAATTAGAGTGTTCAAAGCAGGCTCGGCTGCCTGAATAATGGTGCATGGAATAATGGAATAGGACCTCGGTTCTATTTTGTTGGTTTTCGGAACTAGAGGTAATGATTAACAGGGACAAACGGGGGCATTCGTATTGCTGCGTTAGAGGTGAAATTCTTGGATCGCAGCAAGACGAGCTACTGCGAAAGCATTTGTCAAGAATGTTTTCATTAATCAAGAACGAAAGTCAGAGGCGCGAAGACGATCAGATACCGTCGTAGTTCTGACCATAAACGATGCCAGCTAGCGATCCGCAGGAGTTGCTTCGATGACTCTGCGGGCAGCTTCCGGGAAACCAAAGTTTTTGGGTTCCGGGGGAAGTATGGTTGCAAAGCTGAAACTTAAAGGAATTGACGGAAGGGCACCACCAGGAGTGGAGCCTGCGGCTTAATTTGACTCAACACGGGAAAACTCACCCGGTCCGGACACTGTAAGGATTGACAGATTGATAGCTCTTTCTTGATTCGGTGGGTGGTGGTGCATGGCCGTTCTTAGTTGGTGGAGCGATTTGTCTGGTTAATTCCGATAACGAACGAGACTCTAGCCTATTAAATAGTTCGCCGATTCCTTGATGCGTCGGCGCAACTTCTTAGAGGGACGAGTGGCGTTTAGCCACACGAGATTGAGCAATAACAGGTCTGTGATGCCCTTAGATGTCCGGGGCCGCACGCGCGCTACACTGAAGGAATCAGCGTGGATGCCTCCCTGGTCCGAAAGGATTGGGAAACCCGTTGAATCTCCTTCGTGCTAGGGATTGGGGCTTGTAATTCTTCCCCATGAACGAGGAATTCCCAGTAAGCGCGAGTCATAAGCTCGCGTTGATTACGTCCCTGCCCTTTGTACACACCGCCCGTCGCTACTATCGATTGAGCGGTTCAGTGAGGGCCTCGGATTGGTCTCGGTCTGGTGTGCAAGCGCCGGCACCGTTGGCCGAGAAGACGCTCGAACTCGATCGCTTGGAGAAAGTAAAAGTCGTAACAAGGTTTCCGTAGGTG

>ZSM20071133

TTGTCTCAAAGATTAAGCCATGCATGTCTAAGTTCACACTATCTCACGGTGAAACCGCGAATGGCTCATTAAATCAGTCGAGGTTCCTTAGATGACACGATCCTACTTGGATAACTGTGGCAATTCTAGAGCTAATACATGCCTCTGAAGCTCCGACCTCCCACGGGAAGAGCGCTTTTATTAGTTCAAAACCAATCGTGCGCGCGCGCCCACTTTGGTGACTCTGGATAACTTTGTGCTGATCGCATGGCCTCCTGCGCCGGCGACGCATCTTTCAAATGTCTGCCCTATCAAATGTCGATGGTACGTGACATGCCTACCATGTTTGTAACGGGTAACGGGGAATCAGGGTTCGATTCCGGAGAGGGAGCATGAGAAACGGCTACCACATCCAAGGAAGGCAGCAGGCGCGCAACTTACCCACTCCCGGCACGGGGAGGTAGTGACGAAAAATAACAATACGGGACTCTTTCGAGGCCCCGTAATTGGAATGAGTACACTTTAAACCCTTTAACGAGGATCTATTGGAGGGCAAGTCTGGTGCCAGCAGCCGCGGTAATTCCAGCTCCAATAGCGTATATTAAAGTTGTTGCAGTTAAAAAGCTCGTAGTTGGATCTCAGGCGCAGGCGGGTGGTCCGGCTCGCGCCGGCTCACTGCCCGTACTCCTGCCCTACCTGTTGTCGGCTCTCTCCCGCGGGTGCTCTTCACTGAGCGTCCCGGGTGGCCGGCGCGTTTACTTTGAAAAAATTAGAGTGTTCAAAGCAGGCTCGGCTGCCTGAATAATGGTGCATGGAATAATGGAATAGGACCTCGGTTCTATTTTGTTGGTTTTCGGAACTAGAGGTAATGATTAACAGGGACAAACGGGGGCATTCGTATTGCTGCGTTAGAGGTGAAATTCTTGGATCGCAGCAAGACGAGCTACTGCGAAAGCATTTGTCAAGAATGTTTTCATTAATCAAGAACGAAAGTCAGAGGCGCGAAGACGATCAGATACCGTCGTAGTTCTGACCATAAACGATGCCAGCTAGCGATCCGCAGGAGTTGCTTCGATGACTCTGCGGGCAGCTTCCGGGAAACCAAAGTTTTTGGGTTCCGGGGGAAGTATGGTTGCAAAGCTGAAACTTAAAGGAATTGACGGAAGGGCACCACCAGGAGTGGAGCCTGCGGCTTAATTTGACTCAACACGGGAAAACTCACCCGGTCCGGACACTGTAAGGATTGACAGATTGATAGCTCTTTCTTGATTCGGTGGGTGGTGGTGCATGGCCGTTCTTAGTTGGTGGAGCGATTTGTCTGGTTAATTCCGATAACGAACGAGACTCTAGCCTATTAAATAGTTCGCCGATTCCTTGATGCGTCGGCGCAACTTCTTAGAGGGACGAGTGGCGTTTAGCCACACGAGATTGAGCAATAACAGGTCTGTGATGCCCTTAGATGTCCGGGGCCGCACGCGCGCTACACTGAAGGAATCAGCGTGGATGCCTCCCTGGTCCGAAAGGATTGGGAAACCCGTTGAATCTCCTTCGTGCTAGGGATTGGGGCTTGTAATTCTTCCCCATGAACGAGGAATTCCCAGTAAGCGCGAGTCATAAGCTCGCGTTGATTACGTCCCTGCCCTTTGTACACACCGCCCGTCGCTACTATCGATTGAGCGGTTCAGTGAGGGCCTCGGATTGGTCTCGGTCTGGCGTGCAAGCGCCGGCACCGTTGGCCGAGAAGACGCTCGAACTCGATCGCTTGGAGAAAGTAAAAGTCGTAACAAGGTTTNNNNNNNNN

>ZSM20080054

TTGTCTCAAAGATTAAGCCATGCATGTCTAAGTTCACACTATCTCACGGTGAAACCGCGAATGGCTCATTAAATCAGTCGAGGTTCCTTAGATGACACGATCCTACTTGGATAACTGTGGCAATTCTAGAGCTAATACATGCCTCTGAAGCTCCGACCCTTGCGGGGAAGAGCGCTTTTATTAGTTCAAAACCAATCGTGCGCGCACGCCCACTTTGGTGACTCTGGATAACTTTGTGCTGATCGCATGGCCTCCTGCGCCGGCGACGCATCTTTCAAATGTCTGCCCTATCAAATGTCGATGGTACGTGACATGCCTACCATGTTTGTAACGGGTAACGGGGAATCAGGGTTCGATTCCGGAGAGGGAGCATGAGAAACGGCTACCACATCCAAGGAAGGCAGCAGGCGCGCAACTTACCCACTCCCGGCACGGGGAGGTAGTGACGAAAAATAACAATACGGGACTCTTTCGAGGCCCCGTAATTGGAATGAGTACACTTTAAACCCTTTAACGAGGATCTATTGGAGGGCAAGTCTGGTGCCAGCAGCCGCGGTAATTCCAGCTCCAATAGCGTATATTAAAGTTGTTGCAGTTAAAAAGCTCGTAGTTGGATCTCAGGCGCAGGCGGGTGGTCCGGCTCGCGCCGGCTCACTGCCCGTACTCCTGCCCTACCTGTTGTCGGCTCTCTCCCGCGGGTGCTCTTCACTGAGCGTCCCGGGTGGCCGGCGCGTTTACTTTGAAAAAATTAGAGTGTTCAAAGCAGGCTCGGCTGCCTGAATAATGGTGCATGGAATAATGGAATAGGACCTCGGTTCTATTTTGTTGGTTTTCGGAACTAGAGGTAATGATTAACAGGGACAAACGGGGGCATTCGTATTGCTGCGTTAGAGGTGAAATTCTTGGATCGCAGCAAGACGAGCTACTGCGAAAGCATTTGTCAAGAATGTTTTCATTAATCAAGAACGAAAGTCAGAGGCGCGAAGACGATCAGATACCGTCGTAGTTCTGACCATAAACGATGCCAGCTAGCGATCCGCAGGAGTTGCTTCGATGACTCTGCGGGCAGCTTCCGGGAAACCAAAGTTTTTGGGTTCCGGGGGAAGTATGGTTGCAAAGCTGAAACTTAAAGGAATTGACGGAAGGGCACCACCAGGAGTGGAGCCTGCGGCTTAATTTGACTCAACACGGGAAAACTCACCCGGTCCGGACACTGTAAGGATTGACAGATTGATAGCTCTTTCTTGATTCGGTGGGTGGTGGTGCATGGCCGTTCTTAGTTGGTGGAGCGATTTGTCTGGTTAATTCCGATAACGAACGAGACTCTAGCCTATTAAATAGTTCGCCGATTCCTTGATGCGTCGGCGCAACTTCTTAGAGGGACGAGTGGCGTTTAGCCACACGAGATTGAGCAATAACAGGTCTGTGATGCCCTTAGATGTCCGGGGCCGCACGCGCGCTACACTGAAGGAATCAGCGTGGATGCCTCCCTGGTCCGAAAGGATTGGGAAACCCGTTGAATCTCCTTCGTGCTAGGGATTGGGGCTTGTAATTCTTCCCCATGAACGAGGAATTCCCAGTAAGCGCGAGTCATAAGCTCGCGTTGATTACGTCCCTGCCCTTTGTACACACCGCCCGTCGCTACTATCGATTGAGCGGTTCAGTGAGGGCCTCGGATTGGTCTCGGTCTGGCGTGCAAGCGCCGGCACCGTTGGCCGAGAAGACGCTCGAACTCGATCGCTTGGAGAAAGTAAAAGTCGTAACAAGGTTTCCGTAGGTG

>ZSM20080953

TTGTCTCAAAGATTAAGCCATGCATGTCTAAGTTCACACTATCTCACGGTGAAACCGCGAATGGCTCATTAAATCAGTCGAGGTTCCTTAGATGACACGATCCTACTTGGATAACTGTGGCAATTCTAGAGCTAATACATGCCTCTGAAGCTCCGACCCTTGCGGGGAAGAGCGCTTTTATTAGTTCAAAACCAATCGTGCGCGCACGCCCACTTTGGTGACTCTGGATAACTTTGTGCTGATCGCATGGCCTCCTGCGCCGGCGACGCATCTTTCAAATGTCTGCCCTATCAAATGTCGATGGTACGTGACATGCCTACCATGTTTGTAACGGGTAACGGGGAATCAGGGTTCGATTCCGGAGAGGGAGCATGAGAAACGGCTACCACATCCAAGGAAGGCAGCAGGCGCGCAACTTACCCACTCCCGGCACGGGGAGGTAGTGACGAAAAATAACAATACGGGACTCTTTCGAGGCCCCGTAATTGGAATGAGTACACTTTAAACCCTTTAACGAGGATCTATTGGAGGGCAAGTCTGGTGCCAGCAGCCGCGGTAATTCCAGCTCCAATAGCGTATATTAAAGTTGTTGCAGTTAAAAAGCTCGTAGTTGGATCTCAGGCGCAGGCGGGTGGTCCGGCTCGCGCCGGCTCACTGCCCGTACTCCTGCCCTACCTGTTGTCGGCTCTCTCCCGCGGGTGCTCTTCACTGAGCGTCCCGGGTGGCCGGCGCGTTTACTTTGAAAAAATTAGAGTGTTCAAAGCAGGCTCGGCTGCCTGAATAATGGTGCATGGAATAATGGAATAGGACCTCGGTTCTATTTTGTTGGTTTTCGGAACTAGAGGTAATGATTAACAGGGACAAACGGGGGCATTCGTATTGCTGCGTTAGAGGTGAAATTCTTGGATCGCAGCAAGACGAGCTACTGCGAAAGCATTTGTCAAGAATGTTTTCATTAATCAAGAACGAAAGTCAGAGGCGCGAAGACGATCAGATACCGTCGTAGTTCTGACCATAAACGATGCCAGCTAGCGATCCGCAGGAGTTGCTTCGATGACTCTGCGGGCAGCTTCCGGGAAACCAAAGTTTTTGGGTTCCGGGGGAAGTATGGTTGCAAAGCTGAAACTTAAAGGAATTGACGGAAGGGCACCACCAGGAGTGGAGCCTGCGGCTTAATTTGACTCAACACGGGAAAACTCACCCGGTCCGGACACTGTAAGGATTGACAGATTGATAGCTCTTTCTTGATTCGGTGGGTGGTGGTGCATGGCCGTTCTTAGTTGGTGGAGCGATTTGTCTGGTTAATTCCGATAACGAACGAGACTCTAGCCTATTAAATAGTTCGCCGATTCCTTGATGCGTCGGCGCAACTTCTTAGAGGGACGAGTGGCGTTTAGCCACACGAGATTGAGCAATAACAGGTCTGTGATGCCCTTAGATGTCCGGGGCCGCACGCGCGCTACACTGAAGGAATCAGCGTGGATGCCTCCCTGGTCCGAAAGGATTGGGAAACCCGTTGAATCTCCTTCGTGCTAGGGATTGGGGCTTGTAATTCTTCCCCATGAACGAGGAATTCCCAGTAAGCGCGAGTCATAAGCTCGCGTTGATTACGTCCCTGCCCTTTGTACACACCGCCCGTCGCTACTATCGATTGAGCGGTTCAGTGAGGGCCTCGGATTGGTCTCGGTCTGGCGTGCAAGCGCCGGCACCGTTGGCCGAGAAGACGCTCGAACTCGATCGCTTGGAGAAAGTAAAAGTCGTAACAAGGTTTCNNNNNNNN

>ZSM20081014

TTGTCTCAAAGATTAAGCCATGCATGTCTAAGTTCACACTATCTCACGGTGAAACCGCGAATGGCTCATTAAATCAGTCGAGGTTCCTTAGATGACACGATCCTACTTGGATAACTGTGGCAATTCTAGAGCTAATACATGCCTCTGAAGCTCCGACCTGTGCAGGGAAGAGCGCTTTTATTAGTTCAAAACCAATCGTGCGCGCGCGCCCACTTTGGTGACTCTGGATAACTTTGTGCTGATCGCATGGCCTCCTGCGCCGGCGACGCATCTTTCAAATGTCTGCCCTATCAAATGTCGATGGTACGTGACATGCCTACCATGTTTGTAACGGGTAACGGGGAATCAGGGTTCGATTCCGGAGAGGGAGCATGAGAAACGGCTACCACATCCAAGGAAGGCAGCAGGCGCGCAACTTACCCACTCCCGGCACGGGGAGGTAGTGACGAAAAATAACAATACGGGACTCTTTCGAGGCCCCGTAATTGGAATGAGTACACTTTAAACCCTTTAACGAGGATCTATTGGAGGGCAAGTCTGGTGCCAGCAGCCGCGGTAATTCCAGCTCCAATAGCGTATATTAAAGTTGTTGCAGTTAAAAAGCTCGTAGTTGGATCTCAGGCGCAGGCGGGTGGTCCGGCTCGCGCCGGCTCACTGCCCGTACTCCTGCCCTACCTGTTGTCGGCTCTCTCCCGCGGGTGCTCTTCACTGAGCGTCCCGGGTGGCCGGCGCGTTTACTTTGAAAAAATTAGAGTGTTCAAAGCAGGCTATTCAGCCTGAATAATGGTGCATGGAATAATGGAATAGGACCTCGGTTCTATTTTGTTGGTTTTCGGAACTAGAGGTAATGATTAACAGGGACAAACGGGGGCATTCGTATTGCTGCGTTAGAGGTGAAATTCTTGGATCGCAGCAAGACGAGCTACTGCGAAAGCATTTGTCAAGAATGTTTTCATTAATCAAGAACGAAAGTCAGAGGCGCGAAGACGATCAGATACCGTCGTAGTTCTGACCATAAACGATGCCAGCTAGCGATCCGCAGGAGTTGCTTCGATGACTCTGCGGGCAGCTTCCGGGAAACCAAAGTTTTTGGGTTCCGGGGGAAGTATGGTTGCAAAGCTGAAACTTAAAGGAATTGACGGAAGGGCACCACCAGGAGTGGAGCCTGCGGCTTAATTTGACTCAACACGGGAAAACTCACCCGGTCCGGACACTGTAAGGATTGACAGATTGATAGCTCTTTCTTGATTCGGTGGGTGGTGGTGCATGGCCGTTCTTAGTTGGTGGAGCGATTTGTCTGGTTAATTCCGATAACGAACGAGACTCTAGCCTATTAAATAGTTCGCCGATTCCTTGATGCGTCGGCGCAACTTCTTAGAGGGACGAGTGGCGTTTAGCCACACGAGATTGAGCAATAACAGGTCTGTGATGCCCTTAGATGTCCGGGGCCGCACGCGCGCTACACTGAAGGAATCAGCGTGGATGCCTCCCTGGTCCGAAAGGATTGGGAAACCCGTTGAATCTCCTTCGTGCTAGGGATTGGGGCTTGTAATTCTTCCCCATGAACGAGGAATTCCCAGTAAGCGCGAGTCATAAGCTCGCGTTGATTACGTCCCTGCCCTTTGTACACACCGCCCGTCGCTACTATCGATTGAGCGGTTCAGTGAGGGCCTCGGATTGGTCTCGGTCTGGCGTGCAAGCGCCGGCACCGTTGGCCGAGAAGACGCTCGAACTCGATCGCTTGGAGAAAGTAAAAGTCGTAACAAGGTTTCCGTAGGTG

>ZSM20100379

TTGTCTCAAAGATTAAGCCATGCATGTCTAAGTTCACACTATCTCACGGTGAAACCGCGAATGGCTCATTAAATCAGTCGAGGTTCCTTAGATGACACGATCCTACTTGGATAACTGTGGCAATTCTAGAGCTAATACATGCCTCTGAAGCTCCGACCTGTGCAGGGAAGAGCGCTTTTATTAGTTCAAAACCAATCGTGCGCGCGCGCCCACTTTGGTGACTCTGGATAACTTTGTGCTGATCGCATGGCCTCCTGCGCCGGCGACGCATCTTTCAAATGTCTGCCCTATCAAATGTCGATGGTACGTGACATGCCTACCATGTTTGTAACGGGTAACGGGGAATCAGGGTTCGATTCCGGAGAGGGAGCATGAGAAACGGCTACCACATCCAAGGAAGGCAGCAGGCGCGCAACTTACCCACTCCCGGCACGGGGAGGTAGTGACGAAAAATAACAATACGGGACTCTTTCGAGGCCCCGTAATTGGAATGAGTACACTTTAAACCCTTTAACGAGGATCTATTGGAGGGCAAGTCTGGTGCCAGCAGCCGCGGTAATTCCAGCTCCAATAGCGTATATTAAAGTTGTTGCAGTTAAAAAGCTCGTAGTTGGATCTCAGGCGCAGGCGGGTGGTCCGGCTCGCGCCGGCTCACTGCCCGTACTCCTGCCCTACCTGTTGTCGGCTCTCTCCCGCGGGTGCTCTTCACTGAGCGTCCCGGGTGGCCGGCGCGTTTACTTTGAAAAAATTAGAGTGTTCAAAGCAGGCTATTCAGCCTGAATAATGGTGCATGGAATAATGGAATAGGACCTCGGTTCTATTTTGTTGGTTTTCGGAACTAGAGGTAATGATTAACAGGGACAAACGGGGGCATTCGTATTGCTGCGTTAGAGGTGAAATTCTTGGATCGCAGCAAGACGAGCTACTGCGAAAGCATTTGTCAAGAATGTTTTCATTAATCAAGAACGAAAGTCAGAGGCGCGAAGACGATCAGATACCGTCGTAGTTCTGACCATAAACGATGCCAGCTAGCGATCCGCAGGAGTTGCTTCGATGACTCTGCGGGCAGCTTCCGGGAAACCAAAGTTTTTGGGTTCCGGGGGAAGTATGGTTGCAAAGCTGAAACTTAAAGGAATTGACGGAAGGGCACCACCAGGAGTGGAGCCTGCGGCTTAATTTGACTCAACACGGGAAAACTCACCCGGTCCGGACACTGTAAGGATTGACAGATTGATAGCTCTTTCTTGATTCGGTGGGTGGTGGTGCATGGCCGTTCTTAGTTGGTGGAGCGATTTGTCTGGTTAATTCCGATAACGAACGAGACTCTAGCCTATTAAATAGTTCGCCGATTCCTTGATGCGTCGGCGCAACTTCTTAGAGGGACGAGTGGCGTTTAGCCACACGAGATTGAGCAATAACAGGTCTGTGATGCCCTTAGATGTCCGGGGCCGCACGCGCGCTACACTGAAGGAATCAGCGTGGATGCCTCCCTGGTCCGAAAGGATTGGGAAACCCGTTGAATCTCCTTCGTGCTAGGGATTGGGGCTTGTAATTCTTCCCCATGAACGAGGAATTCCCAGTAAGCGCGAGTCATAAGCTCGCGTTGATTACGTCCCTGCCCTTTGTACACACCGCCCGTCGCTACTATCGATTGAGCGGTTCAGTGAGGGCCTCGGATTGGTCTCGGTCTGGCGTGCAAGCGCCGGCACCGTTGGCCGAGAAGACGCTCGAACTCGATCGCTTGGAGAAAGTAAAAGTCGTAACAAGGTTTCCGTAGGTG

>AMC476051001

TTGTCTCAAAGATTAAGCCATGCATGTCTAAGTTCACACTATCTCACGGTGAAACCGCGAATGGCTCATTAAATCAGTCGAGGTTCCTTAGATGACACGATCCTACTTGGATAACTGTGGCAATTCTAGAGCTAATACATGCCTCTGAAGCTCCGACCTGTGCAGGGAAGAGCGCTTTTATTAGTTCAAAACCAATCGCGCGCGCGCGCCCACTTTGGTGACTCTGGATAACTTTGTGCTGATCGCATGGCCTCCTGCGCCGGCGACGCATCTTTCAAATGTCTGCCCTATCAAATGTCGATGGTACGTGACATGCCTACCATGTTTGTAACGGGTAACGGGGAATCAGGGTTCGATTCCGGAGAGGGAGCATGAGAAACGGCTACCACATCCAAGGAAGGCAGCAGGCGCGCAACTTACCCACTCCCGGCACGGGGAGGTAGTGACGAAAAATAACAATACGGGACTCTTTCGAGGCCCCGTAATTGGAATGAGTACACTTTAAACCCTTTAACGAGGATCTATTGGAGGGCAAGTCTGGTGCCAGCAGCCGCGGTAATTCCAGCTCCAATAGCGTATATTAAAGTTGTTGCAGTTAAAAAGCTCGTAGTTGGATCTCAGGCGCAGGCGGGTGGTCCGGCTCGCGCCGGCTCACTGCCCGTACTCCTGCCCTACCTGTTGTCGGCTCTCTCCCGCGGGTGCTCTTCGCTGAGCGTCCCGGGTGGCCGGCGCGTTTACTTTGAAAAAATTAGAGTGTTCAAAGCAGGCTATTCAGCCTGAATAATGGTGCATGGAATAATGGAATAGGACCTCGGTTCTATTTTGTTGGTTTTCGGAACTAGAGGTAATGATTAACAGGGACAAACGGGGGCATTCGTATTGCTGCGTTAGAGGTGAAATTCTTGGATCGCAGCAAGACGAGCTACTGCGAAAGCATTTGTCAAGAATGTTTTCATTAATCAAGAACGAAAGTCAGAGGCGCGAAGACGATCAGATACCGTCGTAGTTCTGACCATAAACGATGCCAGCTAGCGATCCGCAGGAGTTGCTTCGATGACTCTGCGGGCAGCTTCCGGGAAACCAAAGTTTTTGGGTTCCGGGGGAAGTATGGTTGCAAAGCTGAAACTTAAAGGAATTGACGGAAGGGCACCACCAGGAGTGGAGCCTGCGGCTTAATTTGACTCAACACGGGAAAACTCACCCGGTCCGGACACTGTAAGGATTGACAGATTGATAGCTCTTTCTTGATTCGGTGGGTGGTGGTGCATGGCCGTTCTTAGTTGGTGGAGCGATTTGTCTGGTTAATTCCGATAACGAACGAGACTCTAGCCTATTAACTAGTTCGCCGATTCCTTGATGCGTCGGCGCAACTTCTTAGAGGGACGAGTGGCGTTTAGCCACACGAGATTGAGCAATAACAGGTCTGTGATGCCCTTAGATGTCCGGGGCCGCACGCGCGCTACACTGAAGGAATCAGCGTGGATGCCTCCCTGGTCCGAAAGGATTGGGAAACCCGTTGAATCTCCTTCGTGCTAGGGATTGGGGCTTGTAATTCTTCCCCATGAACGAGGAATTCCCAGTAAGCGCGAGTCATAAGCTCGCGTTGATTACGTCCCTGCCCTTTGTACACACCGCCCGTCGCTACTATCGATTGAGCGGTTCAGTGAGGGCCTCGGATTGGTCTCGGTCTGGCGTGCAAGCGCCGGCACCGTTGGCCGAGAAGACGCTCGAACTCGATCGCTTGGAGAAAGTAAAAGTCGTAACAAGGTTTCCGTAGGTG
