# Supplementary material for: How to describe a cryptic species? Practical challenges of molecular taxonomy
Source: Front Zool. 2013 Sep 27;10:59. doi: 10.1186/1742-9994-10-59 (PMC4015967; doi:10.1186/1742-9994-10-59)
Supplement: Additional file 4 — 28S rRNA alignment of Pontohedyle (fasta format). The alignment was generated with MUSCLE [107] and ambiguous parts of the alignment were masked with Gblocks [108] (settings for a less stringent selection). [file 1742-9994-10-59-S4.docx]

### Additional file 4 – 28S rRNA alignment of *Pontohedyle* (fasta format)

The alignment was generated with Muscle [[96](#_ENREF_96)] and ambiguous parts of the alignment were masked with Gblocks [[97](#_ENREF_97)] (settings for a less stringent selection).

>ZSM20100597

NNNATTCCCCCAGTAACGGCGAGTGAAGCGGGAAGAGCCCAGCACCGAATCCCTCAGTGTCATGCTGACGGGAACTGTGGTGTGTGGGACGCCACCAGTCGCATTAAAGGGCGCCGAAGTCCTCCTGATCGAGGCTTCACCCAGAGCGGGTGTAAGGCCAGTGCTGGTGCCTCTTTGTGCGGCCGCGAGCGTCTCAGGAGTCGGGTTGTTTGGGAATGCAGCCCAAAGCGGGTGGTAAACTCCATCTAAGGCTAAATACCGGCACGAGTCCGATAGCGGACAAGTACCGTGAGGGAAAGTTGAAAAGAACTTTGAAGAGAGAGTTCAAGAGTACGTGAAACCGCCCAGAGGTAAACGGGTGCATCCGCAAAGTCGGCCCGTGGAATTCAGCGCGGCGCGCGGCCTGGGGCTGCTTCGTTTCGGGATCCCTGGGACCCGAGCGGGGTGCTGCGCCGGGCTCCGCCGCGTGCACTTTCTGCGGGTAGAGCGCCACGACCGGTTTCGCGGTGGCGACGAGCCGGGCGGGAAGGTATGC---TGCTTCG--GCTGTTATAGCCCGTCCTGGTGAGCTGCTGCGGGACCGAGGGACGGCCGCGTTCTTCGAGGCCACCCGGCCTTCTCGGGGAGTTCGACTGGTAGAGACTGGGCAACCGTGTCTGCCGACCGCTTCTCGAGACCGGATCGGGCTGGCCGGGAACGCTGAGGGTCGGTGGCGAATCTGTCGGCATTGCACCCGACCCGTCTTGAAACACGGACCAAGGAGTCTAACATGCGCGCGAGTCATTGGGTCGTACGAAACTCAAAGGCGCAGTGAAAGCGAGGGTCGCCCCGGGCTGACCCAGGTGGGATCCCTCCGTTCCTCGGAGCGGGGGGCGCACCACCGGCCCGTCCCGTCCGCGTTGTCGGTGGGGCGGAGCAGGAGCGTGCACGCTGGGACCCGAAAGATGGTGAACTATGCCTGAGTAGAACGAAGCCAGAGAAA

>ZSM20100603

NNNATTCCCCCAGTAACGGCGAGTGAAGCGGGAAGAGCCCAGCACCGAATCCCTCAGTGTCATGCTGACGGGAACTGTGGTGTGTGGGACGCCACCAGTCGCATTAAAGGGCGCCGAAGTCCTCCTGATCGAGGCTTCACCCAGAGCGGGTGTAAGGCCAGTGCTGGTGCCTCTTTGTGCGGCCGCGAGCGTCTCAGGAGTCGGGTTGTTTGGGAATGCAGCCCAAAGCGGGTGGTAAACTCCATCTAAGGCTAAATACCGGCACGAGTCCGATAGCGGACAAGTACCGTGAGGGAAAGTTGAAAAGAACTTTGAAGAGAGAGTTCAAGAGTACGTGAAACCGCCCAGAGGTAAACGGGTGCATCCGCAAAGTCGGCCCGTGGAATTCAGCGCGGCGCGCGGCCTGGGGCTGCTTCGTTTCGGGATCCCTGGGACCCGAGCGGGGTGCTGCGCCGGGCTCCGCCGCGTGCACTTTCTGCGGGTAGAGCGCCACGACCGGTTTCGCGGTGGCGACGAGCCGGGCGGGAAGGTATGC---TGCTTCG--GCTGTTATAGCCCGTCCTGGTGAGCTGCTGCGGGACCGAGGGACGGCCGCGTTCTTCGAGGCCACCCGGCCTTCTCGGGGAGTTCGACTGGTAGAGACTGGGCAACCGTGTCTGCCGACCGCTTCTCGAGACCGGATCGGGCTGGCCGGGAACGCTGAGGGTCGGTGGCGAATCTGTCGGCATTGCACCCGACCCGTCTTGAAACACGGACCAAGGAGTCTAACATGCGCGCGAGTCATTGGGTCGTACGAAACTCAAAGGCGCAGTGAAAGCGAGGGTCGCCCCGGGCTGACCCAGGTGGGATCCCTCCGTTCCTCGGAGCGGGGGGCGCACCACCGGCCCGTCCCGTCCGCGTTGTCGGTGGGGCGGAGCAGGAGCGTGCACGCTGGGACCCGAAAGATGGTGAACTATGCCTGAGTAGAACGAAGCCAGAGGAA

>ZSM20100595

NNNNNNNNNNNAGTAACGGCGAGTGAAGCGGGAAGAGCCCAGCACCGAATCCCTCAGTGTCATGCTGACGGGAACTGTGGTGTGTGGGACGCCACCAGTCGCATTAAAGGGCGCCGAAGTCCTCCTGATCGAGGCTTCACCCAGAGCGGGTGTAAGGCCAGTGCTGGTGCCTCTTTGTGCGGCCGCGAGCGTCTCAGGAGTCGGGTTGTTTGGGAATGCAGCCCAAAGCGGGTGGTAAACTCCATCTAAGGCTAAATACCGGCACGAGTCCGATAGCGGACAAGTACCGTGAGGGAAAGTTGAAAAGAACTTTGAAGAGAGAGTTCAAGAGTACGTGAAACCGCCCAGAGGTAAACGGGTGCATCCGCAAAGTCGGCCCGTGGAATTCAGCGCGGCGCGCGGCCTGGGGCTGCTTCGTTTCGGGATCCCTGGGACCCGAGCGGGGTGCTGCGCCGGGCTCCGCCGCGTGCACTTTCTGCGGGTAGAGCGCCACGACCGGTTTCGCGGTGGCGACGAGCCGGGCGGGAAGGTATGC---TGCTTCG--GCTGTTATAGCCCGTCCTGGTGAGCTGCTGCGGGACCGAGGGACGGCCGCGTTCTTCGAGGCCACCCGGCCTTCTCGGGGAGTTCGACTGGTAGAGACTGGGCAACCGTGTCTGCCGACCGCTTCTCGAGACCGGATCGGGCTGGCCGGGAACGCTGAGGGTCGGTGGCGAATCTGTCGGCATTGCACCCGACCCGTCTTGAAACACGGACCAAGGAGTCTAACATGCGCGCGAGTCATTGGGTCGTACGAAACTCAAAGGCGCAGTGAAAGCGAGGGTCGCCCCGGGCTGACCCAGGTGGGATCCCTCCGTTCCTCGGAGCGGGGGGCGCACCACCGGCCCGTCCCGTCCGCGTTGTCGGTGGGGCGGAGCAGGAGCGTGCACGCTGGGACCCGAAAGATGGTGAACTATGCCTGAGTAGAACGAAGCCAGANNNN

>SICBC2010KJ01C08

AGGATTCCCCCAGTAACGGCGAGTGAAGCGGGAAGAGCCCAGCACCGAATCCCTCAGTGTCATGCTGACGGGAACTGTGGTGTGTGGGACGCCACCAGTCGCATTAACGGGCGCCGAAGTCCTCCTGATCGAGGCTTCACCCAGAGCGGGTGTAAGGCCAGTGCTGGTGCCTCGTTGTGCGGCCGCGAGCGTCTCAGGAGTCGGGTTGTTTGGGAATGCAGCCCAAAGCGGGTGGTAAACTCCATCTAAGGCTAAATACCGGCACGAGTCCGATAGCGGACAAGTACCGTGAGGGAAAGTTGAAAAGAACTTTGAAGAGAGAGTTCAAGAGTACGTGAAACCGCCCAGAGGTAAACGGGTGCATCCGCAAAGTCGGCCCGTGGAATTCAGCGCGGCGCGCGGCCTGGGGCTGCTTCGCTTCGGGATCCCTGGGACCCGGGCGAGGTGCTGCGCCGGGCTCCGCCGCGTGCACTTTCTGCGGGCAGAGCGCCACGACCGGTTTCGCGGCGGCGACGAGCCGGGCGGGAAGGTAGGCGAGGACGGCATTGCTGTTACAGCCCGTCCTGGTGAGCTGCTGCGGGACCGAGGGACGGCCGCGTTCTTCGAGGCCACCCGGCCTTCTCGGGGAGTTCGACTGGTAGAGACTGGGCAACCGTGTCTGCCGACCGCTTCTCGAGACCGGATCGGGCTGGCCGGGAGCGCTGAGGGTCGGTGGCGAATCTGTCGGCATTGCACCCGACCCGTCTTGAAACACGGACCAAGGAGTCTAACATGCGCGCGAGTCATTGGGTCGTACGAAACTCAAAGGCGCAGTGAAAGCGAGGGTCGCCCCGGGCTGACCCAGGTGGGATCTCTCCGTGCC----AACGGGGAGCGCACCACCGGCCCGTCCCGTCCGCGTTGTCGGTGGGGCGGAGCAGGAGCGTGCACGCTGGGACCCGAAAGATGGTGAACTATGCCTGAGTAGAACGAAGCCAGAGGAA

>ZSM20090197

AGGATTCCCCCAGTAACGGCGAGTGAAGCGGGAAGAGCCCAGCACCGAATCCCTCAGTGTCATGCTGACGGGAACTGTGGTGTGTGGGACGCCACCAGTCGCATTAACGGGCGCCGAAGTCCTCCTGATCGAGGCTTCACCCAGAGCGGGTGTAAGGCCAGTGCTGGTGCCTCGTTGTGCGGCCGCGAGCGTCTCAGGAGTCGGGTTGTTTGGGAATGCAGCCCAAAGCGGGTGGTAAACTCCATCTAAGGCTAAATACCGGCACGAGTCCGATAGCGGACAAGTACCGTGAGGGAAAGTTGAAAAGAACTTTGAAGAGAGAGTTCAAGAGTACGTGAAACCGCCCAGAGGTAAACGGGTGCATCCGCAAAGTCGGCCCGTGGAATTCAGCGCGGCGCGCGGCCTGGGGCTGCTTCGCTTCGGGATCCCTGGGACCCGGGCGAGGTGCTGCGCCGGGCTCCGCCGCGTGCACTTTCTGCGGGCAGAGCGCCACGACCGGTTTCGCGGCGGCGACGAGCCGGGCGGGAAGGTAGGCGAGGACGGCATTGCTGTTACAGCCCGTCCTGGTGAGCTGCTGCGGGACCGAGGGACGGCCGCGTTCTTCGAGGCCACCCGGCCTTCTCGGGGAGTTCGACTGGTAGAGACTGGGCAACCGTGTCTGCCGACCGCTTCTCGAGACCGGATCGGGCTGGCCGGGAGCGCTGAGGGTCGGTGGCGAATCTGTCGGCATTGCACCCGACCCGTCTTGAAACACGGACCAAGGAGTCTAACATGCGCGCGAGTCATTGGGTCGTACGAAACTCAAAGGCGCAGTGAAAGCGAGGGTCGCCCCGGGCTGACCCAGGTGGGATCTCTCCGTGCC----AACGGGGAGCGCACCACCGGCCCGTCCCGTCCGCGTTGTCGGTGGGGCGGAGCAGGAGCGTGCACGCTGGGACCCGAAAGATGGTGAACTATGCCTGAGTAGAACGAAGCCAGAGGAA

>ZSM20081013

AGGATTCCCCCAGTAACGGCGAGTGAAGCGGGAAGAGCCCAGCACCGAATCCCTCAGTGTCATGCTGACGGGAACTGTGGTGTGTGGGACGCCACCAGTCGCATTAACGGGCGCCGAAGTCCTCCTGATCGAGGCTTCACCCAGAGCGGGTGTAAGGCCAGTGCTGGTGCCTCGTTGTGCGGCCGCGAGCGTCTCAGGAGTCGGGTTGTTTGGGAATGCAGCCCAAAGCGGGTGGTAAACTCCATCTAAGGCTAAATACCGGCACGAGTCCGATAGCGGACAAGTACCGTGAGGGAAAGTTGAAAAGAACTTTGAAGAGAGAGTTCAAGAGTACGTGAAACCGCCCAGAGGTAAACGGGTGCATCCGCAAAGTCGGCCCGTGGAATTCAGCGCGGCGCGCGGCCTGGGGTTGCTTCGTCTCGGGATCCCTGGGACCCGGGCGGGGTGCTGCGCCGGGCTCCGCCGCGTGCACTTTCTGCGGGCAGAGCGCCACGACCGGTTTCGCGGCGGCGACGAGCCGGGCGGGAAGGTAGGCGAGGACGGCATCGCTGTTACAGCCCGCCCTGGTGAGCTGCTGCGGGACCGAGGGACGGCCGCGTTCTTCGAGGCCACCCGGCCTTCTCGGGGAGTTCGACTGGTAGAGACTGGGCAACCGTGTCTGCCGACCGCTTCTCGAGACCGGATCGGGCTGGCCGGGAGCGCTGAGGGTCGGTGGCGAGTCTGTCGGCATTGCACCCGACCCGTCTTGAAACACGGACCAAGGAGTCTAACATGCGCGCGAGTCATTGGGTCGTACGAAACTCAAAGGCGCAGTGAAAGCGAGGGTCGCCCCGGGCTGACCCAGGTGGGATCTCTCCGTGCCTCGGCGCGGGGAGCGCACCACCGGCCCGTCCCGTCCGCGTTGTCGGTGGGGCGGAGCAGGAGCGTGCACGCTGGGACCCGAAAGATGGTGAACTATGCCTGAGTAGAACGAAGCCAGAGGAA

>ZSM20090471

NNNNTTCCCCCAGTAACGGCGAGTGAAGCGGGACGAGCCCAGCACCGAATCCCTCAGTGTCACGCTGACGGGAACTGTGGTGTGTGGGACGCCACCAGTCGCATTAAAGGGCGCCGAAGTCCTCCTGATCGAGGCTTCACCCAGAGCGGGTGTAAGGCCAGTGCTGGTGCCTCTTTGTGCGGCCGCGAGCGTCTCAGGAGTCGGGTTGTTTGGGAATGCAGCCCAAAGCGGGTGGTAAACTCCATCTAAGGCTAAATACCGGCACGAGTCCGATAGCGGACAAGTACCGTGAGGGAAAGTTGAAAAGAACTTTGAAGAGAGAGTTCAAGAGTACGTGAAACCGCCCAGAGGTAAACGGGTGCATCCGCAAAGTCGGCCCGTGGAATTCAGCGCGGCGCGCGGCCTGGGGCTGCTCCGCTTCGGGATCCCTGGGACCCGGGCGGGGTGCTGCGCCGGGCTCCGCCGCGTGCACTTTCTGCGGGCAGAGCGCCACGACCGGTTTCGCGGCGGCGACGAGCCGGGCGGGAAGGTATGCTGCTCC-CCG--GCTGTTAGAGCCCGTCCCGGTGAGCTGCTGCGGGACCGAGGGTCGGCCGCGCTCTTCGAGGCCACCCGGCCTTCTCGGGGAGTTCGACTGGCAGCTACTGGGCAACCGTGACTGCCGACCGCTCCTCGAGACCGGATCGGGCTGGCCGAGAGCGCTGAGGGTCTGTGGCGAATCTGTCGGCATTGCACCCGACCCGTCTTGAAACACGGACCAAGGAGTCTAACATGCGCGCGAGTCATTGGGTCGTACGAAACTCAAAGGCGCAGTGAAAGCGAGGGTGGCCCCGGGCCGACCCAGGTGGGATCCCTCCGTCTTCCGGAGCGGGGGGCGCACCACCGGCCCGTCCCGTCCGCGCTGTCGGTGGGGCGGAGCAGGAGCGTGCACGCTGGGACCCGAAAGATGGTGAACTATGCTTGAGTAGAACGAAGCCAGAGGAA

>ZSM20090472

NNNNTTCCCCCAGTAACGGCGAGTGAAGCGGGACGAGCCCAGCACCGAATCCCTCAGTGTCACGCTGACGGGAACTGTGGTGTGTGGGACGCCACCAGTCGCATTAAAGGGCGCCGAAGTCCTCCTGATCGAGGCTTCACCCAGAGCGGGTGTAAGGCCAGTGCTGGTGCCTCTTTGTGCGGCCGCGAGCGTCTCAGGAGTCGGGTTGTTTGGGAATGCAGCCCAAAGCGGGTGGTAAACTCCATCTAAGGCTAAATACCGGCACGAGTCCGATAGCGGACAAGTACCGTGAGGGAAAGTTGAAAAGAACTTTGAAGAGAGAGTTCAAGAGTACGTGAAACCGCCCAGAGGTAAACGGGTGCATCCGCAAAGTCGGCCCGTGGAATTCAGCGCGGCGCGCGGCCTGGGGCTGCTCCGCTTCGGGATCCCTGGGACCCGGGCGGGGTGCTGCGCCGGGCTCCGCCGCGTGCACTTTCTGCGGGCAGAGCGCCACGACCGGTTTCGCGGCGGCNNNNNNNNNNNNNNNNNNNNNNNNNNNNNNNNNNNNNNNNNNNNNNNNNNNNNNNNNNNNNNNNNNNNNNNNNNNNNNNNNNNNNNNNNNNNNNNNNNNNNNNNNNNNNNNNNNNNNNNNNNNNNNNNNNNNNNNNNNNNNNNNNNNNNNNNNNNNNNNNNNNNNNNNNNNNNNNNNNNNNNNNNNNNNNNNNNNNNNNNNNNNNNNNNNNNNNNNNNNNNNNNNNNNNNNNNNNNNNNNNNNNNNNNNNNNNNNNNNNNNNNNNNNNNNNNNNNNNNNNNNNNNNNNNNNNNNNNNNNNNNNNNNNNNNNNNNNNNNNNNNNNNNNNNNNNNNNNNNNNNNNNNNNNNNNNNNNNNNNNNNNNNNNNNNNNNNNNNNNNNNNNNNNNNNNNNNNNNNNNNNNNNNNNNNNNNNNNNNNNNNNNNNNNNNNNNNNNNNNNNNNNNNNNNNNNNNNNNNNNNNNNNNNNNNNNN

>AMC476062001

NNNNTTCCCCCAGTAACGGCGAGTGAAGCGGGAAGAGCCCAGCACCGAATCCCTCAGTGTGATGCTGACGGGAACTGTGGTGTGTGGGACGCCACCAGTCGCATTAAAGGGCGCCGAAGTCCTCCTGATCGAGGCTTCACCCAGAGCGGGTGTAAGGCCAGTGCTGGTGCCTCTTTGTGCGGCCGCGAGCGTCTCAGGAGTCGGGTTGTTTGGGAATGCAGCCCAAAGCGGGTGGTAAACTCCATCTAAGGCTAAATACCGGCACGAGTCCGATAGCGGACAAGTACCGTGAGGGAAAGTTGAAAAGAACTTTGAAGAGAGAGTTCAAGAGTACGTGAAACCGCCCAGAGGTAAACGGGTGCATCCGCAAAGTCGGCCCGTGGAATTCAGCGCGGCGCGCGGCCTGGGGCTGCTTCGTTCCGGGATCCCTGGGACCCGAGCGGGGTGCTGCGCCGGGCTCCGCCGCGTGCACTTTCTGCGGGCAGAGCGCCACGACCGGTTTCGCGGCGGCGACGAGCCGGACGGGAAGGTAGGCGAGGACGGCATCGCTGTTACAGCCCGCCCTGGTGAGCTGCTGCGGGACCGAGGGACGGCCGCGTTCTTCGAGGCCACCCGGCCTTCTCGGGGAGTTCGACTGGTAGAGACTGGGCAACCGTGTCTGCCGACCGCTTCTCGAGACCGGATCGGGCTGGCCGGGAGCGCTGAGGGTCGGTGGCGAATCTGTCGGCATTGCACCCGACCCGTCTTGAAACACGGACCAAGGAGTCTAACATGCGCGCGAGTCATTGGGTCGTACGAAACTCAAAGGCGCAGTGAAAGCGAGGGTCGCCCCGGGCTGACCCAGGTGGGATCTCTCCGTTCCTCGGAGCGGGGAGCGCACCACCGGCCCGTCCCGTCCGCGTTGTCGGTGGGGCGGAGCAGGAGCGTGCACGCTGGGACCCGAAAGATGGTGAACTATGCCTGAGTAGAACGAAGCCAGAGGAA

>ZSM20100389

NNNATTCCCCCAGTAACGGCGAGTGAAGCGGGAAGAGCCCAGCACCGAATCCCTCAGTGTCATGCTGGCGGGAACTGTGGTGTGTGGGACGCCACCAGTCG-ATTAAAGGGCGCCGAAGTCCTCCTGATCGAGGCTTCACCCAGAGCGGGTGTAAGGCCAGTGCTGGTGCCTCTTTGTGCGGCCGCGAGCGTCTCAGGAGTCGGGTTGTTTGGGAATGCAGCCCAAAGCGGGTGGTAAACTCCATCTAAGGCTAAATACCGGCACGAGTCCGATAGCGGACAAGTACCGTGAGGGAAAGTTGAAAAGAACTTTGAAGAGAGAGTTCAAGAGTACGTGAAACCGCCCAGAGGTAAACGGGTGCATCCGCAAAGTCGGCCCGTGGAATTCAGCGCGGCGCGCGGCCTGGGGCTGCTTCGTTCCGGGATCCCTGGGACCCGGGCGGGGTGCTGCGCCGGGCTCCGCCGCGTGCACTTTCTGCGGGCAGAGCGCCACGACCGGTTTCGCGGCGGCGACGAGCCGGGCGGGAAGGTAGGCGAGGGCGGCATCGCTGTTACAGCCCGCCCTGGTGAGCTGCTGCGGGACCGAGGGACGGCCGTGCTCTTGGAGGCCACCCGGCCTTCTCGGGGAGTTCGACTGGTAGAGACTGGGCAACCGTGTCTGCCGACCGCTTCTCGAGACCGGATCGGGCTGGCCGGGAGCGCTGAGGGTCGGTGGCGAATCTGTCGGCATTGCACCCGACCCGTCTTGAAACACGGACCAAGGAGTCTAACATGCGCGCGAGTCATTGGGTCGTACGAAACTCAAAGGCGCAGTGAAAGCGAGGGTCGCCCCGGGCTGACCCAGGTGGGATCTCTCCGCTCC----GGCGGGGAGCACACCACCGGCCCGTCCCGTCCGCGTTGTCGGTGGGGCGGAGCAGGAGCGTGCACGCTGGGACCCGAAAGATGGTGAACTATGCCTGAGTAGAACGAAGCCAGAGGAA

>ZSM20071135

NGGATTCCCCCAGTAACGGCGAGTGAAGCGGGAAGAGCCCAGCACCGAATCCCTCAGTGTCATGCTGGCGGGAACTGTGGTGTGTGGGACGCCACCAGTCGCATTAAAGGGCGCCGAAGTCCTCCTGATCGAGGCTTCACCCAGAGCGGGTGTAAGGCCAGTGCTGGTGCCTCTTTGTGCGGCCGCGAGCGTCTCAGGAGTCGGGTTGTTTGGGAATGCAGCCCAAAGCGGGTGGTAAACTCCATCTAAGGCTAAATACCGGCACGAGTCCGATAGCGGACAAGTACCGTGAGGGAAAGTTGAAAAGAACTTTGAAGAGAGAGTTCAAGAGTACGTGAAACCGCCCAGAGGTAAACGGGTGCATCCGCAAAGTCGGCCCGTGGAATTCAGCGCGGCGCGCGGCCTGGGGCTGCTTCGTTCCGGGATCCCTGGGACCCGGGCGGGGTGCTGCGCCGGGCTCCGCCGCGTGCACTTTCTGCGGGCAGAGCGCCACGACCGGTTTCGCGGCGGCGACGAGCCGGGCGGGAAGGTAGGCGAGGGCGGCATCGCTGTTACAGCCCGCCCTGGTGAGCTGCTGCGGGACCGAGGGACGGCCGTGCTCTTGGAGGCCACCCGGCCTTCTCGGGGAGTTCGACTGGTAGAGACTGGGCAACCGTGTCTGCCGACCGCTTCTCGAGACCGGATCGGGCTGGCCGGGAGCGCTGAGGGTCGGTGGCGAATCTGTCGGCATTGCACCCGACCCGTCTTGAAACACGGACCAAGGAGTCTAACATGCGCGCGAGTCATTGGGTCGTACGAAACTCAAAGGCGCAGTGAAAGCGAGGGTCGCCCCGGGCTGACCCAGGTGGGATCTCTCCGCTCC----GGCGGGGAGCGCACCACCGGCCCGTCCCGTCCGCGTTGTCGGTNNNNNNNNNNNNNNNNNNNNNNNNNNNNNNNNNNNNNNNNNNNNNNNNNNNNNNNNNNNNNNNNNNNNNNNNNNN

>ZSM20080176

AGGATTCCCCCAGTAACGGCGAGTGAAGCGGGAAGAGCCCAGCACCGAATCCCTCAGTGTCATGCTGACGGGAACTGTGGTGTGTGGGACGCCACCAGTCGCATTAAAGGGCGCCGAAGTCCTCCTGATCGAGGCTTCACCCAGAGCGGGTGTAAGGCCAGTGCTGGTGCCTCTTTGTGCGGCCGCGAGCGTCTCAGGAGTCGGGTTGTTTGGGAATGCAGCCCAAAGCGGGTGGTAAACTCCATCTAAGGCTAAATACCGGCACGAGTCCGATAGCGGACAAGTACCGTGAGGGAAAGTTGAAAAGAACTTTGAAGAGAGAGTTCAAGAGTACGTGAAACCGCCCAGAGGTAAACGGGTGCATCCGCAAAGTCGGCCCGTGGAATTCAGCGCGGCGCGCGGCCTGGGGCTGCTTCGTTCCGGGATCCCTGGGACCCGGGCGGGGTGCTGCGCCGGGCTCCGCCGCGTGCACTTTCTGCGGGCAGAGCGCCACGACCGGTTTCGCGGCGGCGACGAGCCGGGCGGGAAGGTAGGCGAGGGCGGCATCGCTGTTACAGCCCGCCCTGGTGAGCTGCTGCGGGACCGAGGGACGGCCGTGCTCTTGGAGGCCACCCGGCCTTCTCGGGGAGTTCGACTGGTAGAGACTGGGCAACCGTGTCTGCCGACCGCTTCTCGAGACCGGATCGGGCTGGCCGGGAGCGCTGAGGGTCGGTGGCGAATCTGTCGGCATTGCACCCGACCCGTCTTGNNNNNNNNNNNNNNNNNNNNNNNNNNNNNNNNNNNNNNNNNNNNNNNNNNNNNNNNNNNNNNNNNNNNNNNNNNNNNNNNNNNNNNNNNNNNNNNNNNNNNNNNNNNNNNNNNNNNNNNNNNNNNNNNNNNNNNNNNNNNNNNNNNNNNNNNNNNNNNNNNNNNNNNNNNNNNNNNNNNNNNNNNNNNNNNNNNNNNNNNNNNNNNNNNNNNNNNNNNNNNNNNNNNNNNNNNNNN

>ZSM20071820

AGGATTCCCCCAGTAACGGCGAGTGAAGCGGGAAGAGCCCAGCACCGAATCCCTCAGTGTCATGCTGACGGGAACTGTGGTGTGTGGGACGCCACCAGTCGCATTAAAGGGCGCCGAAGTCCTCCTGATCGAGGCTTCACCCAGAGCGGGTGTAAGGCCAGTGCTGGTGCCTCTTTGTGCGGCCGCGAGCGTCTCAGGAGTCGGGTTGTTTGGGAATGCAGCCCAAAGCGGGTGGTAAACTCCATCTAAGGCTAAATACCGGCACGAGTCCGATAGCGGACAAGTACCGTGAGGGAAAGTTGAAAAGAACTTTGAAGAGAGAGTTCAAGAGTACGTGAAACCGCCCAGAGGTAAACGGGTGCATCCGCAAAGTCGGCCCGTGGAATTCAGCGCGGCGCGCGGCCTGGGGCTGCTTCGTTCCGGGATCCCTGGGACCCGGGCGGGGTGCTGCGCCGGGCTCCGCCGCGTGCACTTTCTGCGGGCAGAGCGCCACGACCGGTTTCGCGGCGGCGACGAGCCGGGCGGGAAGGTAGGCGAGGGCGGCATCGCTGTTACAGCCCGCCCTGGTGAGCTGCTGCGGGACCGAGGGACGGCCGTGCTCTTGGAGGCCACCCGGCCTTCTCGGGGAGTTCGACTGGTAGAGACTGGGCAACCGTGTCTGCCGACCGCTTCTCGAGACCGGATCGGGCTGGCCGGGAGCGCTGAGGGTCGGTGGCGAATCTGTCGGCATTGCACCCGACCCGTCTTGAAACACGGACCAAGGAGTCTAACATGCGCGCGAGTCATTGGGTCGTACGAAACTCAAAGGCGCAGTGAAAGCGAGGGTCGCCCCGGGCTGACCCAGGTGGGATCTCTCCGCTCC----GGCGGGGAGCGCACCACCGGCCCGTCCCGTCCGCGTTGTCGGTGGGGCGGAGCAGGAGCGTGCACGCTGGGACCCGAAAGATGGTGAACTATGCCTGAGTAGAACGAAGCCAGAGGAA

>ZSM20100390

AGGATTCCCCCAGTAACGGCGAGTGAAGCGGGAAGAGCCCAGCACCGAATCCCTCAGTGTCATGCTGACGGGAACTGTGGTGTGTGGGACGCCACCAGTCGCATTAAAGGGCGCCGAAGTCCTCCTGATCGAGGCTTCACCCAGAGCGGGTGTAAGGCCAGTGCTGGTGCCTCTTTGTGCGGCCGCGAGCGTCTCAGGAGTCGGGTTGTTTGGGAATGCAGCCCAAAGCGGGTGGTAAACTCCATCTAAGGCTAAATACCGGCACGAGTCCGATAGCGGACAAGTACCGTGAGGGAAAGTTGAAAAGAACTTTGAAGAGAGAGTTCAAGAGTACGTGAAACCGCCCAGAGGTAAACGGGTGCATCCGCAAAGTCGGCCCGTGGAATTCAGCGCGGCGCGCGGCCTGGGGCTGCTTCGTTCCGGGATCCCTGGGACCCGGGCGGGGTGCTGCGCCGGGCTCCGCCGCGTGCACTTTCTGCGGGCAGAGCGCCACGACCGGTTTCGCGGCGGCGACGAGCCGGGCGGGAAGGTAGGCGAGGGCGGCATCGCTGTTACAGCCCGCCCTGGTGAGCTGCTGCGGGACCGAGGGACGGCCGTGCTCTTGGAGGCCACCCGGCCTTCTCGGGGAGTTCGACTGGTAGAGACTGGGCAACCGTGTCTGCCGACCGCTTCTCGAGACCGGATCGGGCTGGCCGGGAGCGCTGAGGGTCGGTGGCGAATCTGTCGGCATTGCACCCGACCCGTCTTGAAACACGGACCAAGGAGTCTAACATGCGCGCGAGTCATTGGGTCGTACGAAACTCAAAGGCGCAGTGAAAGCGAGGGTCGCCCCGGGCTGACCCAGGTGGGATCTCTCCGCTCC----GGCGGGGAGCGCACCACCGGCCCGTCCCGTCCGCGTTGTCGGTGGGGCGGAGCAGGAGCGTGCACGCTGGGACCCGAAAGATGGTGAACTATGCCTGAGTAGAACGAAGCCAGAGGAA

>SICBC2010KJ02E01

NNNNNNNNNNNNNNNNNNNNNNNNNNNNNNNNNNNNNNNNNNNNNNNNNNNNNNNNNNNNNNNNNNNNNNNNNNNNNNNNNNNNNNNNNNNNNNNNNNNNNNNNNNNNNNNNNCCGAAGTCCTCCTGATCGAGGCTTCACCCAGAGCGGGTGTAAGGCCAGTGCTGGTGCTTCGCTGTGCGGCCGCGAGCGTCTCAGGAGTCGGGTTGTTTGGGAATGCAGCCCAAAGCGGGTGGTAAACTCCATCTAAGGCTAAATACCGGCACGAGTCCGATAGCGGACAAGTACCGTGAGGGAAAGTTGAAAAGAACTTTGAAGAGAGAGTTCAAGAGTACGTGAAACCGCCCAGAGGTAAACGGGTGCATCCGCAAAGTCGGCCCGTGGAATTCAGCGCGGCGCGCGGCCTGGAGCTGCTTCGTTTCGGGATCCC-GGGACCCGGGCGGGGTGCTGCGCCGGGCCCCGCCGCGTGCACTTTCTGCGGGCAGAGCGCCACGACCGGTTTCGCGGCGGCGACGAGCCGGGCGGGAAGGTAGGCGTCTCCTTCGACGCTGTTATAGACCGCCTCGGTGAGCTGCTGCGGGACCGAGGGACGGCCGCGTTCTTCGAGGCCACCCGGCTTTCTCGGGGAGTTCGACTGGCAGAGACTGAGCAATCGTGTCTGCCGACCGCTTCTCGAG-TCGGTCCGGGCTGGCCGGGAGCGCTCAGGGTCGGTGGCGAATCTGTCGGCATTGCACCCGACCCGTCTTGAAACACGGACCAAGGAGTCTAACATGCGCGCGAGTCATTGGGTTTTTTGAAATCCAAAGGCGCAGTGAAAGCGAGGGTCGCCCCGGGCTGACCCAGGTGGGATCTTTCCGTCTCTNNNNNNNNNNNNNNNNNNNNNNNNNNNNNNNNNNNNNNNNNNNNNNNNNNNNNNNNNNNNNNNNNNNNNNNNNNNNNNNNNNNNNNNNNNNNNNNNNNNNNNNNNNNNNNNNNNNNNNNNN

>ZSM20110722

NNNNNNNNNNNNNNNNNNNNNNNNNNNNNNNNNNNNNNNNNNNNNNNNNNNNNNNNNNNNNNNNNNNNNNNNNNNNNNNNNNNNNNNNNNNNNNNNNNNNNNNNNNNNNNNNNNNNNNNNNNNNNNNNNNNNNNNNNNNNNNNNNNNNNNNNNNNNNNNNNNNNNNNNNNNNNNNNNNNNNNNNNNNNNNNNNNNNNNNNNNNNNNNNNNNNNNNNNNNNNNNNNNNNNNNNNNNNNNNNNNNNNNNNNNNNNNNNNNNNNNNNNNNNNNNNNNNNNNNNNNNNNNNNNNNNNNNNNNNNNNNNNNNNNNNNNNNNNNNNNNNNNNNNNNNNNNNNNNNNNNNNNNNNNNNNNNNNNNNNNCATCCGCAAAGTCGGCCCGTGGAATTCAGCGCGGCGCGCGGCCTGGAGCTGCTTCGTTTCGGGATCCCTGGGACCCGGGCGGGGTGCTGCGCCGGGCCCCGCCGCGTGCACTTTCTGCGGGCAGAGCGCCACGACCGGTTTCGCGGCGGCGACGAGCCGGGCGGGAAGGTAGGCGTCTCCTTCGACGCTGTT--TGACCGCCTCGGTGAGCTGCTGCGGGACCGAGGGACGGCCGCGTTCTTCGAGGCCACCCGGCTTTCTCGGGGAGTTCGACTGGCAGAGACTGAGCAATCGTGTCTGCCGACCGCTTCTCGAG-TCGGTCCGGGCTGGCCGGGAGCGCTCAGGGTCGGTGGCGAATCTGTCGGCATTGCACCCGACCCGTCTTGAAACACGGACCAAGGAGTCTAACATGCGCGCGAGTCATTGGGTTCTACGAAATCCAAAGGCGCAGTGAAAGCGAGGGTCGCCCCGGGCTGACCCAGGTGGGATCTTTCCGTCTCTCGGAGCGGGGAGC-CCCCACCGGCCCGTCCCGTCCGCGTCGTCGGTGGGGCGGAGCAGGAGCGTGCACGCTGGGACCCGAAAGATGGTGAACTATGCCTGAGTAGAACGAAGCCAGAGGAA

>ZSM20110723

NNNNNNNNNNNNNNNNNNNNNNNNNNNNNNNNNNNNNNNNNNNNNNNNNNNNNNNNNNNNNNNNNNNNNNNNNNNNNNNNNNNNNNNNNNNNNNNNNNNNNNNNNNNNNNNNNNNNNNNNNNNNNNNNNNNNNNNNNNNNNNNNNNNNNNNNNNNNNNNNNNNNNNNNNNNNNNNNNNNNNNNNNNNNNNNNNNNNNNNNNNNNNNNNNNNNNNNNNNNNNNNNNNNNNNNNNNNNNNNNNNNNNNNNNNNNNNNNNNNNNNNNNNAGTCCGATAGCGGACAAGTACCGTGAGGGAAAGTTGAAAAGAACTTTGAAGAGAGAGTTCAAGAGTACGTGAAACCGCCCAGAGGTAAACGGGTGCATCCGCAAAGTCGGCCCGTGGAATTCAGCGCGGCGCGCGGCCTGGAGCTGCTTCGTTTCGGGATCCCTGGGACCCGGGCGGGGTGCTGCGCCGGGCCCCGCCGCGTGCACTTTCTGCGGGCAGAGCGCCACGACCGGTTTCGCGGCGGCGACGAGCCGGGCGGGAAGGTAGGCGTCTCCTTCGACGCTGTTATAGACCGCCTCGGTGAGCTGCTGCGGGACCGAGGGACGGCCGCGTTCTTCGAGGCCACCCGGCTTTCTCGGGGAGTTCGACTGGCAGAGACTGAGCAATCGTGTCTGCCGACCGCTTCTCGAG-TCGGTCCGGGCTGGCCGGGAGCGCTCAGGGTCGGTGGCGAATCTGTCGGCATTGCACCCGACCCGTCTTGAAACACGGACCAAGGAGTCTAACATGCGCGCGAGTCATTGGGTTCTACGAAATCCAAAGGCGCAGTGAAAGCGAGGGTCGCCCCGGGCTGACCCAGGTGGGATCTTTCCGTCTCCTGGAGCGGGGAGC-CACCACCGGCCC-TCCCGTCCGCGTCGTCGGTGGGGCGGAGCAGGAGCGTGCACGCTGGGACCCGAAAGATGGTGAACTATGCCTGAGTAGAACGAAGCCAGAGGAA

>SICBC2010KJ01E03

NNNATTCCCCCAGTAACGGCGAGTGAAGCGGGAAGAGCCCAGCACCGAATCCCTCAGTGTCATGCTGGCGGGAACTGTGGTGTGTGGGACGCCACCAGTCG-ACATGCGGGCGCCGAAGTCCTCCTGATCGAGGCTTCACCCAGAGCGGGTGTAAGGCCAGTGCTGGTGCTTCGCTGTGCGGCCGCGAGCGTCTCAGGAGTCGGGTTGTTTGGGAATGCAGCCCAAAGCGGGTGGTAAACTCCATCTAAGGCTAAATACCGGCACGAGTCCGATAGCGGACAAGTACCGTGAGGGAAAGTTGAAAAGAACTTTGAAGAGAGAGTTCAAGAGTACGTGAAACCGCCCAGAGGTAAACGGGTGCATCCGCAAAGTCGGCCCGTGGAATTCAGCGCGGCGCGCGGCCTGGAGCTGCTTCGTTTCGGGATCCCTGGGACCCGGGCGGGGTGCTGCGCCGGGCCCCGCCGCGTGCACTTTCTGCGGGCAGAGCGCCACGACCGGTTTCGCGGCGGCGACGAGCCGGGCGGGAAGGTAGGCGTCTCCTTCGACGCTGTTATAGACCGCCTCGGTGAGCTGCTGCGGGACCGAGGGACGGCCGCGTTCTTCGAGGCCACCCGGCTTTCTCGGGGAGTTCGACTGGCAGAGACTGAGCAATCGTGTCTGCCGACCGCTTCTCGAG-TCGGTCCGGGCTGGCCGGGAGCGCTCAGGGTCGGTGGCGAATCTGTCGGCATTGCACCCGACCCGTCTTGAAACACGGACCAAGGAGTCTAACATGCGCGCGAGTCATTGGGTTCTACGAAATCCAAAGGCGCAGTGAAAGCGAGGGTCGCCCCGGGCTGACCCAGGTGGGATCTTTCCGTCTCTCGGAGCGGGGAGCGCACCACCGGCCCGTCCCGTCCGNNNNNNNNNNNNNNNNNNNNNNNNNNNNNNNNNNNNNNNNNNNNNNNNNNNNNNNNNNNNNNNNNNNNNNNNNNNNNNNNNNNNN

>SICBC2010KJ01D07

NNNNTTCCCCCAGTAACGGCGAGTGAAGCGGGAAGAGCCCAGCACCGAATCCCTCAGCGTCATGCTGACGGGAACTGTGGTGTGTGGGACGCCACCAGTCGCACATGCGGGCGCCGAAGTCCTCCTGATCGAGGCTTCACCCAGAGCGGGTGTAAGGCCAGTGCTGGTGCTTCGCTGTGCGGCCGCGAGCGTCTCAGGAGTCGGGTTGTTTGGGAATGCAGCCCAAAGCGGGTGGTAAACTCCATCTAAGGCTAAATACCGGCACGAGTCCGATAGCGGACAAGTACCGTGAGGGAAAGTTGAAAAGAACTTTGAAGAGAGAGTTCAAGAGTACGTGAAACCGCCCAGAGGTAAACGGGTGCATCCGCAAAGTCGGCCCGTGGAATTCAGCGCGGCGCGCGGCCTGGAGCTGCTTCGTTTCGGGATCCCTGGGACCCGGGCGGGGTGCTGCGCCGGGCCCCGCCGCGTGCACTTTCTGCGGGCAGAGCGCCACGACCGGTTTCGCGGCGGCGACGAGCCGGGCGGGAAGGTAGGCGTCTCCTTCGACGCTGTTATAGACCGCCTCGGTGAGCTGCTGCGGGACCGAGGGACGGCCGCGTTCTTCGAGGCCACCCGGCTTTCTCGGGGAGTTCGACTGGCAGAGACTGAGCAATCGTGTCTGCCGACCGCTTCTCGAG-TCGGTCCGGGCTGGCCGGGAGCGCTCAGGGTCGGTGGCGAATCTGTCGGCATTGCACCCGACCCGTCTTGAAACACGGACCAAGGAGTCTAACATGCGCGCGAGTCATTGGGTTCTACGAAATCCAAAGGCGCAGTGAAAGCGAGGGTCGCCCCGGGCTGACCCAGGTGGGATCTTTCCGTCTCTCGGAGCGGGGAGC-CACCACCGGCCCGTCCCGTCCGCGTCGTCGGTGGGGCGGAGCAGGAGCGTGCACGCTGGGACCCGAAAGATGGTGAACTATGCCTGAGTAGAACGAAGCCAGAGGAA

>SICBC2010KJ01B09

AGGATTCCCCCAGTAACGGCGAGTGAAGCGGGAAGAGCCCAGCACCGAATCCCTCAGTGTCATGCTGACGGGAACTGTGGTGTGTGGGACGCCACCAGTCGCACATGCGGGCGCCGAAGTCCTCCTGATCGAGGCTTCACCCAGAGCGGGTGTAAGGCCAGTGCTGGTGCTTCGCTGTGCGGCCGCGAGCGTCTCAGGAGTCGGGTTGTTTGGGAATGCAGCCCAAAGCGGGTGGTAAACTCCATCTAAGGCTAAATACCGGCACGAGTCCGATAGCGGACAAGTACCGTGAGGGAAAGTTGAAAAGAACTTTGAAGAGAGAGTTCAAGAGTACGTGAAACCGCCCAGAGGTAAACGGGTGCATCCGCAAAGTCGGCCCGTGGAATTCAGCGCGGCGCGCGGCCTGGAGCTGCTTCGTTTCGGGATCCCTGGGACCCGGGCGGGGTGCTGCGCCGGGCCCCGCCGCGTGCACTTTCTGCGGGCAGAGCGCCACGACCGGTTTCGCGGCGGCGACGAGCCGGGCGGGAAGGTAGGCGTCTCCTTCGACGCTGTTATAGACCGCCACGGTGAGCTGCTGCGGGACCGAGGGACGGCCGCGTTCTTCGAGGCCACCCGGCTTTCTCGGGGAGTTCGACTGGCAGAGACTGAGCAATCGTGTCTGCCGACCGCTTCTCGAG-TCGGTCCGGGCTGGCCGGGAGCGCTCAGGGTCGGTGGCGAATCTGTCGGCATTGCACCCGACCCGTCTTGAAACACGGACCAAGGAGTCTAACATGCGCGCGAGTCATTGGGTTCTACGAAATCCAAAGGCGCAGTGAAAGCGAGGGTCGCCCCGGGCTGACCCAGGTGGGATCTTTCCGTCTCTCGGAGCGGGGAGCGCACCACCGGCCCGTCCCGTCCGCGTCGTCGGTGGGGCGGAGCAGGAGCGTGCACGCTGGGACCCGAAAGATGGTGAACTATGCCTGAGTAGAACGAAGCCAGANNNN

>ZSM20090198

AGGATTCCCCCAGTAACGGCGAGTGAAGCGGGAAGAGCCCAGCACCGAATCCCTCAGTGTCATGCTGACGGGAACTGTGGTGTGTGGGACGCCACCAGTCGCACATGCGGGCGCCGAAGTCCTCCTGATCGAGGCTTCACCCAGAGCGGGTGTAAGGCCAGTGCTGGTGCTTCGCTGTGCGGCCGCGAGCGTCTCAGGAGTCGGGTTGTTTGGGAATGCAGCCCAAAGCGGGTGGTAAACTCCATCTAAGGCTAAATACCGGCACGAGTCCGATAGCGGACAAGTACCGTGAGGGAAAGTTGAAAAGAACTTTGAAGAGAGAGTTCAAGAGTACGTGAAACCGCCCAGAGGTAAACGGGTGCATCCGCAAAGTCGGCCCGTGGAATTCAGCGCGGCGCGCGGCCTGGAGCTGCTTCGTTTCGGGATCCCTGGGACCCGGGCGGGGTGCTGCGCCGGGCCCCGCCGCGTGCACTTTCTGCGGGCAGAGCGCCACGACCGGTTTCGCGGCGGCGACGAGCCGGGCGGGAAGGTAGGCGTCTCCTTCGACGCTGTTATAGACCGCCACGGTGAGCTGCTGCGGGACCGAGGGACGGCCGCGTTCTTCGAGGCCACCCGGCTTTCTCGGGGAGTTCGACTGGCAGAGACTGAGCAATCGTGTCTGCCGACCGCTTCTCGAG-TCGGTCCGGGCTGGCCGGGAGCGCTCAGGGTCGGTGGCGAATCTGTCGGCATTGCACCCGACCCGTCTTGAAACACGGACCAAGGAGTCTAACATGCGCGCGAGTCATTGGGTTCTACGAAATCCAAAGGCGCAGTGAAAGCGAGGGTCGCCCCGGGCTGACCCAGGTGGGATCTTTCCGTCTCTCGGAGCGGGGAGCGCACCACCGGCCCGTCCCGTCCGCGTCGTCGGTGGGGCGGAGCAGGAGCGTGCACGCTGGGACCCGAAAGATGGTGAACTATGCCTGAGTAGAACGAAGCCAGAGGAA

>SICBC2010KJ01B07

AGGATTCCCCCAGTAACGGCGAGTGAAGCGGGAAGAGCCCAGCACCGAATCCCTCAGTGTCATGCTGACGGGAACTGTGGTGTGTGGGACGCCACCAGTCGCACATGCGGGCGCCGAAGTCCTCCTGATCGAGGCTTCACCCAGAGCGGGTGTAAGGCCAGTGCTGGTGCTTCGCTGTGCGGCCGCGAGCGTCTCAGGAGTCGGGTTGTTTGGGAATGCAGCCCAAAGCGGGTGGTAAACTCCATCTAAGGCTAAATACCGGCACGAGTCCGATAGCGGACAAGTACCGTGAGGGAAAGTTGAAAAGAACTTTGAAGAGAGAGTTCAAGAGTACGTGAAACCGCCCAGAGGTAAACGGGTGCATCCGCAAAGTCGGCCCGTGGAATTCAGCGCGGCGCGCGGCCTGGAGCTGCTTCGTTTCGGGATCCCTGGGACCCGGGCGGGGTGCTGCGCCGGGCCCCGCCGCGTGCACTTTCTGCGGGCAGAGCGCCACGACCGGTTTCGCGGCGGCGACGAGCCGGGCGGGAAGGTAGGCGTCTCCTTCGACGCTGTTATAGACCGCCTCGGTGAGCTGCTGCGGGACCGAGGGACGGCCGCGTTCTTCGAGGCCACCCGGCTTTCTCGGGGAGTTCGACTGGCAGAGACTGAGCAATCGTGTCTGCCGACCGCTTCTCGAG-TCGGTCCGGGCTGGCCGGGAGCGCTCAGGGTCGGTGGCGAATCTGTCGGCATTGCACCCGACCCGTCTTGAAACACGGACCAAGGAGTCTAACATGCGCGCGAGTCATTGGGTTCTACGAAATCCAAAGGCGCAGTGAAAGCGAGGGTCGCCCCGGGCTGACCCAGGTGGGATCTTTCCGTCTCTCGGAGCGGGGAGCGCACCACCGGCCCGTCCCGTCCGCGTCGTCGGTGGGGCGGAGCAGGAGCGTGCACGCTGGGACCCGAAAGATGGTGAACTATGCCTGAGTAGAACGAAGCCAGANNNN

>SICBC2010KJ01C09

AGGATTCCCCCAGTAACGGCGAGTGAAGCGGGAAGAGCCCAGCACCGAATCCCTCAGTGTCATGCTGACGGGAACTGTGGTGTGTGGGACGCCACCAGTCGCACATGCGGGCGCCGAAGTCCTCCTGATCGAGGCTTCACCCAGAGCGGGTGTAAGGCCAGTGCTGGTGCTTCGCTGTGCGGCCGCGAGCGTCTCAGGAGTCGGGTTGTTTGGGAATGCAGCCCAAAGCGGGTGGTAAACTCCATCTAAGGCTAAATACCGGCACGAGTCCGATAGCGGACAAGTACCGTGAGGGAAAGTTGAAAAGAACTTTGAAGAGAGAGTTCAAGAGTACGTGAAACCGCCCAGAGGTAAACGGGTGCATCCGCAAAGTCGGCCCGTGGAATTCAGCGCGGCGCGCGGCCTGGAGCTGCTTCGTTTCGGGATCCCTGGGACCCGGGCGGGGTGCTGCGCCGGGCCCCGCCGCGTGCACTTTCTGCGGGCAGAGCGCCACGACCGGTTTCGCGGCGGCGACGAGCCGGGCGGGAAGGTAGGCGTCTCCTTCGACGCTGTTATAGACCGCCTCGGTGAGCTGCTGCGGGACCGAGGGACGGCCGCGTTCTTCGAGGCCACCCGGCTTTCTCGGGGAGTTCGACTGGCAGAGACTGAGCAATCGTGTCTGCCGACCGCTTCTCGAG-TCGGTCCGGGCTGGCCGGGAGCGCTCAGGGTCGGTGGCGAATCTGTCGGCATTGCACCCGACCCGTCTTGAAACACGGACCAAGGAGTCTAACATGCGCGCGAGTCATTGGGTTCTACGAAATCCAAAGGCGCAGTGAAAGCGAGGGTCGCCCCGGGCTGACCCAGGTGGGATCTTTCCGTCTCTCGGAGCGGGGAGCGCACCACCGGCCCGTCCCGTCCGCGTCGTCGGTGGGGCGGAGCAGGAGCGTGCACGCTGGGACCCGAAAGATGGTGAACTATGCCTGAGTAGAACGAAGCCAGAGGAA

>ZSM20080565

AGGATTCCCCCAGTAACGGCGAGTGAAGCGGGAAGAGCCCAGCACCGAATCCCTCAGTGTCATGCTGACGGGAACTGTGGTGTGTGGGACGCCACCAGTCGCACATGCGAGCGCCGAAGTCCTCCTGATCGAGGCTTCACCCAGAGCGGGTGTAAGGCCAGTGCTGGTGCTTCGCTGTGCGGCCGCGAGCGTCTCAGGAGTCGGGTTGTTTGGGAATGCAGCCCAAAGCGGGTGGTAAACTCCATCTAAGGCTAAATACCGGCACGAGTCCGATAGCGGACAAGTACCGTGAGGGAAAGTTGAAAAGAACTTTGAAGAGAGAGTTCAAGAGTACGTGAAACCGCCCAGAGGTAAACGGGTGCATCCGCAAAGTCGGCCCGTGGAATTCAGCGCGGCGTTCGGTCTGGAA-TGGTTCGTTTCGGGATCCCTGGGACTCGGGCGGG--CCAGAACCGGACCTCGCCGCGTGCACTTTCCGCGGGCAGAGCGCCACGACCGGTTTCGCGGCGGCGACGAGCCGGGCGGGAAGGTAGGCGTCTCCTCCGACGCTGTTATAGACCGCTCTGGTGAGCTGCTGCGGGACCGAGGGACGGCCGCGTTCTTCGAGGCCACCCGGCTTTCTCGGGGAGTTCGACTGGCAGAGACTGGGCAACCGTGTCTGCCGACCGCTTTCCGGGACCGGACCGGGCTGGCCGGGAGCGCTGAGGGTCGGTGGCGAATCTGTCGGCATTGCACCCGACCCGTCTTGAAACACGNNNNNNNNNNNNNNNNNNNNNNNNNNNNNNNNNNNNNNNNNNNNNNNNNNNNNNNNNNNNNNNNNNNNNNNNNNNNNNNNNNNNNNNNNNNNNNNNNNNNNNNNNNNNNNNNNNNNNNNNNNNNNNNNNNNNNNNNNNNNNNNNNNNNNNNNNNNNNNNNNNNNNNNNNNNNNNNNNNNNNNNNNNNNNNNNNNNNNNNNNNNNNNNNNNNNNNNNNNNNNNNNNNNNN

>ZSM20071381

NNNNNNNNNNNNNNNNNNNNNNNNNNNNNNNNNNNNNNNNNNNNNNNNNNNNNNNNNNNNNNNNNNNNNNNNNNNNNNNNNNNNNNNNNNNNNNNNNNNNNNNNNNNNNNNNNNNNNNNNNNNNNNNNNNNNNNNNNNNNNNNNNNNNNNNNNNNNNNNNNNNNNNNNNNNNNNNNNNNNNNNNNNNNNNNNNNNNNNNNNNNNNNNNNNNNNNNNNNNNNNNNNNNNNNNNNNNNNNNNNNNNNNNNNNNNNNNNNNNNNNNNNNNNNNNNNNNNNNNNNNNNNNNNNNNNNNNNNNNNNNNNNNNNNNNNNNNNNNNNNNNNNNNNNNNNNNNNNNNNNNNNNNNNNNNNNNNNNNNNNNNNNNNNNNNNNNNNNNNNNNNNNNNNNNNNNNNNNNNNNNNNNNNNNNNNNNNNNNNNNNNNNNNNNNNNNNNNNNNNNNNNNNNNNNNNNNNNNNNNNNNNNNNNNNNNNNNNNNNNNNNNNNNNNNNNNNNNNNNNNNNNNNNNNNNNNNNNNNNNNNNNNNAAGGTAGGCGTCTCCTTCGACGCTGTTATAGACCGCCCTGGTGAGCTGCTGCGGGACCGAGGGACGGCCGCGTTCTTCGAGGCCACCCGGCTTTCTCGGGGAGTTCGACTGGCAGAGACTGGGCAACCGTGTCTGCCGACCGCTTTCCGGGACCGGACCGGGCTGGCCGGGAGCGCTGAGGGTCGGTGGCGAATCTGTCGGCATTGCACCCGACCCGTCTTGAAACACGGACCAAGGAGTCTAACATGCGCGCGAGTCATTGGGTTGTACGAAACCCAAAGGCGCAGTGAAAGCGAGGGTCGCCCCGGGCTGACCCAGGTGGGATCTTTCCGTTCTTCGGAGCGGGGAGCGCACCACCGGCCCGTCCCGTCCGCGTTGTCGGTGGGGCGGAGCAGGAGCGTGCACGCTGGGACCCGAAAGATGGTGAACTATGCCTGAGTAGAACGAAGCCAGAGGAA

>ZSM20080054

NNNNNTCCCCCAGTAACGGCGAGTGAAGCGGGAAGAGCCCAGCACCGAATCCCTCAGTGTCATGCTGACGGGAACTGTGGTGTGTGGGACGCCACCAGTCGCACATGCGGGCGCCGAAGTCCTCCTGATCGAGGCTTCACCCAGAGCGGGTGTAAGGCCAGTGCTGGTGCTTCGCTGTGCGGCCGCGAGCGTCTCAGGAGTCGGGTTGTTTGGGAATGCAGCCCAAAGCGGGTGGTAAACTCCATCTAAGGCTAAATACCGGCACGAGTCCGATAGCGGACAAGTACCGTGAGGGAAAGTTGAAAAGAACTTTGAAGAGAGAGTTCAATAGTACGTGAAACCGCCCAGAGGTAAACGGGTGCATCCGCAAAGTCGGCCCGTGGAATTCAGCGCGGCGCGCGGCCTGGGGCTGCTTCGTTCCGGGATCCCTGGGACCCGAGCGGGGTGCTGCGCCGGGCTCCGCCGCGTGCACTTTCTGCGGGCAGAGCGCCACGACCGGTTTCGCGGCGGCGACGAGCCGGGCGGGAAGGTAGGCGTCTCCTTCGACGCTGTTATAGACCGCCCTGGTGAGCTGCTGCGGGACCGAGGGACGGCCGCGTTCTTCGAGGCCACCCGGCTTTCTCGGGGAGTTCGACTGGCAGAGACTGGGCAACCGTGTCTGCCGACCGCTTTCCGGGACCGGACCGGGCTGGCCGGGAGCGCTGAGGGTCGGTGGCGAATCTGTCGGCATTGCACCCGACCCGTCTTGAAACACGGACCAAGGAGTCTAACATGCGCGCGAGTCATTGGGTTGTACGAAACCCAAAGGCGCAGTGAAAGCGAGGGTCGCCCCGGGCTGACCCAGGTGGGATCTTTCCGTTCTCCGGAGCGGGGAGCGCACCACCGGCCCGTCCCGTCCGCGTTGTCGGTGGGGCGGAGCAGGAGCGTGCACGCTGGGACCCGAAAGATGGTGAACTATGCCTGAGTAGAACGAAGCCAGANNNN

>AMC476054001

AGGATTCCCCCAGTAACGGCGAGTGAAGCGGGAAGAGCCCAGCACCGAATCCCTCAGTGTCATGCTGACGGGAACTGTGGTGTGTGGGACGCCACCAGTCGCACATGCGGGCGCCGAAGTCCTCCTGATCGAGGCTTCACCCAGAGCGGGTGTAAGGCCAGTGCTGGTGCTTCGCTGTGCGGCCGCGAGCGTCTCAGGAGTCGGGTTGTTTGGGAATGCAGCCCAAAGCGGGTGGTAAACTCCATCTAAGGCTAAATACCGGCACGAGTCCGATAGCGGACAAGTACCGTGAGGGAAAGTTGAAAAGAACTTTGAAGAGAGAGTTCAAGAGTACGTGAAACCGCCCAGAGGTAAACGGGTGCATCCGCAAAGTCGGCCCGTGGAATTCAGCGCGGCGCGCGGCCTGGAGCTGCTTCGTTTCGGGATCCCTGGGACCCGGGCGGGGTGCTGCGCCGGGCTCCGCCGCGTGCACTTTCTGCGGGCAGAGCGCCACGACCGGTTTCGCGGCGGCGACGAGCCGGGCGGGAAGGTAGGCGTCCGCTTCGACGCTGTTATAGACCGCCCTGGTGAGCTGCTGCGGGACCGAGGGACGGCCGCGCTCTTCGAGGCCACCCGGCTTTCTAGGGGAGTTCGACTGGCAGAGACTGGGCAACCGTGTCTGCCGACCGCTCCTCGAGACCGGACCGGGCTGGCCGGGAGCGCTGAGGGTCTGTGGCGAATCTGTCGGCATTGCACCCGACCCGTCTTGAAACACGGACCAAGGAGTCTAACATGCGCGCGAGTCATTGGGTTGTACGAAACCCAAAGGCGCAGTGAAAGCGAGGGTCGCCCCGGGCTGACCCAGGTGGGATCTTTCCGTCCCTCGGGGCGGGGAGCGCACCACCGGCCCGTCCCGTCCGCGTTGTCGGTGGGGCGGAGCAGGAGCGTGCACGCTGGGACCCGAAAGATGGTGAACTATGCCTGAGTAGAACGAAGCCAGAGAAN

>ZSM20100592

AGGATTTCCCCAGTAACGGCGAGTGAAGCGGGAAGAGCCCAGCACCGAATCCCTCAGTGTCATGCTGACGGGAACTGTGGTGTGTGGGACGCCACCAGTCGCACATGCGGGCGCCGAAGTCCTCCTGATCGAGGCTTCACCCAGAGCGGGTGTAAGGCCAGTGCTGGTGCTTCGCTGTGCGGCCGCGAGCGTCTCAGGAGTCGGGTTGTTTGGGAATGCAGCCCAAAGCGGGTGGTAAACTCCATCTAAGGCTAAATACCGGCACGAGTCCGATAGCGGACAAGTACCGTGAGGGAAAGTTGAAAAGAACTTTGAAGAGAGAGTTCAAGAGTACGTGAAACCGCCCAGAGGTAAACGGGTGCATCCGCAAAGTCGGCCCGTGGAATTCAGCGCGGCGCGCGGCCCGGGGCTGCCTCGCTTCGGGATCCCTGGGACCCGAGCGGGGTGCCGCGCCGGGCTCCGCCGCGTGCACTTTCTGCGGGCAGAGCGCCACGACCGGTTTCGCGGCGGCGACGAGCCGGGCGGGAAGGTAGGCGTCAGCTTCGACGCTGTTACAGACCGCCCTGGTGAGCTGCTGCGGGACCGAGGGACGGCCGCGTTCTTCGAGGCCACCCGGCTTTCTCGGGGAGTTCGACTGGCAGAGACTGGGCAACCGTGTCTGCCGACCGCTTCTCGAGACGGGACCGGGCTGGCCGGGAGCGCTGAGGGTCGGTGGCGAATCTGTCGGCATTGCACCCGACCCGTCTTGAAACACGGACCAAGGAGTCTAACATGCGCGCGAGTCATTGAGTTGTACGAAACCCAAAGGCGCAGTGAAAGCGAGGGTCGCCCCGGGCTGACCCAGGTGGGATCTTTCCGTTCCTCGGAGCGGGGAGCGCACCACCGGCCCGTCCCGTCCGCGTCGTCGGTGGGGCGGAGCAGGAGCGTGCACGCTGGGACCCGAAAGATGGTGAACTATGCTTGAGTAGAACGAAGCCAGAGAAN

>ZSM20081014

AGGATTCCCCCAGTAACGGCGAGTGAAGCGGGAAGAGCCCAGCACCGAATCCCTCAGTGTCATGCTGACGGGAACTGTGGTGTGTGGGACGCCACCAGTCGCACATGCGGGCGCCGAAGTCCTCCTGATCGAGGCTTCACCCAGAGCGGGTGTAAGGCCAGTGCTGGTGCTTCGCTGTGCGGCCGCGAGCGTCTCAGGAGTCGGGTTGTTTGGGAATGCAGCCCAAAGCGGGTGGTAAACTCCATCTAAGGCTAAATACCGGCACGAGTCCGATAGCGGACAAGTACCGTGAGGGAAAGTTGAAAAGAACTTTGAAGAGAGAGTTCAAGAGTACGTGAAACCGCCCAGAGGTAAACGGGTGCATCCGCAAAGTCGGCCCGTGGAATTCAGCGCGGCGCGCGGCCTGGGGCTGCTTCGTTTCGGGATCCCTGGGACCCGAGCGGGGTGCCGCGCCGGGCTCCGCCGCGTGCACTTTCTGCGGGCAGAGCGCCACGACCGGTTTCGCGGCGGCGACGAGCCGGGCGGGAAGGTAGGCGTCAGCTTCGACGCTGTTATAGACCGTCCTGGTGAGCTGCTGCGGGACCGAGGGACGGCCGCGTTCTTCGAGGCCACCCGGCTTTCTCGGGGAGTTCGACTGGCAGAGACTGGGCAACCGTGTCTGCCGACCGCTTCTCGAGACCGGACCGGGCTGGCCGGGAGCGCTGAGGGTCGGTGGCGAATCTGTCGGCATTGCACCCGACCCGTCTTGAAACACGGACCAAGGAGTCTAACATGCGCGCGAGTCATTGGGTTGTACGAAACCCAAAGGCGCAGTGAAAGCGAGGGTCGCCCCGGGCTGACCCAGGTGGGATCTTTCCGTCTCCTGGAGCGGGGAGCGCACCACCGGCCCGTCCCGTCCGCGTCGTCGGTGGGGCGGAGCAGGAGCGTGCACGCTGGGACCCGAAAGATGGTGAACTATGCCTGAGTAGAACGAAGCCAGAGGAA

>ZSM20100379

AGGATTCCCCCAGTAACGGCGAGTGAAGCGGGAAGAGCCCAGCACCGAATCCCTCAGTGTCATGCTGACGGGAACTGTGGTGTGTGGGACGCCACCAGTCGCACATGCGGGCGCCGAAGTCCTCCTGATCGAGGCTTCACCCAGAGCGGGTGTAAGGCCAGTGCTGGTGCTTCGCTGTGCGGCCGCGAGCGTCTCAGGAGTCGGGTTGTTTGGGAATGCAGCCCAAAGCGGGTGGTAAACTCCATCTAAGGCTAAATACCGGCACGAGTCCGATAGCGGACAAGTACCGTGAGGGAAAGTTGAAAAGAACTTTGAAGAGAGAGTTCAAGAGTACGTGAAACCGCCCAGAGGTAAACGGGTGCATCCGCAAAGTCGGCCCGTGGAATTCAGCGCGGCGCGCGGCCTGGGGCTGCTTCGTTTCGGGATCCCTGGGACCCGAGCGGGGTGCCGCGCCGGGCTCCGCCGCGTGCACTTTCTGCGGGCAGAGCGCCACGACCGGTTTCGCGGCGGCGACGAGCCGGGCGGGAAGGTAGGCGTCAGCTTCGACGCTGTTATAGACCGTCCTGGTGAGCTGCTGCGGGACCGAGGGACGGCCGCGTTCTTCGAGGCCACCCGGCTTTCTCGGGGAGTTCGACTGGCAGAGACTGGGCAACCGTGTCTGCCGACCGCTTCTCGAGACCGGACCGGGCTGGCCGGGAGCGCTGAGGGTCGGTGGCGAATCTGTCGGCATTGCACCCGACCCGTCTTGAAACACGGACCAAGGAGTCTAACATGCGCGCGAGTCATTGGGTTGTACGAAACCCAAAGGCGCAGTGAAAGCGAGGGTCGCCCCGGGCTGACCCAGGTGGGATCTTTCCGTCTCTTGGAGCGGGGAGCGCACCACCGGCCCGTCCCGTCCGCGTCGTCGGTGGGGCGGAGCAGGAGCGTGCACGCTGGGACCCGAAAGATGGTGAACTATGCCTGAGTAGAACGAAGCCAGAGNNN

>AMC476051001

AGGATTCCCCCAGTAACGGCGAGTGAAGCGGGAAGAGCCCAGCACCGAATCCCTCAGTGTCATGCTGACGGGAACTGTGGTGTGTGGGACGCCACCAGTCGCACATGCGGGCGCCGAAGTCCTCCTGATCGAGGCTTCACCCAGAGCGGGTGTAAGGCCAGTGCTGGTGCTTCGCTGTGCGGCCGCGAGCGTCTCAGGAGTCGGGTTGTTTGGGAATGCAGCCCAAAGCGGGTGGTAAACTCCATCTAAGGCTAAATACCGGCACGAGTCCGATAGCGGACAAGTACCGTGAGGGAAAGTTGAAAAGAACTTTGAAGAGAGAGTTCAAGAGTACGTGAAACCGCCCAGAGGTAAACGGGTGCATCCGCAAAGTCGGCCCGTGGAATTCAGCGCGGCGCGCGGCCTGGGGCTGCTCCGTTTCGGGATCCCTGGGACCCGAGCGGGGTGCCGCGCCGGGCTCCGCCGCGTGCACTTTCTGCGGGCAGAGCGCCACGACCGGTTTCGCGGCGGCGACGAGCCGGGCGGGAAGGTAGGCGTCAGCTTCGACGCTGTTATAGACCGCCCTGGTGAGCTGCTGCGGGACCGAGGGACGGCCGCGTTCTTCGAGGCCTCCCGGCTTTCTCGGGGAGTTCGACTGGCAGANNNNNNNNNNNNNNNNNNNNNNNNNNNNNNNNNNNNNNNNNNNNNNNNNNNNNNNNNNNNNNNNNNNNNNNNNNNNNNNNNNNNNNNNNNNNNNNNNNNNNNNNNNNNNNNNNNNNNNNNNNNNNNNNNNNNNNNNNNNNNNNNNNNNNNNNNNNNNNNNNNNNNNNNNNNNNNNNNNNNNNNNNNNNNNNNNNNNNNNNNNNNNNNNNNNNNNNNNNNNNNNNNNNNNNNNNNNNNNNNNNNNNNNNNNNNNNNNNNNNNNNNNNNNNNNNNNNNNNNNNNNNNNNNNNNNNNNNNNNNNNNNNNNNNNNNNNNNNNNNNNNNNNNNNNNNNNNNNNNNNN
