# Supplementary material for: How to describe a cryptic species? Practical challenges of molecular taxonomy
Source: Front Zool. 2013 Sep 27;10:59. doi: 10.1186/1742-9994-10-59 (PMC4015967; doi:10.1186/1742-9994-10-59)
Supplement: Additional file 5 — 16S rRNA alignment of Pontohedyle (fasta format). The alignment was generated with MUSCLE [107] and ambiguous parts of the alignment were masked with Gblocks [108] (settings for a less stringent selection). [file 1742-9994-10-59-S5.docx]

### Additional file 5 – 16S rRNA alignment of *Pontohedyle* (fasta format)

The alignment was generated with Muscle [[96](#_ENREF_96)] and ambiguous parts of the alignment were masked with Gblocks [[97](#_ENREF_97)] (settings for a less stringent selection).

>AMC476051001

AGCCTTTAGGCTTTATTTTAGGTTGTTTCTGCCCAGTGCTTGTAAAAGGCCGCAGTACCTTGACTGTGCTAAGGTAGCGAAATAAATAGGCTTTTAATTGGAGTCCAGTATGAACGGAAAAGTGGGAATTAACTTTCTCTTAAAATTTTAATTTTTTTAGTAAGTGAAAATACTTACACAAGATAAAAGACGATAAGACCCTGGGAACTTTCGCTTTTGTTGGGGCAACAATGAACTTCCTATCTTGCCAAGTTTCTATTAGGAAAAGTTACCCCAGGGATAACAGCATAATT--TTGAAAAGCTTGTGACCTCGATGTTGGACTAGGAAAATTATGATAAGCCGTCACAAATTAATGTTCTGTTCGAACACTCTCCTACAT

>ZSM20100592

AGCTTTTAGACTTTATTTTAGGTTGTTTCTGCCCAGTGCTTGTAAAAGGCCGCAGTACCTTGACTGTGCTAAGGTAGCGAAATAAGTAGGCTTTTAATTGGAGTCCGGTATGAATGGAAGAGCGGAAATTAACTGTCTCTTTAAAATATAATTTTTTTAATAGGTGAAAATACCTACATAAGATAAAAGACGATAAGACCCTGGGAACTTGTGCTTTTGTTGGGGCAACACTGAACTTCCAATTGTATCAAGTTACTATTAGGGAAAGTTACCCCAGGGATAACAGCATAATT--TTTAAAAGCTTGTGACCTCGATGTTGGACTAGGAAAATTATGATAAGCCATCATAAGTTTATGTTCTGTTCGAACATTCTCCTACAT

>ZSM20081014

AGCCTTTAGACTTTATTTTAGGTTGTTTCTGCCCAGTGCTTGTAAAAGGCCGCAGTACCTTGACTGTGCTAAGGTAGCGAAATAAGTAGGCTTTTAATTGGAGTCCAGTATGAATGGAAGGATGGGAATTAACTGTCTCTTAAAATTTTAACTTTTTTAATAAGTGAAAATACTTATGTTAGATAAAAGACGATAAGACCCTGGGAACTTATGCTTTTGTTGGGGCAACAATGAACTTCCCTACTTACCAAGTTTCTAATAGGGAAAGTTACCCCAGGGATAACAGCATAATT--TATAAAAGCTTGTGACCTCGATGTTGGACTAGGAAAGTTATGATAAGCCATCATAAGTTTATGTTCTGTTCGAACTTTCTCCTACAT

>ZSM20100379

AGCCTTTAGACTTTATTTTAGGTTATTTCTGCCCAGTGCTTGTAAAAGGCCGCAGTACCTTGACTGTGCTAAGGTAGCGAAATAAGTAGGCTTTTAATTGGAGTCCAGTATGAATGGAAGGATGGGAATTAACTGTCTCTTAAAATTTTAACTTTTTTAATAAGTGAAAATACTTATGTTAGATAAAAGACGATAAGACCCTGGGAACTTATGCTTTTGTTGGGGCAACAATGAACTTCCCTATTTACCAAGTTTCTAATAGGGAAAGTTACCCCAGGGATAACAGCATAATT--TATAAAAGCTTGTGACCTCGATGTTGGACTAGGAAAATTATGATAAGCCATCATAAGTTTATGTTCTGTTCGAACTTTCTCCTACAT

>SICBC2010KJ01C08

AGCCTTAAGTCAATATTTTAGGTGATCCCTGCCCACTGCTAGTCAAGGGCCGCAGTACCTTGACTGTGCTAAGGTAGCGAAATCAATAGACTTTTAAATGAAGCCCAGAATGAAGGGGACCTCGGAAATTAACTGTTTCAAGGTGGATTAAATTTATTAGGAGGTGAAAATTCCTCTACACTATAAAAGACGAGAAGACCCCGGGATCTTTGATTTTTGTTGGGGCAACAGTA-AACCTCTATCAAGCCAGT-AAAGGCACGAGAAAGATACCCCGGGGATAACAGCATAATTTTTT----AGTTTATGACCTCGATGTTGGACTAGGGATTTTATGGTTAGCCGCCAAAGAAGGTAGTTCTGTTCGAACAACCCCCTACGT

>SICBC2010KJ01D05

AGCCTTAAGTCAATATTTTAGGTGATCCCTGCCCACTGCTAGTCAAGGGCCGCAGTACCTTGACTGTGCTAAGGTAGCGAAATCAATAGACTTTTAAATGAAGCCCAGAATGAAGGGGACCTCGGAAATTAGCTGTTTCAAGGTGGATTAAATTTATTAGGAGGTGAAAATTCCTCCATGTTATAAAAGACGAGAAGACCCCGGGATCTTTGATTTTTGTTGGGGCAACAGTA-AACCTCTATCAAGCCAGT-TAAGGCACGAGAAAGATACCCCGGGGATAACAGCATAATTTTTT----AGTTTATGACCTCGATGTTGGACTAGGGATTTTATGGTTAGCCGCCAAAGAAAGTAGTTCTGTTCGAACAATCCCCTACGT

>ZSM20090197

AGCCTTAAGTCAATATTTTAGGTGATCCCTGCCCACTGCTAGTCAAGGGCCGCAGTACCTTGACTGTGCTAAGGTAGCGAAATCAATAGACTTTTAAATGAAGCCCAGAATGAAGGGGACCTCGGAAATTAACTGTTTCAAGGTGGATTAAATTTATTAGGAGGTGAAAATTCCTCCATATTATAAAAGACGAGAAGACCCCGGGATCTTTGATTTTTGTTGGGGCAACAGTA-AACCTCTATCAAGCCAGT-TAAGGCACGAGAAAGATACCCCGGGGATAACAGCATAATTTTTT----AGTTTATGACCTCGATGTTGGACTAGGGATTTTATGGTTAGCCGCCAAAGAAAGTAGTTCTGTTCGAACAATCCCCTNNNN

>ZSM20081013

AGCTTGAAGTTTTTATTTTAAGTGATTTCTGCCCACTGCTAGTAAAAGGCCGCAGTACTTTGACTGTGCTAAGGTAGCGAAATCAATAGACTTTTAATTGGAGCCCAGTATGAAAGAAATAACGGAAAGTAACTGTTTCTTTTTATATAAACTTTATTAAAAGGTGAAAATGCCTTTAATAGAAGACAGACGAGAAGACCCTGGGATCTTTTATTTTTGTTGGGGCAACATTAAAGTTAT----ATGCCAGA-AAAGGAATGAATAAGATACCCCAGGGATAACAGCATAATTGATT----GGCTTGTGACCTCGATGTTGGACTAGGGATTTTATGGCTAGCCGCCAAAAATGAGTGTTCTGTTCGAACGTTCCCCTACAT

>AMC476062001

AGCTTAATGTGGTTATTTTAAGTGATTCCTGCCCATTGCTGGTAAAAGGCCGCAGTACCTTGACTGTGCTAAGGTAGCGAAATCAATAGACTTTTAAATGAAGCCCAGAATGAATGGAAATACGGAGGTTAGCTGTTTCTTTTTTAGGAAACTTTGTTAGAGGGTGAAAATACCCCTATTAGATGAAAGACGAGAAGACCCTGGGATCTTTTATTTTTGTTGGGGCAACACTA-ATTCTTCTTCTAGTCAGC-GAAGGTTTGTATAAGATACCCCAGGGATAACAGCATAATTTTTT----AGCTTATGACCTCGATGTTGGACTAGGGGTTTTAGGGCTAGAAGCCCAAAAAAGGTGTTCTGTTCGAACCTTCTCCTACAT

>ZSM20071135

AGCCTAATGAAATTATTTTAGGTGATTTCTGCCCGTTGCGAGTGAAAGGCCGCAGTACCTTGACTGTGCTAAGGTAGCGAAATCAGTTGACTTTTAAATGGAGCCCAGAATGAAAGGAATAACGGGGGATTGCTGTTTCTTTTTATAATAACTTTATTATAAGGTGAAAATTCCTTTAATAGATGAAAGACGAGAAGACCCTGGGATTTTTAATTTTTGTTGGGGCAACAACA--CTCTTCTCCTTGCTAATTAAAGGAACGAAAAAAATACCCCAGGGATAACAGCATAATTTATT----AGCTTATGACCTCGATGTTGGATTAGGACTTTTTTGGTTAGAAGCCTAAAAAAGCTGTTCTGTTCGAACTTTATCCTATTT

>ZSM20080176

AGCCTAATGAAACCATYTTAGGTGATTTCTGCCCGTTGCGAGTGAAAGGCCGCAGTACCTTGACTGTGCTAAGGTAGCGAAATCAGTTGACTTTTAAATGGAGCCCAGAATGAAAGGAACAACGGGGGATTACTGTTTCTTTTTATAATAACTTTATTAAAAGGTGAAAATTCCTTTAATAGATGAAAGACGAGAAGACCCTGGGATTTTTAGTTTTTGTTGGGGCAACAACA--CTCGTTTCCTTGCTAGTTAAAGGAACGAAAAAAATACCCCAGGGATAACAGCATAATTTATT----AGCTTATGACCTCGATGTTGGACTAGGACTTTTTTGGTTAGAAGCCTAAAAAAGTTGTTCTGTTCGAACCTTATCCTACAT

>ZSM20071820

AGCCTAATGAAACTATYTTAGGTGATTTCTGCCCGTTGCAAGTGAAAGGCCGCAGTACCTTGACTGTGCTAAGGTAGCAAAATCAGTTGACTTTTAAATGGAGCCCAGAATGAAAGGAACAACGGGGGATTGCTGTTTCTTTTTATAATAACTTTATTAAAAGGTGAAAATTCCTTTAATAGATGAAAGACGAGAAGACCCTGGGATTTTTTATTTTTGTTGGGGCAACAACA--CTCGCCTCCTTGCTAATTAAAGGAACGGGAAAAATACCCCAGGGATAACAGCATAATTTATT----AGCTTATGACCTCGATGTTGGACTAGGACTTTTTTGGTTAGAAGCCTAAAAAAGTTGTTCTGTTCGAACCTTATCCTACAC

>ZSM20100391

AGCCTAATGAAATCATCTTAGGTGACTTCTGCCCGTTGCAAGTGAAAGGCCGCAGTACCTTGACTGTGCTAAGGTAGCGAAATCAGTTGACTTTTAAATGGAGCCCAGAATGAAAGGAACAACGGGGGATTGCTGTTTCTTTTTATAATAACTTTATTAAAAGGTGAAAATTCCTTTAATAGATGAAAGACGAGAAGACCCTGGGATTTTTAGTTTTTGTTGGGGCAACAACA--CTCGTCTCCTTGCTAAT--AAGGAACGAGAAAAATACCCCAGGGATAACAGCATAATTTATT----AGCTTATGACCTCGATGTTGGACTAGGACTTTTTTGGTTAGAAGCCTAAAAAAGTTGTTCTGTTCGAACCTTATCCTACAT

>ZSM20090471

AGCCTGTTGCAAAAATAGTAGGTTATTCCTGCCCATTGCAGGTAAAAGGCCGCAGTACTTTGACTGTGCTAAGGTAGCGAAATCAGTAGACTTTTAAATGGAGCCCAGAATGAATGGAAAAACGGAAATTTACTGTTTCTTTTTATAATAACTTTATTAAAAGGTGAAAATGCCTCTAATAGATGAAAGACGAGAAGACCCTGGGACCTTCAGCTTTTGTTGGGGCAACAGCA-AGTCCTAGTCTCGTCAAT-TCAAGTTAGAATAAGGTACCCCAGGGATAACAGCATAATTTTTT----AGTTTATGACCTCGATGTTGGACTAGGATTTCTACGGTTAGACGCCTCAAGAACACGTTCTGTTCGAACCTTCTCCTACAT

>ZSM20090472

AGCCTGTTGCAAAAATAGTAGGTTATTCCTGCCCATTGCAGGTAAAAGGCCGCAGTACTTTGACTGTGCTAAGGTAGCGAAATCAGTAGACTTTTAAATGGAGCCCAGAATGAATGGAAAAACGGAAATTTACTGTTTCTTTTTATAATAACTTTATTAAAAGGTGAAAATGCCTCTCATAGATGAAAGACGAGAAGACCCTGGGACCTTCAGCTTTTGTTGGGGCAACAGCA-AGTCCTAGTCTCGTCAAT-TCAAGTTAGAATAAGGTACCCCAGGGATAACAGCATAATTTTTT----AGTTTATGACCTCGATGTTGGACTAGGATTTCTACGGTTAGACGCCTCAAGAACACGTTCTGTTCGAACCTTCTCCTNNNN

>ZSM20100595

AGCCTAGTGAAGTTATAGTAGGTAGTTCCTGCCCGTTGCAAGTGAAAGGCCGCAGTACCTTGACTGTGCTAAGGTAGCGAAATCAGTAGACTTTTAAATGGAGCCCAGAATGAAAGGAAGCACGGAAGCTTACTGTTTCTTTTTATATAAACTTTTTTAAAAGGTGAAAACACCTTTAACAGATGAAAGACGAGAAGACCCTGGGACCTTTGATTTTTGTTGGGGCAACATTA-ATTCCTTATCTTGTCAAA-TCAAGGATGGATAAGGTACCCCAGGGATAACAGCATAATTTTTT----AGTTTATGACCTCGATGTTGGACTAGGAATTCTATGGCTAGACGCCTAAAAATATTGTTCTGTTCGAACTTTTTCCTACAT

>ZSM20100596

AGCCTAGTGAAGTTATAGTAGGTAGTTCCTGCCCGTTGCAAGTGAAAGGCCGCAGTACCTTGACTGTGCTAAGGTAGCGAAATCAGTAGACTTTTAAATGGAGCCCAGAATGAAAGGAGGCACGGAAGCTTACTGTTTCTTTTTATATAAACTTTTTTAAAAGGTGAAAACACCTTTTACAGATGAAAGACGAGAAGACCCTGGGACCTTTGATTTTTGTTGGGGCAACATTA-ATTCCTTATCTTGTCAAA-TCAAGAATGGATAAGGTACCCCAGGGATAACAGCATAATTTTTT----AGTTTATGACCTCGATGTTGGACTAGGAATTCTATGGCTAGACGCCTAAAAATATTGTTCTGTTCGAACTTTTTCCTACAT

>ZSM20100597

AGCCTAGTGAAGTTATAGTAGGTAGTTCCTGCCCGTTGCAAGTGAAAGGCCGCAGTACCTTGACTGTGCTAAGGTAGCGAAATCAGTAGACTTTTAAATGGAGCCCAGAATGAAAGGAAGCACGGAAGCTTACTGTTTCTTTTTATATAAACTTTTTTAAAAGGTGAAAACACCTTTAACAGATGAAAGACGAGAAGACCCTGGGACCTTTGATTTTTGTTGGGGCAACATTA-ATTCCTTATCTTGTCAAA-TCAAGAATGGATAAGGTACCCCAGGGATAACAGCATAATTTTTT----AGTTTATGACCTCGATGTTGGACTAGGAATTCTATGGCTAGACGCCTAAAAATATTGTTCTGTTCGAACTTTTTCCTACAT

>ZSM20100603

AGCCTAGTGAAGTTATAGTAGGTAGTTCCTGCCCGTTGCAAGTGAAAGGCCGCAGTACCTTGACTGTGCTAAGGTAGCGAAATCAGTAGACTTTTAAATGGAGCCCAGAATGAAAGGAAGCACGGAAGCTTACTGTTTCTTTTTATATAAACTTTTTTAAAAGGTGAAAACACCTTTAACAGATGAAAGACGAGAAGACCCTGGGACCTTTGATTTTTGTTGGGGCAACATTA-ATTCCTTATCTTGTCAAA-TCAAGAATGGATAAGGTACCCCAGGGATAACAGCATAATTTTTT----AGTTTATGACCTCGATGTTGGACTAGGAATTCTATGGCTAGACGCCTAAAAATATTGTTCTGTTCGAACTTTTTCCTACAT

>SICBC2010KJ01B09

TGCCTGTTGG-GTCATAATAGGTTGTTTCTGCCCACTGCTAGTGAAGGGCCGCAGTACTTTGACTGTGCTAAGGTAGCGTAATCATTTGGCTTTTAATTGGGGTCCTGTATGAA-GAAAGAACGGGGGGGGGCTGTCTCTCCCTGCTTAAAGTTACTAAGAAGGTGAAAATCCCTTCAAAAAACAAAAGACGAGAAGACCCCGGGAGCTATTA-TTTTGTTGGGGCAACAAAGAACCTCCTTTCTAGCCAATTTGTTTTGTGGGTAAGCTACCCCGGGGATAACAGCGTAATTTTTGAAAAAGCTTGCGACCTCGATGTTGGACTAGGCACTTTTAGGTTAGCCGCCTAAGAAGATAGCTCTGTTCGAGCGGTAGCCTACGT

>SICBC2010KJ01E03

TGCCTGTTGG-GTCATAATAGGTTGTTTCTGCCCACTGCTAGTGAAGGGCCGCAGTACTTTGACTGTGCTAAGGTAGCGTAATCATTTGGCTTTTAATTGGGGTCCTGTATGAA-GAAAGAACGGGGGGGGGCTGTCTCTCCCTGCTTAAAGTTACTTAGAAGGTGAAAATCCCTTCAAAAAACAAAAGACGAGAAGACCCCGGGAGCTATCA-TTTTGTTGGGGCAACAAAGAACCTCCTTTCTAGCCAATTTGTTTTGTGGATAAGCTACCCCGGGGATAACAGCGTAATT-TTTGAAAAGCTTGCGACCTCGATGTTGGACTAGGCACTTTTAGGTTAGCCGCCTAAGAAGATGGCTCTGTTCGAGCGACAGCCTACGT

>ZSM20110723

TGCCTGTTGG-GTCATAATAGGTTGTTTCTGCCCACTGCTAGTGAAGGGCCGCAGTACTTTGACTGTGCTAAGGTAGCGTAATCATTTGGCTTTTAATTGGGGTCCTGTATGAA-GAAAGAACGGGGGGGGGCTGTCTCTCCCTGCTTAAAGTTACTAAGAAGGTGAAAATCCCTTCAAAAAACAAAAGACGAGAAGACCCCGGGAGCTATCA-TTTTGTTGGGGCAACAAAGAACCTCCTTTCTAGCCAATTTGTTTTGTGGGTAAGCTACCCCGGGGATAACAGCGTAATT-TTTGAAAAGCTTGCGACCTCGATGTTGGACTAGGCACTTTTAGGTTAGCCGCCTAAGAAGATAGCTCTGTTCGAGCGACAGCCTACAT

>SICBC2010KJ01C09

TGCCTGTTGG-GTCATAATAGGTTGTTTCTGCCCACTGCTAGTGAAGGGCCGCAGTACTTTGACTGTGCTAAGGTAGCGTAATCATTTGGCTTTTAATTGGGGTCCTGTATGAA-GAAAGAACGGGGGAGGGCTGTCTCTCCCTGCTTAAAGTTACTAAGAAGGTGAAAATCCCTTCAAAAAACAAAAGACGAGAAGACCCCGGGAGCTATCA-TTTTGTTGGGGCAACAAAGAACCTCCTTTCTAGCCAATTTGTTTTGTGGGTAAGCTACCCCGGGGATAACAGCGTAATT-TTTGAAAAGCTTGCGACCTCGATGTTGGACTAGGCACTTTTAGGTTAGCCGCCTAAGAAGATAGCTCTGTTCGAGCGACAGCCTACGT

>SICBC2010KJ01B07

TGCCTGTTGG-GTCATAATAGGTTGTTTCTGCCCACTGCTAGTGAAGGGCCGCAGTACTTTGACTGTGCTAAGGTAGCGTAATCATTTGGCTTTTAATTGGGGTCCTGTATGAA-GAAAGAACGGGGGGGGGCTGTCTCTCCCTGCTTAAAGTTACTAAGAAGGTGAAAATCCCTTCAAAAAACAAAAGACGAGAAGACCCCGGGAGCTATCA-TTTTGTTGGGGCAACAAAGAACCTCCTTTCTAGCCAATTTGTTTTGTGGGTAAGCTACCCCGGGGATAACAGCGTAATT-TTTGAAAAGCTTGCGACCTCGATGTTGGACTAGGCACTTTTAGGTTAGCCGCCTAAGAAGATAGCTCTGTTCGAGCGACAGCCTACGT

>ZSM20110722

TGCCTGTTGG-GCCATAATAGGTTGTTTCTGCCCACTGCTAGTGAAGGGCCGCAGTACTTTGACTGTGCTAAGGTAGCGTAATCATTTGGCTTTTAATTGGGGTCCTGTATGAA-GAAAGCACGGGGGGGGGCTGTCTCTCCCTGTTTAAAGTTGCTTAGAAGGTGAAAATCCCTTCAATAAATAAAAGACGAGAAGACCCCGGGAGCTATTA-TTTTGTTGGGGCAACAAAGAACCTCCTTTCTTGCCAATTTGTTTTGTGGGTAAGCTACCCCGGGGATAACAGCGTAATT-TTTGAAAAGCTTGCGACCTCGATGTTGGACTAGGCACTTTAAGGTTAGCCGCCCAAGAAGTCAGCTCTGTTCGAGCGACAGCCTACAT

>SICBC2010KJ01A10

TGCCTGTTGG-GTCATAATAGGTTGTTTCTGCCCACTGCTAGTGAAGGGCCGCAGTACTTTGACTGTGCTAAGGTAGCGTAATCATTTGGCTTTTAATTGGGGTCCTGTATGAA-GAAAGCACGGGGGGGGGCTGTCTCTCCCTGTTTAAAGTTGCTTAGAAGGTGAAAATCCCTTCAATAAATAAAAGACGAGAAGACCCCGGGAGCTATTA-TTTTGTTGGGGCAACAAAGAACCTCCTTTCTTGCCAATTTGTTTTGTGGGTAAGCTACCCCGGGGATAACAGCGTAATT-TTTGAAAAGCTTGCGACCTCGATGTTGGACTAGGCACTTTAAGGTTAGCCGCCCAAGAAGACAGCTCTGTTCGAGCGACAGCCTACGT

>ZSM20090198

TGCCTGTTGG-GTCATAATAGGTTGTTTCTGCCCACTGCTAGTGAAGGGCCGCAGTACTTTGACTGTGCTAAGGTAGCGTAATCATTTGGCTTTTAATTGGGGTCCTGTATGAA-GAAAGCACGGGGGGGGGCTGTCTCTCCCTGTTTAAAGTTGCTTAGAAGGTGAAAATCCCTTCAATAAATAAAAGACGAGAAGACCCCGGGAGCTATTA-TTTTGTTGGGGCAACAAAGAACCTCCTTTCTTGCCAATTTGTTTTGTGGGTAAGCTACCCCGGGGATAACAGCGTAATT-TTTGAAAAGCTTGCGACCTCGATGTTGGACTAGGCACTTTAAGGTTAGCCGCCCAAGGAGACAGCTCTGTTCAAGCGACAACCTACGT

>AMC476054001

AGCCTGG-GGCTTTATCTTAGGTAGCCTCTGCTCACTGCTAGTAAAGGGCCGCAGTACTTTGACTGTGCTAAGGTAGCGAAATCAATAGGCTTTTAATTGGAGCCCGGTATGAATGAGGGCACGGGGACCAGCTGTCTCACAC-ACATAAGCTTTCTTGTAGGGTGAGAAAGCCCTTTCAAGATAAAAGACGAGAAGACCCTGGGAGTTTTTGCTTTAGTTGGGGCAACTAGGACCTTCCCCTCTAGCCAGATTCATTTGAGAACAAACTACCCCAGGGATAACAGCATAATTTTTTTA--AGCTTGTGACCTCGATGTTGGACTAGGCACCTGATGATTAGCCGTCAAAAGGTTTAGTTCTGTTCGAACATGCGCCTACAT

>ZSM20071381 (1,414)

AGCCTGAGGACGAAATCTTAGGTAAATTCTGCCCACTGCAAGTAAAAGGCCGCAGTACTTTGACTGTGCTAAGGTAGCGAAATCATTAGGCTTTTAAATGAAGCCCGGAATGAAGGAAACCACGGAAAGGAGCTGTCTCTTTTTCCTGAAAGTTAGTTGTAGGGTGAAAACACCCTCTTTAGATAAAAGACGAGAAGACCCCAGGAGCTTTAATTTTTGTTGGGGCAACACAGAACTTCCAGGCTAGTCAAATGGTTTTCAGAGCAAGCTACCCTGGGGATAACAGCATTATT-TTTTAAAAGCTTGTGACCTCGATGTTGGACTAGGGACTATTAGACTAGAAGTCTAATTAGCCGGTTCTGTTCGAACCTCACCCTACGT

>ZSM20080953

AGCCTGAGGGCGAAATCTTAGGTAAATTCTGCCCACTGCAAGTAAAAGGCCGCAGTACTTTGACTGTGCTAAGGTAGCGAAATCATTAGGCTTTTAAATGAAGCCCGGAATGAAGGAAACCACGGAAAGGAGCTGTCTCTTTTTCCTGAAAGTTAGTTGTGGGGTGAAAACACCCCCTTTAGATAAAAGACGAGAAGACCCCAGGAGCTTTAATTTTTGTTGGGGCAACACAGAACTTCCAGGCTAGTCAAGTGGTTTTCAGAGCAAGCTACCCTGGGGATAACAGCATTATTTTTTAAAAAGCTTGTGACCTCGATGTTGGACTAGGGACTATTAGACTAGAAGTCTAACTAGCCGGTTCTGTTCGAACCTACCCCTACAC

>ZSM20080925

AGCCTGAGGGCGAAATCTTAGGTAAATTCTGCCCACTGCAAGTAAAAGGCCGCAGTACTTTGACTGTGCTAAGGTAGCGAAATCATTAGGCTTTTAAATGAAGCCCGGAATGAAGGAAACCACGGAAAGGAGCTGTCTCTTTTTCCTGAAAGTTAGTTGTGGGGTGAAAACACCCCCTTTAGATAAAAGACGAGAAGACCCCAGGAGCTTTAATTTTTGTTGGGGCAACACAGAACTTCCAGGCTAGTCAAGTTGTTTTTAGAGCAAGCTACCCTGGGGATAACAGCATTATTTTTTAAAAAGCTTGTGACCTCGATGTTGGACTAGGGACTATTAGACTAGAAGTCTAATTAGCCGGTTCTGTTCGAACCTACCCCTACAC

>ZSM20080054

AGCCTGAGGGCGAAATCTTAGGTAAATTCTGCCCACTGCAAGTAAAAGGCCGCAGTACTTTGACTGTGCTAAGGTAGCGAAATCATTAGGCTTTTAAATGAAGCCCGGAATGAAGGAAACCACGGAAAGGAGCTGTCTCTTTTTCCTGAAAGTTAGTTGTGGGGTGAAAACACCCCCTTTAGATAAAAGACGAGAAGACCCCAGGAGCTTTAATTTTTGTTGGGGCAACACAGAACTTCCAGGCTAGTCAAGTGGTTTTCARAGCAAGCTACCCTGGGGATAACAGCATTATT-TTTTAAAAGCTTGTGACCTCGATGTTGGACTAGGGACTATTAGACTAGAAGTCTAATTAGCCGGTTCTGTTCGAACCTACCCCTNNNN

>ZSM20080055

AGCCTGAGGGAGAAATCTTAGGTAAATTCTGCCCACTGCAAGTAAAAGGCCGCAGTACTTTGACTGTGCTAAGGTAGCGAAATCATTAGGCTTTTAAATGAAGCCCGGAATGAAGGAAACCACGGAAAGGAGCTGTCTCTTTTTCCTGAAAGTTAGTTGTGGGGTGAAAACACCCCCTTTAGATAAAAGACGAGAAGACCCCAGGAGCTTTAATTTTTGTTGGGGCAACACAGAACTTCCAGGCTAGTCAAGTGGTTTTCAGAGCAAGCTACCCTGGGGATAACAGCATTATTTTTTAAAAAGCTTGTGACCTCGATGTTGGACTAGGGACTATTAGACTAGAAGTCTAATTAGCCGGTTCTGTTCGAACCTACCCCTACGT
