# Supplementary material for: How to describe a cryptic species? Practical challenges of molecular taxonomy
Source: Front Zool. 2013 Sep 27;10:59. doi: 10.1186/1742-9994-10-59 (PMC4015967; doi:10.1186/1742-9994-10-59)
Supplement: Additional file 6 — COI alignment of Pontohedyle (fasta format). The alignment was generated with MUSCLE [107]. [file 1742-9994-10-59-S6.docx]

### Additional file 6 – COI alignment of *Pontohedyle* (fasta format)

The alignment was generated with Muscle [[96](#_ENREF_96)].

>ZSM20110722

TACGCTATACATTATCTTTGGGGTTTGATGCGGCTTGGTCGGTGCGGGGTTGTCTTTGTTGATTCGGGTGGAGTTAGGGACTTCTGGGGTGTTAACCGACCCACACTTCTACAATGTGGTAGTGACTGCCCATGCTTTCGTCATGATTTTTTTTATGGTTATACCCGTCTTGATCGGGGGTTTCGGTAATTGAATGATCCCGCTCCTGATTGGGGCACCTGATATGGCCTTTCCCCGGTTAAATAATTTAAGGTTTTGGCTGCTTCCTCCTGCGTTTATTTTACTTATAAGCTCGGTTTTAGTAGAAGGCGGTGCGGGGACAGGCTGAACTTTATACCCCCCCTTGAGCG---CGGAGGGTCACTCAGGGTTTTCAGTTGATTTAGCTATTTTTTCTCTGCACTTGGCGGGGGTCTCATCTATTCTAGGGGCGGTTAACTTCATCACAACTATCTGGAATATGCGGGCCCCAGGGGTTACTTGAGAGCGGTTGAATCTTTTTGTGTGGTCTTTACTCATTACAGCGTTATTGTTGTTGTTGTCACTACCAGTACTGGCTGGTGCTCTTACAATGTTGTTAACTGATCGGAATTTTAATACTACTTTTTTTGATCCAGCGGGGGGTGGGGACCCAGTTTTATATCAACATCTGTTC

>SICBC2010KJ01B09

CACGTTGTACATTATCTTTGGGGTTTGATGCGGTTTGGTCGGTGCGGGGTTGTCTTTGTTGATTCGGGTAGAGTTGGGGACTTCTAGGGTGTTAACCGACCCTCACTTCTACAATGTGGTTGTGACTGCCCATGCTTTCGTTATGATTTTTTTTATGGTTATACCTGTCTTGATCGGGGGTTTTGGTAACTGAATGATCCCGCTTCTGATTGGGGCACCTGATATGGCTTTTCCTCGGTTAAATAACCTAAGGTTTTGGCTGCTCCCGCCTGCGTTTATTTTACTTATAAGCTCGGTTTTGGTAGAAGGCGGAGCGGGGACAGGTTGAACCTTGTATCCCCCTCTGAGCG---CGGAGGGGCATTCAGGATTTTCAGTTGATTTAGCCATTTTTTCTCTGCACTTGGCAGGGGTCTCTTCTATCTTAGGGGCAGTTAATTTTATTACCACTATTTGGAATATGCGGGCCCCGGGGGTTACTTGAGAGCGGCTGAATCTTTTTGTCTGGTCTTTACTTATTACAGCGTTATTGTTGCTGTTGTCACTGCCTGTGCTAGCTGGTGCTCTTACAATGTTGTTAACTGATCGGAATTTTAATACTACTTTTTTTGATCCGGCGGGGGGCGGGGACCCTGTTTTATACCAACACCTGTTC

>ZSM20110723

CACGTTGTACATTATCTTTGGGGTTTGATGCGGTTTGGTCGGTGCGGGGTTGTCTTTGTTGATTCGGGTAGAGTTGGGGGCTTCTAGGGTGTTAACCGACCCTCACTTCTACAATGTGGTTGTGACTGCCCATGCTTTCGTTATGATTTTTTTTATGGTTATACCTGTCTTGATCGGGGGGTTTGGTAACTGAATGATCCCGCTTCTGATTGGGGCACCTGATATGGCTTTTCCTCGGTTAAATAACCTAAGGTTTTGGCTGCTCCCTCCCGCGTTTATTTTACTTATAAGCTCGGTTCTGGTAGAAGGCGGAGCGGGGACAGGTTGAACCTTGTATCCCCCTCTGAGCG---CGGAGGGTCATTCAGGGTTTTCAGTTGATTTAGCCATTTTTTCTCTGCACTTGGCAGGGGTCTCTTCTATTTTAGGGGCAGTTAACTTTATTACCACTATTTGGAATATGCGGGCCCCGGGGGTTACTTGAGAGCGGCTGAATCTTTTTGTCTGGTCTTTACTTATTACAGCGTTATTGTTGCTGTTGTCACTGCCTGTGCTAGCTGGTGCTCTTACAATGTTGTTAACTGATCGGAATTTTAATACTACTTTTTTTGATCCGGCGGGGGGCGGGGACCCTGTTTTATACCAACACTTGTTC

>SICBC2010KJ01C09

CACGTTGTACATTATCTTTGGGGTTTGATGTGGTTTGGTCGGTGCGGGGTTGTCTTTGTTGATTCGGGTAGAGTTGGGGACTTCTAGAGTGTTAACCGACCCTCACTTCTACAATGTGGTTGTGACTGCCCATGCTTTCGTTATGATTTTTTTTATGGTTATACCTGTCTTGATTGGGGGTTTTGGTAACTGAATGATCCCGCTTCTGATTGGGGCACCTGATATGGCTTTTCCTCGGTTAAATAATCTAAGGTTTTGGCTGCTCCCTCCCGCGTTTATTTTACTTATAAGCTCGGTTCTGGTAGAAGGCGGAGCGGGGACAGGTTGAACCTTGTATCCCCCTCTGAGCG---CGGAGGGTCATTCAGGGTTTTCAGTTGATTTAGCCATTTTTTCTCTGCACTTGGCAGGGGTTTCTTCTATTTTAGGGGCAGTTAACTTTATTACCACTATTTGGAATATGCGGGCCCCGGGGGTTACTTGAGAGCGGCTGAATCTTTTTGTCTGGTCTTTACTTATTACAGCGTTATTGCTGCTGTTGTCACTGCCTGTGCTAGCTGGTGCTCTTACAATGTTGTTAACTGATCGGAATTTTAATACTACTTTTTTTGATCCGGCGGGGGGCGGGGACCCTGTTTTATACCAACACCTGTTC

>ZSM20071133

AACCTTATACATGTTGTTTGGAATTTGGTGCGGATTAGTTGGGACAGCTTTGTCACTGCTGATTCGGATTGAGCTCGGCGTGACTTCTGTGTTTTCAGATAGTCACTTTTACAATGTTATTGTTACTGCGCATGCTTTCACTATAATTTTTTTTATGGTTATGCCAATTATAATTGGCGGGTTCGGCAATTGAATGGTCCCCTTGCTTCTCGGTGCCCCTGATATGAGATTCCCTCGAATAAACAATCTAAGATTTTGATTACTACCTCCATCTTTTCTACTTCTCCTGTGTAGAAGTATAGTAGAAGGAGGCGCAGGGACTGGGTGAACAGTTTACCCCCCTCTTAGTGGTCCAACAGGACACAGAAGTTCATCAGTCGACTTAGTAATTTTCTCCCTTCATTTAGCTGGGGTTTCTTCTATTTTAGGGGCCATTAATTTTATTACGACTATTTACAATATACGAATTCCCGGAGTTACAATAGATCGGCTGAACTTATTTTGTTGGTCGATTTTGGTGACAGCGTTTCTTCTCTTACTGAGTCTTCCAGTTCTCGCCGGAGCTATTACTATACTTTTGACTGACCGGAACTTCAATACTAGATTTTTTGATCCAGCTGGGGGAGGCGACCCCATTTTATACCAGCATTTATTT

>ZSM20080953

AACTCTATATCTTGTTTTTGGAGTCTGGTGCGGTTTAGTGGGTACAGGGCTTTCTCTTCTTATCCGGTTTGAGCTAGGCACGTCCTCCGTCTTCATAGACGAGCACTTTTATAATGTTATTGTAACGGCCCATGCATTTGTTATAATTTTTTTTATGGTCATGCCACTAATAATTGGCGGTTTTGGTAATTGAATGGTTCCCCTGTTAATTGGGGCCCCCGACATAAGGTTTCCTCGAATAAATAACATAAGGTTTTGGTTGCTTCCCCCTAGGTTTGTTCTTTTGTTAAGCTCAAGATTAGTTGAGGGGGGCGCGGGTACAGGGTGAACTGTCTATCCGCCCCTAAGCGGGTCTGTTGCGCACGGAGGAGCCTCGGTAGACTTAGTTATTTTTTCACTACATTTGGCGGGAATGTCTTCTATTCTTGGGGCTATCAACTTCATTACAACAATCTTTAATATACGGTCCCCCGGGCTAACAATGGAACGGCTGAGGTTGTTTGTTTGGTCTGTTCTAGTCACGGCTTTTTTACTTCTGCTAAGATTGCCCGTGCTAGCAGGGGCTATCACAATGCTTTTAACTGATCGTAACTTTAACACTAGTTTTTTTGACCCGGCCGGAGGGGGGGACCCTATTTTATACCAACACCTTTTC

>ZSM20071381

NNNTCTATATCTTGTTTTTGGAGTCTGGTGCGGTTTAGTGGGTACAGGGCTTTCCCTTCTTATCCGGTTTGAGCTAGGCACGTCCTCCGTCTTCATAGACGAGCACTTTTATAATGTTATTGTGACGGCCCATGCATTTGTTATAATTTTTTTTATGGTCATGCCACTAATAATTGGTGGTTTTGGGAATTGAATGGTTCCCCTGTTAATTGGGGCCCCCGACATAAGGTTTCCTCGAATAAATAACATAAGATTTTGGTTGCTTCCCCCTAGGTTTGTTCTTTTGTTGAGCTCAAGATTAGTTGAGGGGGGTGCGGGTACAGGGTGAACTGTCTATCCGCCCCTAAGCGGGTCTGTTGCGCACGGAGGAGCCTCGGTAGACTTAGTTATTTTTTCACTACATTTGGCGGGAATGTCTTCTATTCTTGGGGCTATCAACTTCATTACAACAATCTTTAATATGCGGTCCCCCGGGCTAACAATGGAACGGCTGAGGTTGTTTGTTTGGTCTGTTCTAGTCACGGCTTTTTTACTTCTGCTAAGACTGCCTGTGCTAGCAGGGGCTATCACAATGCTTTTAACTGATCGTAACTTTAACACTAGTTTTTTTGACCCGGCCGGAGGGGGGGACCCTATTTTATACCAACACCTTTTC

>ZSM20080925

NNNTCTATATCTTGTTTTTGGAGTCTGGTGCGGTTTAGTAGGTACAGGGCTTTCCCTTCTTATCCGGTTTGAGCTAGGCACGTCCTCCGTGTTCATAGACGAGCACTTTTATAATGTTATTGTGACGGCCCATGCATTTGTTATAATTTTTTTTATGGTCATGCCACTAATAATTGGTGGTTTTGGGAATTGAATGGTTCCCCTGTTAATTGGGGCCCCCGACATAAGGTTTCCTCGGATAAACAACATAAGGTTTTGGTTGCTTCCCCCTAGGTTTGTTCTTTTGTTGAGCTCAAGATTAGTTGAGGGGGGTGCGGGTACAGGGTGAACTGTCTATCCGCCCCTAAGCGGGTCTGTTGCGCACGGAGGAGCCTCGGTAGACTTGGTTATTTTTTCACTACATTTGGCGGGAATGTCTTCTATTCTTGGGGCTATCAACTTCATTACAACAATCTTTAATATGCGGTCCCCCGGGCTAACAATGGAGCGGCTGAGGTTGTTTGTTTGGTCTGTTCTAGTCACGGCTTTTTTACTTCTGCTAAGATTGCCTGTGCTAGCAGGGGCTATCACAATGCTTTTAACTGATCGTAACTTTAACACTAGTTTTTTTGACCCGGCCGGAGGGGGGGACCCTATTTTATACCAACACCTTTTC

>AMC476062001

AACTTTGTACATGGTTTTTGGAGTTTGGGCTGGTCTTGTGGGGACCGGCTTGTCTTTATTAATTCGATTTGAGTTAGGGACGGCAAGAGTTTTTATGGATGAACACTTTTATAATGTGATTGTGACGGCTCATGCTTTTGTTATAATTTTCTTTATGGTTATACCCTTGATGATTGGAGGGTTTGGGAATTGAATAGTCCCTCTGTTAATTGGGGCCCCAGACATAAGGTTTCCACGTATAAACAATATAAGTTTCTGACTACTACCTCCTTCGTTCTTGCTTCTTCTTTGTTCTGCAATGGTTGAAGGAGGAGCTGGAACAGGTTGAACTGTTTACCCTCCTCTTAGTGGACCTATTGCGCATGGTGGGTCTTCTGTTGACTTGGTAATCTTTTCGTTACACTTGGCTGGTATATCTTCCATTTTAGGAGCTATTAACTTTATTACAACTATCTTCAACATACGATCCCCAGGAATGTCTATGGAGCGACTGAATTTATTTGTATGATCAGTTTTAGTTACGGCTTTTTTATTATTATTGAGTTTACCTGTCCTTGCTGGTGCCATTACAATGCTGTTGACTGATCGGAATTTTAATACCAGCTTTTTTGACCCTGCCGGAGGGGGGGATCCTATTTTGTATCAACATCTTTTC

>ZSM20100592

AACTTTATACATAATTTTTGGTGTTTGGTGTGGGTTAGTTGGAACTGGGCTTTCTCTGCTTATTCGATTTGAGCTAGGAACTGCCTCTGTCTTAATAGATGAACATTTTTATAATGTGATTGTTACAGCTCATGCATTTGTCATAATTTTTTTCATAGTTATACCCTTAATAATTGGAGGATTTGGGAATTGAATAGTTCCATTATTAATTGGAGCTGTGGATATAAGCTTTCCACGTATAAATAATATAAGATTTTGATTGCTTCCCCCTTCCTTTATTTTTCTACTGTGTTCATCTATAATTGAAGGAGGTGCTGGAACTGGGTGAACAGTATATCCTCCTCTGAGGGGTCCTATTGCTCATGCTGGGTCTTCAGTCGATCTTGTAATTTTTTCTTTACACTTGGCAGGGATATCTTCTATTTTAGGTGCTATTAATTTTATTACTACTATTTTTAATATACGATCTCCTGGGGTAGGAATAGAACGTCTAAATTTGTTTGTTTGATCTGTATTAGTAACAGCTTTTCTTTTACTTCTAAGACTTCCTGTTTTAGCAGGAGCTATTACTATGCTATTAACTGATCGTAATATTAATACAACATTCTTTGACCCCGCAGGAGGAGGTGACCCTATTTTATACCAACATTTGTTT

>ZSM20081014

AACTTTATATATAATTTTTGGTGTTTGATGTGGATTAGTTGGAACTGGGCTTTCATTACTCATTCGATTTGAGTTAGGGACTGCTTCCGTTTTAATAGACGAGCACTTTTATAATGTGATTGTAACTGCTCATGCATTCGTAATAATTTTTTTTATGGTTATACCCCTAATAATTGGAGGATTTGGAAATTGAATAGTACCTTTATTAATTGGTGCCGTCGATATAAGGTTTCCCCGTATAAATAATATAAGATTCTGGTTACTTCCTCCATCATTTATCTTTCTTCTATGCTCTTCTATAGTCGAAGGAGGGGCTGGGACAGGTTGAACAGTATATCCTCCTTTAAGAGGATCTATTGCTCATGCTGGATCTTCAGTAGATCTAGTAATTTTTTCTCTACATTTAGCAGGTATGTCTTCTATTCTTGGTGCAATTAATTTTATTACTACTATTTTTAATATGCGGTCTCCAGGAATTACCCTAGAACGCTTAAATTTGTTCGTTTGGTCGGTATTGGTAACAGCTTTTCTGTTACTTTTAAGATTACCTGTTTTAGCTGGAGCAATTACTATGTTGTTAACTGATCGTAACATTAATACGACTTTCTTTGATCCTGCAGGAGGAGGGGATCCTATTTTATACCAACACTTATTT

>ZSM20100379

AACTTTATATATAATTTTTGGTGTTTGATGTGGATTAGTTGGAACTGGGCTTTCATTACTCATTCGATTTGAGTTAGGGACTGCTTCCGTTTTAATAGACGAGCACTTTTATAATGTGATTGTAACTGCTCATGCATTCGTAATAATTTTTTTTATGGTTATACCCCTAATAATTGGAGGATTTGGAAATTGAATAGTACCTTTGTTAATTGGTGCCGTCGATATAAGGTTTCCTCGTATAAATAATATAAGATTCTGGTTACTTCCTCCATCATTTATCTTTCTTCTATGCTCTTCTATAGTCGAAGGAGGAGCTGGGACAGGTTGAACAGTATATCCTCCTTTAAGAGGATCTATTGCTCATGCTGGATCTTCAGTAGATCTAGTAATTTTTTCTCTACACTTAGCAGGTATGTCTTCTATTCTTGGTGCAATTAATTTTATTACTACTATTTTTAATATGCGGTCTCCAGGAATCACCCTAGAACGCTTAAATTTGTTCGTTTGATCGGTATTGGTAACAGCTTTTTTGTTACTTTTAAGATTACCTGTTTTAGCTGGAGCAATTACTATGTTGTTAACTGATCGTAACATTAATACGACTTTCTTTGATCCTGCAGGAGGAGGGGATCCTATTTTATACCAACATTTATTT

>ZSM20100595

TACTTTATACATGATTTTTGGGGTATGATGTGGCCTGGTAGGGACTGGTCTATCCCTATTAATTCGTTTCGAACTGGGAACTGCTACAGTTTTTATAGATGAGCACTTTTACAATGTTGTTGTAACCGCTCATGCTTTTGTAATAATTTTTTTTATGGTTATGCCTCTTATGATTGGGGGTTTTGGAAACTGAATAGTTCCTCTACTGATTGGAGCTCCTGATATAAGATTTCCTCGTATGAACAACATAAGTTTTTGACTATTGCCTCCTTCTTTTATTCTTTTGCTGTGCTCTGCTATGGTTGAGGGGGGAGCAGGGACTGGATGGACAGTTTATCCACCTCTTAGAGGCCCAATTGCCCATGGAGGTTCTTCTGTTGACTTAGTTATTTTTTCCCTTCATTTGGCAGGGATGTCTTCTATTTTAGGGGCAATTAATTTTATCACAACTATTTTTAATATACGATCTCCGGGTATAAGAATGGAACGTTTAAATTTGTTTGTTTGATCAGTATTAGTGACTGCCTTTTTGCTTTTACTAAGTTTACCTGTCTTGGCTGGTGCTATCACCATGCTTTTAACCGATCGAAATTTCAACACTAGCTTCTTTGATCCGGCAGGAGGGGGGGATCCTATTTTGTATCAACATCTATTT

>ZSM20100596

TACTTTATACATGATTTTTGGGGTATGATGTGGCCTGGTAGGGACTGGTCTATCCTTATTAATTCGTTTCGAACTAGGAACTGCTACAGTTTTTATAGATGAGCACTTTTACAATGTTGTTGTAACCGCTCATGCTTTTGTAATAATTTTTTTTATGGTTATGCCTCTTATGATTGGGGGTTTCGGAAACTGAATAGTTCCTCTACTGATTGGAGCTCCTGATATAAGATTTCCTCGCATAAACAACATAAGTTTTTGACTGTTGCCTCCTTCTTTTATTCTTTTGCTGTGTTCCGCTATGGTTGAGGGGGGAGCAGGGACTGGATGGACAGTTTATCCGCCTCTTAGAGGCCCAATTGCCCATGGAGGTTCTTCTGTTGACTTAGTTATTTTTTCCCTTCATTTGGCAGGGATGTCTTCTATTTTAGGGGCAATTAATTTTATTACAACTATTTTTAATATACGATCTCCGGGTATAAGAATGGAACGTTTAAATTTGTTTGTTTGATCAGTATTAGTGACTGCCTTTTTGCTTTTACTAAGTTTACCTGTCTTGGCTGGTGCTATTACCATGCTTTTAACCGATCGAAATTTCAACACTAGCTTCTTTGATCCGGCAGGAGGGGGGGATCCTATTTTGTATCAACATCTATTC

>ZSM20100597

TACTTTATACATGATTTTTGGGGTGTGATGTGGCCTGGTAGGGACTGGTCTATCCCTATTAATTCGTTTCGAACTAGGAACTGCTACAGTTTTTATAGATGAGCACTTTTACAATGTTGTTGTAACCGCTCATGCTTTTGTAATAATTTTTTTTATGGTTATGCCTCTTATGATTGGGGGTTTCGGAAACTGAATAGTTCCTCTACTGATTGGAGCTCCTGACATAAGATTTCCTCGTATAAACAACATAAGTTTTTGACTGTTGCCTCCTTCTTTTATTCTTTTGCTGTGTTCCGCTATGGTTGAGGGGGGAGCAGGGACTGGATGGACAGTTTATCCGCCTCTTAGAGGCCCAATTGCCCATGGAGGTTCTTCTGTTGACTTAGTTATTTTTTCCCTTCATTTGGCAGGGATGTCTTCTATTTTAGGGGCAATTAATTTTATCACAACTATTTTTAATATACGATCTCCGGGTATAAGAATGGAACGTTTAAATTTGTTTGTTTGATCAGTATTAGTGACTGCCTTTTTGCTTTTACTAAGTTTACCTGTCTTGGCTGGTGCTATTACCATGCTTTTAACCGATCGAAATTTCAACACTAGCTTCTTTGATCCGGCAGGAGGGGGGGATCCTATTTTGTATCAACATCTATTC

>ZSM20100603

TACTTTATACATGATTTTTGGGGTATGATGTGGCCTGGTAGGGACTGGTCTATCCCTATTAATTCGTTTCGAACTAGGAACTGCTACAGTTTTTATAGATGAGCACTTTTACAATGTTGTTGTAACCGCTCATGCTTTTGTAATAATTTTTTTTATGGTTATGCCTCTTATGATTGGGGGTTTCGGAAACTGAATAGTTCCTCTACTGATTGGAGCTCCTGATATAAGATTTCCTCGTATAAACAACATAAGTTTTTGACTGTTGCCTCCTTCTTTTATTCTTTTGCTGTGTTCCGCTATGGTTGAGGGGGGAGCAGGGACTGGATGGACAGTTTATCCGCCTCTTAGAGGCCCAATTGCCCATGGAGGTTCTTCTGTTGACTTAGTTATTTTTTCCCTTCATTTGGCAGGGATGTCTTCTATTTTAGGGGCAATTAATTTTATCACAACTATTTTTAATATACGATCTCCAGGTATAAGAATGGAACGTTTAAATTTGTTTGTTTGATCAGTATTAGTGACTGCCTTTTTGCTTTTACTAAGTTTACCTGTCTTGGCTGGTGCTATTACCATGCTTTTAACCGATCGAAATTTCAACACTAGCTTCTTTGATCCGGCAGGAGGGGGGGATCCTATTTTGTATCAACATCTATTC

>ZSM20081013

GACTTTGTATATAGTATTTGGTGTATGAGCTGGGTTAGTAGGAACAGGACTTTCTTTGTTAATCCGATTTGAGTTAGGTACAGCATCAGTGTTTATAGATGAGCATTTTTATAATGTCATTGTCACAGCGCATGCCTTTGTAATAATCTTTTTCATAGTAATGCCTCTAATGATTGGGGGTTTTGGGAATTGGATGGTTCCTTTATTAATTGGAGCTCCGGATATAAGGTTTCCTCGAATAAATAATATGAGATTTTGGCTGCTTCCCCCCTCATTTCTGTTACTGTTAAGGTCAGTTATGGTAGAAGGGGGAGCAGGCACAGGTTGGACGGTATACCCTCCTTTGAGGGGCCCAATTGCTCATGGTGGTTCTTCTGTTGATTTGGTAATTTTCTCCTTGCATCTAGCTGGGATGTCTTCTATTTTAGGGGCTATTAACTTTATTACAACTATTTATAACATGCGTTCTCCAGGTATAACAATAGAACGTTTAGATTTATTTGTTTGGTCCGTTCTAGTTACTGCGTTTTTATTACTTTTAAGTCTTCCCGTTCTAGCAGGGGCCATTACCATGCTTTTAACGGATCGGAATTTTAACACTAGCTTTTTTGATCCGGCTGGAGGGGGAGATCCAATTCTATACCAGCATTTGTTT

>SICBC2010KJ01C08

CACTTTGTATATAGTGTTTGGTGTTTGAGCAGGTCTTGTGGGTACAGGTCTGTCTTTATTAATTCGTTTTGAACTAGGAACAGCGTCTGTCTTCATAGACGAGCATTTCTACAATGTTGTTGTCACGGCTCATGCTTTTGTAATAATTTTTTTTATAGTGATGCCTTTAATAATTGGGGGTTTTGGTAACTGAATGGTTCCTTTACTAATCGGGGCTCCTGATATAAGTTTTCCTCGTATAAATAATATAAGTTTTTGGCTACTTCCTCCCTCTTTTGTTTTGTTGTTATGCTCAGCGATAGTAGAAGGAGGCGCTGGAACTGGTTGAACAGTATATCCCCCTCTTAGAGGTCCGGTCGGTCATGGAGGTTCCTCTGTAGATTTGGTGATTTTTTCTCTTCATTTGGCAGGGATATCTTCTATTTTAGGGGCTATTAATTTTATCACTACGATTTTCAACATACGGTCTCCAGGAATAACTATGGAACGATTGAATTTGTTTGTCTGATCAGTTTTAGTTACCGCATTTCTTTTATTATTAAGACTTCCAGTTCTAGCCGGGGCAATTACTATGCTTCTTACAGATCGGAACTTTAATACAAGGTTCTTTGATCCAGCCGGGGGTGGGGACCCTATTTTATATCAACACTTGTTC

>SICBC2010KJ01D05

CACTTTGTATATAGTGTTTGGTGTTTGGGCAGGTCTTGTGGGTACAGGTCTCTCTTTATTAATTCGTTTCGAACTAGGGACAGCGTCTGTCTTCATAGACGAGCATTTCTATAATGTTGTTGTCACGGCTCATGCTTTTGTAATAATTTTTTTTATAGTGATGCCTTTAATAATTGGAGGTTTTGGTAATTGAATGGTTCCTTTATTAATCGGGGCTCCTGATATAAGTTTTCCTCGTATAAACAATATGAGTTTTTGGCTACTTCCTCCCTCTTTTGTTTTGTTGTTATGCTCAGCGATAGTAGAAGGAGGCGCTGGAACTGGTTGAACAGTATACCCTCCTCTTAGAGGTCCGGTTGGTCATGGGGGTTCTTCTGTAGATTTAGTGATTTTTTCTCTTCATTTGGCAGGAATATCTTCTATTTTAGGGGCTATTAATTTTATTACTACGATTTTCAACATACGGTCTCCAGGGATAACTATGGAGCGATTGAATTTATTTGTCTGATCAGTTTTAGTTACCGCATTTCTTTTATTATTAAGACTTCCAGTCTTAGCTGGGGCAATTACTATGCTTCTTACAGATCGGAACTTTAATACAAGGTTCTTTGATCCGGCCGGGGGTGGGGACCCTATTTTATATCAACACTTGTTC

>ZSM20090197

CACTTTGTATATAGTGTTTGGTGTTTGAGCAGGTCTTGTGGGTACAGGTCTGTCTTTATTAATTCGTTTCGAACTAGGAACAGCGTCTGTCTTCATAGACGAGCATTTCTATAATGTTGTTGTCACGGCTCATGCTTTTGTAATAATTTTTTTTATAGTGATGCCTTTAATAATTGGGGGTTTTGGAAATTGAATGGTTCCTTTACTAATCGGGGCTCCTGATATAAGTTTTCCTCGTATAAACAATATAAGTTTTTGGCTACTTCCTCCCTCTTTTGTTTTGTTGTTATGCTCAGCGATAGTAGAAGGAGGCGCTGGAACTGGTTGAACAGTATACCCTCCTCTTAGAGGTCCCGTTGGTCATGGAGGCTCTTCTGTAGATTTGGTGATTTTTTCTCTTCATTTGGCAGGGATATCTTCTATTTTGGGGGCTATCAATTTTATTACTACGATTTTCAATATACGGTCTCCAGGGATAACTATGGAACGATTGAATTTATTTGTCTGATCAGTTTTAGTTACCGCATTTCTTTTATTATTAAGACTTCCAGTCCTAGCTGGGGCAATTACTATGCTTCTTACAGATCGGAACTTTAATACAAGGTTCTTTGATCCAGCCGGGGGTGGGGACCCTATTTTATATCAACACTTGTTC

>ZSM20080176

GACTTTATATATAGTGTTTGGTGTGTGAGCTGGGTTGGTGGGAACTGGCTTATCCTTGTTAATTCGTTTTGAGTTGGGAACAGCATCTGTTTTTATGGATGAGCATTTTTATAATGTAGTTGTCACTGCACATGCCTTTGTCATGATTTTTTTTATAGTTATGCCTCTTATAATTGGGGGGTTCGGAAATTGAATAGTTCCTTTGCTTATTGGTGCTCCCGATATAAGCTTTCCTCGAATAAACAATATAAGATTCTGATTATTGCCGCCCTCATTTATTTTACTTTTATGCTCTGCTATGGTAGAAGGAGGGGCTGGGACTGGGTGAACTGTTTACCCTCCGTTAAGAGGTCCTATTGCCCATGGCAGGTCTTCTGTTGATTTAGTAATTTTTTCTTTACATCTGGCTGGAATGTCTTCAATCTTGGGAGCTATTAATTTTATTACTACCATTTTTAATATACGGTCTCCTGGGATGACTATAGAACGCTTGAATTTATTTGTTTGATCTGTATTAGTGACTGCCTTTTTGCTTTTACTCAGACTTCCTGTTCTTGCTGGGGCTATTACAATGCTTTTAACAGATCGAAACTTTAATACTAGGTTTTTTGATCCTGCTGGGGGCGGGGATCCAATTTTATACCAACATTTGTTT

>ZSM20100388

GACTTTGTATATAGTGTTTGGTGTGTGAGCTGGGTTGGTGGGAACTGGTTTATCCTTGTTAGTTCGTTTTGAGCTGGGAACAGCATCTGTTTTTATGGATGAGCATTTTTATAACGTAGTTGTCACTGCGCATGCCTTTGTTATGATTTTTTTTATAGTTATGCCTCTTATAATTGGAGGGTTCGGAAACTGAATAGTTCCTTTACTTATTGGTGCTCCCGATATAAGCTTTCCTCGAATAAACAATATAAGATTCTGATTATTGCCGCCATCATTTATTTTACTTTTATGTTCTGCTATGGTAGAAGGAGGAGCTGGGACTGGGTGAACTGTCTATCCTCCGTTAAGAGGTCCTATTGCCCATGGCGGATCTTCTGTTGACTTAGTGATTTTTTCTTTACATCTGGCTGGGATATCTTCAATCTTGGGAGCTATTAATTTTATTACTACCATTTTTAATATACGGTCTCCTGGGATGACTATAGAACGCTTGAACTTATTTGTTTGATCTGTATTAGTAACTGCCTTTTTGCTTTTACTTAGACTTCCTGTTCTTGCTGGGGCCATTACAATGCTTTTAACAGATCGAAACTTTAATACTAGGTTTTTTGATCCTGCTGGGGGCGGGGATCCAATTTTATACCAACATTTGTTC

>ZSM20100389

GACTTTGTATATAGTGTTTGGTGTGTGAGCTGGGTTGGTGGGAACTGGTTTATCCTTGTTAGTTCGTTTTGAGCTGGGAACAGCATCTGTTTTTATGGATGAGCATTTTTATAACGTAGTTGTCACTGCGCATGCCTTTGTTATGATTTTTTTTATAGTTATGCCTCTTATAATTGGAGGGTTCGGAAACTGAATAGTTCCTTTACTTATTGGTGCTCCCGATATAAGCTTTCCTCGAATAAACAATATAAGATTCTGATTATTGCCGCCATCATTTATTTTACTTTTATGTTCTGCTATGGTAGAAGGAGGAGCTGGGACTGGGTGAACTGTCTATCCTCCGTTAAGAGGTCCTATTGCCCATGGCGGATCTTCTGTTGACTTAGTGATTTTTTCTTTACATCTGGCTGGGATATCTTCAATCTTGGGAGCTATTAATTTTATTACTACCATTTTTAATATACGGTCTCCTGGGATGACTATAGAACGCTTGAACTTATTTGTTTGATCTGTATTAGTAACTGCCTTTTTGCTTTTACTTAGACTTCCTGTTCTTGCTGGGGCCATTACAATGCTTTTAACAGATCGAAACTTTAATACTAGGTTTTTTGATCCTGCTGGGGGCGGGGATCCAATTTTATACCAACATTTGTTC

>ZSM20100390

GACTTTGTATATAGTGTTTGGTGTGTGAGCTGGGTTGGTGGGAACTGGTTTATCCTTGTTAGTTCGTTTTGAGCTGGGAACAGCATCTGTTTTTATGGATGAGCATTTTTATAACGTAGTTGTCACTGCGCATGCCTTTGTTATGATTTTTTTTATAGTTATGCCTCTTATAATTGGAGGGTTCGGAAACTGAATAGTTCCTTTACTTATTGGTGCTCCCGATATAAGCTTTCCTCGAATAAACAATATAAGATTCTGATTATTGCCGCCATCATTTATTTTACTTTTATGTTCTGCTATGGTAGAAGGAGGAGCTGGGACTGGGTGAACTGTCTATCCTCCGTTAAGAGGTCCTATTGCCCATGGCGGATCTTCTGTTGACTTAGTGATTTTTTCTTTACATCTGGCTGGGATATCTTCAATCTTGGGAGCTATTAATTTTATTACTACCATTTTTAATATACGGTCTCCTGGGATGACTATAGAACGCTTGAACTTATTTGTTTGATCTGTATTAGTAACTGCCTTTTTGCTTTTACTTAGACTTCCTGTTCTTGCTGGGGCCATTACAATGCTTTTAACAGATCGAAACTTTAATACTAGGTTTTTTGATCCTGCTGGGGGCGGGGATCCAATTTTATACCAACATTTGTTC

>ZSM20071135

AACTTTATATATAGTATTTGGTGTGTGAGCTGGGTTGGTGGGAACTGGCTTATCCTTGTTAATTCGTTTTGAGTTGGGAACGGCATCTGTTTTTATGGATGAACATTTTTATAATGTAGTTGTCACTGCGCATGCCTTTGTTATAATTTTTTTTATAGTTATGCCTCTTATAATTGGGGGGTTTGGAAACTGAATAGTTCCTTTACTTATTGGTGCTCCCGATATAAGCTTTCCTCGAATAAATAATATAAGATTCTGATTATTACCGCCATCATTTATTTTACTTTTATGCTCTGCTATGGTAGAAGGAGGAGCTGGGACTGGGTGAACCGTCTATCCTCCGTTAAGAGGTCCTATTGCCCATGGCGGATCTTCTGTTGACTTAGTAATTTTTTCTTTACATCTGGCTGGGATATCTTCAATCTTGGGAGCTATTAATTTTATTACTACCATTTTTAATATACGGTCTCCTGGGATAAATATAGAACGTTTAAACTTATTTGTTTGATCCGTATTAGTGACTGCCTTTTTGCTTTTACTTAGACTTCCTGTTCTTGCTGGGGCCATTACAATGCTTTTAACAGATCGAAACTTTAATACTAGGTTTTTTGATCCTGCTGGGGGTGGGGATCCAATTTTATATCAACATTTGTTC

>ZSM20071820

GACCTTGTATATAGTATTTGGTGTGTTAGCTGGGTTGGTGGGAACTGGCTTATCCTTGTTAATTCGTTTTGAGTTGGGAACAGCATCTGTTTTTATGGATGAGCATTTTTATAATGTAGTTGTCACTGCGCATGCCTTTGTTATGATTTTTTTTATAGTTATGCCTCTTATAATTGGGGGGTTTGGAAACTGAATAGTTCCTTTACTTATTGGTGCCCCCGATATAAGTTTTCCTCGAATAAATAATATAAGATTCTGATTATTGCCGCCCTCATTTATTTTACTTTTATGCTCTGCTATGGTAGAAGGAGGAGCTGGCACTGGGTGAACTGTCTATCCTCCGTTAAGAGGTCCTATTGCCCATGGCGGATCTTCTGTTGACTTAGTAATTTTTTCTTTACATCTGGCTGGGATATCTTCAATCTTGGGAGCTATTAATTTTATTACTACCATTTTTAATATACGGTCTCCTGGGATGACTATAGAGCGCTTGAACTTATTTGTTTGATCCGTATTAGTAACTGCCTTTTTGCTTTTACTTAGACTTCCTGTTCTTGCTGGGGCTATTACAATGCTTTTAACAGATCGAAACTTTAATACTAGGTTTTTTGATCCTGCCGGGGGTGGGGATCCAATTTTATATCAACACTTGTTC

>ZSM20100391

GACCTTATATATAGTATTTGGTGTGTGAGCTGGGTTGGTGGGAACTGGCCTATCCTTGTTGATTCGTTTTGAGTTGGGAACAGCATCTGTTTTTATAGATGAGCATTTTTATAATGTAGTTGTCACTGCGCATGCCTTTGTTATGATTTTTTTTATAGTTATGCCTCTTATAATTGGGGGTTTTGGAAACTGAATAGTTCCTTTACTTATTGGTGCTCCCGATATAAGCTTTCCTCGAATAAATAATATAAGATTCTGATTATTGCCGCCATCATTTATTTTACTTTTATGCTCTGCTATGGTAGAAGGAGGAGCTGGGACTGGGTGAACTGTCTATCCTCCGTTAAGAGGTCCTATCGCCCATGGCGGATCTTCTGTTGACTTAGTAATTTTTTCTTTACATCTGGCTGGGATATCTTCAATCTTGGGAGCTATTAATTTTATTACTACCATTTTTAATATGCGGTCTCCTGGGATGACTATAGAACGCTTGAATTTATTTGTTTGATCCGTATTAGTGACTGCCTTTTTGCTTTTACTTAGACTTCCTGTTCTTGCTGGGGCCATTACAATGCTTTTAACAGATCGAAACTTTAATACTAGGTTTTTTGATCCTGCTGGAGGTGGGGATCCAATTTTATATCAACATTTGTTC
